# Revision of *Telothyria* van der Wulp (Diptera: Tachinidae) and twenty-five new species from Area de Conservación Guanacaste in northwestern Costa Rica with a key to Mesoamerican species

**DOI:** 10.3897/BDJ.8.e47157

**Published:** 2020-04-28

**Authors:** AJ Fleming, D. Monty Wood, M. Alex Smith, Tanya Dapkey, Winnie Hallwachs, Daniel Janzen

**Affiliations:** 1 Agriculture and Agri-Food Canada, Ottawa, Canada Agriculture and Agri-Food Canada Ottawa Canada; 2 University of Guelph, Guelph, Canada University of Guelph Guelph Canada; 3 University of Pennsylvania, Philadelphia, United States of America University of Pennsylvania Philadelphia United States of America; 4 University of Pennsylvania, Philadelphia, United States of America University of Pennsylvania Philadelphia United States of America

**Keywords:** tropical rain forest, tropical dry forest, cloud forest, parasitoid flies, host-specificity, caterpillars, ACG, Dexiinae, Telothyriini

## Abstract

**Background:**

We describe 25 new species in the genus *Telothyria* van der Wulp, 1890 from Area de Conservación Guanacaste (ACG) in northwestern Costa Rica. All species herein described were reared from an ongoing inventory of wild-caught caterpillars spanning two families (Lepidoptera: Crambidae, and Tortricidae). Our study provides a concise description of each new species using morphology, life history, molecular data, and photographic documentation; a redescription of the genus, and its type species as well as a revised key to species of *Telothyria* occurring in the Mesoamerican region. We also suggest seven new synonymies resulting in 11 new combinations.

**New information:**

The following 25 new species of *Telothyria* are described: *T. aidani* **sp. n.**, *T. alexanderi* **sp. n.**, *T. auranticrus* **sp. n.**, *T. auriolus* **sp. n.**, *T. bicuspidata* **sp. n.**, *T. carolinacanoae* **sp. n.**, *T. clavata* **sp. n.**, *T. cristata* **sp. n.**, *T. diniamartinezae* **sp. n.**, *T. duniagarciae* **sp. n.**, *T. duvalierbricenoi* **sp. n.**, *T. eldaarayae* **sp. n.**, *T. erythropyga* **sp. n.**, *T. fimbriata* **sp. n.**, *T. fulgida* **sp. n.**, *T. gloriashihezarae* **sp. n.**, *T. grisea* **sp. n.**, *T. harryramirezi* **sp. n.**, *T. incisa* **sp. n.**, *T. manuelpereirai* **sp. n.**, *T. obscura* **sp. n.**, *T. omissa* **sp. n.**, *T. osvaldoespinozai* **sp. n.**, *T. peltata* **sp. n.**, and *T. ricardocaleroi* **sp. n.**

The following are proposed by Fleming & Wood as new generic synonyms of *Telothyria*: *Comatacta* Coquillett **Syn. n.**, *Floradalia* Thompson **Syn. n.**, *Ptilomyia* Curran **Syn. n.**, *Ptilomyiopsis* Townsend **Syn. n.**, *Ptilomyoides* Curran **Syn. n.**, *Euptilomyia* **Syn. n.**, *Eutelothyria* Townsend **Syn. n.**

The following new combinations are proposed as a result of the new synonymies: *Telothyria bequaerti* (Curran, 1925) **Comb. n.**, *Telothyria cruenta* (Giglio-Tos, 1893) **Comb. n.**, *Telothyria frontalis* (Townsend, 1939) **Comb. n.**, *Telothyria insularis* (Curran, 1927) **Comb. n.**, *Telothyria itaquaquecetubae* (Townsend, 1931) **Comb. n.**, *Telothyria major* (Thompson, 1963) **Comb. n.**, *Telothyria micropalpus* (Curran, 1925) **Comb. n.**, *Telothyria minor* (Thompson, 1963) **Comb. n.**, *Telothyria nautlana* (Townsend, 1908) **Comb. n.**, *Telothyria plumata* (Curran, 1925) **Comb. n.**, *Telothyria trinitatis* (Thompson, 1963) **Comb. n.**, *Telothyria variegata* (Fabricius, 1805) **Comb. n.** *Musca tricincta* Fabricius is synonymized under *Telothyria variegata* Fabricius, **Syn. n.**

*Telothyria schineri* Fleming & Wood **nom. n.** is proposed as a replacement name for *Miltogramma brevipennis* Schiner.

Additionally we provide redescriptions of two previously named species: the type species *Telothyria cupreiventris* (van der Wulp) due to its being the type species, and *Telothyria relicta* (van der Wulp) due to its having been reared as an outcome of the inventory.

## Introduction

**Figure 1.**
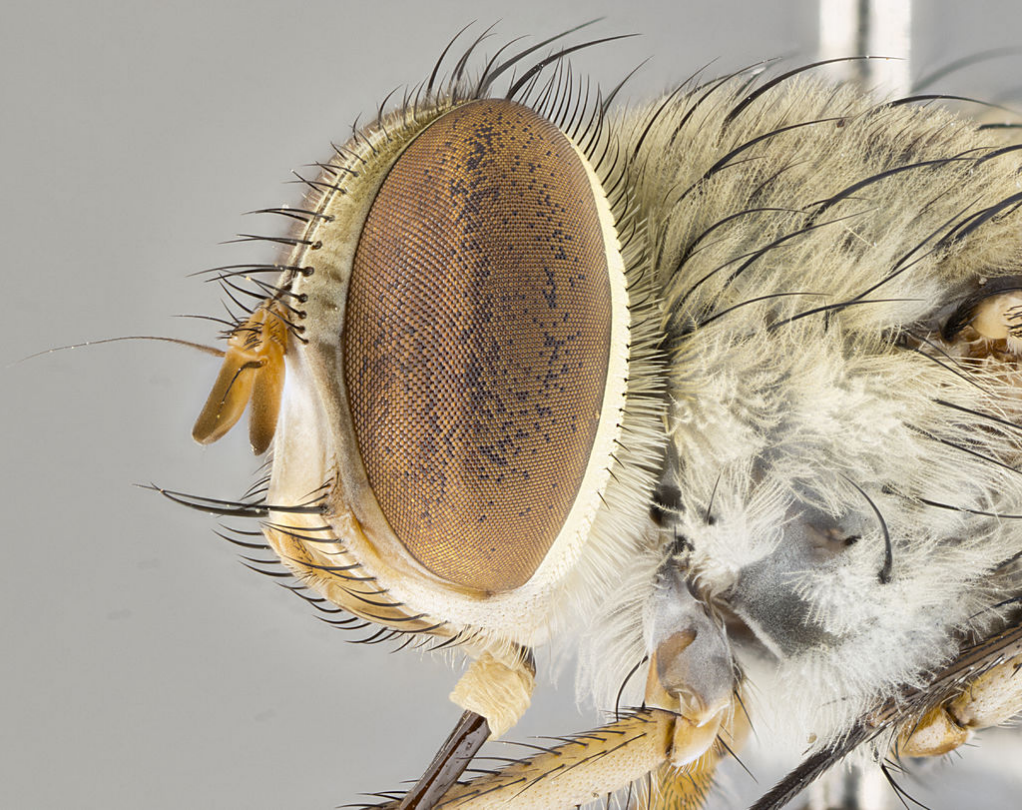
Detail of thorax and head of *Telothyria relicta* van der Wulp, 1890 displaying the characteristic plumose hairs that cover the surfaces of the thorax. These hairs are unique to this genus, within the family Tachinidae.

The Neotropical genus *Telothyria* van der Wulp (1890) was erected to include 38 nominal species. The original work, however, was vague in its concept of the genus, lacking even a designation of a type species. The following year, Brauer and Bergenstamm (1891) fixed *Telothyria cupreiventris* van der Wulp as the type species, thereby restricting the generic concept. While no subsequent work focused on *Telothyria* as a genus, a number of species were added and removed as the years passed. With the publication of Wood (1985)'s revision of the Blondeliini of North and Central America, the genus *Telothyria* *sensu* van der Wulp underwent an enormous upheaval (because most of the original species were blondeliines). Wood's revision resulted in 177 new generic synonyms and 321 new combinations. Among these new synonymies, and combinations, Wood (1985) reviewed and repositioned many of the species originally included in the Telothyriini. Thirty-three of van der Wulp's original *Telothyria* were transferred as new combinations or synonyms into the Blondeliini, leaving nine species of *Telothyria*, with only five of these derived from the original paper. No further work on *Telothyria* has been published since the changes proposed by Wood (1985). The tribe Telothyriini presently includes eight genera with a combined total of 21 species among them. One of the simplest and most easily recognizable traits of the tribe is the presence of long blonde to reddish-copper plumose thoracic hairs (Fig. 1). This paper provides a comprehensive review of the genus, including seven new synonymies, a generic redescription, a description of 25 new species from Area de Conservación Guanacaste (ACG), and a key to the identification of the Mesoamerican species.

Our goal is to systematically revise and analyze the known members of the New World tachinid genus *Telothyria* van der Wulp (Dexiinae: Telothyriini). The tribe Telothyriini is distributed almost exclusively within the Neotropical region, except for one species from North America. The vast tachinid fauna of the Neotropical region, along with the huge number of genera, has proven to be one of the largest hurdles to understanding the generic boundaries within the Telothyriini, and properly understanding Neotropical Tachinidae.

All flies and rearing information described here derive from the ongoing inventory of the tri-trophic relationships between caterpillars, their food plants and their parasitoids within the dry, rain, and cloud forests of the terrestrial portion of ACG (Janzen et al. 2009, Janzen and Hallwachs 2011, Fernandez-Triana et al. 2014). Since 1978 this inventory has yielded an unprecedented amount of invaluable information on the tri-trophic relationships between parasitoids, hosts, and associated food plants.

Our descriptions of these 25 new species of *Telothyria* are based on differences in external morphology, COI (coxI or cytochrome *c* oxidase I) gene sequences, and male terminalia (whenever possible). As the inventory is continually growing, this paper should not be taken as an indication of the final total number of species of *Telothyria* present in Costa Rica or even within ACG. While our key is comprehensive across the Mesoamerican range (also inclusive of several species from the Antilles), our descriptions are limited to a redescription of the type species, and the species known and reared from ACG to date. This paper on *Telothyria* is part of a larger effort to describe new species reared during the ACG inventory (Fleming et al. 2014, Fleming et al. 2014, Fleming et al. 2015c, Fleming et al. 2015a, Fleming et al. 2015b, Fleming et al. 2015d, Fleming et al. 2016a, Fleming et al. 2016b, Fleming et al. 2017). This series of taxonomic papers will represent a baseline for later, detailed ecological and behavioral accounts and studies extending across ACG ecological groups, whole ecosystems, and taxonomic assemblages much larger than a genus.

## Materials and methods

### Project aims and rearing intensity

All reared specimens were obtained from host caterpillars collected in ACG (Smith et al. 2006, Smith et al. 2007, Smith et al. 2008, Janzen et al. 2009, Smith et al. 2009, Janzen and Hallwachs 2011, Rodriguez et al. 2012, Smith et al. 2012, Fleming et al. 2014, Janzen and Hallwachs 2016). ACG's 125,000+ terrestrial hectares span the provinces of Alajuela and Guanacaste, along the dry forested northwestern coast of Costa Rica and inland to the Caribbean lowland rain forest. ACG comprises several different biomes and intergrades, ranging from sea level up to 2,000 m. The tachinid rearing methods are described at http://janzen.bio.upenn.edu/caterpillars/methodology/how/parasitoid_husbandry.htm. Since its inception, this inventory has reared over 750,000 wild-caught ACG caterpillars. Any frequencies of parasitization reported here need to be considered against this background inventory. Comparative details of the parasitization ecology of these flies will be treated separately in later papers, in the context of the study of all parasitization rates of tachinids on ACG caterpillars, once the overall alpha taxonomy of ACG caterpillar-attacking tachinids is more complete than at present.

### Voucher specimen management

The management of voucher specimens has been detailed in previous papers in this series (Fleming et al. 2014). In brief, all caterpillars reared from the ACG efforts receive a unique voucher code in the format yy–SRNP–xxxxx. Any parasitoid emerging from a caterpillar receives the same voucher code as a record of the rearing event. If and when the parasitoid is later dealt with individually it receives a second voucher code unique to it, in the format DHJPARxxxxxxx. These voucher codes assigned to both host and parasitoids may be used to obtain the individual rearing record at http://janzen.bio.upenn.edu/caterpillars/database.lasso.

To date, all DHJPARxxxxxx-coded tachinids have had one leg removed for DNA barcoding at the Biodiversity Institute of Ontario (BIO) in Guelph, ON, Canada. All successful barcodes and collateral data are first deposited in the Barcode of Life Data System (BOLD, www.boldsystems.org) (Ratnasingham and Hebert 2007), and later migrated to GenBank. Each barcoded specimen is also assigned unique accession codes from both the Barcode of Life Data System (BOLD) and GenBank respectively.

Inventoried Tachinidae were collected under Costa Rican government research permits issued to DHJ, and exported from Costa Rica to Philadelphia, en route to their final depository in the Canadian National Insect collection in Ottawa, Canada (CNC). Tachinid identifications for the inventory were done by DHJ in coordination with a) visual inspection by AJF and DMW, b) DNA barcode sequence examination by MAS and DHJ, and c) correlation with host caterpillar identifications by DHJ and WH through the inventory itself. Dates of collection cited for each ACG specimen are the dates of eclosion of the fly, not the date of capture of the caterpillar since the fly eclosion date is much more representative of the time when that fly species is on the wing than is the time of capture of the host caterpillar. The collector listed on the label is the parataxonomist who found the caterpillar, rather than the person who retrieved the newly eclosed fly from its rearing container. Unless otherwise noted the primary type material of the species newly described herein are all deposited in the CNC.

### Descriptions and imaging

Species accounts and descriptions are complemented with a series of color photos of every species, used to illustrate the morphological differences among them. The morphological terminology used follows Cumming and Wood (2009). All dissections and photography were carried out following the methods detailed by Fleming et al. (2014). If only one male was available, it was designated as the holotype and not subjected to dissection. Landmark body structures, measurements and examples of parts of the terminalia are illustrated in Fig. 2.

### Interim names of undescribed host species

Names of undescribed host species follow a standardized, interim naming system used for taxonomic units considered as distinct species and identified by DNA barcodes. The interim names are given in the format "*Phostria* Janzen52" or "*Desmia benealis*DHJ03", where the "species epithet" is either composed of the name of the taxonomist who identified the species and a number or the name of a species-group followed by a code. This prevents confusion with already described species while maintaining traceability of each undescribed species within the ACG project.

### DNA Barcoding

DNA barcodes sequences derived from a standardized 5’ region of the mitochondrial cytochrome c oxidase I (COI) gene were obtained for all ACG inventoried specimens using DNA extracts prepared from single legs using a modified glass fibre protocol (Ivanova et al. 2006). A 658-bp region near the 5’ terminus of the COI gene was amplified from the total genomic DNA extract using standard insect primers (LepF1–LepR1 and following established protocols (Smith et al. 2007, Smith et al. 2006, Smith et al. 2008). All information for the sequences associated with each individual specimen (including GenBank and BOLD accession) can be retrieved from the Barcode of Life Data System (BOLD) (Ratnasingham and Hebert 2007) via the public dataset: http://dx.doi.org/10.5883/DS-ASTELOTH.

### Acronyms for depositories

AMNH, American Museum of Natural History, New York, New York, USA

BMNH, The Natural History Museum, London, United Kingdom

CNC, Canadian National Collection of Insects, Arachnids and Nematodes, Ottawa, Canada

MRSN, Museo Regionale di Scienze Naturali di Torino (collection formerly housed at Museo di Zoologia, Istituto di Zoologia e Anatomia Comparata, Università di Torino - MZUT), Turin, Italy

NHMW, Naturhistorisches Museum Wien, Vienna, Austria

USNM, United States National Museum of Natural History, Washington, D.C., USA

ZMUC, Natural History Museum of Denmark, Zoological Museum, Copenhagen, Denmark

## Taxon treatments

### Telothyria

van der Wulp, 1890

95B4FE67-0487-5BED-AA7D-B795A869C8CB

Telothyria van der Wulp, 1890: 44, [also 1890: 167]. Type species: *Telothyria cupreiventris* van der Wulp, 1890, by subsequent designation of Brauer & Bergenstamm (1891: 378 [also 1891: 74]).
Thereuops Brauer & Bergenstamm, 1891: 378 [also 1891: 74]. Type species: *Miltogramma brevipennis* Schiner, 1868 (preocc. by *Miltogramma brevipennis* Bigot, 1861), by subsequent designation of Brauer & Bergenstamm (1891: 378 [also 1891: 74]). Synonymy proposed by Aldrich 1929 :7. [see below under *Telothyria schineri* Fleming & Wood, **nom. n.**]
Therevops . Incorrect subsequent spelling of *Thereuops* Brauer & Bergenstamm, 1891 (Aldrich 1929: 7, 33).
Thelothyria . Incorrect subsequent spelling of *Telothyria* van der Wulp, 1890 (Brauer & Bergenstamm 1893: 132 [also 1893: 44]).
Comatacta Coquillett, 1902: 199. Type species: *Brachycoma pallidula* van der Wulp, 1890 (= *Stomoxys variegata* Fabricius, 1805), by original designation. **Syn. n.**
Leskiopsis Townsend, 1916: 627. Type species: *Myiobia thecata* Coquillett, 1895, by original designation. Synonymy proposed by Wood and Zumbado 2010: 1412.
Ptilomyia Curran, 1925: 8 (preocc. by *Ptilomyia* Coquillett, 1910). Type species: *Ptilomyia plumata* Curran, 1925, by original designation. Synonymy proposed by Curran 1928: 112.
Ptilomyoides Curran, 1928: 112. Type species: *Ptilomyia becquaerti* Curran, 1925, by monotypy. **Syn. n.**
Eutelothyria Townsend, 1931: 332. Type species: *Eutelothyria itaquaquecetubae* Townsend, 1931, by original designation. **Syn. n.**
Ptilomyiopsis Townsend, 1933: 527 (*nomen novum* for *Ptilomyia* Curran). Type species: *Ptilomyia plumata* Curran, 1925, by designation of the same species for *Ptilomyia* Curran, 1925. [Curran 1928 proposed the synonymy of *Ptilomyia* Curran, 1925 with *Comatacta* Coquillett, 1902. Despite this proposed synonymy Townsend 1933 proposed a replacement name for *Ptilomyia* Curran, erected on the basis of *Ptilomyia plumata* Curran, 1925 which Townsend considered to be generically distinct from *Comatacta*; junior synonym of *Comatacta* Coquillett, 1902 [*teste Curran 1928*: 112]]. **Syn. n.**
Euptilomyia Townsend, 1939: 451. Type species: *Euptilomyia frontalis* Townsend, 1939, by original designation. **Syn. n.**
Floradalia Thompson, 1963: 486. Type species: *Floradalia major* Thompson, 1963, by original designation. **Syn. n.**
Telothyria **Other species included in *Telothyria* Robineau-Desvoidy**
bequaerti Curran, 1925: 352 (*Ptilomyia*). Holotype male (AMNH), by original designation. Type locality: Brazil, Roraima, San Alberto. [Type locality cited in Curran (1928) as Honduras in error] **Comb. n.**
Telothyria *brasiliensisLeskiopsis*
cruenta Giglio-Tos, 1893: 3 (*Chaetona*). Holotype female (MRSN), by original designation. Type locality: Mexico. **Comb. n.**
cupreiventris van der Wulp, 1890: 169 in key [1890: 182, description] (*Telothyria*). Lectotype male [not female as published, Townsend 1931: 91] (BMNH), by fixation of Townsend 1931: 91. Type locality: Mexico, Tabasco, Teapa.
frontalis Townsend, 1939: 451 (*Euptilomyia*). Syntypes, 2 males (USNM), by original designation. Type locality: Brazil, São Paulo, Juquía [cited in Guimarães 1971: 121 as Itaquaquecetuba]. **Comb. n.**
Telothyria *illucensTelothyria*
insularis Curran, 1927: 12 (*Comatacta*). Holotype male (AMNH), by original designation. Type locality: Puerto Rico, San Juan. **Comb. n.**
itaquaquecetubae Townsend, 1931: 333 (*Eutelothyria*). Holotype male (USNM), by original designation. Type locality: Brazil, São Paulo, Itaquaquecetuba **Comb. n.**
major Thompson, 1963: 486 (*Floradalia*). Holotype female (CNC), by original designation. Type locality: Trinidad, Maracas Valley. **Comb. n.**
micropalpus Curran, 1925: 9 (*Ptilomya*). Holotype male (AMNH), by original designation. Type locality: Brazil, “Piedra Blanca” (as “Chapada”, in error according to Arnaud 1963: 126). **Comb. n.**
minor Thompson, 1963: 488 (*Floradalia*). Holotype male (CNC), by original designation. Type locality: Trinidad, St. Augustine. **Comb. n.**
nautlana Townsend, 1908: 101 (*Comatacta*). Holotype male [sex not given in original description, determined from holotype examination] (USNM), by original designation. Type locality: Mexico, Veracruz, San Rafael, Jicaltepec. **Comb. n.**
Telothyria *placidaTelothyria*
plumata Curran, 1925: 8 (*Ptilomya*). Lectotype male (AMNH), designated by Arnaud (1963). Type locality: Brazil, Mato Grosso, "Chapada" [probably in or near present-day Parque Nacional da Chapada dos Guimarães]. **Comb. n.**
relicta van der Wulp, 1890: 171 (*Telothyria*). Holotype female (BMNH). Type locality: Mexico, Veracruz, Atoyac.
Telothyria *rufopygataVivianaV.? rufopygata*
Telothyria *rufostriataTelothyria*
schineri Fleming & Wood, **nom. n.** for *Miltogramma brevipennis* Schiner, 1869
brevipennis Schiner, 1868: 324 (*Miltogramma*). Holotype male (NHMW). Type locality: Brazil. Junior primary homonym of *Miltogramma brevipennisBigot 1861*. [*Miltogramma brevipennis*Schiner 1868 is a junior primary homonym of *Miltogramma brevipennis* Bigot, 1861 a valid name within the Sarcophagidae. The authors hereby propose the replacement name *Telothyria schineri* for *Miltogramma brevipennis* Schiner. The type material originally referenced by Schiner is conserved, with the specific epithet being selected in honor of Ignaz Rudolph Schiner.]
thecata Coquillett, 1895: 105 (*Myiobia*). Lectotype male (USNM), by fixation of Townsend in Townsend 1939: 250 (mention of “Ht male” from Bucks and Delaware counties in USNM is regarded as a lectotype fixation). Type locality: USA, Pennsylvania, Bucks County.
trinitatis Thompson, 1963: 484 (*Eutelothyria*). Syntypes males and females (1 male in CNC), by original designation. Type locality: Trinidad, Brazil (village name). **Comb. n.**
variegata Fabricius, 1805: 281 (*Stomoxys*). Holotype male (ZMUC), by original designation. Type locality: South America. **Comb. n.**
tricincta Fabricius, 1805: 301 (*Musca*). Holotype female (ZMUC). Type locality: South America. **Syn. n.**
pallidula van der Wulp, 1890: 95 (*Brachycoma*). Holotype male (BMNH). Type locality: Mexico, North Yucatan, Temax.
Telothyria Telothyria cupreiventris van der Wulp, 1890: 169. by subsequent designation

#### Description

**Male. Head**: frons narrow 1/10-1/8 of head width; 1–4 reclinate orbital setae; anteriormost reclinate orbital seta distinctly longer than uppermost frontal seta; ocellar setae most often absent, if present then these appearing short and underdeveloped, easily confused with vertical setulae arising behind anterior ocellus; eye bare, ventral margin below level of vibrissa; fronto-orbital plate ranging from shining silver or gold to brownish with a silver sheen; fronto-orbital plate with short black or blonde hairs interspersed among frontal setae; fronto-orbital plate with setae not extending below lower margin of pedicel; lower margin of face slightly lower than vibrissa almost not visible in profile; facial ridge bare in most species, the few exceptions possessing yellow almost inconspicuous hairs along margin; palpus either straight or with a slight club at apex, sparsely haired; arista ranging from bare to plumose, usually distinctly-thickened on basal 1/2, ranging in color from orange to dark brown-black. **Thorax**: gray to golden tomentose over a black to reddish-brown ground color; thorax covered in dense plumose blonde hairs or plumose hairs confined to lateral surfaces with disc of scutum covered in thin black hairs; prosternum bare; chaetotaxy: one proepimeral seta; one proepisternal seta; 4–5 postpronotal setae, basal setae arranged in a straight line; supra-alar setae 1–2:3; intra-alar setae 1–2:2–3; dorsocentral setae 3–4:3–4; acrostichal setae 3–4:3–4; katepisternum with 2–3 setae; meral setae usually absent in the traditional sense instead meral row replaced by a fan of long plumose hairs (Fig. 2c). Scutellum with three pairs marginal setae; apical scutellar setae crossed apically, 1/8–1/10th as long as subapical scutellars; basal scutellar setae equal in length to subapical setae, often slightly shorter; subapical setae straight, ranging from divergent to convergent; ranging from gray to golden pollinose. **Legs**: ranging in ground color from yellow to dark reddish-brown; coxae covered in dense plumose blonde hairs. **Wing**: slightly longer than abdomen; translucent slightly hyaline; all veins bare, with 1–2 setula at base of vein R_4+5_; apical cell open at or just before the apex of wing; bend of vein M obtuse-angled. **Abdomen**: ground color ranging from a deep maroon, to different tonalities of yellow-orange with longitudinal middorsal brown markings; middorsal depression on syntergosternite 1+2 (ST1+2) reaching to hind margin of tergite; median marginal setae present only on tergite 4 (T4) and tergite 5 (T5) (one exception *Telothyria omissa* **sp. n.**, which lacks the marginal setae on tergite 4 (T4)); median discal setae absent on ST1+2–T4, occasionally present on T5; sex patch absent. **Male terminalia**: Sternite 5 with median cleft ranging from deeply excavated and smoothly V-shaped, to shallow and only slightly separated; margins either bare or covered in dense pollinosity; lateral lobes of sternite either sharply pointed, rounded apically or squared, sometimes with a small group of strong setulae along outer margins; basal section of sternite 5 subequal to slightly longer than length of apical lobes. Cerci in posterior view sharply pointed and triangular typically with a well defined basal shoulder separating upper lobe from apical section, ranging from slightly shorter to subequal in length of surstyli, fused along entire length; in lateral view, with a strong downward curve on apical 1/3, and several strong widely spaced setae along basal 2/3. Surstylus in lateral view, almost equilateral along its length, rounded at tip, sometimes slightly pinched at midpoint appearing digitiform, appearing fused with epandrium, when viewed dorsally straight and slender or with a slight sinusoidal curve, parallel at apices. Distiphallus either long and slender or short and stout, ranging from 1.5X to 2X as long as basiphallus and tubular, weakly tapering apically. Distiphallus, hinged at a strong acute angle with basiphallus, a synapomorphy of the Dexiinae.

**Female** as in male except in the following aspects: head: bearing 2–3 pairs of proclinate orbital setae, as well as 2–3 pairs of reclinate inner orbital setae; one pair of outer vertical setae present; thorax: meron bearing either typical meral setae not plumose blonde hairs as in male (Fig. 2d) or a mix of both plumose blonde hairs and regular setae (Fig. 2e); legs: can display dimorphic coloration from males; abdomen: slightly more globose than males, coloration of the abdomen can be dimorphic between the sexes; female terminalia were not dissected, however external examination showed these to be unspecialized.

#### Diagnosis

*Telothyria* can be recognized most easily by the presence of long plumose hairs covering more than 50% of the thoracic surfaces, a trait that was historically used to unify the genera within the tribe. In males of the genus, and many of the females, the meral setae are also replaced with these plumose hairs. Characters of note within *Telothyria* are: prosternum bare; fronto-orbital plate haired; parafacial bare; arista ranging from plumose to bare; ocellar setae weakly developed or absent; eye bare; females of all species with two pairs of well-developed proclinate orbital setae, absent in males; first postsutural supra-alar seta poorly developed in length at most 0.5X second postsutural supra-alar; the three major setae of the postpronotum arranged in a straight line; most of the thorax covered in plumose blonde or coppery hairs (some species lack these setae dorsally) (Fig. 1); wings lacking costal spine. Abdomen with median marginal setae only on T4 and T5 (exception *Telothyria omissa* **sp. n.**), and discal setae absent.

#### Distribution

From southeastern USA west to Mexico and south to Brazil.

#### Ecology

Within the ACG inventory *Telothyria* has been reared from two families of lepidopteran hosts throughout the diverse ecosystems of the research area, Crambidae, and Tortricidae. Guimarães (1977), suggested *Spodoptera* sp. of the family Noctuidae, however he failed to identify the species of *Telothyria* and as such casts a doubt on this potential host.

#### Taxon discussion

Based on our observations of the apomorphies shared by the species assigned to the tribe Telothyriini, expressed as a result described herein, we propose the synonymy of all the genus-group names listed above within the tribe Telothyriini. Most recently, it has been suggested that the Telothyriini are a phylogenetically nested sub-clade within the Dexiinae (Stireman et al. 2019); this evidence, is still the subject of discussion, as the reconstruction of the Dexiinae is still unclear. So, for the sake of continuity, taking into account all the available evidence, and given the remarkable difference between *Telothyria* and other genera within the Dexiinae, the authors have chosen to maintain the Telothyriini as a monotypic tribe, until further examination is conducted to clarify its classification.

### Telothyria aidani

Fleming & Wood sp. n.

4354D415-661A-5196-AF45-12E0BDE7D12C

urn:lsid:zoobank.org:act:72A5107B-0D06-4B50-AB9B-8633A4D89624

#### Materials

- **Type status:** Holotype. **Occurrence:** occurrenceDetails: http://janzen.sas.upenn.edu; catalogNumber: DHJPAR0010393; recordedBy: D.H. Janzen, W. Hallwachs & Lucia Vargas; individualID: DHJPAR0010393; individualCount: 1; sex: M; lifeStage: adult; preparations: pinned; otherCatalogNumbers: ASTAS224-06, 06-SRNP-17596, BOLD:AAD4191; **Taxon:** scientificName: Telothyria aidani; phylum: Arthropoda; class: Insecta; order: Diptera; family: Tachinidae; genus: Telothyria; specificEpithet: aidani; scientificNameAuthorship: Fleming & Wood, 2018; **Location:** continent: Central America; country: Costa Rica; countryCode: CR; stateProvince: Guanacaste; county: Sector Horizontes; locality: Area de Conservacion Guanacaste; verbatimLocality: Vado Esperanza; verbatimElevation: 85; verbatimLatitude: 10.7894; verbatimLongitude: -85.551; verbatimCoordinateSystem: Decimal; decimalLatitude: 10.7894; decimalLongitude: -85.551; **Identification:** identifiedBy: AJ Fleming; dateIdentified: 2018; **Event:** samplingProtocol: Reared from the larva of the Crambidae, Spoladea recurvalis; verbatimEventDate: 18-Jul-2006; **Record Level:** language: en; institutionCode: CNC; collectionCode: Insects; basisOfRecord: Pinned Specimen
- **Type status:** Paratype. **Occurrence:** occurrenceDetails: http://janzen.sas.upenn.edu; catalogNumber: DHJPAR0010392; recordedBy: D.H. Janzen, W. Hallwachs & Lucia Vargas; individualID: DHJPAR0010392; individualCount: 1; sex: F; lifeStage: adult; preparations: pinned; otherCatalogNumbers: ASTAS223-06, 06-SRNP-17612, BOLD:AAD4191; **Taxon:** scientificName: Telothyria aidani; phylum: Arthropoda; class: Insecta; order: Diptera; family: Tachinidae; genus: Telothyria; specificEpithet: aidani; scientificNameAuthorship: Fleming & Wood, 2018; **Location:** continent: Central America; country: Costa Rica; countryCode: CR; stateProvince: Guanacaste; county: Sector Horizontes; locality: Area de Conservacion Guanacaste; verbatimLocality: Vado Esperanza; verbatimElevation: 85; verbatimLatitude: 10.7894; verbatimLongitude: -85.551; verbatimCoordinateSystem: Decimal; decimalLatitude: 10.7894; decimalLongitude: -85.551; **Identification:** identifiedBy: AJ Fleming; dateIdentified: 2018; **Event:** samplingProtocol: Reared from the larva of the Crambidae, Spoladea recurvalis; verbatimEventDate: 19-Jul-2006; **Record Level:** language: en; institutionCode: CNC; collectionCode: Insects; basisOfRecord: Pinned Specimen
- **Type status:** Paratype. **Occurrence:** occurrenceDetails: http://janzen.sas.upenn.edu; catalogNumber: DHJPAR0010311; recordedBy: D.H. Janzen, W. Hallwachs & Lucia Vargas; individualID: DHJPAR0010311; individualCount: 1; sex: M; lifeStage: adult; preparations: pinned; otherCatalogNumbers: ASTAS142-06, 06-SRNP-17508, BOLD:AAD4191; **Taxon:** scientificName: Telothyria aidani; phylum: Arthropoda; class: Insecta; order: Diptera; family: Tachinidae; genus: Telothyria; specificEpithet: aidani; scientificNameAuthorship: Fleming & Wood, 2018; **Location:** continent: Central America; country: Costa Rica; countryCode: CR; stateProvince: Guanacaste; county: Sector Horizontes; locality: Area de Conservacion Guanacaste; verbatimLocality: Vado Esperanza; verbatimElevation: 85; verbatimLatitude: 10.7894; verbatimLongitude: -85.551; verbatimCoordinateSystem: Decimal; decimalLatitude: 10.7894; decimalLongitude: -85.551; **Identification:** identifiedBy: AJ Fleming; dateIdentified: 2018; **Event:** samplingProtocol: Reared from the larva of the Crambidae, Spoladea recurvalis; verbatimEventDate: 18-Jul-2006; **Record Level:** language: en; institutionCode: CNC; collectionCode: Insects; basisOfRecord: Pinned Specimen
- **Type status:** Paratype. **Occurrence:** occurrenceDetails: http://janzen.sas.upenn.edu; catalogNumber: DHJPAR0010395; recordedBy: D.H. Janzen, W. Hallwachs & Lucia Vargas; individualID: DHJPAR0010395; individualCount: 1; sex: F; lifeStage: adult; preparations: pinned; otherCatalogNumbers: ASTAS226-06, 06-SRNP-17532, BOLD:AAD4191; **Taxon:** scientificName: Telothyria aidani; phylum: Arthropoda; class: Insecta; order: Diptera; family: Tachinidae; genus: Telothyria; specificEpithet: aidani; scientificNameAuthorship: Fleming & Wood, 2018; **Location:** continent: Central America; country: Costa Rica; countryCode: CR; stateProvince: Guanacaste; county: Sector Horizontes; locality: Area de Conservacion Guanacaste; verbatimLocality: Vado Esperanza; verbatimElevation: 85; verbatimLatitude: 10.7894; verbatimLongitude: -85.551; verbatimCoordinateSystem: Decimal; decimalLatitude: 10.7894; decimalLongitude: -85.551; **Identification:** identifiedBy: AJ Fleming; dateIdentified: 2018; **Event:** samplingProtocol: Reared from the larva of the Crambidae, Spoladea recurvalis; verbatimEventDate: 20-Jul-2006; **Record Level:** language: en; institutionCode: CNC; collectionCode: Insects; basisOfRecord: Pinned Specimen
- **Type status:** Paratype. **Occurrence:** occurrenceDetails: http://janzen.sas.upenn.edu; catalogNumber: DHJPAR0010396; recordedBy: D.H. Janzen, W. Hallwachs & Lucia Vargas; individualID: DHJPAR0010396; individualCount: 1; sex: M; lifeStage: adult; preparations: pinned; otherCatalogNumbers: ASTAS227-06, 06-SRNP-17550, BOLD:AAD4191; **Taxon:** scientificName: Telothyria aidani; phylum: Arthropoda; class: Insecta; order: Diptera; family: Tachinidae; genus: Telothyria; specificEpithet: aidani; scientificNameAuthorship: Fleming & Wood, 2018; **Location:** continent: Central America; country: Costa Rica; countryCode: CR; stateProvince: Guanacaste; county: Sector Horizontes; locality: Area de Conservacion Guanacaste; verbatimLocality: Vado Esperanza; verbatimElevation: 85; verbatimLatitude: 10.7894; verbatimLongitude: -85.551; verbatimCoordinateSystem: Decimal; decimalLatitude: 10.7894; decimalLongitude: -85.551; **Identification:** identifiedBy: AJ Fleming; dateIdentified: 2018; **Event:** samplingProtocol: Reared from the larva of the Crambidae, Spoladea recurvalis; verbatimEventDate: 20-Jul-2006; **Record Level:** language: en; institutionCode: CNC; collectionCode: Insects; basisOfRecord: Pinned Specimen
- **Type status:** Paratype. **Occurrence:** occurrenceDetails: http://janzen.sas.upenn.edu; catalogNumber: DHJPAR0010398; recordedBy: D.H. Janzen, W. Hallwachs & Lucia Vargas; individualID: DHJPAR0010398; individualCount: 1; sex: M; lifeStage: adult; preparations: pinned; otherCatalogNumbers: ASTAS229-06, 06-SRNP-17593, BOLD:AAD4191; **Taxon:** scientificName: Telothyria aidani; phylum: Arthropoda; class: Insecta; order: Diptera; family: Tachinidae; genus: Telothyria; specificEpithet: aidani; scientificNameAuthorship: Fleming & Wood, 2018; **Location:** continent: Central America; country: Costa Rica; countryCode: CR; stateProvince: Guanacaste; county: Sector Horizontes; locality: Area de Conservacion Guanacaste; verbatimLocality: Vado Esperanza; verbatimElevation: 85; verbatimLatitude: 10.7894; verbatimLongitude: -85.551; verbatimCoordinateSystem: Decimal; decimalLatitude: 10.7894; decimalLongitude: -85.551; **Identification:** identifiedBy: AJ Fleming; dateIdentified: 2018; **Event:** samplingProtocol: Reared from the larva of the Crambidae, Spoladea recurvalis; verbatimEventDate: 20-Jul-2006; **Record Level:** language: en; institutionCode: CNC; collectionCode: Insects; basisOfRecord: Pinned Specimen
- **Type status:** Paratype. **Occurrence:** occurrenceDetails: http://janzen.sas.upenn.edu; catalogNumber: DHJPAR0010404; recordedBy: D.H. Janzen, W. Hallwachs & Lucia Vargas; individualID: DHJPAR0010404; individualCount: 1; sex: F; lifeStage: adult; preparations: pinned; otherCatalogNumbers: ASTAS235-06, 06-SRNP-17622, BOLD:AAD4191; **Taxon:** scientificName: Telothyria aidani; phylum: Arthropoda; class: Insecta; order: Diptera; family: Tachinidae; genus: Telothyria; specificEpithet: aidani; scientificNameAuthorship: Fleming & Wood, 2018; **Location:** continent: Central America; country: Costa Rica; countryCode: CR; stateProvince: Guanacaste; county: Sector Horizontes; locality: Area de Conservacion Guanacaste; verbatimLocality: Vado Esperanza; verbatimElevation: 85; verbatimLatitude: 10.7894; verbatimLongitude: -85.551; verbatimCoordinateSystem: Decimal; decimalLatitude: 10.7894; decimalLongitude: -85.551; **Identification:** identifiedBy: AJ Fleming; dateIdentified: 2018; **Event:** samplingProtocol: Reared from the larva of the Crambidae, Spoladea recurvalis; verbatimEventDate: 24-Jun-2006; **Record Level:** language: en; institutionCode: CNC; collectionCode: Insects; basisOfRecord: Pinned Specimen
- **Type status:** Paratype. **Occurrence:** occurrenceDetails: http://janzen.sas.upenn.edu; catalogNumber: DHJPAR0035637; recordedBy: D.H. Janzen, W. Hallwachs & Guillermo Pereira; individualID: DHJPAR0035637; individualCount: 1; sex: F; lifeStage: adult; preparations: pinned; otherCatalogNumbers: ASHYD1018-09, 09-SRNP-13714, BOLD:AAD4191; **Taxon:** scientificName: Telothyria aidani; phylum: Arthropoda; class: Insecta; order: Diptera; family: Tachinidae; genus: Telothyria; specificEpithet: aidani; scientificNameAuthorship: Fleming & Wood, 2018; **Location:** continent: Central America; country: Costa Rica; countryCode: CR; stateProvince: Guanacaste; county: Sector Horizontes; locality: Area de Conservacion Guanacaste; verbatimLocality: Vado Esperanza; verbatimElevation: 85; verbatimLatitude: 10.7894; verbatimLongitude: -85.551; verbatimCoordinateSystem: Decimal; decimalLatitude: 10.7894; decimalLongitude: -85.551; **Identification:** identifiedBy: AJ Fleming; dateIdentified: 2018; **Event:** samplingProtocol: Reared from the larva of the Crambidae, Spoladea recurvalis; verbatimEventDate: 26-Jun-2009; **Record Level:** language: en; institutionCode: CNC; collectionCode: Insects; basisOfRecord: Pinned Specimen
- **Type status:** Paratype. **Occurrence:** occurrenceDetails: http://janzen.sas.upenn.edu; catalogNumber: DHJPAR0044934; recordedBy: D.H. Janzen, W. Hallwachs & Mariano Pereira; individualID: DHJPAR0044934; individualCount: 1; sex: F; lifeStage: adult; preparations: pinned; otherCatalogNumbers: ACGAZ158-11, 11-SRNP-55546, BOLD:AAD4191; **Taxon:** scientificName: Telothyria aidani; phylum: Arthropoda; class: Insecta; order: Diptera; family: Tachinidae; genus: Telothyria; specificEpithet: aidani; scientificNameAuthorship: Fleming & Wood, 2018; **Location:** continent: Central America; country: Costa Rica; countryCode: CR; stateProvince: Guanacaste; county: Sector Mundo Nuevo; locality: Area de Conservacion Guanacaste; verbatimLocality: Vado Miramonte; verbatimElevation: 305; verbatimLatitude: 10.7718; verbatimLongitude: -85.434; verbatimCoordinateSystem: Decimal; decimalLatitude: 10.7718; decimalLongitude: -85.434; **Identification:** identifiedBy: AJ Fleming; dateIdentified: 2018; **Event:** samplingProtocol: Reared from the larva of the Crambidae, Herpetogramma Janzen04; verbatimEventDate: 13-Jun-2011; **Record Level:** language: en; institutionCode: CNC; collectionCode: Insects; basisOfRecord: Pinned Specimen
- **Type status:** Paratype. **Occurrence:** occurrenceDetails: http://janzen.sas.upenn.edu; catalogNumber: DHJPAR0044938; recordedBy: D.H. Janzen, W. Hallwachs & Mariano Pereira; individualID: DHJPAR0044938; individualCount: 1; sex: M; lifeStage: adult; preparations: pinned; otherCatalogNumbers: ACGAZ162-11, 11-SRNP-55548, BOLD:AAD4191; **Taxon:** scientificName: Telothyria aidani; phylum: Arthropoda; class: Insecta; order: Diptera; family: Tachinidae; genus: Telothyria; specificEpithet: aidani; scientificNameAuthorship: Fleming & Wood, 2018; **Location:** continent: Central America; country: Costa Rica; countryCode: CR; stateProvince: Guanacaste; county: Sector Mundo Nuevo; locality: Area de Conservacion Guanacaste; verbatimLocality: Vado Miramonte; verbatimElevation: 305; verbatimLatitude: 10.7718; verbatimLongitude: -85.434; verbatimCoordinateSystem: Decimal; decimalLatitude: 10.7718; decimalLongitude: -85.434; **Identification:** identifiedBy: AJ Fleming; dateIdentified: 2018; **Event:** samplingProtocol: Reared from the larva of the Crambidae, Herpetogramma Janzen04; verbatimEventDate: 14-Jun-2011; **Record Level:** language: en; institutionCode: CNC; collectionCode: Insects; basisOfRecord: Pinned Specimen

#### Description

**Male.** Length: 5–9 mm (Fig. 3). **Head** (Fig. 3b): frons narrow, 1/5 of head width; frontal vitta prominent and visible 1/12 head width; gena 1/5 of head height; four reclinate orbital setae; anteriormost reclinate orbital almost equal to uppermost frontal seta; ocellar setae absent; outer vertical seta absent; fronto-orbital plate gold throughout inclusive of ocellar triangle; fronto-orbital plate with short blonde hairs interspersed among frontal setae; parafacial pale silver; facial ridge bare; palpus short digitiform, sparsely haired along outer margin; arista brown, distinctly thickened on basal 1/10, microtrichia at most 3X as long as width of arista; postpedicel entirely orange; postocular region behind margin of eye upper half gold, with lower half including gena silver tomentose; upper half of occiput gold tomentose. It should be noted that in the case of the holotype of *Telothyria aidani* **sp. n.** the type specimen has dented eyes, a feature not normally present in the species. However, due to the condition of the remainder of the series this specimen was chosen as the best representative. **Thorax** (Fig. 3a, c): golden tomentose, with one pair of distinct outer dorsal stripes, inner pair of stripes reaching up to halfway between insertion of first and second postsutural dorsocentral setae; thorax entirely covered in dense plumose blonde hairs; chaetotaxy: 4–5 postpronotal setae, basal setae arranged in a straight line; supra-alar setae 2:3; intra-alar setae 1:3; dorsocentral setae 3:3; acrostichal setae 3:3; katepisternum with three setae. Scutellum golden tomentose; two pairs of strong marginal setae (basal and subapical) and a small pair of crossed apical scutellar setae 1/5th as long as subapical scutellars; basal scutellar setae subequal in length to subapical setae; subapical setae straight; underside of scutellum bearing plumose blonde hairs below basal scutellar setae. **Legs**: foreleg and midleg all yellow ground color, with only tarsal segments brown; hindleg yellow ground color on coxa and proximal half of femur and dark brown extending from distal half of femur to tarsal segments; anterior leg tibia with irregularly sized fringe of equally spaced setae along anteroventral surface, with two posterodorsal setae. **Wings**: basicosta ivory white; all veins bare, with only 1–2 setulae at base of R_4+5_.; calypters pale white translucent with a narrow yellowish fringe, upper calypter with a fringe of long setulae along margin. **Abdomen** (Fig. 3a, c): ground color yellow-orange; ST1+2 brown over medial 50%, with yellow ventrolaterally, extending into a longitudinal middorsal brown stripe terminating in a band along posterior edge of T4; T3–T5 with dense gold tomentum along anterior marginal 10%, thinning and extending over remainder of tergite; T5 orange ground color with gold tomentum, anterior edge of tergite with a slightly darker brown band and medial triangle; median marginal setae present only on T4 and T5; median discal setae absent. **Male terminalia** (Fig. 3d, e, f): Sternite 5 with a wide deeply separated median cleft, widely U-shaped, margins tomentose; lateral lobes of sternite rounded and blunt apically, outer margins covered in strong setae; basal section of sternite 5 2X as long as apical lobes. Cerci in posterior view sharply pointed, basally swollen along the basal 1/5, fused along entire length; medial undeveloped, entire structure narrow and needle-like spatha shaped. In lateral view cerci, with a strong beaklike downward curve, and several strong widely spaced setae along basal 1/5th. Surstylus in lateral view rounded and curved at tip, overall narrow digitiform in appearance; fused with epandrium; when viewed dorsally surstyli appear inwardly convergent with a very slight club or swelling apically. Distiphallus 3X as long as basiphallus and tubular, slightly pointed at apex.

**Female.** Length: 5–7 mm (Fig. 4). **Head** (Fig. 4b): as in male with the following exceptions: fronto-orbital plate 50% gold; parafacial brilliant silver; frons 1/4 of head width; three inner reclinate orbital setae; two proclinate orbital setae; outer vertical seta present; palpus short and clubbed. **Thorax** (Fig. 4a, c): katepisternum with three setae; meron with 6–7 typical meral setae. Legs: foreleg brilliant yellow ground color throughout; midleg and hindleg brilliant yellow ground color. **Abdomen** (Fig. 4a, c): ground color brown dorsally on ST1+2 and T3 with orange laterally; T4 entirely brown ground color; T5 as in male; marginal setae present on T4, T5 only.

#### Diagnosis

*Telothyria aidani* **sp. n.** can be distinguished from all other *Telothyria* by the following combination of traits: ocellar setae absent, parafacial pale silver, postpedicel entirely orange, arista plumose on lower half with microtrichia at most 3X as wide as arista, facial ridge bare, thorax entirely covered in dense plumose blonde hairs. Differs from *T. alexanderi* by ST1+2 brown over medial 50%, in females abdominal coloration overall darker than that of females of *T. alexanderi*, ground color brown dorsally on ST1+2 and T3 with orange laterally, and T4 entirely brown ground color. CO1 barcode differs from *Telothyria alexanderi* by 1%.

#### Etymology

*Telothyria aidani* **sp. n.** the new species is named in honor of my second son Aidan José Fleming. Just as we honor those who have worked before us, we must also recognize the potential of those who might continue our work and carry our legacy into the future.

#### Distribution

Costa Rica, ACG, Guanacaste Province, 85–305 m elevation.

#### Ecology

*Telothyria aidani* **sp. n.** has been reared 11 times from two species of Lepidoptera in the family Crambidae: *Herpetogramma Janzen04* and *Spoladea recurvalis* (Fabricius, 1775) in dry forest and dry-rain lowland intergrade.

### Telothyria alexanderi

Fleming & Wood sp. n.

66D61077-2BF0-539C-92EF-BB2A10D2128A

urn:lsid:zoobank.org:act:66F9D066-A701-4A01-A0D1-677AA0E61FAF

#### Materials

- **Type status:** Holotype. **Occurrence:** occurrenceDetails: http://janzen.sas.upenn.edu; catalogNumber: DHJPAR0010309; recordedBy: D.H. Janzen, W. Hallwachs & Lucia Vargas; individualID: DHJPAR0010309; individualCount: 1; sex: M; lifeStage: adult; preparations: pinned; otherCatalogNumbers: ASTAS140-06, 06-SRNP-17436, BOLD:AAD4191; **Taxon:** scientificName: Telothyria alexanderi; phylum: Arthropoda; class: Insecta; order: Diptera; family: Tachinidae; genus: Telothyria; specificEpithet: alexanderi; scientificNameAuthorship: Fleming & Wood, 2018; **Location:** continent: Central America; country: Costa Rica; countryCode: CR; stateProvince: Guanacaste; county: Sector Horizontes; locality: Area de Conservacion Guanacaste; verbatimLocality: Vado Esperanza; verbatimElevation: 85; verbatimLatitude: 10.7894; verbatimLongitude: -85.551; verbatimCoordinateSystem: Decimal; decimalLatitude: 10.7894; decimalLongitude: -85.551; **Identification:** identifiedBy: AJ Fleming; dateIdentified: 2018; **Event:** samplingProtocol: Reared from the larva of the Crambidae, Spoladea recurvalis; verbatimEventDate: 18-Jul-2006; **Record Level:** language: en; institutionCode: CNC; collectionCode: Insects; basisOfRecord: Pinned Specimen
- **Type status:** Paratype. **Occurrence:** occurrenceDetails: http://janzen.sas.upenn.edu; catalogNumber: DHJPAR0010391; recordedBy: D.H. Janzen, W. Hallwachs & Lucia Vargas; individualID: DHJPAR0010391; individualCount: 1; sex: F; lifeStage: adult; preparations: pinned; otherCatalogNumbers: ASTAS222-06, 06-SRNP-17529, BOLD:AAD4191; **Taxon:** scientificName: Telothyria alexanderi; phylum: Arthropoda; class: Insecta; order: Diptera; family: Tachinidae; genus: Telothyria; specificEpithet: alexanderi; scientificNameAuthorship: Fleming & Wood, 2018; **Location:** continent: Central America; country: Costa Rica; countryCode: CR; stateProvince: Guanacaste; county: Sector Horizontes; locality: Area de Conservacion Guanacaste; verbatimLocality: Vado Esperanza; verbatimElevation: 85; verbatimLatitude: 10.7894; verbatimLongitude: -85.551; verbatimCoordinateSystem: Decimal; decimalLatitude: 10.7894; decimalLongitude: -85.551; **Identification:** identifiedBy: AJ Fleming; dateIdentified: 2018; **Event:** samplingProtocol: Reared from the larva of the Crambidae, Spoladea recurvalis; verbatimEventDate: 19-Jul-2006; **Record Level:** language: en; institutionCode: CNC; collectionCode: Insects; basisOfRecord: Pinned Specimen
- **Type status:** Paratype. **Occurrence:** occurrenceDetails: http://janzen.sas.upenn.edu; catalogNumber: DHJPAR0010394; recordedBy: D.H. Janzen, W. Hallwachs & Lucia Vargas; individualID: DHJPAR0010394; individualCount: 1; sex: F; lifeStage: adult; preparations: pinned; otherCatalogNumbers: ASTAS225-06, 06-SRNP-17461, BOLD:AAD4191; **Taxon:** scientificName: Telothyria alexanderi; phylum: Arthropoda; class: Insecta; order: Diptera; family: Tachinidae; genus: Telothyria; specificEpithet: alexanderi; scientificNameAuthorship: Fleming & Wood, 2018; **Location:** continent: Central America; country: Costa Rica; countryCode: CR; stateProvince: Guanacaste; county: Sector Horizontes; locality: Area de Conservacion Guanacaste; verbatimLocality: Vado Esperanza; verbatimElevation: 85; verbatimLatitude: 10.7894; verbatimLongitude: -85.551; verbatimCoordinateSystem: Decimal; decimalLatitude: 10.7894; decimalLongitude: -85.551; **Identification:** identifiedBy: AJ Fleming; dateIdentified: 2018; **Event:** samplingProtocol: Reared from the larva of the Crambidae, Spoladea recurvalis; verbatimEventDate: 20-Jul-2006; **Record Level:** language: en; institutionCode: CNC; collectionCode: Insects; basisOfRecord: Pinned Specimen
- **Type status:** Paratype. **Occurrence:** occurrenceDetails: http://janzen.sas.upenn.edu; catalogNumber: DHJPAR0010397; recordedBy: D.H. Janzen, W. Hallwachs & Guillermo Pereira; individualID: DHJPAR0010397; individualCount: 1; sex: F; lifeStage: adult; preparations: pinned; otherCatalogNumbers: ASTAS228-06, 06-SRNP-17206, BOLD:AAD4191; **Taxon:** scientificName: Telothyria alexanderi; phylum: Arthropoda; class: Insecta; order: Diptera; family: Tachinidae; genus: Telothyria; specificEpithet: alexanderi; scientificNameAuthorship: Fleming & Wood, 2018; **Location:** continent: Central America; country: Costa Rica; countryCode: CR; stateProvince: Guanacaste; county: Sector Horizontes; locality: Area de Conservacion Guanacaste; verbatimLocality: Bejuco; verbatimElevation: 180; verbatimLatitude: 10.7671; verbatimLongitude: -85.5966; verbatimCoordinateSystem: Decimal; decimalLatitude: 10.7671; decimalLongitude: -85.5966; **Identification:** identifiedBy: AJ Fleming; dateIdentified: 2018; **Event:** samplingProtocol: Reared from the larva of the Crambidae, Spoladea recurvalis; verbatimEventDate: 20-Jul-2006; **Record Level:** language: en; institutionCode: CNC; collectionCode: Insects; basisOfRecord: Pinned Specimen
- **Type status:** Paratype. **Occurrence:** occurrenceDetails: http://janzen.sas.upenn.edu; catalogNumber: DHJPAR0044941; recordedBy: D.H. Janzen, W. Hallwachs & Mariano Pereira; individualID: DHJPAR0044941; individualCount: 1; sex: F; lifeStage: adult; preparations: pinned; otherCatalogNumbers: ACGAZ165-11, 11-SRNP-55540, BOLD:AAD4191; **Taxon:** scientificName: Telothyria alexanderi; phylum: Arthropoda; class: Insecta; order: Diptera; family: Tachinidae; genus: Telothyria; specificEpithet: alexanderi; scientificNameAuthorship: Fleming & Wood, 2018; **Location:** continent: Central America; country: Costa Rica; countryCode: CR; stateProvince: Guanacaste; county: Sector Mundo Nuevo; locality: Area de Conservacion Guanacaste; verbatimLocality: Vado Miramonte; verbatimElevation: 305; verbatimLatitude: 10.7718; verbatimLongitude: -85.434; verbatimCoordinateSystem: Decimal; decimalLatitude: 10.7718; decimalLongitude: -85.434; **Identification:** identifiedBy: AJ Fleming; dateIdentified: 2018; **Event:** samplingProtocol: Reared from the larva of the Crambidae, Herpetogramma Janzen04; verbatimEventDate: 11-Jun-2011; **Record Level:** language: en; institutionCode: CNC; collectionCode: Insects; basisOfRecord: Pinned Specimen

#### Description

**Male.** Length: 6 mm (Fig. 5). **Head** (Fig. 5b): frons narrow, 1/5 of head width; frontal vitta prominent and visible 1/12 head width; gena 1/5 of head height; 3–4 reclinate orbital setae; anteriormost reclinate orbital almost equal to uppermost frontal seta; ocellar setae absent; outer vertical seta absent; fronto-orbital plate gold throughout; fronto-orbital plate with short blonde hairs interspersed among frontal setae; parafacial pale silver; facial ridge bare; palpus short digitiform, sparsely haired along outer margin; arista plumose, brown, distinctly thickened on basal 1/10, microtrichia at most 3X as long as width of arista; postpedicel entirely orange; postocular region behind margin of eye upper half gold, with lower half including gena silver tomentose; upper half of occiput gold tomentose. **Thorax** (Fig. 5a, c): pale brassy-golden tomentose, with two distinct outer dorsal stripes, inner stripes reaching only slightly beyond suture, up to but not beyond insertion of first postsutural dorsocentral seta, these almost invisible through dense blonde hairs; thorax entirely covered in dense plumose blonde hairs; chaetotaxy: 4–5 postpronotal setae, basal setae arranged in a straight line; supra-alar setae 2:3; intra-alar setae 1:3; dorsocentral setae 3:3; acrostichal setae 3:3; katepisternum with three setae. Scutellum golden tomentose; two pairs of strong marginal setae (basal and subapical) and a small pair of crossed apical scutellar setae 1/5th as long as subapical scutellars; basal scutellar setae subequal in length to subapical setae; subapical setae straight; underside of scutellum bearing plumose blonde hairs below basal scutellar setae. **Legs**: foreleg and midleg all yellow ground color, with only tarsal segments brown; hindleg yellow ground color on coxa and proximal 2/3 of femur and dark brown extending from distal 1/3 of femur, with yellow tibia, and brown tarsal segments; anterior leg tibia with irregularly sized fringe of equally spaced setae along anteroventral surface, with two posterodorsal setae. **Wings**: basicosta ivory white; all veins bare, with only 1–2 setulae at base of R_4+5_; calypters pale white translucent with a narrow yellowish fringe, upper calypter with a fringe of long setulae along margin. **Abdomen** (Fig. 5a, c): ground color yellow-orange; ST1+2 brown over medial 30%, with yellow ventrolaterally, extending into a longitudinal middorsal brown stripe terminating in a band along posterior edge of T4; T3–T5 with dense brassy-silver tomentum along anterior marginal 10%, thinning and extending over remainder of tergite; T5 orange ground color with gold tomentum, anterior edge of tergite with a slightly darker brown band and medial triangle; median marginal setae present only on T4 and T5; median discal setae absent. **Male terminalia**: not examined.

**Female.** Length: 6–8 mm (Fig. 6). **Head** (Fig. 6b): as in male with the following exceptions: fronto-orbital plate up to 50% gold; parafacial brilliant silver; frons 1/4 of head width; three inner reclinate orbital setae; two proclinate orbital setae; outer vertical seta present; palpus short and clubbed. **Thorax** (Fig. 6a, c): pale brassy-golden tomentose beneath golden hairs; meron with 5–7 typical meral setae. Legs: colored as in male. **Abdomen** (Fig. 6a, c): ground color orange entirely with a dorsocentral brown stripe along T3 and T4 and anterior 1/2 of T5; T5 as in male; marginal setae present on T4, T5 only.

#### Diagnosis

*Telothyria alexanderi* **sp. n.** can be distinguished from all other *Telothyria* by the following combination of traits: ocellar setae absent, parafacial pale silver, postpedicel entirely orange, arista plumose on lower half with microtrichia at most 3X as long as width of arista, facial ridge bare, thorax entirely covered in dense plumose blonde hairs. Differs from *T. aidani* by ST1+2 brown over medial 30%, females generally lighter than those of *T. aidani*, with ground color orange entirely and a dorsocentral brown stripe along T3 and T4 and anterior 1/2 of T5. CO1 barcode differs from *Telothyria aidani* **sp. n.** by 1%.

#### Etymology

*Telothyria alexanderi* **sp. n.** the new species is named in honor of my first son Alexander José Fleming, who inspires me everyday to continue to learn and strive to make this world a better place for the future.

#### Distribution

Costa Rica, ACG, Guanacaste Province, 85–305 m elevation.

#### Ecology

*Telothyria alexanderi* **sp. n.** has been reared five times from two species of Lepidoptera in the family Crambidae: *Herpetogramma* Janzen04 and *Spoladea recurvalis* in dry forest and dry-rain lowland intergrade.

### Telothyria auranticrus

Fleming & Wood sp. n.

231F1D46-427E-5B26-ADA3-E7DB431234FC

urn:lsid:zoobank.org:act:FBB2B540-13E0-403A-B9C4-3277D56D1853

#### Materials

- **Type status:** Holotype. **Occurrence:** catalogNumber: CNC618908; recordedBy: B.V. Peterson; individualID: CNC618908; individualCount: 1; sex: M; lifeStage: adult; preparations: pinned; **Taxon:** scientificName: Telothyria auranticrus; phylum: Arthropoda; class: Insecta; order: Diptera; family: Tachinidae; genus: Telothyria; specificEpithet: auranticrus; scientificNameAuthorship: Fleming & Wood, 2019; **Location:** continent: Central America; country: Mexico; countryCode: MX; stateProvince: Veracruz; verbatimLocality: Lake Catemaco; **Identification:** identifiedBy: AJ Fleming; dateIdentified: 2019; **Event:** samplingProtocol: Hand collected; verbatimEventDate: 17-Jun-1969; **Record Level:** language: en; institutionCode: CNC; collectionCode: Insects; basisOfRecord: Pinned Specimen
- **Type status:** Paratype. **Occurrence:** recordedBy: Jas. S. Hine; sex: M; lifeStage: adult; preparations: pinned; **Taxon:** scientificName: Telothyria auranticrus; phylum: Arthropoda; class: Insecta; order: Diptera; family: Tachinidae; genus: Telothyria; specificEpithet: auranticrus; scientificNameAuthorship: Fleming & Wood, 2019; **Location:** continent: Central America; country: Guatemala; verbatimLocality: Los Amates; **Identification:** identifiedBy: AJ Fleming; dateIdentified: 2018; **Event:** samplingProtocol: Hand collected; verbatimEventDate: 16-Jan-2005; **Record Level:** language: en; institutionCode: CNC; collectionCode: Insects; basisOfRecord: Pinned Specimen
- **Type status:** Paratype. **Occurrence:** recordedBy: J.G. Chillcott; sex: M; lifeStage: adult; preparations: pinned; **Taxon:** scientificName: Telothyria auranticrus; phylum: Arthropoda; class: Insecta; order: Diptera; family: Tachinidae; genus: Telothyria; specificEpithet: auranticrus; scientificNameAuthorship: Fleming & Wood, 2019; **Location:** continent: Central America; country: Mexico; countryCode: MX; stateProvince: San Luis Potosí; verbatimLocality: 5 mi E of Xilitla; verbatimElevation: 487; **Identification:** identifiedBy: AJ Fleming; dateIdentified: 2018; **Event:** samplingProtocol: Hand collected; verbatimEventDate: 23-Jul-1954; **Record Level:** language: en; institutionCode: CNC; collectionCode: Insects; basisOfRecord: Pinned Specimen

#### Description

**Male.** Length: 9–11mm (Fig. 7). **Head** (Fig. 7b): frons narrow, 1/7 of head width; frontal vitta narrow, but prominent and visible 1/48 head width; gena 1/10 of head height; three reclinate orbital setae; anteriormost reclinate orbital almost equal to uppermost frontal seta; ocellar setae reduced but present, at most hair-like; outer vertical seta absent; fronto-orbital plate pale brassy-gold throughout; fronto-orbital plate with short pale reddish blonde hairs interspersed among frontal setae; parafacial pale silver; facial ridge bare with at most 6–8 supravibrissal hairs extending 1/5 the facial ridge; palpus elongate and digitiform with slight upward turn apically, sparsely haired along outer margin; arista brown, smoothly tapered, microtrichia at most equal to width of arista; postpedicel entirely orange; postocular region behind margin of eye upper half gold, with lower half including gena silver tomentose; upper half of occiput gold tomentose. **Thorax** (Fig. 7a, c): brassy-gold tomentose, with four distinct dorsal stripes, outer pair light and diffuse but evident, inner pair extending midway between 1st and 2nd dorsocentral setae; thorax entirely covered in dense plumose blonde hairs (sometimes sparse on disc of scutum); chaetotaxy: 4–5 postpronotal setae, basal setae arranged in a straight line; supra-alar setae 2:3; intra-alar setae 2:2; dorsocentral setae 3:3; acrostichal setae 3:3; katepisternum with two setae. Scutellum gold tomentose; with plumose hairs along anterior margins only, disc of scutellum with only short black setulae; two pairs of strong marginal setae (basal and subapical) and a small pair of crossed apical scutellar setae 1/5th as long as subapical scutellars; basal scutellar setae subequal in length to subapical setae; subapical setae straight; underside of scutellum bearing plumose blonde hairs below basal scutellar setae. **Legs**: all legs with yellow ground color, midleg and hindleg appearing darker due to dense covering of black hairs; anterior leg tibia with regular fringe of equally spaced setae along anteroventral surface, with two posterodorsal setae. **Wings**: basicosta orange; all veins bare, with only 1–2 setulae at base of R_4+5_; calypters pale white translucent, upper calyter with fringe of long white hairs. **Abdomen** (Fig. 7a, c): ground color dark brown dorsocentrally, with yellow lateroventrally apparent when viewed dorsally; ST1+2 dorsomedially dark brown over 3/5 with yellow laterally; T4 entirely dark brown ground color, and T5 entirely orange; T3–T5 with dense gold tomentum along anterior margin of tergite, diffusing over entire tergite appearing to have a golden sheen when viewed with the naked eye; T5 orange ground color with gold tomentum; a complete row of marginal setae on T4 and T5; median discal setae absent. **Male terminalia**: Sternite 5 with a wide deeply separated median cleft, widely U-shaped, margins tomentose; lateral lobes of sternite elongate, rounded and blunt apically, bearing many strong setae; basal section of sternite 5 .75X as long as apical lobes. Cerci in posterior view sharply pointed, basally swollen along the basal 2/3, fused along entire length; medial shoulder well developed, anterior 1/3 narrow and needle-like sharply tapered. In lateral view cerci, with a strong beaklike downward curve, and several strong widely spaced setae along basal 1/5th. Surstylus in lateral view rounded and curved at tip, overall narrow digitiform in appearance; fused with epandrium; when viewed dorsally surstyli appear inwardly convergent with a very slight club or swelling apically. Distiphallus 4X as long as basiphallus and tubular, slightly pointed at apex.

**Female.** Unknown at this time.

#### Diagnosis

*Telothyria auranticrus* **sp. n.** can be distinguished from all other *Telothyria* by the following combination of traits: frons narrow, fronto-orbital plate pale brassy-gold throughout, parafacial pale silver, two katepisternal setae, thorax entirely covered in dense plumose blonde hairs, sometimes sparse on disc of scutum, T4 entirely dark brown, and T5 orange with gold tomentum.

#### Etymology

*Telothyria auranticrus* **sp. n.** From the Latin adjective, “*aurantium*” for orange and the noun "*crus*" for leg, in reference to its bright orange legs.

#### Distribution

Mexico, Veracruz Province, Lake Catemaco; San Luis Potosi Province, Xilitila.

#### Ecology

Specimens hand collected two times, further ecology not available.

### Telothyria auriolus

Fleming & Wood sp. n.

222ED289-819D-5D8B-8D83-74C511D67C42

urn:lsid:zoobank.org:act:2B76B229-8DFE-4A0C-8E1D-DBC15CFF0847

#### Materials

- **Type status:** Holotype. **Occurrence:** occurrenceDetails: http://janzen.sas.upenn.edu; catalogNumber: CNC618910; recordedBy: B.V. Peterson; individualID: CNC618910; individualCount: 1; sex: M; lifeStage: adult; preparations: pinned; **Taxon:** scientificName: Telothyria auriolus; phylum: Arthropoda; class: Insecta; order: Diptera; family: Tachinidae; genus: Telothyria; specificEpithet: auriolus; scientificNameAuthorship: Fleming & Wood, 2019; **Location:** continent: Central America; country: Mexico; countryCode: MX; stateProvince: Chiapas; verbatimLocality: Palenque ruins; **Identification:** identifiedBy: AJ Fleming; dateIdentified: 2019; **Event:** samplingProtocol: Hand collected; verbatimEventDate: 22-Jun-1969; **Record Level:** language: en; institutionCode: CNC; collectionCode: Insects; basisOfRecord: Pinned Specimen

#### Description

**Male.** Length: 10 mm (Fig. 8). **Head** (Fig. 8b): frons narrow, 1/6 of head width; frontal vitta narrow yet prominent and visible 1/24 head width; gena 1/11 of head height; 2–3 reclinate orbital setae; anteriormost reclinate orbital almost equal to uppermost frontal seta; ocellar setae absent; outer vertical seta absent; fronto-orbital plate silver throughout; fronto-orbital plate with short blonde hairs interspersed among frontal setae; parafacial pale silver; facial ridge bare; palpus short digitiform with slight upward turn apically, sparsely haired along outer margin; arista brown, apically orange, smoothly tapered, microtrichia at most as long as width of arista; postpedicel entirely orange; postocular region behind margin of eye including gena silver tomentose; occiput gold tomentose over upper 3/4. **Thorax** (Fig. 8a, c): gold tomentose, with two almost indistinct pairs of dorsal stripes, outer pair short extending up to 2nd postsutural dorsocentral, broken widely across suture, inner stripes short and slightly broken across suture, only extending up to 1st postsutural dorsocentral; thorax entirely covered in dense plumose blonde hairs; chaetotaxy: 4–5 postpronotal setae, basal setae arranged in a straight line; supra-alar setae 2:3; intra-alar setae 1:2; dorsocentral setae 3:4; acrostichal setae 3:3; katepisternum with three setae. Scutellum gold tomentose; two pairs of strong marginal setae (basal and subapical) and a small pair of crossed apical scutellar setae 1/5th as long as subapical scutellars; basal scutellar setae subequal in length to subapical setae; subapical setae straight; underside of scutellum bearing plumose blonde hairs below basal scutellar setae. **Legs**: foreleg yellow entirely, midleg dark along tibia and tarsal segments, hindleg with coxa and proximal half of femur yellow, and remainder dark brown in color; anterior leg tibia with regular fringe of equally spaced setae along anteroventral surface, with two posterodorsal setae. **Wings**: basicosta ivory white; all veins bare, with only 1–2 setulae at base of R_4+5_; calypteres white translucent with a narrow yellowish fringe. **Abdomen** (Fig. 8a, c): ground color dark brown dorsocentrally, with yellow lateroventrally apparent when viewed dorsally; ST1+2 dorsomedially dark brown over 60%, with yellow laterally; T4 with anterior half of tergite yellow ground color, and caudal 1/2 dark brown ground color; T3–T5 with dense silver tomentum along anterior margin of tergite, diffusing over entire tergite, appearing to have a silver sheen when viewed with the naked eye; T5 orange ground color with silver tomentum; median marginal setae absent from T3 and a complete row of marginal setae on T4 and T5; median discal setae absent. **Male terminalia**: not examined.

**Female.** unknown at this time.

#### Diagnosis

*Telothyria auriolus* **sp. n.** can be distinguished from all other *Telothyria* by the following combination of traits: frons narrow, fronto-orbital plate silver throughout, parafacial pale silver, three katepisternal setae, two postsutural intra-alar setae, basicosta ivory white, thorax entirely covered in dense plumose blonde hairs, median marginal setae absent from T3.

#### Etymology

*Telothyria auriolus* **sp. n.** From the Latin adjective, “*auriolus*” meaning made of gold, in reference to its overall light color and its brilliant yellow legs.

#### Distribution

Mexico, Chiapas Province, Palenque Ruins; Honduras, Atlantida, 950 m elevation.

#### Ecology

Specimens hand collected five times at high elevations, further ecology not available.

### Telothyria bicuspidata

Fleming & Wood sp. n.

1F121281-CC94-52E9-AE4B-8378A29AD066

urn:lsid:zoobank.org:act:BE5AB41C-143F-4AF3-B1A4-ABE0A5AE543F

#### Materials

- **Type status:** Holotype. **Occurrence:** catalogNumber: CNC618907; recordedBy: D.M. Wood; individualID: CNC618907; individualCount: 1; sex: M; lifeStage: adult; preparations: pinned; **Taxon:** scientificName: Telothyria bicuspidata; phylum: Arthropoda; class: Insecta; order: Diptera; family: Tachinidae; genus: Telothyria; specificEpithet: bicuspidata; scientificNameAuthorship: Fleming & Wood, 2019; **Location:** continent: Central America; country: Costa Rica; countryCode: CR; stateProvince: Puntarenas; verbatimLocality: Monteverde; verbatimElevation: 1500; **Identification:** identifiedBy: AJ Fleming; dateIdentified: 2019; **Event:** samplingProtocol: Hand collected; verbatimEventDate: 20-22-Jul-1993; **Record Level:** language: en; institutionCode: CNC; collectionCode: Insects; basisOfRecord: Pinned Specimen
- **Type status:** Paratype. **Occurrence:** catalogNumber: CNC618893; recordedBy: D.M. Wood; individualID: CNC618893; individualCount: 1; sex: M; lifeStage: adult; preparations: pinned; **Taxon:** scientificName: Telothyria bicuspidata; phylum: Arthropoda; class: Insecta; order: Diptera; family: Tachinidae; genus: Telothyria; specificEpithet: bicuspidata; scientificNameAuthorship: Fleming & Wood, 2019; **Location:** continent: Central America; country: Costa Rica; countryCode: CR; stateProvince: Puntarenas; verbatimLocality: Monteverde; verbatimElevation: 1500; **Identification:** identifiedBy: AJ Fleming; dateIdentified: 2019; **Event:** samplingProtocol: Hand collected; verbatimEventDate: 20-22-Jul-1993; **Record Level:** language: en; institutionCode: CNC; collectionCode: Insects; basisOfRecord: Pinned Specimen
- **Type status:** Paratype. **Occurrence:** recordedBy: D.M. Wood; individualCount: 1; sex: M; lifeStage: adult; preparations: pinned; **Taxon:** scientificName: Telothyria bicuspidata; phylum: Arthropoda; class: Insecta; order: Diptera; family: Tachinidae; genus: Telothyria; specificEpithet: bicuspidata; scientificNameAuthorship: Fleming & Wood, 2019; **Location:** continent: Central America; country: Costa Rica; countryCode: CR; stateProvince: Puntarenas; verbatimLocality: Monteverde; verbatimElevation: 1500; **Identification:** identifiedBy: AJ Fleming; dateIdentified: 2019; **Event:** samplingProtocol: Hand collected; verbatimEventDate: 20-22-Jul-1993; **Record Level:** language: en; institutionCode: CNC; collectionCode: Insects; basisOfRecord: Pinned Specimen
- **Type status:** Paratype. **Occurrence:** recordedBy: D.M. Wood; individualCount: 1; sex: M; lifeStage: adult; preparations: pinned; **Taxon:** scientificName: Telothyria bicuspidata; phylum: Arthropoda; class: Insecta; order: Diptera; family: Tachinidae; genus: Telothyria; specificEpithet: bicuspidata; scientificNameAuthorship: Fleming & Wood, 2019; **Location:** continent: Central America; country: Costa Rica; countryCode: CR; stateProvince: Puntarenas; verbatimLocality: Monteverde; verbatimElevation: 1500; **Identification:** identifiedBy: AJ Fleming; dateIdentified: 2019; **Event:** samplingProtocol: Hand collected; verbatimEventDate: 20-22-Jul-1993; **Record Level:** language: en; institutionCode: CNC; collectionCode: Insects; basisOfRecord: Pinned Specimen
- **Type status:** Paratype. **Occurrence:** recordedBy: D.M. Wood; individualCount: 1; sex: M; lifeStage: adult; preparations: pinned; **Taxon:** scientificName: Telothyria bicuspidata; phylum: Arthropoda; class: Insecta; order: Diptera; family: Tachinidae; genus: Telothyria; specificEpithet: bicuspidata; scientificNameAuthorship: Fleming & Wood, 2019; **Location:** continent: Central America; country: Costa Rica; countryCode: CR; stateProvince: Puntarenas; verbatimLocality: Monteverde; verbatimElevation: 1500; **Identification:** identifiedBy: AJ Fleming; dateIdentified: 2019; **Event:** samplingProtocol: Hand collected; verbatimEventDate: 20-22-Jul-1993; **Record Level:** language: en; institutionCode: CNC; collectionCode: Insects; basisOfRecord: Pinned Specimen
- **Type status:** Paratype. **Occurrence:** recordedBy: D.M. Wood; individualCount: 1; sex: M; lifeStage: adult; preparations: pinned; **Taxon:** scientificName: Telothyria bicuspidata; phylum: Arthropoda; class: Insecta; order: Diptera; family: Tachinidae; genus: Telothyria; specificEpithet: bicuspidata; scientificNameAuthorship: Fleming & Wood, 2019; **Location:** continent: Central America; country: Costa Rica; countryCode: CR; stateProvince: Puntarenas; verbatimLocality: Monteverde; verbatimElevation: 1500; **Identification:** identifiedBy: AJ Fleming; dateIdentified: 2019; **Event:** samplingProtocol: Hand collected; verbatimEventDate: 20-22-Jul-1993; **Record Level:** language: en; institutionCode: CNC; collectionCode: Insects; basisOfRecord: Pinned Specimen

#### Description

**Male.** Length: 8–10 mm (Fig. 9). **Head** (Fig. 9b): frons narrow, 1/6 of head width; frontal vitta narrow yet prominent and visible 1/24 head width; gena 1/12 of head height; four reclinate orbital setae; anteriormost reclinate orbital subequal to uppermost frontal seta; ocellar setae reduced, almost absent; outer vertical seta absent; fronto-orbital plate pale brassy-gold throughout; fronto-orbital plate with short blonde hairs interspersed among frontal setae; parafacial pale silver; facial ridge bare; palpus elongate and slender digitiform with slight upward turn apically, sparsely haired along outer margin slightly darkened at tips; arista brown, smoothly tapered, microtrichia short at most as long as width of arista; postpedicel only 1/10 orange adjacent to pedicel; postocular region behind margin of eye upper 3/4 gold, with lower portion including gena silver tomentose; upper 3/4 of occiput gold tomentose. **Thorax** (Fig. 9a, c): dark ground color with brassy gold tomentum tomentose, with two pairs of distinct dorsal stripes, outer pair thick and prominent, inner pair extending up to second postsutural dorsocentral seta, when viewed from behind a fifth stripe appears dorsocentrally between postsutural acrostichal setae; thorax covered in dense plumose blonde hairs throughout; chaetotaxy: 4–5 postpronotal setae, basal setae arranged in a straight line; supra-alar setae 2:3; intra-alar setae 2:3; dorsocentral setae 3:3; acrostichal setae 4:3; katepisternum with two setae. Scutellum dark brown ground color, slightly gold tomentose; two pairs of strong marginal setae (basal and subapical) and a small pair of crossed apical scutellar setae 1/5th as long as subapical scutellars; basal scutellar setae subequal in length to subapical setae; subapical setae straight; underside of scutellum bearing plumose blonde hairs below basal scutellar setae. **Legs**: foreleg with yellow ground color covered in dark hairs giving tibia and tarsal segments an overall dark appearance; midleg and hind leg dark brown ground color, both with yellow coxa; anterior leg tibia with regular fringe of equally spaced setae along anteroventral surface, and 2–3 posterodorsal setae. **Wings**: basicosta beige/orange brown basally; wings brown slightly infuscate, all veins bare, with only 1–2 setulae at base of R_4+5_; calypters brassy brown translucent, with a narrow yellowish fringe. **Abdomen** (Fig. 9a, c): ground color dark burnt orange to with brown medially apparent when viewed dorsally; ST1+2 dark over dorsomedial 50%, with yellow laterally and dark stripe laterally which continues along T3 and T4; T3–T5 with dense gold tomentum along anterior margin of tergite, diffusing over entire tergite appearing to have a golden sheen when viewed with the naked eye; T5 orange ground color with gold tomentum; median marginal setae on ST1+2–T3 and a complete row on T4 and T5; median discal setae absent. **Male terminalia** (Fig. 9d, e, f): Sternite 5 with a narrow deeply cloven median cleft, narrowly V-shaped, margins tomentose; lateral lobes of sternite elongate and subtriangular apically, outer margins covered in strong setae, overall appearance like rabbit ears; basal section of sternite 5 almost 1/2 as long as length of apical lobes. Cerci in posterior view sharply pointed with a strong rectangular shoulder, slightly longer than surstyli, fused along entire length; medial shoulder rounded and smoothly tapered, not abrupt as in other species. In lateral view cerci, with a strong downward bend, along apical 1/2, and several strong widely spaced setae along basal 1/3rd. Surstylus in lateral view narrow and digitiform rounded at tip; fused with epandrium; when viewed dorsally surstyli appearing straight and convergent. Basiphallus long and narrow, distiphallus 3X as long as basiphallus, weakly tapering apically.

**Female.** Unknown at this time.

#### Diagnosis

*Telothyria bicuspidata* **sp. n.** can be distinguished from its congeners by the following combination of traits: frons narrow, fronto-orbital plate pale brassy-gold throughout, thorax covered in dense plumose blonde hairs throughout, two katepisternal setae, three postsutral intra-alar setae, wings slightly infuscate, calypters brassy brown, and median marginal setae present on ST1+2 and T3.

#### Etymology

*Telothyria bicuspidata* **sp. n.** From the Latin prefix "*bi*-" meaning two, the noun, "*cuspis*" meaning tooth, and the suffix "*ata*" in reference to T5 resembling a pair of canine (cuspid) teeth.

#### Distribution

Costa Rica, Puntarenas Province, Monteverde 1500 m elevation.

#### Ecology

Specimens hand collected, six times from 1500 m, further ecology not available.

### Telothyria carolinacanoae

Fleming & Wood sp. n.

A6CD7408-FDDB-5F4E-965B-942169D8172B

urn:lsid:zoobank.org:act:BE0DA15B-1378-41C5-9DC5-C8A4FDF924EB

#### Materials

- **Type status:** Holotype. **Occurrence:** occurrenceDetails: http://janzen.sas.upenn.edu; catalogNumber: DHJPAR0050516; recordedBy: D.H. Janzen, W. Hallwachs & Roster Moraga; individualID: DHJPAR0050516; individualCount: 1; sex: F; lifeStage: adult; preparations: pinned; otherCatalogNumbers: ACGBA3108-13, 12-SRNP-21998, BOLD:ABU7495; **Taxon:** scientificName: Telothyria carolinacanoae; phylum: Arthropoda; class: Insecta; order: Diptera; family: Tachinidae; genus: Telothyria; specificEpithet: carolinacanoae; scientificNameAuthorship: Fleming & Wood, 2018; **Location:** continent: Central America; country: Costa Rica; countryCode: CR; stateProvince: Guanacaste; county: Sector Del Oro; locality: Area de Conservacion Guanacaste; verbatimLocality: Suampo Guapinoles; verbatimElevation: 292; verbatimLatitude: 11.04725; verbatimLongitude: -85.474; verbatimCoordinateSystem: Decimal; decimalLatitude: 11.04725; decimalLongitude: -85.474; **Identification:** identifiedBy: AJ Fleming; dateIdentified: 2018; **Event:** samplingProtocol: Reared from the larva of the Crambidae, Herpetogramma phaeopteralis; verbatimEventDate: 25-Nov-2012; **Record Level:** language: en; institutionCode: CNC; collectionCode: Insects; basisOfRecord: Pinned Specimen

#### Description

**Female.** Length: 6 mm (Fig. 10). **Head** (Fig. 10b): frons narrow, 1/3 of head width; frontal vitta narrow yet prominent and visible 1/12 head width; gena 1/10 of head height; three reclinate inner orbital setae uppermost reclinate orbital pair slightly convergent, and two proclinate orbital setae; ocellar setae absent; outer vertical seta present; fronto-orbital plate pale brassy-gold along upper third inclusive of ocellar triangle; fronto-orbital plate with short blonde hairs interspersed among frontal setae; parafacial brilliant silver; facial ridge bare; palpus apically clubbed and slightly upturned; arista brown, smoothly tapering to apical 1/8, microtrichia at most 1.5X as long as width of arista; postpedicel orange over at most 30% of surface, proximal to pedicel; postocular region behind margin of eye upper 1/3 gold, with lower 2/3 including gena silver tomentose; upper 1/3 of occiput gold tomentose. **Thorax** (Fig. 10a, c): light brown ground color covered with pale gold tomentum, with four distinct thoracic stripes outer pair broken across suture; plumose blonde hairs absent from disc of scutum; chaetotaxy: 5–6 postpronotal setae, basal setae arranged in a straight line; supra-alar setae 2:3; intra-alar setae 2:2; dorsocentral setae 3:3; acrostichal setae 3:3; katepisternum with three setae; meron lacking plumose hairs with 4–5 typical meral setae. Scutellum brassy-gold tomentose; underside of scutellum bearing plumose blonde hairs; two pairs of strong marginal setae (basal and subapical) and a small pair of crossed apical scutellar setae 1/8–1/10th as long as subapical scutellars; basal scutellar setae subequal in length to subapical setae; subapical setae straight; underside of scutellum bearing plumose blonde hairs below basal scutellar setae. **Legs**: foreleg ground color yellow over coxa, femur, and tibia, tarsal segments appearing darker due to covering of dark hairs; midleg and hindleg with yellow coxa, and remainder dark brown entirely; anterior leg tibia with irregularly sized, tapered fringe of equally spaced setae along basal half of anteroventral surface, one posterodorsal seta. **Wings**: basicosta ivory white; all veins bare, with only one setula at base of R_4+5_; calypters pale white translucent. **Abdomen** (Fig. 10a, c): ground color mostly brown with yellow-orange present ventrolaterally; ST1+2–T4 with gold tomentum at tergal margin changing to silver tomentum extending over up to 50% of tergite; T5 brown along anterior margin with yellow apically, tergite covered with silver tomentum along anterior 50%; marginal setae present on T4 and T5; median discal setae absent.

**Male.** Unknown at this time.

#### Diagnosis

*Telothyria carolinacanoae* **sp. n.** can be distinguished from all other *Telothyria* by the following combination of traits: ocellar setae absent, plumose blonde hairs absent from disc of scutum, katepisternum with three setae, two postsutural intra-alar setae, and T1+2–T4 with gold tomentum at tergal margin changing to silver tomentum extending over up to 50% of tergite, ground color mostly brown with yellow-orange present ventrolaterally.

#### Etymology

*Telothyria carolinacanoae* **sp. n.** is named in recognition of Carolina Cano's outstanding work on the team that conducts the caterpillar and parasite inventory from ACG’s Estación Biológica San Gerardo.

#### Distribution

Costa Rica, ACG, Guanacaste Province, 292m elevation.

#### Ecology

*Telothyria carolinacanoae* **sp. n.** has been reared once from a single species of Lepidoptera in the family Crambidae: *Herpetogramma phaeopteralis*, in rain forest.

### Telothyria clavata

Fleming & Wood sp. n.

7C95639D-EF50-5987-86D3-F65EE0761F9A

urn:lsid:zoobank.org:act:08E53E79-B368-4E7F-A02B-137545B134E4

#### Materials

- **Type status:** Holotype. **Occurrence:** catalogNumber: CNC618909; recordedBy: M. Pollack, and D.M. Wood; individualID: CNC618909; individualCount: 1; sex: M; lifeStage: adult; preparations: pinned; **Taxon:** scientificName: Telothyria clavata; phylum: Arthropoda; class: Insecta; order: Diptera; family: Tachinidae; genus: Telothyria; specificEpithet: clavata; scientificNameAuthorship: Fleming & Wood, 2019; **Location:** continent: Central America; country: Costa Rica; countryCode: CR; stateProvince: Puntarenas; verbatimLocality: Monteverde; verbatimElevation: 1400; **Identification:** identifiedBy: AJ Fleming; dateIdentified: 2019; **Event:** samplingProtocol: Hand collected; verbatimEventDate: 17-20-Sep-1989; **Record Level:** language: en; institutionCode: CNC; collectionCode: Insects; basisOfRecord: Pinned Specimen
- **Type status:** Paratype. **Occurrence:** catalogNumber: CNC618894; recordedBy: D.M. Wood; individualID: CNC618894; sex: M; lifeStage: adult; preparations: pinned; **Taxon:** scientificName: Telothyria clavata; phylum: Arthropoda; class: Insecta; order: Diptera; family: Tachinidae; genus: Telothyria; specificEpithet: clavata; scientificNameAuthorship: Fleming & Wood, 2019; **Location:** continent: Central America; country: Costa Rica; countryCode: CR; stateProvince: Puntarenas; verbatimLocality: Monteverde; verbatimElevation: 1500; **Identification:** identifiedBy: AJ Fleming; dateIdentified: 2019; **Event:** samplingProtocol: Hand collected; verbatimEventDate: 28-Aug-1993; **Record Level:** language: en; institutionCode: CNC; collectionCode: Insects; basisOfRecord: Pinned Specimen
- **Type status:** Paratype. **Occurrence:** recordedBy: M. Pollack, and D.M. Wood; sex: M; lifeStage: adult; preparations: pinned; **Taxon:** scientificName: Telothyria clavata; phylum: Arthropoda; class: Insecta; order: Diptera; family: Tachinidae; genus: Telothyria; specificEpithet: clavata; scientificNameAuthorship: Fleming & Wood, 2019; **Location:** continent: Central America; country: Costa Rica; countryCode: CR; stateProvince: Puntarenas; verbatimLocality: Monteverde; verbatimElevation: 1400; **Identification:** identifiedBy: AJ Fleming; dateIdentified: 2019; **Event:** samplingProtocol: Hand collected; verbatimEventDate: 17-20-Sep-1989; **Record Level:** language: en; institutionCode: CNC; collectionCode: Insects; basisOfRecord: Pinned Specimen
- **Type status:** Paratype. **Occurrence:** recordedBy: D.M. Wood; sex: M; lifeStage: adult; preparations: pinned; **Taxon:** scientificName: Telothyria clavata; phylum: Arthropoda; class: Insecta; order: Diptera; family: Tachinidae; genus: Telothyria; specificEpithet: clavata; scientificNameAuthorship: Fleming & Wood, 2019; **Location:** continent: Central America; country: Costa Rica; countryCode: CR; stateProvince: Puntarenas; verbatimLocality: Monteverde; verbatimElevation: 1500; **Identification:** identifiedBy: AJ Fleming; dateIdentified: 2019; **Event:** samplingProtocol: Hand collected; verbatimEventDate: 28-Aug-1993; **Record Level:** language: en; institutionCode: CNC; collectionCode: Insects; basisOfRecord: Pinned Specimen
- **Type status:** Paratype. **Occurrence:** recordedBy: D.M. Wood; sex: M; lifeStage: adult; preparations: pinned; **Taxon:** scientificName: Telothyria clavata; phylum: Arthropoda; class: Insecta; order: Diptera; family: Tachinidae; genus: Telothyria; specificEpithet: clavata; scientificNameAuthorship: Fleming & Wood, 2019; **Location:** continent: Central America; country: Costa Rica; countryCode: CR; stateProvince: Puntarenas; verbatimLocality: Monteverde; verbatimElevation: 1500; **Identification:** identifiedBy: AJ Fleming; dateIdentified: 2019; **Event:** samplingProtocol: Hand collected; verbatimEventDate: 28-Aug-1993; **Record Level:** language: en; institutionCode: CNC; collectionCode: Insects; basisOfRecord: Pinned Specimen

#### Description

**Male.** Length: 10–11mm (Fig. 11). **Head** (Fig. 11b): frons narrow, 1/5 of head width; gena 1/8 of head height; two reclinate orbital setae; anteriormost reclinate orbital almost equal to uppermost frontal seta; ocellar setae absent, or apparently absent; outer vertical seta absent; fronto-orbital plate brassy-gold throughout; fronto-orbital plate with short black hairs interspersed among frontal setae; parafacial brassy-gold, almost concolorous with fronto-orbital plate; facial ridge bare; palpus long slender digitiform with slight upward turn apically, sparsely haired along outer margin; arista orange-brown, smoothly tapered, microtrichia at most 3X as long as width of arista, concolorous with postpedicel; postpedicel only 30% orange, directly adjacent to pedicel; postocular region behind margin of eye including gena gold tomentose; occiput dark grey to silver tomentose. **Thorax** (Fig. 11a, c): pale brassy tomentose, with four thick and distinct dorsal stripes, bearing a basal dark dorsomedial stripe on postsutural scutum directly adjacent to scutellum; plumose blonde hairs absent from disc of scutum, punctuated on anepisternum at base of postpronotum with a spot of long brown plumose hairs, dorsally thorax densely covered in black hairs; chaetotaxy: 4–5 postpronotal setae, basal setae arranged in a straight line; supra-alar setae 2:3; intra-alar setae 2:3; dorsocentral setae 3:3; acrostichal setae 4:4; katepisternum with three setae. Scutellum dark brown ground color with brassy tomentosity along margin 20%; two pairs of strong marginal setae (basal and subapical) and a small pair of crossed apical scutellar setae 1/5th as long as subapical scutellars; basal scutellar setae subequal in length to subapical setae; subapical setae straight; underside of scutellum bearing regular non-plumose black hairs below basal scutellar setae. **Legs**: all legs dark brown ground color; anterior leg tibia with regular fringe of equally spaced setae along anteroventral surface, one posterodorsal setae. **Wings**: basicosta brown; all smoky-brown slightly infuscate all veins bare, with only one setula at base of R_4+5_; calypters brassy brown, lower calypter with a narrow yellow fringe, upper calypter with a narrow brown fringe. **Abdomen** (Fig. 11a, c): ground color bright orange, with orange to maroon spots apparent when viewed dorsally; ST1+2 maroon over dorsomedial 40%, T3 and T4 each with only some dark brown spots present dorsomedially; light silver tomentum along T3–T5, extending over entire tergite appearing to have a silver sheen when viewed with under certain angles of light; T5 orange ground color with a slightly darker apex and a light silver tomentum; median marginal setae present only on T4 and T5; median discal setae absent. **Male terminalia** (Fig. 11d, e, f): Sternite 5 with a wide deeply excavated median cleft, smoothly V-shaped, margins covered in dense pollinosity; lateral lobes of sternite rounded apically, with a group of strong setulae along outer margins; basal section of sternite 5 subequal to slightly longer than length of apical lobes. Cerci in posterior view sharply pointed triangular sharply widening to a rectangular shoulder along the basal section, equal in length to surstyli, fused along entire length; in lateral view, with a slight downward angle on apical 1/3; when viewed dorsally entire genital capsule can be said to be quite hirsute bearing several strong setulae throughout. Surstylus in lateral view, almost equilateral along its length rounded and downwardly curved at tip, appearing digitiform; surstylus appearing fused with epandrium; when viewed dorsally surstyli appear slender with a slight inward curve at apices. Pregonite short and not very well developed, apically rounded, bare. Postgonite, elongate and slender, sharply pointed at its tip, subequal in length to pregonite. Basiphallus long and slender, as a short humplike process. Distiphallus subequal in length to basiphallus and tubular, slightly pointed at apex.

**Female.** Unknown at this time.

#### Diagnosis

Very distinctive species of *Telothyria* can be distinguished from its congeners by the following combination of traits: ocellar setae absent, or apparently absent, fronto-orbital plate and parafacial pale brassy-gold, with four thick and distinct dorsal stripes, bearing a basal dark dorsomedial stripe on postsutural scutum directly adjacent to scutellum, plumose blonde hairs absent from disc of scutum, punctuated on anepisternum at base of postpronotum with a spot of long brown plumose hairs, dorsally thorax densely covered in black hairs, katepisternum with three setae, and entire abdomen bright orange.

#### Etymology

*Telothyria clavata* **sp. n.** From the Latin noun, “*clavus*” for the stripes on the tunics of Roman senators, in reference to the uniquely bold dorsal stripes.

#### Distribution

Costa Rica, Puntarenas Province, Monteverde 1400–1500 m elevation.

#### Ecology

Specimens hand collected once, ecology not available.

### Telothyria cristata

Fleming & Wood sp. n.

52E491CA-7335-53AC-BD60-50D5B6E5E6A6

urn:lsid:zoobank.org:act:891486E6-60C5-4937-9DDF-A4348716C7F5

#### Materials

- **Type status:** Holotype. **Occurrence:** occurrenceDetails: http://janzen.sas.upenn.edu; catalogNumber: DHJPAR0016499; recordedBy: D.H. Janzen, W. Hallwachs & Elda Araya; individualID: DHJPAR0016499; individualCount: 1; sex: M; lifeStage: adult; preparations: pinned; otherCatalogNumbers: ASTAP703-07, 06-SRNP-67877, BOLD:AAE9747; **Taxon:** scientificName: Telothyria cristata; phylum: Arthropoda; class: Insecta; order: Diptera; family: Tachinidae; genus: Telothyria; specificEpithet: cristata; scientificNameAuthorship: Fleming & Wood, 2018; **Location:** continent: Central America; country: Costa Rica; countryCode: CR; stateProvince: Alajuela; county: Buenos Aires; locality: Area de Conservacion Guanacaste; verbatimLocality: Finca Tomate; verbatimElevation: 360; verbatimLatitude: 10.9035; verbatimLongitude: -85.3092; verbatimCoordinateSystem: Decimal; decimalLatitude: 10.9035; decimalLongitude: -85.3092; **Identification:** identifiedBy: AJ Fleming; dateIdentified: 2018; **Event:** samplingProtocol: Reared from the larva of the Crambidae, Rhectocraspeda Janzen42; verbatimEventDate: 22-Jan-2007; **Record Level:** language: en; institutionCode: CNC; collectionCode: Insects; basisOfRecord: Pinned Specimen
- **Type status:** Paratype. **Occurrence:** occurrenceDetails: http://janzen.sas.upenn.edu; catalogNumber: DHJPAR0016505; recordedBy: D.H. Janzen, W. Hallwachs & Anabelle Cordoba; individualID: DHJPAR0016505; individualCount: 1; sex: F; lifeStage: adult; preparations: pinned; otherCatalogNumbers: ASTAP709-07, 06-SRNP-67868, BOLD:AAE9747; **Taxon:** scientificName: Telothyria cristata; phylum: Arthropoda; class: Insecta; order: Diptera; family: Tachinidae; genus: Telothyria; specificEpithet: cristata; scientificNameAuthorship: Fleming & Wood, 2018; **Location:** continent: Central America; country: Costa Rica; countryCode: CR; stateProvince: Alajuela; county: Buenos Aires; locality: Area de Conservacion Guanacaste; verbatimLocality: Finca Tomate; verbatimElevation: 360; verbatimLatitude: 10.9035; verbatimLongitude: -85.3092; verbatimCoordinateSystem: Decimal; decimalLatitude: 10.9035; decimalLongitude: -85.3092; **Identification:** identifiedBy: AJ Fleming; dateIdentified: 2018; **Event:** samplingProtocol: Reared from the larva of the Crambidae, Rhectocraspeda Solis05; verbatimEventDate: 24-Jan-2007; **Record Level:** language: en; institutionCode: CNC; collectionCode: Insects; basisOfRecord: Pinned Specimen
- **Type status:** Paratype. **Occurrence:** occurrenceDetails: http://janzen.sas.upenn.edu; catalogNumber: DHJPAR0016508; recordedBy: D.H. Janzen, W. Hallwachs & Carolina Cano; individualID: DHJPAR0016508; individualCount: 1; sex: M; lifeStage: adult; preparations: pinned; otherCatalogNumbers: ASTAP712-07, 06-SRNP-67864, BOLD:AAE9747; **Taxon:** scientificName: Telothyria cristata; phylum: Arthropoda; class: Insecta; order: Diptera; family: Tachinidae; genus: Telothyria; specificEpithet: cristata; scientificNameAuthorship: Fleming & Wood, 2018; **Location:** continent: Central America; country: Costa Rica; countryCode: CR; stateProvince: Alajuela; county: Buenos Aires; locality: Area de Conservacion Guanacaste; verbatimLocality: Finca Tomate; verbatimElevation: 360; verbatimLatitude: 10.9035; verbatimLongitude: -85.3092; verbatimCoordinateSystem: Decimal; decimalLatitude: 10.9035; decimalLongitude: -85.3092; **Identification:** identifiedBy: AJ Fleming; dateIdentified: 2018; **Event:** samplingProtocol: Reared from the larva of the Crambidae, Rhectocraspeda Janzen42; verbatimEventDate: 25-Jan-2007; **Record Level:** language: en; institutionCode: CNC; collectionCode: Insects; basisOfRecord: Pinned Specimen
- **Type status:** Paratype. **Occurrence:** occurrenceDetails: http://janzen.sas.upenn.edu; catalogNumber: DHJPAR0034513; recordedBy: D.H. Janzen, W. Hallwachs & Elda Araya; individualID: DHJPAR0034513; individualCount: 1; sex: M; lifeStage: adult; preparations: pinned; otherCatalogNumbers: ASHYC1165-09, 09-SRNP-1132, BOLD:AAE9747; **Taxon:** scientificName: Telothyria cristata; phylum: Arthropoda; class: Insecta; order: Diptera; family: Tachinidae; genus: Telothyria; specificEpithet: cristata; scientificNameAuthorship: Fleming & Wood, 2018; **Location:** continent: Central America; country: Costa Rica; countryCode: CR; stateProvince: Alajuela; county: Sector San Cristobal; locality: Area de Conservacion Guanacaste; verbatimLocality: Rio Blanco Abajo; verbatimElevation: 500; verbatimLatitude: 10.9004; verbatimLongitude: -85.3725; verbatimCoordinateSystem: Decimal; decimalLatitude: 10.9004; decimalLongitude: -85.3725; **Identification:** identifiedBy: AJ Fleming; dateIdentified: 2018; **Event:** samplingProtocol: Reared from the larva of the Crambidae, Herpetogramma Solis11; verbatimEventDate: 08-Apr-2009; **Record Level:** language: en; institutionCode: CNC; collectionCode: Insects; basisOfRecord: Pinned Specimen
- **Type status:** Paratype. **Occurrence:** occurrenceDetails: http://janzen.sas.upenn.edu; catalogNumber: DHJPAR0037443; recordedBy: D.H. Janzen, W. Hallwachs & Elieth Cantillano; individualID: DHJPAR0037443; individualCount: 1; sex: M; lifeStage: adult; preparations: pinned; otherCatalogNumbers: ASHYC4188-10, 09-SRNP-23251, BOLD:AAE9747; **Taxon:** scientificName: Telothyria cristata; phylum: Arthropoda; class: Insecta; order: Diptera; family: Tachinidae; genus: Telothyria; specificEpithet: cristata; scientificNameAuthorship: Fleming & Wood, 2018; **Location:** continent: Central America; country: Costa Rica; countryCode: CR; stateProvince: Guanacaste; county: Sector Del Oro; locality: Area de Conservacion Guanacaste; verbatimLocality: Sendero Puertas; verbatimElevation: 400; verbatimLatitude: 11.0109; verbatimLongitude: -85.4882; verbatimCoordinateSystem: Decimal; decimalLatitude: 11.0109; decimalLongitude: -85.4882; **Identification:** identifiedBy: AJ Fleming; dateIdentified: 2018; **Event:** samplingProtocol: Reared from the larva of the Crambidae, Desmia benealisDHJ02; verbatimEventDate: 01-Nov-2009; **Record Level:** language: en; institutionCode: CNC; collectionCode: Insects; basisOfRecord: Pinned Specimen

#### Description

**Male.** Length: 7–9 mm (Fig. 12). **Head** (Fig. 12b): frons narrow, 1/6 of head width; gena 1/8 of head height; three reclinate orbital setae; anteriormost reclinate orbital almost equal to uppermost frontal seta; ocellar setae absent; outer vertical seta absent; fronto-orbital plate brassy-gold throughout; vertex and ocellar triangle black; fronto-orbital plate with short blonde hairs interspersed among frontal setae; parafacial pale brassy-gold; facial ridge bare; palpus short digitiform with slight upward turn apically, sparsely haired along outer margin; arista brown, smoothly tapered, microtrichia at most 3X as long as width of arista; postpedicel only 30% orange, directly adjacent to pedicel; postocular region behind margin of eye including upper hald of gena gold tomentose, lower half of gena silver tomentose; upper 1/4 of occiput gold tomentose. **Thorax** (Fig. 12a, c): gray tomentose, with two almost indistinct outer dorsal stripes, and inner stripes not evident; thorax laterally covered in dense plumose blonde hairs; chaetotaxy: 4–5 postpronotal setae, basal setae arranged in a straight line; supra-alar setae 1:3; intra-alar setae 1:2; dorsocentral setae 3:3; acrostichal setae 3:3; katepisternum with three setae. Scutellum gray tomentose; two pairs of strong marginal setae (basal and subapical) and a small pair of crossed apical scutellar setae 1/5th as long as subapical scutellars; basal scutellar setae subequal in length to subapical setae; subapical setae straight; underside of scutellum bearing regular non-plumose black hairs below basal scutellar setae. **Legs**: all legs dark brown ground color throughout; anterior leg tibia with irregularly sized fringe of equally spaced setae along anterodorsal surface, one posterodorsal seta. **Wings**: basicosta dark brown, wing slightly infuscate, almost imperceptibly so, amber color overall; all veins bare, with only 1–2 setulae at base of R_4+5_; calypters cinereous translucent with a thin brown fringe on lower calypter. **Abdomen** (Fig. 12a, c): ground maroon apparent when viewed dorsally with dark burnt orange color along lateral surfaces; ST1+2 maroon over 90%, with yellow spots, T3 and T4 each with some orange present ventrolaterally; T3–T5 with dense silver tomentum along extending over entire tergite appearing to have a silver sheen when viewed with the naked eye; T5 maroon ground color with gold tomentum; median marginal setae present only on T4 and T5; median discal setae absent. **Male terminalia** (Fig. 12d, e, f): Sternite 5 with an extremely wide and deeply separated median cleft, V-shaped, margins tomentose; lateral lobes of sternite outwardly pointed subtriangular apically, outer margins covered in a crest of strong setae; basal section of sternite 5 1/3 as long as length of apical lobes. Cerci in posterior view sharply pointed along apical half, basal half rectangular with a widened shoulder medially, equal in length to surstyli, fused along entire length. In lateral view cerci, with a strong downward curve, and several strong widely spaced setae along basal 2/3rds. Surstylus in lateral view bluntly rounded at tip, slightly downwardly pointed but not curved, overall digitiform in appearance; fused with epandrium; when viewed dorsally surstyli appear robust and straight with a very slight club apically. Basiphallus short and stout and stout, distiphallus subequal to in length to basiphallus, weakly tapering apically.

**Female.** Length: 5–7 mm (Fig. 13). **Head** (Fig. 13b): as in male with the following exceptions: ocellar triangle dark brassy; fronto-orbital plate gray; parafacial silvery–gray; frons 1/5 of head width; 2–4 inner reclinate orbital setae; two proclinate orbital setae; outer vertical seta present; palpus long and clubbed bare apically. **Thorax** (Fig. 13a, c): katepisternum with three setae; meron with 9–12 typical meral setae and some long blonde hairs along anterior edge. Legs: colored as in male. **Abdomen** (Fig. 13a, c): ground color as in male; T3–T5 with silver tomentum along anterior edge of tergites; T5 as in male; marginal setae present on T4, T5 only.

#### Diagnosis

*Telothyria cristata* **sp. n.** can be distinguished from all other *Telothyria* by the following combination of traits: ocellar setae absent, parafacial entirely gold, silvery–gray in females, postpedicel only 30% orange, directly adjacent to pedicel, thorax with only two outer stripes evident, plumose blonde hairs absent from disc of scutum, abdominal ground color dark maroon to blackish under certain angles of light, with dark orange lateroventrally from ST1+2–T5, and T5 maroon with silver tomentum. *T. cristata* differs from its closest congener *T. cupreiventris* Van der Wulp, by the presence of orange along the lateral surfaces of the abdomen in both males and females.

#### Etymology

*Telothyria cristata* **sp. n.** From the Latin adjective “*cristatum*” meaning crested, in reference to the crest of hairs that line ST5 in the male terminalia.

#### Distribution

Costa Rica, ACG, Alajuela Province, 360–500 m elevation.

#### Ecology

*Telothyria cristata* **sp. n.** has been reared six times from six species of Lepidoptera in the family Crambidae: *Pilemia* Janzen42, *Pilemia periusalis*, *Piletosoma thialis*, *Desmia benealis*DHJ02, *Herpetogramma* Solis11 in dry forest, rain forest, and dry-rain lowland intergrade.

### Telothyria cupreiventris

van der Wulp, 1890

0A1DC1C2-115B-5B0C-B2A3-155C9BBF4D06

Telothyria cupreiventris van der Wulp, 1890: 169 in key [also 1890: 182 in description]. Lectotype male [not female as published, Townsend 1931: 91] (BMNH), by fixation of Townsend (1931: 91). Type locality: Mexico, Tabasco, Teapa.

#### Description

**Male.** Length: 7–9 mm (Fig. 14). **Head** (Fig. 14b): frons narrow, 1/5 of head width; gena 1/15 of head height; 3–4 reclinate orbital setae; anteriormost reclinate orbital almost subequal to uppermost frontal seta; ocellar setae absent; outer vertical seta absent; fronto-orbital plate brassy-gold throughout; fronto-orbital plate with short reddish hairs interspersed among frontal setae; parafacial pale brassy-gold; facial ridge bare; palpus short digitiform with slight upward turn apically, sparsely haired along outer margin; arista brown, smoothly tapered, microtrichia at most 3X as long as width of arista; postpedicel only 30% dark burnt-orange, directly adjacent to pedicel. **Thorax** (Fig. 14a, c): gray tomentose, with two almost indistinct outer dorsal stripes, and inner stripes not evident, these truncated and only slightly evident; thorax laterally covered in dense plumose reddish-brown hairs; chaetotaxy: 4–5 postpronotal setae, basal setae arranged in a straight line; supra-alar setae 2:3; intra-alar setae 1:2; dorsocentral setae 3:3; acrostichal setae 3:3; katepisternum with 3–5 setae. Scutellum reddish-brown glabrous, lacking any obvious tomentum; two pairs of strong marginal setae (basal and subapical) and a small pair of crossed apical scutellar setae 1/5th as long as subapical scutellars; basal scutellar setae subequal in length to subapical setae; subapical setae straight. **Legs**: all legs dark brown ground color throughout. **Wings**: smoky brown infuscate; basicosta dark brown; all veins bare, with only 1–2 setulae at base of R_4+5_. **Abdomen** (Fig. 14a, c): ground color dark maroon apparent when viewed dorsally; ST1+2–T5 maroon throughout, lacking any yellow or orange spots; T3–T5 with dense silver tomentum extending over entire tergite appearing to have a silver sheen when viewed with the naked eye; T5 maroon ground color with gold tomentum; median marginal setae present only on T4 and T5; median discal setae absent. **Terminalia**: not examined.

**Female.** Length: 5–7 mm (Fig. 15). **Head** (Fig. 15b): as in male with the following exceptions: fronto-orbital plate gray; parafacial silvery–gray; frons 1/5 of head width; 2–4 inner reclinate orbital setae; two proclinate orbital setae; outer vertical seta present; palpus long and clubbed bare apically. **Thorax** (Fig. 15a, c): katepisternum with three setae; meron with 9–12 typical meral setae. Legs: colored as in male. **Abdomen** (Fig. 15a, c): ground color as in male; T3–T5 with silver tomentum along anterior edge of tergites; T5 as in male; marginal setae present on T4, T5 only.

#### Diagnosis

*Telothyria cupreiventris* can be distinguished from all other *Telothyria* by the following combination of traits: ocellar setae absent, parafacial entirely gold, silvery–gray in the female, postpedicel only 30% dark burnt-orange, directly adjacent to pedicel, thoracic stripes truncated and only slightly evident, plumose hairs on thorax absent from disc of scutum, lateral plumose hairs reddish-brown mixed with blonde, abdominal ground color dark maroon appearing blackish under certain angles of light, without any traces of orange lateroventrally, and T5 with silver tomentum. *Telothyria cupreiventris* differs from *T. cristata* by the entirely maroon abdomen, and its reddish-brown plumose hairs present on lateral surfaces of thorax.

#### Distribution

Mexico, Tabasco and Veracruz.

#### Ecology

Ecology of *Telothyria cupreiventris* van der Wulp unknown.

### Telothyria diniamartinezae

Fleming & Wood sp. n.

BF3E1032-9AC8-5556-B0DB-F9020DBE72F7

urn:lsid:zoobank.org:act:FFA23D62-770E-4A94-A748-FE7113DDD243

#### Materials

- **Type status:** Holotype. **Occurrence:** occurrenceDetails: http://janzen.sas.upenn.edu; catalogNumber: DHJPAR0050693; recordedBy: D.H. Janzen, W. Hallwachs & Anabelle Cordoba; individualID: DHJPAR0050693; individualCount: 1; sex: F; lifeStage: adult; preparations: pinned; otherCatalogNumbers: ACGBA3285-13, 12-SRNP-86004, BOLD:ACJ2139; **Taxon:** scientificName: Telothyria diniamartinezae; phylum: Arthropoda; class: Insecta; order: Diptera; family: Tachinidae; genus: Telothyria; specificEpithet: diniamartinezae; scientificNameAuthorship: Fleming & Wood, 2018; **Location:** continent: Central America; country: Costa Rica; countryCode: CR; stateProvince: Alajuela; county: Sector Rincon Rain Forest; locality: Area de Conservacion Guanacaste; verbatimLocality: Sendero Juntas; verbatimElevation: 400; verbatimLatitude: 10.9066; verbatimLongitude: -85.2878; verbatimCoordinateSystem: Decimal; decimalLatitude: 10.9066; decimalLongitude: -85.2878; **Identification:** identifiedBy: AJ Fleming; dateIdentified: 2018; **Event:** samplingProtocol: Reared from the larva of the Crambidae, Neoleucinodes Janzen02; verbatimEventDate: 11-Nov-2012; **Record Level:** language: en; institutionCode: CNC; collectionCode: Insects; basisOfRecord: Pinned Specimen

#### Description

**Female.** Length: 5 mm (Fig. 16). **Head** (Fig. 16b): frons 1/3 of head width; gena 1/10 of head height; three pairs of reclinate inner orbital setae uppermost pair slightly convergent, and two proclinate orbital setae; ocellar setae minimal but present; outer vertical seta present; fronto-orbital plate pale brassy-gold along upper half inclusive of ocellar triangle; fronto-orbital plate with short blonde hairs interspersed among frontal setae; lower half of fronto-orbital plate and entire parafacial brilliant silver; facial ridge bare; palpus bearing a very slight apical club and slightly upturned; arista brown, apically turning almost orange at base, smoothly tapering to apical 1/8, microtrichia shorter than width of arista; postpedicel orange over at most 30% of surface; postocular region behind margin of eye upper 2/3 gold, with lower 1/3 including gena silver tomentose; upper 3/4 of occiput gold tomentose. **Thorax** (Fig. 16a, c): brassy-gold tomentose, with four distinct thoracic stripes outer pair broken across suture; thorax covered in dense plumose blonde hairs laterally; chaetotaxy: four postpronotal setae, basal setae arranged in a straight line; supra-alar setae 2:3; intra-alar setae 2:3; dorsocentral setae 3:3; acrostichal setae 3: (unknown as holotype is damaged); katepisternum with three setae; meron lacking plumose hairs with six typical meral setae. Scutellum brassy-gold tomentose; two pairs of strong marginal setae (basal and subapical) and a small pair of parallel apical scutellar setae 1/8–1/10th as long as subapical scutellars; basal scutellar setae subequal in length to subapical setae; subapical setae convergent; underside of scutellum bearing plumose blonde hairs below basal scutellar setae. **Legs**: foreleg with yellow ground color on coxa and femur, tibia and tarsal segments darkened due to hair covering; midleg and hindleg with yellow coxae, pale brown femur, and dark brown remainder; anterior leg tibia with regular fringe of equally spaced setae along anteroventral surface, one posterodorsal setae. **Wings**: basicosta beige; all veins bare, with only one setula at base of R_4+5_, present on both dorsal and ventral surfaces; calypters pale white translucent. **Abdomen** (Fig. 16a, c): holotype female abdomen damaged, making the discerning of features difficult. Ground color dark brown dorsally with yellow-orange present ventrolaterally; T1+2–T4 with gold-silver tomentum extending over up to 50% of tergite; T4 entirely dark brown in ground color; T5 with a bronze-brown tomentum, and dark brown ground color basally turning orange apically; marginal setae present on T4 and T5; median discal setae absent.

**Male.** Unknown at this time.

#### Diagnosis

*Telothyria diniamartinezae* **sp. n.** can be distinguished from all other *Telothyria* by the following combination of traits: ocellar setae minimal but present, plumose hairs on thorax absent from disc of scutum, katepisternum with three setae, three postsutural intra-alar setae, legs with yellow coxa, T4 entirely dark brown, and T5 mostly dark brown with orange apically and covered in bronze-brown tomentum.

#### Etymology

*Telothyria diniamartinezae* **sp. n.** is named in recognition of Dinia Martinez's outstanding work on the team that conducts the caterpillar and parasite inventory from ACG’s Estación Biológica Quica.

#### Distribution

Costa Rica, ACG, Alajuela Province, 400 m elevation.

#### Ecology

*Telothyria diniamartinezae* **sp. n.** has been reared once from a single species of Lepidoptera in the family Crambidae: *Neoleucinodes* Janzen02, in rain forest.

### Telothyria duniagarciae

Fleming & Wood sp. n.

6109E20C-39FA-559C-98A9-DE18440A2FC2

urn:lsid:zoobank.org:act:6E9B1ABB-989F-4132-9000-EA44A2FBCC59

#### Materials

- **Type status:** Holotype. **Occurrence:** occurrenceDetails: http://janzen.sas.upenn.edu; catalogNumber: DHJPAR0052058; recordedBy: D.H. Janzen, W. Hallwachs & Manuel Rios; individualID: DHJPAR0052058; individualCount: 1; sex: M; lifeStage: adult; preparations: pinned; otherCatalogNumbers: ASHYH1170-13, 13-SRNP-30774, BOLD:ACI1191; **Taxon:** scientificName: Telothyria duniagarciae; phylum: Arthropoda; class: Insecta; order: Diptera; family: Tachinidae; genus: Telothyria; specificEpithet: duniagarciae; scientificNameAuthorship: Fleming & Wood, 2018; **Location:** continent: Central America; country: Costa Rica; countryCode: CR; stateProvince: Guanacaste; county: Sector Pitilla; locality: Area de Conservacion Guanacaste; verbatimLocality: Colocho; verbatimElevation: 375; verbatimLatitude: 11.0237; verbatimLongitude: -85.4188; verbatimCoordinateSystem: Decimal; decimalLatitude: 11.0237; decimalLongitude: -85.4188; **Identification:** identifiedBy: AJ Fleming; dateIdentified: 2018; **Event:** samplingProtocol: Reared from the larva of the Crambidae, Ategumia Solis01; verbatimEventDate: 24-Jun-2013; **Record Level:** language: en; institutionCode: CNC; collectionCode: Insects; basisOfRecord: Pinned Specimen
- **Type status:** Paratype. **Occurrence:** occurrenceDetails: http://janzen.sas.upenn.edu; catalogNumber: DHJPAR0056114; recordedBy: D.H. Janzen, W. Hallwachs & Manuel Rios; individualID: DHJPAR0056114; individualCount: 1; sex: F; lifeStage: adult; preparations: pinned; otherCatalogNumbers: ASHYH2371-14, 14-SRNP-80910, BOLD:ACI1191; **Taxon:** scientificName: Telothyria duniagarciae; phylum: Arthropoda; class: Insecta; order: Diptera; family: Tachinidae; genus: Telothyria; specificEpithet: duniagarciae; scientificNameAuthorship: Fleming & Wood, 2018; **Location:** continent: Central America; country: Costa Rica; countryCode: CR; stateProvince: Alajuela; county: Sector Rincon Rain Forest; locality: Area de Conservacion Guanacaste; verbatimLocality: Selva; verbatimElevation: 410; verbatimLatitude: 10.9229; verbatimLongitude: -85.3188; verbatimCoordinateSystem: Decimal; decimalLatitude: 10.9229; decimalLongitude: -85.3188; **Identification:** identifiedBy: AJ Fleming; dateIdentified: 2018; **Event:** samplingProtocol: Reared from the larva of the Crambidae, Ategumia Solis01; verbatimEventDate: 08-Aug-2014; **Record Level:** language: en; institutionCode: CNC; collectionCode: Insects; basisOfRecord: Pinned Specimen
- **Type status:** Paratype. **Occurrence:** occurrenceDetails: http://janzen.sas.upenn.edu; catalogNumber: DHJPAR0057130; recordedBy: D.H. Janzen, W. Hallwachs & Keiner Aragon; individualID: DHJPAR0057130; individualCount: 1; sex: F; lifeStage: adult; preparations: pinned; otherCatalogNumbers: ACGBA5040-15, 14-SRNP-47584, BOLD:ACI1191; **Taxon:** scientificName: Telothyria duniagarciae; phylum: Arthropoda; class: Insecta; order: Diptera; family: Tachinidae; genus: Telothyria; specificEpithet: duniagarciae; scientificNameAuthorship: Fleming & Wood, 2018; **Location:** continent: Central America; country: Costa Rica; countryCode: CR; stateProvince: Alajuela; county: Sector Rincon Rain Forest; locality: Area de Conservacion Guanacaste; verbatimLocality: Casa Keyner; verbatimElevation: 121; verbatimLatitude: 10.9564; verbatimLongitude: -85.2661; verbatimCoordinateSystem: Decimal; decimalLatitude: 10.9564; decimalLongitude: -85.2661; **Identification:** identifiedBy: AJ Fleming; dateIdentified: 2018; **Event:** samplingProtocol: Reared from the larva of the Crambidae, Ategumia Solis01; verbatimEventDate: 09-Dec-2014; **Record Level:** language: en; institutionCode: CNC; collectionCode: Insects; basisOfRecord: Pinned Specimen

#### Description

**Male.** Length: 8 mm (Fig. 17). **Head** (Fig. 17b): frons narrow, 1/5 of head width; gena 1/12 of head height; three reclinate orbital setae uppermost reclinate orbital pair slightly convergent; anteriormost reclinate orbital subequal in length to uppermost frontal seta; ocellar setae absent; outer vertical seta absent; ocellar triangle and fronto-orbital plate dark gold; fronto-orbital plate with short brown to black hairs interspersed among frontal setae; parafacial gold; facial ridge bare; palpus digitiform, apically terminating in a small bulbous club; arista brown, smoothly tapering to apical 1/8, microtrichia at most 1.5X as long as width of arista; pedicel orange and postpedicel orange over 60% of surface; postocular region behind margin of eye including gena gold tomentose; upper half of occiput gold tomentose, postgena silver tomentose. **Thorax** (Fig. 17a, c): brassy-gold tomentose, with two distinct outer dorsal stripes broken across suture, and two short inner stripes extending up to first postsutural dorsocentral seta; thorax covered in dense plumose blonde hairs laterally, plumose hairs on disc of scutum sparse, and mixed in with short black hairs; chaetotaxy: five postpronotal setae, basal setae arranged in a straight line; supra-alar setae 2:3; intra-alar setae 2:3; dorsocentral setae 3:3; acrostichal setae 3:3; katepisternum with three setae. Scutellum brassy-gold tomentose; two pairs of strong marginal setae (basal and subapical) and a small pair of crossed apical scutellar setae 1/8–1/10th as long as subapical scutellars; basal scutellar setae subequal in length to subapical setae; subapical setae straight; underside of scutellum bearing plumose blonde hairs below basal scutellar setae. **Legs**: foreleg ground color yellow on coxa and femur, appearing darker from tibia to tarsi; both midleg and hindleg dark brown entirely, with yellow coxae; anterior leg tibia with regular fringe of equally spaced setae along anteroventral surface, with one posterodorsal setae. **Wings**: basicosta brown; all veins bare, with only one setula at base of R_4+5_; calypters pale translucent with thin slightly orange fringe. **Abdomen** (Fig. 17a, c): ground color appearing brown-black dorsally with yellow-orange ventrolaterally; ST1+2 brown over medial 50%, with yellow ventrolaterally, extending into a longitudinal middorsal brown stripe bisected by a brown band along posterior edges of T3 and T4; T1+2–T4 with dense brassy tomentum extending over entire tergite; T5 brown ground color changing to dark orange apically, covered with gold tomentum; marginal setae present on T4 1/2 as long as those present on and T5; median discal setae absent. **Male terminalia**: not examined.

**Female.** Length: 6 mm (Fig. 18). **Head** (Fig. 18b): as in male with the following exceptions: fronto-orbital plate pale brassy gold over upper 50%; parafacial brilliant silver; frons 1/3 of head width; two inner reclinate orbital setae; two proclinate orbital setae; outer vertical seta present; palpus apically clubbed and distinctly upturned. **Thorax** (Fig. 18a, c): katepisternum with three setae; meron with only 10–12 typical meral setae. Legs: foreleg coxa with yellow ground color, femur yellow on ventral half, dark gray on posterior surfaces, yellow ground color but appearing darker from tibia to tarsal segments; both midleg and hindleg black throughout, with yellow coxae; anterior leg tibia with irregular tapered fringe of equally spaced setae along basal half of anteroventral surface, 2–3 anterodorsal setae, and 1–2 strong posterodorsal seta. **Abdomen** (Fig. 18a, c): ST1+2 and T3 50% brown dorsally, with yellow lateroventrally, T4 entirely brown, and T5 yellow-orange entirely.

#### Diagnosis

*Telothyria duniagarciae* **sp. n.** can be distinguished from all other *Telothyria* by the following combination of traits: ocellar setae absent, arista brown, with microtrichia at most 1.5X as long as width of arista, pedicel orange and postpedicel orange over 60% of surface, parafacial gold, silver in females, thorax covered in dense plumose blonde hairs laterally, plumose hairs on disc of scutum present yet sparse, and mixed in with short black hairs, katepisternum with three setae, legs yellow, abdominal ground color yellow-orange, and T5 yellow with silver tomentum.

#### Etymology

*Telothyria duniagarciae* **sp. n.** is named in recognition of Dunia Garcia's outstanding work on the team that conducts the caterpillar and parasite inventory from ACG’s Estación Biológica Cacao.

#### Distribution

Costa Rica, ACG, Alajuela and Guanacaste Provinces, 121–410 m elevation.

#### Ecology

*Telothyria duniagarciae* **sp. n.** has been reared three times from two species of Lepidoptera in the families Crambidae and Depressariidae: *Ategumia* Solis01, and *Filinota* Janzen154 respectively, in rain forest.

### Telothyria duvalierbricenoi

Fleming & Wood sp. n.

AB3B6F3E-D229-5890-8390-CC968D947295

urn:lsid:zoobank.org:act:8B406CFD-F613-4746-811F-D94729FD26C7

#### Materials

- **Type status:** Holotype. **Occurrence:** occurrenceDetails: http://janzen.sas.upenn.edu; catalogNumber: DHJPAR0055913; recordedBy: D.H. Janzen, W. Hallwachs & Cirilo Umana; individualID: DHJPAR0055913; individualCount: 1; sex: F; lifeStage: adult; preparations: pinned; otherCatalogNumbers: ASHYH2645-14, 14-SRNP-76251, BOLD:AAL7641; **Taxon:** scientificName: Telothyria duvalierbricenoi; phylum: Arthropoda; class: Insecta; order: Diptera; family: Tachinidae; genus: Telothyria; specificEpithet: duvalierbricenoi; scientificNameAuthorship: Fleming & Wood, 2018; **Location:** continent: Central America; country: Costa Rica; countryCode: CR; stateProvince: Alajuela; county: Sector Rincon Rain Forest; locality: Area de Conservacion Guanacaste; verbatimLocality: Quebrada Bambu; verbatimElevation: 109; verbatimLatitude: 10.9301; verbatimLongitude: -85.2521; verbatimCoordinateSystem: Decimal; decimalLatitude: 10.9301; decimalLongitude: -85.2521; **Identification:** identifiedBy: AJ Fleming; dateIdentified: 2018; **Event:** samplingProtocol: Reared from the larva of the Crambidae, Salbia cassidalis; verbatimEventDate: 10-Jul-2014; **Record Level:** language: en; institutionCode: CNC; collectionCode: Insects; basisOfRecord: Pinned Specimen

#### Description

**Female.** Length: 5 mm (Fig. 19). **Head** (Fig. 19b): frons narrow, 1/4 of head width; gena 1/10 of head height; three reclinate inner orbital setae uppermost reclinate orbital pair slightly convergent, and two proclinate orbital setae; ocellar setae absent; outer vertical seta present; fronto-orbital plate pale brassy-gold along upper third inclusive of ocellar triangle; fronto-orbital plate with short blonde hairs interspersed among frontal setae; parafacial brilliant silver, posterior half of gena concolorous silver tomentose; facial ridge bare; palpus apically clubbed and slightly upturned; arista brown, smoothly tapering to apical 1/8, microtrichia at most 1.5X as long as width of arista; pedicel orange, postpedicel orange over at most 30% of surface; postocular region behind margin of eye upper 1/3 gold, with lower 2/3 including gena silver tomentose; upper 1/3 of occiput gold tomentose. **Thorax** (Fig. 19a, c): brassy-gold tomentose dorsally, grey laterally, with four distinct thoracic stripes outer pair broken across suture; thorax covered in dense plumose blonde hairs laterally, short black hairs dorsally; chaetotaxy: 4–5 postpronotal setae, basal setae arranged in a straight line; supra-alar setae 2:3; intra-alar setae 2:3; dorsocentral setae 3:3; acrostichal setae 3:3; katepisternum with three setae; meron lacking plumose hairs with 4–5 typical meral setae. Scutellum brassy-gold tomentose; two pairs of strong marginal setae (basal and subapical) and a small pair of crossed apical scutellar setae 1/8–1/10th as long as subapical scutellars; basal scutellar setae subequal in length to subapical setae; subapical setae straight; underside of scutellum bearing plumose blonde hairs below basal scutellar setae. **Legs**: foreleg with yellow ground color throughout; midleg and hindleg bearing yellow coxae with dark yellow-brown femur, tibia, and tarsal segments; anterior leg tibia with regular tapered fringe of equally spaced setae along basal 1/3 of anteroventral surface, at most 3–4 setae and one strong posterodorsal seta. **Wings**: basicosta beige, with slight darkening to pale brown towards wing insertion; all veins bare, with only one setula at base of R_4+5_; calypters pale white translucent with a pale beige fringe. **Abdomen** (Fig. 19a, c): ground color mostly brown with yellow-orange present ventrolaterally; T1+2–T4 with gold tomentum at tergal margin changing to silver tomentum extending over up to 50% of tergite; T5 black marroon along anterior margin with yellow apically, tergite covered with silver tomentum along anterior 50%; marginal setae present on T4 and T5; median discal setae absent.

**Male.** Unknown at this time.

#### Diagnosis

*Telothyria duvalierbricenoi* **sp. n.** can be distinguished from all other *Telothyria* by the following combination of traits: ocellar setae absent, fronto-orbital plate mostly silver, pale brassy-gold along upper third inclusive of ocellar triangle, plumose hairs on thorax absent from disc of scutum, thorax brassy-gold tomentose dorsally, grey laterally, katepisternum with three setae, legs dark reddish-brown, abdominal ground color yellow-orange, and T5 black marroon along anterior margin with yellow apically, entirely covered in silver tomentum. Differentiates from *Telothyria insularis* Curran with its entirely silver gena, the presence of four thin dorsal stripes on the thorax, and the color of the coxae.

#### Etymology

*Telothyria duvalierbricenoi* **sp. n.** is named in recognition of Duvalier Briceño's outstanding work on managing the caterpillar and parasite inventory from his home and rearing barn in Brasilia, Alajuela Province. Costa Rica.

#### Distribution

Costa Rica, ACG, Alajuela Province, 109 m elevation.

#### Ecology

*Telothyria duvalierbricenoi* **sp. n.** has been reared once times from a single species of Lepidoptera in the family Crambidae: *Salbia cassidalis* (Guenée, 1854), in rain forest.

### Telothyria eldaarayae

Fleming & Wood sp. n.

0FCBB3B9-E1CC-5446-A1B9-F19B7EE7120B

urn:lsid:zoobank.org:act:0F16F033-CB8C-4CA0-975A-FE3A19BC4473

#### Materials

- **Type status:** Holotype. **Occurrence:** occurrenceDetails: http://janzen.sas.upenn.edu; catalogNumber: DHJPAR0050477; recordedBy: D.H. Janzen, W. Hallwachs & Keiner Aragon; individualID: DHJPAR0050477; individualCount: 1; sex: M; lifeStage: adult; preparations: pinned; otherCatalogNumbers: ACGBA3069-13, 13-SRNP-67007, BOLD:ACC0861; **Taxon:** scientificName: Telothyria *eldaarayae*; phylum: Arthropoda; class: Insecta; order: Diptera; family: Tachinidae; genus: Telothyria; specificEpithet: eldaarayae; scientificNameAuthorship: Fleming & Wood, 2018; **Location:** continent: Central America; country: Costa Rica; countryCode: CR; stateProvince: Alajuela; county: Sector Rincon Rain Forest; locality: Area de Conservacion Guanacaste; verbatimLocality: Palomo; verbatimElevation: 96; verbatimLatitude: 10.9619; verbatimLongitude: -85.2804; verbatimCoordinateSystem: Decimal; decimalLatitude: 10.9619; decimalLongitude: -85.2804; **Identification:** identifiedBy: AJ Fleming; dateIdentified: 2018; **Event:** samplingProtocol: Reared from the larva of the Crambidae, Salbia haemorrhoidalis; verbatimEventDate: 21-Jan-2013; **Record Level:** language: en; institutionCode: CNC; collectionCode: Insects; basisOfRecord: Pinned Specimen
- **Type status:** Paratype. **Occurrence:** occurrenceDetails: http://janzen.sas.upenn.edu; catalogNumber: DHJPAR0048500; recordedBy: D.H. Janzen, W. Hallwachs & Anabelle Cordoba; individualID: DHJPAR0048500; individualCount: 1; sex: F; lifeStage: adult; preparations: pinned; otherCatalogNumbers: ACGBA2042-12, 12-SRNP-41686, BOLD:ACC0861; **Taxon:** scientificName: Telothyria *eldaarayae*; phylum: Arthropoda; class: Insecta; order: Diptera; family: Tachinidae; genus: Telothyria; specificEpithet: eldaarayae; scientificNameAuthorship: Fleming & Wood, 2018; **Location:** continent: Central America; country: Costa Rica; countryCode: CR; stateProvince: Alajuela; county: Sector Rincon Rain Forest; locality: Area de Conservacion Guanacaste; verbatimLocality: Puente Rio Negro; verbatimElevation: 340; verbatimLatitude: 10.9038; verbatimLongitude: -85.3027; verbatimCoordinateSystem: Decimal; decimalLatitude: 10.9038; decimalLongitude: -85.3027; **Identification:** identifiedBy: AJ Fleming; dateIdentified: 2018; **Event:** samplingProtocol: Reared from the larva of the Crambidae, Salbia haemorrhoidalis; verbatimEventDate: 05-May-2012; **Record Level:** language: en; institutionCode: CNC; collectionCode: Insects; basisOfRecord: Pinned Specimen
- **Type status:** Paratype. **Occurrence:** occurrenceDetails: http://janzen.sas.upenn.edu; catalogNumber: DHJPAR0050243; recordedBy: D.H. Janzen, W. Hallwachs & Keiner Aragon; individualID: DHJPAR0050243; individualCount: 1; sex: M; lifeStage: adult; preparations: pinned; otherCatalogNumbers: ACGAZ1557-12, 12-SRNP-67345, BOLD:ACC0861; **Taxon:** scientificName: Telothyria *eldaarayae*; phylum: Arthropoda; class: Insecta; order: Diptera; family: Tachinidae; genus: Telothyria; specificEpithet: eldaarayae; scientificNameAuthorship: Fleming & Wood, 2018; **Location:** continent: Central America; country: Costa Rica; countryCode: CR; stateProvince: Alajuela; county: Sector Rincon Rain Forest; locality: Area de Conservacion Guanacaste; verbatimLocality: Estacion Botarrama; verbatimElevation: 160; verbatimLatitude: 10.9599; verbatimLongitude: -85.283; verbatimCoordinateSystem: Decimal; decimalLatitude: 10.9599; decimalLongitude: -85.283; **Identification:** identifiedBy: AJ Fleming; dateIdentified: 2018; **Event:** samplingProtocol: Reared from the larva of the Crambidae, Salbia haemorrhoidalis; verbatimEventDate: 10-Mar-2012; **Record Level:** language: en; institutionCode: CNC; collectionCode: Insects; basisOfRecord: Pinned Specimen
- **Type status:** Paratype. **Occurrence:** occurrenceDetails: http://janzen.sas.upenn.edu; catalogNumber: DHJPAR0050487; recordedBy: D.H. Janzen, W. Hallwachs & Edwin Apu; individualID: DHJPAR0050487; individualCount: 1; sex: F; lifeStage: adult; preparations: pinned; otherCatalogNumbers: ACGBA3079-13, 12-SRNP-82171, BOLD:ACC0861; **Taxon:** scientificName: Telothyria *eldaarayae*; phylum: Arthropoda; class: Insecta; order: Diptera; family: Tachinidae; genus: Telothyria; specificEpithet: eldaarayae; scientificNameAuthorship: Fleming & Wood, 2018; **Location:** continent: Central America; country: Costa Rica; countryCode: CR; stateProvince: Alajuela; county: Sector Rincon Rain Forest; locality: Area de Conservacion Guanacaste; verbatimLocality: Selva; verbatimElevation: 410; verbatimLatitude: 10.9229; verbatimLongitude: -85.3188; verbatimCoordinateSystem: Decimal; decimalLatitude: 10.9229; decimalLongitude: -85.3188; **Identification:** identifiedBy: AJ Fleming; dateIdentified: 2018; **Event:** samplingProtocol: Reared from the larva of the Crambidae, Salbia haemorrhoidalis; verbatimEventDate: 11-Jan-2013; **Record Level:** language: en; institutionCode: CNC; collectionCode: Insects; basisOfRecord: Pinned Specimen
- **Type status:** Paratype. **Occurrence:** occurrenceDetails: http://janzen.sas.upenn.edu; catalogNumber: DHJPAR0051596; recordedBy: D.H. Janzen, W. Hallwachs & Mercedes Moraga; individualID: DHJPAR0051596; individualCount: 1; sex: F; lifeStage: adult; preparations: pinned; otherCatalogNumbers: ACGBA4188-13, 13-SRNP-75466, BOLD:ACC0861; **Taxon:** scientificName: Telothyria *eldaarayae*; phylum: Arthropoda; class: Insecta; order: Diptera; family: Tachinidae; genus: Telothyria; specificEpithet: eldaarayae; scientificNameAuthorship: Fleming & Wood, 2018; **Location:** continent: Central America; country: Costa Rica; countryCode: CR; stateProvince: Alajuela; county: Sector Rincon Rain Forest; locality: Area de Conservacion Guanacaste; verbatimLocality: Finca Esmeralda; verbatimElevation: 123; verbatimLatitude: 10.9355; verbatimLongitude: -85.2531; verbatimCoordinateSystem: Decimal; decimalLatitude: 10.9355; decimalLongitude: -85.2531; **Identification:** identifiedBy: AJ Fleming; dateIdentified: 2018; **Event:** samplingProtocol: Reared from the larva of the Crambidae, Salbia haemorrhoidalis; verbatimEventDate: 28-Feb-2013; **Record Level:** language: en; institutionCode: CNC; collectionCode: Insects; basisOfRecord: Pinned Specimen
- **Type status:** Paratype. **Occurrence:** occurrenceDetails: http://janzen.sas.upenn.edu; catalogNumber: DHJPAR0057157; recordedBy: D.H. Janzen, W. Hallwachs & Edwin Apu; individualID: DHJPAR0057157; individualCount: 1; sex: M; lifeStage: adult; preparations: pinned; otherCatalogNumbers: ACGBA5067-15, 14-SRNP-81393, BOLD:ACC0861; **Taxon:** scientificName: Telothyria *eldaarayae*; phylum: Arthropoda; class: Insecta; order: Diptera; family: Tachinidae; genus: Telothyria; specificEpithet: eldaarayae; scientificNameAuthorship: Fleming & Wood, 2018; **Location:** continent: Central America; country: Costa Rica; countryCode: CR; stateProvince: Alajuela; county: Sector Rincon Rain Forest; locality: Area de Conservacion Guanacaste; verbatimLocality: Jacobo; verbatimElevation: 461; verbatimLatitude: 10.9408; verbatimLongitude: -85.3177; verbatimCoordinateSystem: Decimal; decimalLatitude: 10.9408; decimalLongitude: -85.3177; **Identification:** identifiedBy: AJ Fleming; dateIdentified: 2018; **Event:** samplingProtocol: Reared from the larva of the Crambidae, Salbia haemorrhoidalis; verbatimEventDate: 01-Nov-2014; **Record Level:** language: en; institutionCode: CNC; collectionCode: Insects; basisOfRecord: Pinned Specimen

#### Description

**Male.** Length: 6–7 mm (Fig. 20). **Head** (Fig. 20b): frons narrow, 1/6 of head width; gena less than 1/12 of head height; four reclinate orbital setae; anteriormost reclinate orbital shorter than uppermost frontal seta; ocellar setae present but minimal; outer vertical absent; ocellar triangle and fronto-orbital plate brassy-gold; fronto-orbital plate with short blonde hairs interspersed among frontal setae; parafacial brilliant silver; facial ridge bare; palpus oar-shaped, sparsely haired along outer margin; arista brown, smoothly tapering to apical 1/8, microtrichia at most 1X as long as width of arista; postpedicel orange over at most 30% of surface; postocular region behind margin of eye upper half gold, with lower half including gena silver tomentose; upper half of occiput gold tomentose. **Thorax** (Fig. 20a, c): brassy-gold tomentose, with two distinct outer dorsal stripes, and two short inner stripes; plumose hairs absent from disc of scutum; chaetotaxy: five postpronotal setae, basal setae arranged in a straight line; supra-alar setae 2:3; intra-alar setae 2:2; dorsocentral setae 2:3; acrostichal setae 3:3; katepisternum with three setae. Scutellum brassy-gold tomentose; two pairs of strong marginal setae (basal and subapical) and a small pair of crossed apical scutellar setae 1/8–1/10th as long as subapical scutellars; basal scutellar setae subequal in length to subapical setae; subapical setae straight; underside of scutellum bearing plumose blonde hairs below basal scutellar setae. **Legs**: foreleg with yellow ground color extending from coxa to tibia, tarsal segments darkened, posterodorsal surface with silver tomentum, and sparse short blonde hairs; midleg with yellow coxa, femur, tibia and tarsal segments dark brown with silver tomentum; hindleg dark brown extending from halfway along femur to tarsal segments, silver tomentum posterodorsally; anterior leg tibia with regular tapered fringe of equally spaced setae along basal half of anteroventral surface, and one strong posterodorsal seta up to 2X as long as width of tibia. **Wings**: basicosta bright ivory white; all veins bare, with only 1–2 setulae at base of R_4+5_; calypters pale white translucent with narrow yellow fringe. **Abdomen** (Fig. 20a, c): ground color dark yellow-orange; ST1+2 brown over medial 30%, with yellow ventrolaterally, extending into a wide longitudinal middorsal brown stripe bisected by a brown band along posterior edges of T3 and T4; entire abdomen covered in dense gold tomentum; T5 entirely yellow, covered with silver tomentum; median marginal setae present only on T4 and T5, those present on T4 variable in length from drastically reduced to strongly present; median discal setae absent. **Male terminalia** (Fig. 20d, e, f): Sternite 5 with a tight and shallow median cleft, narrowly V-shaped, margins bare; lateral lobes of sternite rounded subtriangular apically, outer margins devoid of setulae; basal section of sternite 5 1.3X longer than length of apical lobes. Cerci in posterior view sharply pointed triangular, equal in length to surstyli, fused along entire length; basal shoulder weakly developed almost absent. In lateral view with a strong downward curve on apical 1/3; several strong widely spaced setulae along basal 1/3rd. Surstylus in lateral view, almost subrectangular along its length rounded at tip, slightly pinched at midpoint appearing digitiform; surstylus appearing fused with epandrium; when viewed dorsally surstyli appear slender and straight with a club apically. Distiphallus subequal to in length to basiphallus, weakly tapering apically.

**Female.** Length: 5 mm (Fig. 21). **Head** (Fig. 21b): as in male with the following exceptions: fronto-orbital plate pale brassy gold over upper 30%; parafacial brilliant silver; frons 1/4 of head width; three inner reclinate orbital setae; three proclinate orbital setae; outer vertical seta present; palpus apically clubbed and distinctly upturned. **Thorax** (Fig. 21c): katepisternum with three setae; meron plumose hairs as well as 6–8 typical meral setae. Legs: colored as in male; anterior tibia with irregular tapering fringe of equally spaced setae along anteroventral surface, and one posterodorsal seta. **Abdomen** (Fig. 21a, c): ST1+2 and T3 50% brown dorsally, with yellow-orange lateroventrally, T4 entirely brown, and T5 yellow-orange entirely; T3 with single pair of weak almost hairlike median marginal setae.

#### Diagnosis

*Telothyria eldaarayae* **sp. n.** can be distinguished from all other *Telothyria* by the following combination of traits: ocellar setae present but minimal, plumose hairs absent from disc of scutum, katepisternum with three setae, two postsutural intra-alar setae, three postsutural intra-alar setae in females, and T5 yellow with silver tomentum. Can be distinguished from *T. minor* (Thompson) by the yellow ground color and tomentum present on the legs.

#### Etymology

*Telothyria eldaarayae* **sp. n.** is named in recognition of Elda Araya’s outstanding work on the team that conducts the caterpillar and parasite inventory from ACG’s Estación Biológica San Gerardo.

#### Distribution

Costa Rica, ACG, Alajuela Province, 123–461 m elevation.

#### Ecology

*Telothyria eldaarayae* **sp. n.** has been reared six times from a single species of Lepidoptera in the family Crambidae: *Salbia hameorrhoidalis* Guenée, 1854, in rain forest.

### Telothyria erythropyga

Fleming & Wood sp. n.

09E24D06-8AB8-5739-8B05-E319AB805717

urn:lsid:zoobank.org:act:FB7BF6A0-003A-4387-AA67-999D78DB99B4

#### Materials

- **Type status:** Holotype. **Occurrence:** occurrenceDetails: http://janzen.sas.upenn.edu; catalogNumber: DHJPAR0007125; recordedBy: D.H. Janzen, W. Hallwachs & Anabelle Cordoba; individualID: DHJPAR0007125; individualCount: 1; sex: M; lifeStage: adult; preparations: pinned; otherCatalogNumbers: ASTAV367-06, 06-SRNP-1595; **Taxon:** scientificName: Telothyria erythropyga; phylum: Arthropoda; class: Insecta; order: Diptera; family: Tachinidae; genus: Telothyria; specificEpithet: erythropyga; scientificNameAuthorship: Fleming & Wood, 2018; **Location:** continent: Central America; country: Costa Rica; countryCode: CR; stateProvince: Alajuela; county: Sector San Cristobal; locality: Area de Conservacion Guanacaste; verbatimLocality: Puente Palma; verbatimElevation: 460; verbatimLatitude: 10.9163; verbatimLongitude: -85.3787; verbatimCoordinateSystem: Decimal; decimalLatitude: 10.9163; decimalLongitude: -85.3787; **Identification:** identifiedBy: AJ Fleming; dateIdentified: 2018; **Event:** samplingProtocol: Reared from the larva of the Crambidae, Phostria Janzen03; verbatimEventDate: 21-Mar-2006; **Record Level:** language: en; institutionCode: CNC; collectionCode: Insects; basisOfRecord: Pinned Specimen
- **Type status:** Paratype. **Occurrence:** occurrenceDetails: http://janzen.sas.upenn.edu; catalogNumber: DHJPAR0011560; recordedBy: D.H. Janzen, W. Hallwachs & Jose Perez; individualID: DHJPAR0011560; individualCount: 1; sex: M; lifeStage: adult; preparations: pinned; otherCatalogNumbers: ASTAQ947-06, 05-SRNP-41630; **Taxon:** scientificName: Telothyria erythropyga; phylum: Arthropoda; class: Insecta; order: Diptera; family: Tachinidae; genus: Telothyria; specificEpithet: erythropyga; scientificNameAuthorship: Fleming & Wood, 2018; **Location:** continent: Central America; country: Costa Rica; countryCode: CR; stateProvince: Alajuela; county: Sector Rincon Rain Forest; locality: Area de Conservacion Guanacaste; verbatimLocality: Sendero Tucan; verbatimElevation: 410; verbatimLatitude: 10.9042; verbatimLongitude: -85.2712; verbatimCoordinateSystem: Decimal; decimalLatitude: 10.9042; decimalLongitude: -85.2712; **Identification:** identifiedBy: AJ Fleming; dateIdentified: 2018; **Event:** samplingProtocol: Reared from the larva of the Crambidae, Phostria Janzen05; verbatimEventDate: 21-Jul-2005; **Record Level:** language: en; institutionCode: CNC; collectionCode: Insects; basisOfRecord: Pinned Specimen
- **Type status:** Paratype. **Occurrence:** occurrenceDetails: http://janzen.sas.upenn.edu; catalogNumber: DHJPAR0011561; recordedBy: D.H. Janzen, W. Hallwachs & Jose Perez; individualID: DHJPAR0011561; individualCount: 1; sex: F; lifeStage: adult; preparations: pinned; otherCatalogNumbers: ASTAQ948-06, 05-SRNP-41628; **Taxon:** scientificName: Telothyria erythropyga; phylum: Arthropoda; class: Insecta; order: Diptera; family: Tachinidae; genus: Telothyria; specificEpithet: erythropyga; scientificNameAuthorship: Fleming & Wood, 2018; **Location:** continent: Central America; country: Costa Rica; countryCode: CR; stateProvince: Alajuela; county: Sector Rincon Rain Forest; locality: Area de Conservacion Guanacaste; verbatimLocality: Sendero Tucan; verbatimElevation: 410; verbatimLatitude: 10.9042; verbatimLongitude: -85.2712; verbatimCoordinateSystem: Decimal; decimalLatitude: 10.9042; decimalLongitude: -85.2712; **Identification:** identifiedBy: AJ Fleming; dateIdentified: 2018; **Event:** samplingProtocol: Reared from the larva of the Crambidae, Phostria Janzen05; verbatimEventDate: 18-Jul-2005; **Record Level:** language: en; institutionCode: CNC; collectionCode: Insects; basisOfRecord: Pinned Specimen
- **Type status:** Paratype. **Occurrence:** occurrenceDetails: http://janzen.sas.upenn.edu; catalogNumber: DHJPAR0011563; recordedBy: D.H. Janzen, W. Hallwachs & Jose Perez; individualID: DHJPAR0011563; individualCount: 1; sex: F; lifeStage: adult; preparations: pinned; otherCatalogNumbers: ASTAQ950-06, 05-SRNP-41977; **Taxon:** scientificName: Telothyria erythropyga; phylum: Arthropoda; class: Insecta; order: Diptera; family: Tachinidae; genus: Telothyria; specificEpithet: erythropyga; scientificNameAuthorship: Fleming & Wood, 2018; **Location:** continent: Central America; country: Costa Rica; countryCode: CR; stateProvince: Alajuela; county: Sector Rincon Rain Forest; locality: Area de Conservacion Guanacaste; verbatimLocality: Sendero Rincon; verbatimElevation: 430; verbatimLatitude: 10.8962; verbatimLongitude: -85.2777; verbatimCoordinateSystem: Decimal; decimalLatitude: 10.8962; decimalLongitude: -85.2777; **Identification:** identifiedBy: AJ Fleming; dateIdentified: 2018; **Event:** samplingProtocol: Reared from the larva of the Crambidae, Phostria Solis01; verbatimEventDate: 30-Aug-2005; **Record Level:** language: en; institutionCode: CNC; collectionCode: Insects; basisOfRecord: Pinned Specimen
- **Type status:** Paratype. **Occurrence:** occurrenceDetails: http://janzen.sas.upenn.edu; catalogNumber: DHJPAR0011564; recordedBy: D.H. Janzen, W. Hallwachs & Minor Carmona; individualID: DHJPAR0011564; individualCount: 1; sex: F; lifeStage: adult; preparations: pinned; otherCatalogNumbers: ASTAQ951-06, 05-SRNP-42282; **Taxon:** scientificName: Telothyria erythropyga; phylum: Arthropoda; class: Insecta; order: Diptera; family: Tachinidae; genus: Telothyria; specificEpithet: erythropyga; scientificNameAuthorship: Fleming & Wood, 2018; **Location:** continent: Central America; country: Costa Rica; countryCode: CR; stateProvince: Alajuela; county: Sector Rincon Rain Forest; locality: Area de Conservacion Guanacaste; verbatimLocality: Sendero Rincon; verbatimElevation: 430; verbatimLatitude: 10.8962; verbatimLongitude: -85.2777; verbatimCoordinateSystem: Decimal; decimalLatitude: 10.8962; decimalLongitude: -85.2777; **Identification:** identifiedBy: AJ Fleming; dateIdentified: 2018; **Event:** samplingProtocol: Reared from the larva of the Crambidae, Desmia Janzen576; verbatimEventDate: 21-Sep-2005; **Record Level:** language: en; institutionCode: CNC; collectionCode: Insects; basisOfRecord: Pinned Specimen
- **Type status:** Paratype. **Occurrence:** occurrenceDetails: http://janzen.sas.upenn.edu; catalogNumber: DHJPAR0011568; recordedBy: D.H. Janzen, W. Hallwachs & Jose Perez; individualID: DHJPAR0011568; individualCount: 1; sex: F; lifeStage: adult; preparations: pinned; otherCatalogNumbers: ASTAQ955-06, 04-SRNP-42880; **Taxon:** scientificName: Telothyria erythropyga; phylum: Arthropoda; class: Insecta; order: Diptera; family: Tachinidae; genus: Telothyria; specificEpithet: erythropyga; scientificNameAuthorship: Fleming & Wood, 2018; **Location:** continent: Central America; country: Costa Rica; countryCode: CR; stateProvince: Alajuela; county: Sector Rincon Rain Forest; locality: Area de Conservacion Guanacaste; verbatimLocality: Sendero Rincon; verbatimElevation: 430; verbatimLatitude: 10.8962; verbatimLongitude: -85.2777; verbatimCoordinateSystem: Decimal; decimalLatitude: 10.8962; decimalLongitude: -85.2777; **Identification:** identifiedBy: AJ Fleming; dateIdentified: 2018; **Event:** samplingProtocol: Reared from the larva of the Crambidae, Phostria Janzen05; verbatimEventDate: 10-Jan-2005; **Record Level:** language: en; institutionCode: CNC; collectionCode: Insects; basisOfRecord: Pinned Specimen
- **Type status:** Paratype. **Occurrence:** occurrenceDetails: http://janzen.sas.upenn.edu; catalogNumber: DHJPAR0016623; recordedBy: D.H. Janzen, W. Hallwachs & Jose Perez; individualID: DHJPAR0016623; individualCount: 1; sex: F; lifeStage: adult; preparations: pinned; otherCatalogNumbers: ASTAP827-07, 06-SRNP-44079; **Taxon:** scientificName: Telothyria erythropyga; phylum: Arthropoda; class: Insecta; order: Diptera; family: Tachinidae; genus: Telothyria; specificEpithet: erythropyga; scientificNameAuthorship: Fleming & Wood, 2018; **Location:** continent: Central America; country: Costa Rica; countryCode: CR; stateProvince: Alajuela; county: Sector Rincon Rain Forest; locality: Area de Conservacion Guanacaste; verbatimLocality: Conguera; verbatimElevation: 420; verbatimLatitude: 10.9159; verbatimLongitude: -85.2663; verbatimCoordinateSystem: Decimal; decimalLatitude: 10.9159; decimalLongitude: -85.2663; **Identification:** identifiedBy: AJ Fleming; dateIdentified: 2018; **Event:** samplingProtocol: Reared from the larva of the Crambidae, Desmia Solis19; verbatimEventDate: 25-Nov-2006; **Record Level:** language: en; institutionCode: CNC; collectionCode: Insects; basisOfRecord: Pinned Specimen
- **Type status:** Paratype. **Occurrence:** occurrenceDetails: http://janzen.sas.upenn.edu; catalogNumber: DHJPAR0018614; recordedBy: D.H. Janzen, W. Hallwachs & Jose Perez; individualID: DHJPAR0018614; individualCount: 1; sex: M; lifeStage: adult; preparations: pinned; otherCatalogNumbers: ASTAI1261-07, 01-SRNP-23343; **Taxon:** scientificName: Telothyria erythropyga; phylum: Arthropoda; class: Insecta; order: Diptera; family: Tachinidae; genus: Telothyria; specificEpithet: erythropyga; scientificNameAuthorship: Fleming & Wood, 2018; **Location:** continent: Central America; country: Costa Rica; countryCode: CR; stateProvince: Alajuela; county: Sector Rincon Rain Forest; locality: Area de Conservacion Guanacaste; verbatimLocality: Sendero Rincon; verbatimElevation: 430; verbatimLatitude: 10.8962; verbatimLongitude: -85.2777; verbatimCoordinateSystem: Decimal; decimalLatitude: 10.8962; decimalLongitude: -85.2777; **Identification:** identifiedBy: AJ Fleming; dateIdentified: 2018; **Event:** samplingProtocol: Reared from the larva of the Crambidae, Phostria Janzen05; verbatimEventDate: 05-Jan-2002; **Record Level:** language: en; institutionCode: CNC; collectionCode: Insects; basisOfRecord: Pinned Specimen
- **Type status:** Paratype. **Occurrence:** occurrenceDetails: http://janzen.sas.upenn.edu; catalogNumber: DHJPAR0034439; recordedBy: D.H. Janzen, W. Hallwachs & Anabelle Cordoba; individualID: DHJPAR0034439; individualCount: 1; sex: F; lifeStage: adult; preparations: pinned; otherCatalogNumbers: ASHYC1091-09, 09-SRNP-41069; **Taxon:** scientificName: Telothyria erythropyga; phylum: Arthropoda; class: Insecta; order: Diptera; family: Tachinidae; genus: Telothyria; specificEpithet: erythropyga; scientificNameAuthorship: Fleming & Wood, 2018; **Location:** continent: Central America; country: Costa Rica; countryCode: CR; stateProvince: Alajuela; county: Sector Rincon Rain Forest; locality: Area de Conservacion Guanacaste; verbatimLocality: San Lucas; verbatimElevation: 320; verbatimLatitude: 10.9185; verbatimLongitude: -85.3034; verbatimCoordinateSystem: Decimal; decimalLatitude: 10.9185; decimalLongitude: -85.3034; **Identification:** identifiedBy: AJ Fleming; dateIdentified: 2018; **Event:** samplingProtocol: Reared from the larva of the Crambidae, Phostria Janzen05; verbatimEventDate: 06-Sep-2009; **Record Level:** language: en; institutionCode: CNC; collectionCode: Insects; basisOfRecord: Pinned Specimen
- **Type status:** Paratype. **Occurrence:** occurrenceDetails: http://janzen.sas.upenn.edu; catalogNumber: DHJPAR0035742; recordedBy: D.H. Janzen, W. Hallwachs & Anabelle Cordoba; individualID: DHJPAR0035742; individualCount: 1; sex: F; lifeStage: adult; preparations: pinned; otherCatalogNumbers: ASHYD1123-09, 09-SRNP-41748; **Taxon:** scientificName: Telothyria erythropyga; phylum: Arthropoda; class: Insecta; order: Diptera; family: Tachinidae; genus: Telothyria; specificEpithet: erythropyga; scientificNameAuthorship: Fleming & Wood, 2018; **Location:** continent: Central America; country: Costa Rica; countryCode: CR; stateProvince: Alajuela; county: Sector Rincon Rain Forest; locality: Area de Conservacion Guanacaste; verbatimLocality: Sendero Tucan; verbatimElevation: 410; verbatimLatitude: 10.9042; verbatimLongitude: -85.2712; verbatimCoordinateSystem: Decimal; decimalLatitude: 10.9042; decimalLongitude: -85.2712; **Identification:** identifiedBy: AJ Fleming; dateIdentified: 2018; **Event:** samplingProtocol: Reared from the larva of the Crambidae, Phostria Janzen05; verbatimEventDate: 14-Aug-2009; **Record Level:** language: en; institutionCode: CNC; collectionCode: Insects; basisOfRecord: Pinned Specimen
- **Type status:** Paratype. **Occurrence:** occurrenceDetails: http://janzen.sas.upenn.edu; catalogNumber: DHJPAR0037318; recordedBy: D.H. Janzen, W. Hallwachs & Pablo Umana Calderon; individualID: DHJPAR0037318; individualCount: 1; sex: F; lifeStage: adult; preparations: pinned; otherCatalogNumbers: ASHYC4063-10, 09-SRNP-42882; **Taxon:** scientificName: Telothyria erythropyga; phylum: Arthropoda; class: Insecta; order: Diptera; family: Tachinidae; genus: Telothyria; specificEpithet: erythropyga; scientificNameAuthorship: Fleming & Wood, 2018; **Location:** continent: Central America; country: Costa Rica; countryCode: CR; stateProvince: Alajuela; county: Sector Rincon Rain Forest; locality: Area de Conservacion Guanacaste; verbatimLocality: Sendero Rincon; verbatimElevation: 430; verbatimLatitude: 10.8962; verbatimLongitude: -85.2777; verbatimCoordinateSystem: Decimal; decimalLatitude: 10.8962; decimalLongitude: -85.2777; **Identification:** identifiedBy: AJ Fleming; dateIdentified: 2018; **Event:** samplingProtocol: Reared from the larva of the Crambidae, Desmia Janzen07; verbatimEventDate: 10-Nov-2009; **Record Level:** language: en; institutionCode: CNC; collectionCode: Insects; basisOfRecord: Pinned Specimen
- **Type status:** Paratype. **Occurrence:** occurrenceDetails: http://janzen.sas.upenn.edu; catalogNumber: DHJPAR0039300; recordedBy: D.H. Janzen, W. Hallwachs & Elda Araya; individualID: DHJPAR0039300; individualCount: 1; sex: M; lifeStage: adult; preparations: pinned; otherCatalogNumbers: ASTAV863-10, 10-SRNP-2485; **Taxon:** scientificName: Telothyria erythropyga; phylum: Arthropoda; class: Insecta; order: Diptera; family: Tachinidae; genus: Telothyria; specificEpithet: erythropyga; scientificNameAuthorship: Fleming & Wood, 2018; **Location:** continent: Central America; country: Costa Rica; countryCode: CR; stateProvince: Alajuela; county: Sector San Cristobal; locality: Area de Conservacion Guanacaste; verbatimLocality: Tajo Angeles; verbatimElevation: 540; verbatimLatitude: 10.8647; verbatimLongitude: -85.4153; verbatimCoordinateSystem: Decimal; decimalLatitude: 10.8647; decimalLongitude: -85.4153; **Identification:** identifiedBy: AJ Fleming; dateIdentified: 2018; **Event:** samplingProtocol: Reared from the larva of the Crambidae, Desmia Solis19; verbatimEventDate: 10-Jun-2010; **Record Level:** language: en; institutionCode: CNC; collectionCode: Insects; basisOfRecord: Pinned Specimen
- **Type status:** Paratype. **Occurrence:** occurrenceDetails: http://janzen.sas.upenn.edu; catalogNumber: DHJPAR0045686; recordedBy: D.H. Janzen, W. Hallwachs & Anabelle Cordoba; individualID: DHJPAR0045686; individualCount: 1; sex: M; lifeStage: adult; preparations: pinned; otherCatalogNumbers: ACGAZ875-11, 11-SRNP-43494; **Taxon:** scientificName: Telothyria erythropyga; phylum: Arthropoda; class: Insecta; order: Diptera; family: Tachinidae; genus: Telothyria; specificEpithet: erythropyga; scientificNameAuthorship: Fleming & Wood, 2018; **Location:** continent: Central America; country: Costa Rica; countryCode: CR; stateProvince: Alajuela; county: Sector Rincon Rain Forest; locality: Area de Conservacion Guanacaste; verbatimLocality: Rio Francia Arriba; verbatimElevation: 400; verbatimLatitude: 10.8967; verbatimLongitude: -85.29; verbatimCoordinateSystem: Decimal; decimalLatitude: 10.8967; decimalLongitude: -85.29; **Identification:** identifiedBy: AJ Fleming; dateIdentified: 2018; **Event:** samplingProtocol: Reared from the larva of the Crambidae, Phostria Janzen05; verbatimEventDate: 28-Aug-2011; **Record Level:** language: en; institutionCode: CNC; collectionCode: Insects; basisOfRecord: Pinned Specimen
- **Type status:** Paratype. **Occurrence:** occurrenceDetails: http://janzen.sas.upenn.edu; catalogNumber: DHJPAR0051982; recordedBy: D.H. Janzen, W. Hallwachs & Ricardo Calero; individualID: DHJPAR0051982; individualCount: 1; sex: M; lifeStage: adult; preparations: pinned; otherCatalogNumbers: ASHYH1094-13, 13-SRNP-70434; **Taxon:** scientificName: Telothyria erythropyga; phylum: Arthropoda; class: Insecta; order: Diptera; family: Tachinidae; genus: Telothyria; specificEpithet: erythropyga; scientificNameAuthorship: Fleming & Wood, 2018; **Location:** continent: Central America; country: Costa Rica; countryCode: CR; stateProvince: Guanacaste; county: Sector Pitilla; locality: Area de Conservacion Guanacaste; verbatimLocality: Sendero Suspiro; verbatimElevation: 439; verbatimLatitude: 10.9839; verbatimLongitude: -85.3885; verbatimCoordinateSystem: Decimal; decimalLatitude: 10.9839; decimalLongitude: -85.3885; **Identification:** identifiedBy: AJ Fleming; dateIdentified: 2018; **Event:** samplingProtocol: Reared from the larva of the Crambidae, Phostria Janzen05; verbatimEventDate: 14-Apr-2013; **Record Level:** language: en; institutionCode: CNC; collectionCode: Insects; basisOfRecord: Pinned Specimen

#### Description

**Male.** Length: 5–9 mm (Fig. 22). **Head** (Fig. 22b): frons narrow, 1/8 of head width; gena 1/9 of head height; four reclinate orbital setae; anteriormost reclinate orbital almost equal to uppermost frontal seta; ocellar setae present but so small as to appear absent; outer vertical seta absent; fronto-orbital plate brassy-gold on uppermost 30%; ocellar triangle with pale gold tomentum on posterior half, with anterior margin concolorous with frontal vitta; fronto-orbital plate with short blonde hairs interspersed among frontal setae; parafacial brilliant silver; facial ridge bare; palpus apically slightly inflated to appear slightly oar-shaped, sparsely haired along outer margin; arista brown, almost bare, distinctly-thickened on basal 1/10, microtrichia at most equally as long as width of arista; postpedicel orange over at most 60% of surface; postocular region behind margin of eye upper half gold, with lower half including gena silver tomentose; upper half of occiput gold tomentose. **Thorax** (Fig. 22a, c): golden tomentose, with four distinct dorsal stripes; thorax entirely covered in dense plumose blonde hairs; chaetotaxy: 5–6 postpronotal setae, basal setae arranged in a straight line; supra-alar setae 2:3; intra-alar setae 1–2:2; dorsocentral setae 3:3; acrostichal setae 3:3; katepisternum with three setae. Scutellum golden tomentose; two pairs of strong marginal setae (basal and subapical) and a small pair of crossed apical scutellar setae 1/3rd as long as subapical scutellars; basal scutellar setae subequal in length to subapical setae; subapical setae straight; underside of scutellum bearing plumose blonde hairs below basal scutellar setae. **Legs**: foreleg with yellow ground color throughout, tibia and tarsal segments appearing darker due to hair covering; midleg, coxa and proximal half of femur yellow, remainder of femur, tibia and tarsal segments brown; hindleg dark brown extending from distal half of femur to tarsal segments; anterior leg tibia with regular fringe of equally spaced setae along anteroventral surface, and two strong posterodorsal seta. **Wings**: basicosta ivory white; all veins bare, with only 1–2 setulae at base of R_4+5_.; calypters pale white translucent with a narrow yellowish fringe. **Abdomen** (Fig. 22a, c): ground color yellow-orange; ST1+2 brown over medial 30%, with yellow ventrolaterally, extending into a longitudinal middorsal brown stripe up to posterior edge of T3 and T4; T3–T5 with dense gold tomentum along anterior marginal 10%, thinning and extending over remainder of tergite; T5 entirely orange with gold tomentum; median marginal setae present only on T4 and T5; median discal setae absent. **Male terminalia** (Fig. 22d, e, f): Sternite 5 with a wide deeply separated median cleft, widely V-shaped, margins tomentose; lateral lobes of sternite subtriangular apically, outer margins covered in strong setae; basal section of sternite 5 subequal in length to apical lobes. Cerci in posterior view sharply pointed rectangular, fused along entire length; medial shoulder weak to undeveloped, entire structure vaguely sword shaped. In lateral view cerci, with a mild downward curve, and several strong widely spaced setae along basal 2/3rds. Surstylus in lateral view rounded at tip, not downwardly curved, overall digitiform in appearance; fused with epandrium; when viewed dorsally surstyli appear straight with a very slight club or swelling apically. Distiphallus 2X as long as basiphallus and tubular, slightly pointed at apex.

**Female.** Length: 5–7 mm (Fig. 23). **Head** (Fig. 23b): as in male with the following exceptions: fronto-orbital plate 30% gold; ocellar triangle concolorous with remainder of vertex; parafacial brilliant silver; frons 1/4 of head width; four inner reclinate orbital setae; two proclinate orbital setae; outer vertical seta present; palpus significantly inflated and oar-shaped apically. **Thorax** (Fig. 23a, c): katepisternum with three setae; meron with a brush of plumose setae and 2–3 typical meral setae. Legs: colored as in male. **Abdomen** (Fig. 23a, c): ground color brown dorsally on ST1+2 and T3 with orange laterally; T4 entirely brown ground color; T5 as in male; marginal setae present on T4, T5 and sometimes on T3.

#### Diagnosis

*Telothyria erythropyga* **sp. n.** can be distinguished from all other *Telothyria* by the following combination of traits: ocellar setae present but so small as to appear absent, parafacial brilliant silver, postpedicel orange over at most 60% of surface, arista plumose microtrichia at most equally as long as width of arista, facial ridge bare, thorax entirely covered in dense plumose blonde hairs, and T5 orange with gold tomentum.

#### Etymology

*Telothyria erythropyga* **sp. n.** From the Greek adjective “*erythros*” meaning red and the Greek noun "*pygo*" meaning rump or tail, in reference to the apically orange T5.

#### Distribution

Costa Rica, ACG, Alajuela and Guanacaste Provinces, 320–540 m elevation.

#### Ecology

*Telothyria erythropyga* **sp. n.** has been reared 33 times from four species of Lepidoptera in the family Crambidae: *Phostria* Janzen05, *Phostria* Janzen03, *Desmia* Solis19, *Desmia* Janzen576, *Desmia* Janzen07 in rain forest and dry-rain lowland intergrade.

### Telothyria fimbriata

Fleming & Wood sp. n.

03B827B1-0249-5B89-96CF-FA80C6B1F00B

urn:lsid:zoobank.org:act:14551CCF-920F-4BD1-9048-09346B608B25

#### Materials

- **Type status:** Holotype. **Occurrence:** occurrenceDetails: http://janzen.sas.upenn.edu; catalogNumber: DHJPAR0046710; recordedBy: D.H. Janzen, W. Hallwachs & Cirilo Umana; individualID: DHJPAR0046710; individualCount: 1; sex: M; lifeStage: adult; preparations: pinned; otherCatalogNumbers: ACGBA883-12, 11-SRNP-76463, BOLD:AAA1948; **Taxon:** scientificName: Telothyria fimbriata; phylum: Arthropoda; class: Insecta; order: Diptera; family: Tachinidae; genus: Telothyria; specificEpithet: fimbriata; scientificNameAuthorship: Fleming & Wood, 2018; **Location:** continent: Central America; country: Costa Rica; countryCode: CR; stateProvince: Alajuela; county: Sector Rincon Rain Forest; locality: Area de Conservacion Guanacaste; verbatimLocality: Finca Esmeralda; verbatimElevation: 123; verbatimLatitude: 10.9355; verbatimLongitude: -85.2531; verbatimCoordinateSystem: Decimal; decimalLatitude: 10.9355; decimalLongitude: -85.2531; **Identification:** identifiedBy: AJ Fleming; dateIdentified: 2018; **Event:** samplingProtocol: Reared from the larva of the Tortricidae, Phricanthes flexilineana; verbatimEventDate: 19-Nov-2011; **Record Level:** language: en; institutionCode: CNC; collectionCode: Insects; basisOfRecord: Pinned Specimen
- **Type status:** Paratype. **Occurrence:** occurrenceDetails: http://janzen.sas.upenn.edu; catalogNumber: DHJPAR0035697; recordedBy: D.H. Janzen, W. Hallwachs & Cirilo Umana; individualID: DHJPAR0035697; individualCount: 1; sex: F; lifeStage: adult; preparations: pinned; otherCatalogNumbers: ASHYD1078-09, 09-SRNP-44699, BOLD:AAA1948; **Taxon:** scientificName: Telothyria fimbriata; phylum: Arthropoda; class: Insecta; order: Diptera; family: Tachinidae; genus: Telothyria; specificEpithet: fimbriata; scientificNameAuthorship: Fleming & Wood, 2018; **Location:** continent: Central America; country: Costa Rica; countryCode: CR; stateProvince: Alajuela; county: Sector Rincon Rain Forest; locality: Area de Conservacion Guanacaste; verbatimLocality: Estacion Llanura; verbatimElevation: 135; verbatimLatitude: 10.9333; verbatimLongitude: -85.2533; verbatimCoordinateSystem: Decimal; decimalLatitude: 10.9333; decimalLongitude: -85.2533; **Identification:** identifiedBy: AJ Fleming; dateIdentified: 2018; **Event:** samplingProtocol: Reared from the larva of the Tortricidae, Phricanthes flexilineana; verbatimEventDate: 12-Jul-2009; **Record Level:** language: en; institutionCode: CNC; collectionCode: Insects; basisOfRecord: Pinned Specimen
- **Type status:** Paratype. **Occurrence:** occurrenceDetails: http://janzen.sas.upenn.edu; catalogNumber: DHJPAR0036676; recordedBy: D.H. Janzen, W. Hallwachs & Cirilo Umana; individualID: DHJPAR0036676; individualCount: 1; sex: F; lifeStage: adult; preparations: pinned; otherCatalogNumbers: ASHYE1587-09, 09-SRNP-75787, BOLD:AAA1948; **Taxon:** scientificName: Telothyria fimbriata; phylum: Arthropoda; class: Insecta; order: Diptera; family: Tachinidae; genus: Telothyria; specificEpithet: fimbriata; scientificNameAuthorship: Fleming & Wood, 2018; **Location:** continent: Central America; country: Costa Rica; countryCode: CR; stateProvince: Alajuela; county: Sector Rincon Rain Forest; locality: Area de Conservacion Guanacaste; verbatimLocality: Finca Esmeralda; verbatimElevation: 123; verbatimLatitude: 10.9355; verbatimLongitude: -85.2531; verbatimCoordinateSystem: Decimal; decimalLatitude: 10.9355; decimalLongitude: -85.2531; **Identification:** identifiedBy: AJ Fleming; dateIdentified: 2018; **Event:** samplingProtocol: Reared from the larva of the Tortricidae, Phricanthes flexilineana; verbatimEventDate: 06-Oct-2009; **Record Level:** language: en; institutionCode: CNC; collectionCode: Insects; basisOfRecord: Pinned Specimen
- **Type status:** Paratype. **Occurrence:** occurrenceDetails: http://janzen.sas.upenn.edu; catalogNumber: DHJPAR0037320; recordedBy: D.H. Janzen, W. Hallwachs & Mercedes Moraga; individualID: DHJPAR0037320; individualCount: 1; sex: M; lifeStage: adult; preparations: pinned; otherCatalogNumbers: ASHYC4065-10, 09-SRNP-76169, BOLD:AAA1948; **Taxon:** scientificName: Telothyria fimbriata; phylum: Arthropoda; class: Insecta; order: Diptera; family: Tachinidae; genus: Telothyria; specificEpithet: fimbriata; scientificNameAuthorship: Fleming & Wood, 2018; **Location:** continent: Central America; country: Costa Rica; countryCode: CR; stateProvince: Alajuela; county: Sector Rincon Rain Forest; locality: Area de Conservacion Guanacaste; verbatimLocality: Estacion Llanura; verbatimElevation: 135; verbatimLatitude: 10.9333; verbatimLongitude: -85.2533; verbatimCoordinateSystem: Decimal; decimalLatitude: 10.9333; decimalLongitude: -85.2533; **Identification:** identifiedBy: AJ Fleming; dateIdentified: 2018; **Event:** samplingProtocol: Reared from the larva of the Tortricidae, Phricanthes flexilineana; verbatimEventDate: 11-Oct-2009; **Record Level:** language: en; institutionCode: CNC; collectionCode: Insects; basisOfRecord: Pinned Specimen
- **Type status:** Paratype. **Occurrence:** occurrenceDetails: http://janzen.sas.upenn.edu; catalogNumber: DHJPAR0037334; recordedBy: D.H. Janzen, W. Hallwachs & Cirilo Umana; individualID: DHJPAR0037334; individualCount: 1; sex: F; lifeStage: adult; preparations: pinned; otherCatalogNumbers: ASHYC4079-10, 09-SRNP-76194, BOLD:AAA1948; **Taxon:** scientificName: Telothyria fimbriata; phylum: Arthropoda; class: Insecta; order: Diptera; family: Tachinidae; genus: Telothyria; specificEpithet: fimbriata; scientificNameAuthorship: Fleming & Wood, 2018; **Location:** continent: Central America; country: Costa Rica; countryCode: CR; stateProvince: Alajuela; county: Sector Rincon Rain Forest; locality: Area de Conservacion Guanacaste; verbatimLocality: Estacion Llanura; verbatimElevation: 135; verbatimLatitude: 10.9333; verbatimLongitude: -85.2533; verbatimCoordinateSystem: Decimal; decimalLatitude: 10.9333; decimalLongitude: -85.2533; **Identification:** identifiedBy: AJ Fleming; dateIdentified: 2018; **Event:** samplingProtocol: Reared from the larva of the Tortricidae, Phricanthes flexilineana; verbatimEventDate: 17-Nov-2009; **Record Level:** language: en; institutionCode: CNC; collectionCode: Insects; basisOfRecord: Pinned Specimen
- **Type status:** Paratype. **Occurrence:** occurrenceDetails: http://janzen.sas.upenn.edu; catalogNumber: DHJPAR0045651; recordedBy: D.H. Janzen, W. Hallwachs & Cirilo Umana; individualID: DHJPAR0045651; individualCount: 1; sex: M; lifeStage: adult; preparations: pinned; otherCatalogNumbers: ACGAZ840-11, 11-SRNP-75740, BOLD:AAA1948; **Taxon:** scientificName: Telothyria fimbriata; phylum: Arthropoda; class: Insecta; order: Diptera; family: Tachinidae; genus: Telothyria; specificEpithet: fimbriata; scientificNameAuthorship: Fleming & Wood, 2018; **Location:** continent: Central America; country: Costa Rica; countryCode: CR; stateProvince: Alajuela; county: Sector Rincon Rain Forest; locality: Area de Conservacion Guanacaste; verbatimLocality: Finca Esmeralda; verbatimElevation: 123; verbatimLatitude: 10.9355; verbatimLongitude: -85.2531; verbatimCoordinateSystem: Decimal; decimalLatitude: 10.9355; decimalLongitude: -85.2531; **Identification:** identifiedBy: AJ Fleming; dateIdentified: 2018; **Event:** samplingProtocol: Reared from the larva of the Tortricidae, Phricanthes flexilineana; verbatimEventDate: 20-Sep-2011; **Record Level:** language: en; institutionCode: CNC; collectionCode: Insects; basisOfRecord: Pinned Specimen
- **Type status:** Paratype. **Occurrence:** occurrenceDetails: http://janzen.sas.upenn.edu; catalogNumber: DHJPAR0034416; recordedBy: D.H. Janzen, W. Hallwachs & Cirilo Umana; individualID: DHJPAR0034416; individualCount: 1; sex: M; lifeStage: adult; preparations: pinned; otherCatalogNumbers: ASHYC1068-09, 09-SRNP-44473, BOLD:AAA1948; **Taxon:** scientificName: Telothyria fimbriata; phylum: Arthropoda; class: Insecta; order: Diptera; family: Tachinidae; genus: Telothyria; specificEpithet: fimbriata; scientificNameAuthorship: Fleming & Wood, 2018; **Location:** continent: Central America; country: Costa Rica; countryCode: CR; stateProvince: Alajuela; county: Sector Rincon Rain Forest; locality: Area de Conservacion Guanacaste; verbatimLocality: Estacion Llanura; verbatimElevation: 135; verbatimLatitude: 10.9333; verbatimLongitude: -85.2533; verbatimCoordinateSystem: Decimal; decimalLatitude: 10.9333; decimalLongitude: -85.2533; **Identification:** identifiedBy: AJ Fleming; dateIdentified: 2018; **Event:** samplingProtocol: Reared from the larva of the Tortricidae, Phricanthes flexilineana; verbatimEventDate: 24-Jun-2009; **Record Level:** language: en; institutionCode: CNC; collectionCode: Insects; basisOfRecord: Pinned Specimen
- **Type status:** Paratype. **Occurrence:** occurrenceDetails: http://janzen.sas.upenn.edu; catalogNumber: DHJPAR0057225; recordedBy: D.H. Janzen, W. Hallwachs & Mercedes Moraga; individualID: DHJPAR0057225; individualCount: 1; sex: M; lifeStage: adult; preparations: pinned; otherCatalogNumbers: ACGBA5135-15, 14-SRNP-77549, BOLD:AAA1948; **Taxon:** scientificName: Telothyria fimbriata; phylum: Arthropoda; class: Insecta; order: Diptera; family: Tachinidae; genus: Telothyria; specificEpithet: fimbriata; scientificNameAuthorship: Fleming & Wood, 2018; **Location:** continent: Central America; country: Costa Rica; countryCode: CR; stateProvince: Alajuela; county: Sector Rincon Rain Forest; locality: Area de Conservacion Guanacaste; verbatimLocality: Finca Esmeralda; verbatimElevation: 123; verbatimLatitude: 10.9355; verbatimLongitude: -85.2531; verbatimCoordinateSystem: Decimal; decimalLatitude: 10.9355; decimalLongitude: -85.2531; **Identification:** identifiedBy: AJ Fleming; dateIdentified: 2018; **Event:** samplingProtocol: Reared from the larva of the Tortricidae, Phricanthes flexilineana; verbatimEventDate: 27-Dec-2014; **Record Level:** language: en; institutionCode: CNC; collectionCode: Insects; basisOfRecord: Pinned Specimen

#### Description

**Figure 25.**
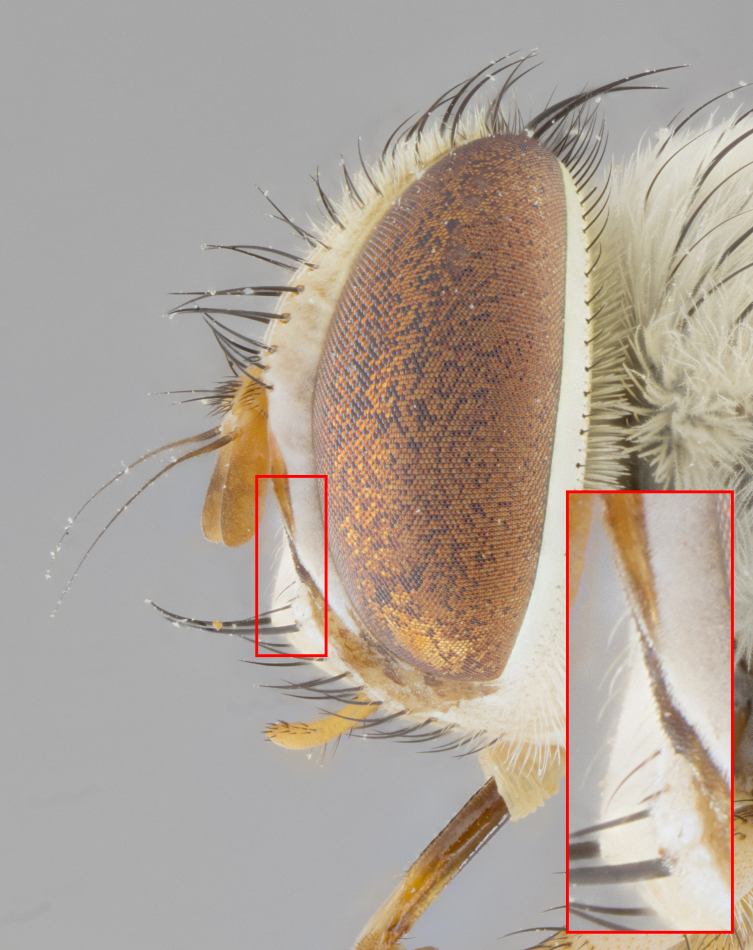
Detailed lateral view of holotype of *T. fimbriata* **sp. n.** male head, inset displaying characteristic pale haired facial ridge

**Male.** Length: 6–8 mm (Fig. 24). **Head** (Figs 24b, 25): frons narrow, 1/8 of head width; gena 1/10 of head height; three reclinate orbital setae, anteriormost reclinate orbital shorter than uppermost frontal seta; ocellar setae present but minimal; outer vertical seta small yet present; fronto-orbital plate and ocellar triangle coloration gold; fronto-orbital plate with short blonde hairs interspersed among frontal setae; parafacial brilliant silver; facial ridge bearing minuscule blonde hairs at least along lower 1/2 (Fig. 25); palpus digitiform, sparsely haired along outer margin; arista brown, smoothly tapering to apical 1/8, microtrichia at most 1X as long as width of arista; postpedicel almost entirely orange; postocular region behind margin of eye upper half gold, with lower half including gena silver tomentose; upper half of occiput gold tomentose. **Thorax** (Fig. 24a, c): brassy-gold tomentose, with two distinct outer dorsal stripes, and two short inner stripes; thorax entirely covered in dense plumose blonde hairs; chaetotaxy: five postpronotal setae, basal setae arranged in a straight line; supra-alar setae 1:3; intra-alar setae 2:2; dorsocentral setae 3:3; acrostichal setae 3:3; katepisternum with two setae. Scutellum brassy-gold tomentose; two pairs of strong marginal setae (basal and subapical) and a small pair of crossed apical scutellar setae 1/8–1/10th as long as subapical scutellars; basal scutellar setae subequal in length to subapical setae; subapical setae straight; underside of scutellum bearing plumose blonde hairs below basal scutellar setae. **Legs**: foreleg yellow ground color, densely haired on tarsal segments with short black hairs making them appear darkened almost black; midleg with yellow coxa and femur and brown tibia and tarsal segments; hindleg dark brown extending from halfway along femur inclusive of tarsal segments; anterior leg tibia with regular fringe of equally spaced setae along anteroventral surface, with 2–3 anterodorsal setae, and 2–3 posterodorsal setae. **Wings**: basicosta ivory white; all veins bare, with only 1–2 setulae at base of R_4+5_; calypters pale white translucent with narrow pale beige fringe. **Abdomen** (Fig. 24a, c): ground color yellow-orange; ST1+2 brown over medial 30%, with yellow ventrolaterally, extending into a longitudinal middorsal brown stripe bisected by a brown band along posterior edges of T3 and T4; T3 and T4 each with dense gold tomentum along extending over entire tergite; T5 entirely yellow, covered with gold tomentum; median marginal setae present only on T4 and T5; median discal setae absent. **Male terminalia** (Fig. 24d, e, f): Sternite 5 with a wide deeply excavated median cleft, smoothly V-shaped, margins covered in dense pollinosity; lateral lobes of sternite rounded apically, with a small group of strong setulae along outer margins; basal section of sternite 5 subequal to slightly longer than length of apical lobes. Cerci in posterior view sharply pointed triangular sharply widening to a moderate rectangular shoulder along the basal section, equal in length to surstyli, fused along entire length; in lateral view, with a strong downward curve on apical 1/3; several strong widely spaced setulae along basal 2/3rds. Surstylus in lateral view, almost equilateral along its length rounded at tip, slightly pinched at midpoint appearing digitiform; surstylus appearing fused with epandrium; when viewed dorsally surstyli appear slender with a slight sinusoidal curve, parallel at apices. Distiphallus 1.5X as long as basiphallus and tubular, slightly pointed at apex.

**Female.** Length: 6–8 mm (Fig. 26). **Head** (Fig. 26b): as in male with the following exceptions: fronto-orbital plate pale brassy–gold over upper 30%; parafacial brilliant silver; frons 1/4 of head width; three inner reclinate orbital setae; three orbital setae two proclinate, and uppermost reclinate; outer vertical a small group of strong present; palpus apically clubbed and distinctly upturned. **Thorax** (Fig. 26a, c): katepisternum with two setae; meron with plumose hairs as well as 4–6 typical meral setae. Legs: colored as in male with the following exception: anterior tibia with regular fringe of equally spaced setae along anteroventral surface, and 1–2 posterodorsal setae. **Abdomen** (Fig. 26a, c): ST1+2 and T3 50% brown dorsally, with yellow-orange lateroventrally, T4 entirely brown, and T5 yellow-orange entirely.

#### Diagnosis

*Telothyria fimbriata* **sp. n.** can be distinguished from all other *Telothyria* by the following combination of traits: ocellar setae present but minimal, parafacial brilliant silver, postpedicel almost entirely orange, arista plumose on lower half with microtrichia not exceeding the width of the arista, fine yellow hairs extending along at least lower half of facial ridge, thorax entirely covered in dense plumose blonde hairs, and T5 yellow with gold tomentum.

#### Etymology

*Telothyria fimbriata* **sp. n.** From the Latin adjective “*fimbriatus*” meaning fringed in reference to the pale blonde hairs that line the facial ridge.

#### Distribution

Costa Rica, ACG, Alajuela Province, 123–135 m elevation.

#### Ecology

*Telothyria fimbriata* **sp. n.** has been reared eight times from a single species of Lepidoptera in the family Tortricidae: *Phricanthes flexilineana* (Walker, 1863), in rain forest.

### Telothyria fulgida

Fleming & Wood sp. n.

A8E006FE-7BC8-5FC1-B57D-071E5DFCC5B9

urn:lsid:zoobank.org:act:9BB929B6-8EF5-4299-8610-C931A567D8BB

#### Materials

- **Type status:** Holotype. **Occurrence:** catalogNumber: CNC618904; recordedBy: D.M. Wood; individualID: CNC618904; individualCount: 1; sex: M; lifeStage: adult; preparations: pinned; **Taxon:** scientificName: Telothyria fulgida; phylum: Arthropoda; class: Insecta; order: Diptera; family: Tachinidae; genus: Telothyria; specificEpithet: fulgida; scientificNameAuthorship: Fleming & Wood, 2019; **Location:** continent: Central America; country: Costa Rica; countryCode: CR; stateProvince: Puntarenas; verbatimLocality: Monteverde; verbatimElevation: 1500; **Identification:** identifiedBy: AJ Fleming; dateIdentified: 2019; **Event:** samplingProtocol: Hand collected; verbatimEventDate: 10-12-Dec-1990; **Record Level:** language: en; institutionCode: CNC; collectionCode: Insects; basisOfRecord: Pinned Specimen
- **Type status:** Paratype. **Occurrence:** catalogNumber: CNC618891; recordedBy: D.M. Wood; individualID: CNC618891; individualCount: 1; sex: M; lifeStage: adult; preparations: pinned; **Taxon:** scientificName: Telothyria fulgida; phylum: Arthropoda; class: Insecta; order: Diptera; family: Tachinidae; genus: Telothyria; specificEpithet: fulgida; scientificNameAuthorship: Fleming & Wood, 2019; **Location:** continent: Central America; country: Costa Rica; countryCode: CR; stateProvince: Puntarenas; verbatimLocality: Monteverde; verbatimElevation: 1500; **Identification:** identifiedBy: AJ Fleming; dateIdentified: 2019; **Event:** samplingProtocol: Hand collected; verbatimEventDate: 10-12-Dec-1990; **Record Level:** language: en; institutionCode: CNC; collectionCode: Insects; basisOfRecord: Pinned Specimen
- **Type status:** Paratype. **Occurrence:** recordedBy: D.M. Wood; individualCount: 1; sex: M; lifeStage: adult; preparations: pinned; **Taxon:** scientificName: Telothyria fulgida; phylum: Arthropoda; class: Insecta; order: Diptera; family: Tachinidae; genus: Telothyria; specificEpithet: fulgida; scientificNameAuthorship: Fleming & Wood, 2019; **Location:** continent: Central America; country: Costa Rica; countryCode: CR; stateProvince: Puntarenas; verbatimLocality: Monteverde; verbatimElevation: 1500; **Identification:** identifiedBy: AJ Fleming; dateIdentified: 2019; **Event:** samplingProtocol: Hand collected; verbatimEventDate: 10-12-Dec-1990; **Record Level:** language: en; institutionCode: CNC; collectionCode: Insects; basisOfRecord: Pinned Specimen
- **Type status:** Paratype. **Occurrence:** recordedBy: D.M. Wood; individualCount: 1; sex: M; lifeStage: adult; preparations: pinned; **Taxon:** scientificName: Telothyria fulgida; phylum: Arthropoda; class: Insecta; order: Diptera; family: Tachinidae; genus: Telothyria; specificEpithet: fulgida; scientificNameAuthorship: Fleming & Wood, 2019; **Location:** continent: Central America; country: Costa Rica; countryCode: CR; stateProvince: Puntarenas; verbatimLocality: Monteverde; verbatimElevation: 1500; **Identification:** identifiedBy: AJ Fleming; dateIdentified: 2019; **Event:** samplingProtocol: Hand collected; verbatimEventDate: 10-12-Dec-1990; **Record Level:** language: en; institutionCode: CNC; collectionCode: Insects; basisOfRecord: Pinned Specimen
- **Type status:** Paratype. **Occurrence:** recordedBy: D.M. Wood; individualCount: 1; sex: M; lifeStage: adult; preparations: pinned; **Taxon:** scientificName: Telothyria fulgida; phylum: Arthropoda; class: Insecta; order: Diptera; family: Tachinidae; genus: Telothyria; specificEpithet: fulgida; scientificNameAuthorship: Fleming & Wood, 2019; **Location:** continent: Central America; country: Costa Rica; countryCode: CR; stateProvince: Puntarenas; verbatimLocality: Monteverde; verbatimElevation: 1500; **Identification:** identifiedBy: AJ Fleming; dateIdentified: 2019; **Event:** samplingProtocol: Hand collected; verbatimEventDate: 20-22-Jul-1993; **Record Level:** language: en; institutionCode: CNC; collectionCode: Insects; basisOfRecord: Pinned Specimen
- **Type status:** Paratype. **Occurrence:** recordedBy: D.M. Wood; individualCount: 1; sex: M; lifeStage: adult; preparations: pinned; **Taxon:** scientificName: Telothyria fulgida; phylum: Arthropoda; class: Insecta; order: Diptera; family: Tachinidae; genus: Telothyria; specificEpithet: fulgida; scientificNameAuthorship: Fleming & Wood, 2019; **Location:** continent: Central America; country: Mexico; countryCode: MX; stateProvince: Chiapas; verbatimLocality: 6 km SW of Ocosingo; verbatimElevation: 1400; **Identification:** identifiedBy: AJ Fleming; dateIdentified: 2019; **Event:** samplingProtocol: Hand collected; verbatimEventDate: 20-Sep-1991; **Record Level:** language: en; institutionCode: CNC; collectionCode: Insects; basisOfRecord: Pinned Specimen
- **Type status:** Paratype. **Occurrence:** recordedBy: D.M. Wood; individualCount: 1; sex: M; lifeStage: adult; preparations: pinned; **Taxon:** scientificName: Telothyria fulgida; phylum: Arthropoda; class: Insecta; order: Diptera; family: Tachinidae; genus: Telothyria; specificEpithet: fulgida; scientificNameAuthorship: Fleming & Wood, 2019; **Location:** continent: Central America; country: Mexico; countryCode: MX; stateProvince: Chiapas; verbatimLocality: 6 km SW of Ocosingo; verbatimElevation: 1400; **Identification:** identifiedBy: AJ Fleming; dateIdentified: 2019; **Event:** samplingProtocol: Hand collected; verbatimEventDate: 20-Sep-1991; **Record Level:** language: en; institutionCode: CNC; collectionCode: Insects; basisOfRecord: Pinned Specimen
- **Type status:** Paratype. **Occurrence:** recordedBy: D.M. Wood; individualCount: 1; sex: M; lifeStage: adult; preparations: pinned; **Taxon:** scientificName: Telothyria fulgida; phylum: Arthropoda; class: Insecta; order: Diptera; family: Tachinidae; genus: Telothyria; specificEpithet: fulgida; scientificNameAuthorship: Fleming & Wood, 2019; **Location:** continent: Central America; country: Mexico; countryCode: MX; stateProvince: Chiapas; verbatimLocality: 6 km SW of Ocosingo; verbatimElevation: 1400; **Identification:** identifiedBy: AJ Fleming; dateIdentified: 2019; **Event:** samplingProtocol: Hand collected; verbatimEventDate: 22-Sep-1992; **Record Level:** language: en; institutionCode: CNC; collectionCode: Insects; basisOfRecord: Pinned Specimen
- **Type status:** Paratype. **Occurrence:** recordedBy: D.M. Wood; individualCount: 1; sex: M; lifeStage: adult; preparations: pinned; **Taxon:** scientificName: Telothyria fulgida; phylum: Arthropoda; class: Insecta; order: Diptera; family: Tachinidae; genus: Telothyria; specificEpithet: fulgida; scientificNameAuthorship: Fleming & Wood, 2019; **Location:** continent: Central America; country: Mexico; countryCode: MX; stateProvince: Chiapas; verbatimLocality: 6 km SW of Ocosingo; verbatimElevation: 1400; **Identification:** identifiedBy: AJ Fleming; dateIdentified: 2019; **Event:** samplingProtocol: Hand collected; verbatimEventDate: 22-Sep-1992; **Record Level:** language: en; institutionCode: CNC; collectionCode: Insects; basisOfRecord: Pinned Specimen

#### Description

**Male.** Length: 7–9 mm (Fig. 27). **Head** (Fig. 27b): frons wide almost 1/3 of head width; gena 1/10 of head height; two reclinate orbital setae; anteriormost reclinate orbital almost equal to uppermost frontal seta; ocellar setae reduced but present, arising behind anterior ocellus; outer vertical seta absent; fronto-orbital plate enlarged, frontal vitta almost absent coloration brilliant silver throughout, ocellar triangle silver and contiguous with fronto-orbital plate; fronto-orbital plate densely covered with short pale blonde hairs interspersed among frontal setae; parafacial pale brilliant silver; facial ridge bare; palpus short yellow and digitiform with slight upward turn apically, sparsely haired along outer margin; arista brown, smoothly tapered, microtrichia at most 2X as long as width of arista; pedicel orange, postpedicel with slight orange apex, adjacent to pedicel; postocular region behind margin of eye gold tomentose, gena mostly silver tomentose; upper half of occiput gold tomentose. **Thorax** (Fig. 27a, c): dark brownish ground color with light gold tomentum, dorsal stripes indistinct and not evident; thorax densely covered in plumose blonde hairs throughout; chaetotaxy: 4–5 postpronotal setae, basal setae arranged in a straight line; supra-alar setae 2:3; intra-alar setae 3:3; dorsocentral setae 4:4; acrostichal setae 3:3; katepisternum with three setae. Scutellum brown with slight gold tomentum; two pairs of strong marginal setae (basal and subapical) and a small pair of crossed apical scutellar setae 1/5th as long as subapical scutellars; basal scutellar setae subequal in length to subapical setae; subapical setae straight; underside of scutellum bearing plumose blonde hairs below basal scutellar setae. **Legs**: foreleg with dark brown-orange femur and yellow tibia appearing darkened due to hair covering, with dark yellow-brown ground color tarsal segments; mid leg and hind leg similar to foreleg; hind femur with covering of blonde hairs proximal on proximal half of femur; anterior leg tibia with regular fringe of equally spaced setae along anteroventral surface, with one posterodorsal seta. **Wings**: basicosta orange; all veins bare, and very slightly infuscate, with one setula at base of R_4+5_.; calypters strongly cinereous infuscate with a narrow yellowish fringe. **Abdomen** (Fig. 27a, c): ground color dark brown dorsally, T1+2–T4 with yellow ventrolaterally and T5 brown with orange apically; T3–T5 with dense gold tomentum along margin, extending and thinning over entire tergite appearing to have a gold sheen when viewed with the naked eye; median marginal setae present only on T4 and T5; median discal setae absent. **Terminalia** (Fig. 27d, e, f): Sternite 5 with a wide deeply separated median cleft, widely V-shaped, margins tomentose; lateral lobes of sternite subtriangular apically, outer margins covered in strong setae; basal section of sternite 5 2X longer than length of apical lobes. Cerci in posterior view sharply pointed rectangular with a widened shoulder medially, equal in length to surstyli, fused along entire length; medial shoulder weakly developed, entire structure vaguely dagger-shaped. In lateral view cerci, with a strong downward curve, with several strong widely spaced setae along basal 2/3rds. Surstylus in lateral view pointed at tip, downwardly curved overall digitiform in appearance; fused with epandrium; when viewed dorsally surstyli appear robust and straight with a very slight club apically, strongly hirsute along entire length. Basiphallus short and stout and stout, distiphallus subequal to in length to basiphallus, weakly tapering apically.

**Female.** Unknown at this time.

#### Diagnosis

*Telothyria fulgida* **sp. n.** is easily distinguished from all other *Telothyria* by the following combination of traits: ocellar setae reduced but present, frons wide almost 1/3 of head width, prominent and brilliant silver, with frontal vitta almost obliterated; parafacial entirely silver; pedicel orange with postpedicel mostly dark, thorax entirely covered in plumose blonde hairs, dorsal stripes indistinct and not evident; abdominal ground color dark brown, with yellow ventrolaterally from ST1+2–T4. Differentiated from *T. frontalis* Townsend by the tibia of fore leg having only one posterodorsal seta, and an anteroventral fringe, and the abdominal ground color being dark brown dorsally with yellow laterally, T5 orange only apically.

#### Etymology

*Telothyria fulgida* **sp. n.** From the Latin adjective, “*fulgidus*” meaning shining, in reference to its remarkable shining silver frons.

#### Distribution

Southern Mexico south to Costa Rica, 1400–1500 m.

#### Ecology

Specimens hand collected, 15 times from altitudes 1400–1500 m, further ecology not available.

### Telothyria gloriasihezarae

Fleming & Wood sp. n.

72F186F7-E82A-52D9-B711-D3AF9C16D4EB

urn:lsid:zoobank.org:act:087E9E50-124E-4DCA-AEC2-6C257C47F0A1

#### Materials

- **Type status:** Holotype. **Occurrence:** occurrenceDetails: http://janzen.sas.upenn.edu; catalogNumber: DHJPAR0046451; recordedBy: D.H. Janzen, W. Hallwachs & Gloria Sihezar; individualID: DHJPAR0046451; individualCount: 1; sex: F; lifeStage: adult; preparations: pinned; otherCatalogNumbers: ACGBA624-12, 11-SRNP-5113, BOLD:ABX0074; **Taxon:** scientificName: Telothyria gloriasihezarae; phylum: Arthropoda; class: Insecta; order: Diptera; family: Tachinidae; genus: Telothyria; specificEpithet: gloriasihezarae; scientificNameAuthorship: Fleming & Wood, 2018; **Location:** continent: Central America; country: Costa Rica; countryCode: CR; stateProvince: Alajuela; county: Sector San Cristobal; locality: Area de Conservacion Guanacaste; verbatimLocality: Estacion San Gerardo; verbatimElevation: 575; verbatimLatitude: 10.8801; verbatimLongitude: -85.3889; verbatimCoordinateSystem: Decimal; decimalLatitude: 10.8801; decimalLongitude: -85.3889; **Identification:** identifiedBy: AJ Fleming; dateIdentified: 2018; **Event:** samplingProtocol: Reared from the larva of the Crambidae, Desmia Janzen03; verbatimEventDate: 15-Jan-2012; **Record Level:** language: en; institutionCode: CNC; collectionCode: Insects; basisOfRecord: Pinned Specimen

#### Description

**Female.** Length: 5 mm (Fig. 28). **Head** (Fig. 28a, b): frons 1/4 of head width; gena 1/7 of head height; three reclinate inner orbital setae uppermost reclinate orbital pair slightly convergent, and two proclinate orbital setae; ocellar setae absent; outer vertical seta present; fronto-orbital plate pale brassy-gold; fronto-orbital plate with short blonde hairs interspersed among frontal setae; parafacial brilliant silver; facial ridge bare; palpus apically clubbed and slightly upturned; arista brown, smoothly tapering to apical 1/8, microtrichia at most 1.5X as long as width of arista; postpedicel orange over at most 30% of surface; postocular region behind margin of eye including gena gold tomentose; occiput gold tomentose. **Thorax** (Fig. 28a, c): brassy-gold tomentose, with four distinct thoracic stripes outer pair broken across suture; plumose hairs absent from disc of scutum; chaetotaxy: 5–6 postpronotal setae, basal setae arranged in a straight line; supra-alar setae 2:3; intra-alar setae 2:3; dorsocentral setae 3:3; acrostichal setae 3:3; katepisternum with three setae; meron lacking plumose hairs with 9–12 typical meral setae. Scutellum brassy-gold tomentose; two pairs of strong marginal setae (basal and subapical) and a small pair of crossed apical scutellar setae 1/8–1/10th as long as subapical scutellars; basal scutellar setae subequal in length to subapical setae; subapical setae straight; underside of scutellum bearing plumose blonde hairs below basal scutellar setae. **Legs**: foreleg brown coxa and proximal half of femur, yellow with ground color extending from distal half of femur, tarsal segments darkened by hair covering; both midleg and hindleg with yellow coxa, and remainder of both legs yellow-brown appearing dark brown due to hair covering; anterior leg tibia with irregular sized fringe of equally spaced setae along anteroventral surface, with one posterodorsal setae. **Wings**: basicosta ivory white; all veins bare, with only one setula at base of R_4+5_; calypters pale white translucent. **Abdomen** (Fig. 28a, c): ground color yellow-orange; ST1+2 brown over medial 50%, with yellow ventrolaterally, extending into a longitudinal middorsal brown stripe bisected by a brown band along posterior edge of T3 and T4 entirely brown dorsally; T1+2–T4 with dense gold tomentum extending over entire tergite; T5 entirely yellow, covered with gold tomentum; marginal setae present on T4 and T5; median discal setae absent.

**Male.** Unknown at this time.

#### Diagnosis

*Telothyria gloriasihezarae* **sp. n.** can be distinguished from all other *Telothyria* by the following combination of traits: ocellar setae absent, plumose hairs absent from disc of scutum, katepisternum with three setae, three postsutural intra-alar setae, and T5 yellow with gold tomentum.

#### Etymology

*Telothyria gloriasihezarae* **sp. n.** is named in recognition of Gloria Sihezar's outstanding work on the team that conducts the caterpillar and parasite inventory from ACG’s Estación Biológica San Gerardo.

#### Distribution

Costa Rica, ACG, Alajuela Province, 575 m elevation.

#### Ecology

*Telothyria gloriasihezarae* **sp. n.** has been reared two times from a single species of Lepidoptera in the family Crambidae: *Desmia Janzen03*, in rain forest.

### Telothyria grisea

Fleming & Wood sp. n.

6CAAA128-203F-530C-A17A-AC9DCD670457

urn:lsid:zoobank.org:act:FC166EC0-C6A1-4271-9EC4-F4BF7265DBF8

#### Materials

- **Type status:** Holotype. **Occurrence:** occurrenceDetails: http://janzen.sas.upenn.edu; catalogNumber: DHJPAR0052019; recordedBy: D.H. Janzen, W. Hallwachs & Gloria Sihezar; individualID: DHJPAR0052019; individualCount: 1; sex: M; lifeStage: adult; preparations: pinned; otherCatalogNumbers: ASHYH1131-13, 13-SRNP-1775, BOLD:AAM9452; **Taxon:** scientificName: Telothyria grisea; phylum: Arthropoda; class: Insecta; order: Diptera; family: Tachinidae; genus: Telothyria; specificEpithet: grisea; scientificNameAuthorship: Fleming & Wood, 2018; **Location:** continent: Central America; country: Costa Rica; countryCode: CR; stateProvince: Alajuela; county: Sector San Cristobal; locality: Area de Conservacion Guanacaste; verbatimLocality: Sendero Perdido; verbatimElevation: 620; verbatimLatitude: 10.8794; verbatimLongitude: -85.3861; verbatimCoordinateSystem: Decimal; decimalLatitude: 10.8794; decimalLongitude: -85.3861; **Identification:** identifiedBy: AJ Fleming; dateIdentified: 2018; **Event:** samplingProtocol: Reared from the larva of the Crambidae, Rhectocraspeda periusalis; verbatimEventDate: 05-May-2013; **Record Level:** language: en; institutionCode: CNC; collectionCode: Insects; basisOfRecord: Pinned Specimen
- **Type status:** Paratype. **Occurrence:** occurrenceDetails: http://janzen.sas.upenn.edu; catalogNumber: DHJPAR0048413; recordedBy: D.H. Janzen, W. Hallwachs & Gloria Sihezar; individualID: DHJPAR0048413; individualCount: 1; sex: F; lifeStage: adult; preparations: pinned; otherCatalogNumbers: ACGBA1955-12, 12-SRNP-718, BOLD:AAM9452; **Taxon:** scientificName: Telothyria grisea; phylum: Arthropoda; class: Insecta; order: Diptera; family: Tachinidae; genus: Telothyria; specificEpithet: grisea; scientificNameAuthorship: Fleming & Wood, 2018; **Location:** continent: Central America; country: Costa Rica; countryCode: CR; stateProvince: Alajuela; county: Sector San Cristobal; locality: Area de Conservacion Guanacaste; verbatimLocality: Tajo Angeles; verbatimElevation: 540; verbatimLatitude: 10.8647; verbatimLongitude: -85.4153; verbatimCoordinateSystem: Decimal; decimalLatitude: 10.8647; decimalLongitude: -85.4153; **Identification:** identifiedBy: AJ Fleming; dateIdentified: 2018; **Event:** samplingProtocol: Reared from the larva of the Crambidae, Rhectocraspeda periusalis; verbatimEventDate: 21-Mar-2012; **Record Level:** language: en; institutionCode: CNC; collectionCode: Insects; basisOfRecord: Pinned Specimen
- **Type status:** Paratype. **Occurrence:** occurrenceDetails: http://janzen.sas.upenn.edu; catalogNumber: DHJPAR0039254; recordedBy: D.H. Janzen, W. Hallwachs & Gloria Sihezar; individualID: DHJPAR0039254; individualCount: 1; sex: F; lifeStage: adult; preparations: pinned; otherCatalogNumbers: ASTAV817-10, 10-SRNP-1610, BOLD:AAM9452; **Taxon:** scientificName: Telothyria grisea; phylum: Arthropoda; class: Insecta; order: Diptera; family: Tachinidae; genus: Telothyria; specificEpithet: grisea; scientificNameAuthorship: Fleming & Wood, 2018; **Location:** continent: Central America; country: Costa Rica; countryCode: CR; stateProvince: Alajuela; county: Sector Rincon Rain Forest; locality: Area de Conservacion Guanacaste; verbatimLocality: Sendero Albergue Crater; verbatimElevation: 980; verbatimLatitude: 10.8489; verbatimLongitude: -85.3281; verbatimCoordinateSystem: Decimal; decimalLatitude: 10.8489; decimalLongitude: -85.3281; **Identification:** identifiedBy: AJ Fleming; dateIdentified: 2018; **Event:** samplingProtocol: Reared from the larva of the Crambidae, Rhectocraspeda periusalis; verbatimEventDate: 16-Apr-2010; **Record Level:** language: en; institutionCode: CNC; collectionCode: Insects; basisOfRecord: Pinned Specimen
- **Type status:** Paratype. **Occurrence:** occurrenceDetails: http://janzen.sas.upenn.edu; catalogNumber: DHJPAR0038741; recordedBy: D.H. Janzen, W. Hallwachs & Gloria Sihezar; individualID: DHJPAR0038741; individualCount: 1; sex: F; lifeStage: adult; preparations: pinned; otherCatalogNumbers: 10-SRNP-858; **Taxon:** scientificName: Telothyria grisea; phylum: Arthropoda; class: Insecta; order: Diptera; family: Tachinidae; genus: Telothyria; specificEpithet: grisea; scientificNameAuthorship: Fleming & Wood, 2018; **Location:** continent: Central America; country: Costa Rica; countryCode: CR; stateProvince: Alajuela; county: Sector Rincon Rain Forest; locality: Area de Conservacion Guanacaste; verbatimLocality: Sendero Albergue Oscar; verbatimElevation: 560; verbatimLatitude: 10.87741; verbatimLongitude: -85.32363; verbatimCoordinateSystem: Decimal; decimalLatitude: 10.87741; decimalLongitude: -85.32363; **Identification:** identifiedBy: AJ Fleming; dateIdentified: 2018; **Event:** samplingProtocol: Reared from the larva of the Crambidae, Rhectocraspeda periusalis; verbatimEventDate: 09-Mar-2010; **Record Level:** language: en; institutionCode: CNC; collectionCode: Insects; basisOfRecord: Pinned Specimen
- **Type status:** Paratype. **Occurrence:** occurrenceDetails: http://janzen.sas.upenn.edu; catalogNumber: DHJPAR0038732; recordedBy: D.H. Janzen, W. Hallwachs & Elda Araya; individualID: DHJPAR0038732; individualCount: 1; sex: F; lifeStage: adult; preparations: pinned; otherCatalogNumbers: 10-SRNP-1092; **Taxon:** scientificName: Telothyria grisea; phylum: Arthropoda; class: Insecta; order: Diptera; family: Tachinidae; genus: Telothyria; specificEpithet: grisea; scientificNameAuthorship: Fleming & Wood, 2018; **Location:** continent: Central America; country: Costa Rica; countryCode: CR; stateProvince: Alajuela; county: Sector Rincon Rain Forest; locality: Area de Conservacion Guanacaste; verbatimLocality: Sendero Albergue Oscar; verbatimElevation: 561; verbatimLatitude: 10.87741; verbatimLongitude: -85.32363; verbatimCoordinateSystem: Decimal; decimalLatitude: 10.87741; decimalLongitude: -85.32363; **Identification:** identifiedBy: AJ Fleming; dateIdentified: 2018; **Event:** samplingProtocol: Reared from the larva of the Crambidae, Rhectocraspeda periusalis; verbatimEventDate: 29-Mar-2011; **Record Level:** language: en; institutionCode: CNC; collectionCode: Insects; basisOfRecord: Pinned Specimen
- **Type status:** Paratype. **Occurrence:** occurrenceDetails: http://janzen.sas.upenn.edu; catalogNumber: DHJPAR0054089; recordedBy: D.H. Janzen, W. Hallwachs & Gloria Sihezar; individualID: DHJPAR0054089; individualCount: 1; sex: F; lifeStage: adult; preparations: pinned; otherCatalogNumbers: 13-SRNP-5783; **Taxon:** scientificName: Telothyria grisea; phylum: Arthropoda; class: Insecta; order: Diptera; family: Tachinidae; genus: Telothyria; specificEpithet: grisea; scientificNameAuthorship: Fleming & Wood, 2018; **Location:** continent: Central America; country: Costa Rica; countryCode: CR; stateProvince: Alajuela; county: Sector San Cristobal; locality: Area de Conservacion Guanacaste; verbatimLocality: Jardin Estrada; verbatimElevation: 722; verbatimLatitude: 10.86546; verbatimLongitude: -85.39694; verbatimCoordinateSystem: Decimal; decimalLatitude: 10.86546; decimalLongitude: -85.39694; **Identification:** identifiedBy: AJ Fleming; dateIdentified: 2018; **Event:** samplingProtocol: Reared from the larva of the Crambidae, Rhectocraspeda periusalis; verbatimEventDate: 19-Nov-2013; **Record Level:** language: en; institutionCode: CNC; collectionCode: Insects; basisOfRecord: Pinned Specimen

#### Description

**Male** (Fig. 29), length: 5–9 mm. **Head** (Fig. 29b): frons narrow, 1/6 of head width; gena 1/9 of head height; three reclinate orbital setae; anteriormost reclinate orbital shorter than uppermost frontal seta; ocellar setae absent; outer vertical seta present; ocellar triangle slightly darkened, overall appearing concolorous with fronto-orbital plate; fronto-orbital plate gold, with short blonde hairs on lower portion transitioning to black hairs on upper half interspersed among frontal setae; parafacial brilliant silver; facial ridge bare; palpus clubbed, sparsely haired along outer margin; arista brown, plumose, smoothly tapering to apical 1/8, microtrichia at most 3X as long as width of arista; postpedicel orange over at most 30% of surface; postocular region behind margin of eye including gena silver tomentose; occiput silver tomentose. **Thorax** (Fig. 29a, c): gray tomentose, with two diffuse outer dorsal stripes, inner pair 1/4 width of outer pair postsuturally, only reaching up to 1st postsutural dorsocentral seta; thorax covered in dense plumose blonde hairs laterally, absent dorsally; chaetotaxy: five postpronotal setae, basal setae arranged in a straight line; supra-alar setae 1:3; intra-alar setae 1:2; dorsocentral setae 3:3; acrostichal setae 3:3; katepisternum with two setae; two pairs of strong marginal setae (basal and subapical) and a small pair of apical scutellar setae (orientation of apicals and subapicals unknown as these were broken off on single male specimen); scutellum gray tomentose; underside of scutellum bearing regular non-plumose black hairs below basal scutellar setae. **Legs**: anterior femur dark overall, yellow posteroventrally, remainder of legs entirely dark orange-brown, becoming bright orange on coxae, and all joints; anterior leg tibia with regular fringe of equally spaced setae along anteroventral surface, with two posterodorsal seta; midleg femur with three strong anteroventral setae. **Wings**: basicosta brown; all veins bare, with only 1–2 setula at base of R_4+5_; calypters pale translucent. **Abdomen** (Fig. 29a, c): ground color yellow-orange; ST1+2 brown over medial 30%, with yellow ventrolaterally, a longitudinal middorsal brown stripe up to T5, bisected by a brown band along posterior edges of T3 and T4; T3 and T4 each with light gold tomentum along anterior margin, occupying about 10%, thinning and extending over remainder of tergite; T5 entirely dark brown; median marginal setae present only on T4 and T5; median discal setae absent. **Male terminalia**: not examined.

**Female.** Length: 5–7 mm (Fig. 30). **Head** (Fig. 30b): as in male with the following exceptions: fronto-orbital plate 70% gray, with pale brassy color only along apical portion and ocellar triangle; parafacial brilliant silver; frons 1/4 of head width; three inner reclinate orbital setae; three orbital setae, two proclinate, and uppermost reclinate; outer vertical seta present. **Thorax** (Fig. 30a, c): three postsutural intra-alar setae; katepisternum with three setae; meron lacking plumose hairs only 9–12 typical meral setae. **Legs**: ground color as in male, but with bright orange color from joints extending up to 1/2 of leg; anterior leg tibia with irregular fringe of widely spaced setae along anteroventral surface, and single posterodorsal seta. **Abdomen** (Fig. 30a, c): colored as in male except for strong row of marginal setae on T4.

#### Diagnosis

*Telothyria grisea* **sp. n.** can be distinguished from all other *Telothyria* by the following combination of traits: thorax dark ground color, gray tomentose dorsally, plumose hairs absent from disc of scutum; four distinct dorsal stripes, outer pair diffues and 4X as wide as inner pair, with inner pair only reaching up to 1st postsutural dorsocentral seta, katepisternum with two setae.

#### Etymology

*Telothyria grisea* **sp. n.** From the Latin adjective “*griseus*” meaning grey in reference to the coloration of the thoracic tomentum.

#### Distribution

Costa Rica, ACG, Alajuela Province, 560–980 m elevation.

#### Ecology

*Telothyria grisea* **sp. n.** has been reared six times from a single species of Lepidoptera in the family Crambidae: *Rhectocraspeda periusalis* (Walker, 1859), in rain forest.

### Telothyria harryramirezi

Fleming & Wood sp. n.

18EA8112-E7F7-5B1E-84C4-DA9ADB9B527C

urn:lsid:zoobank.org:act:D6C9AD9F-2547-4571-9F8F-191E736735B3

#### Materials

- **Type status:** Holotype. **Occurrence:** occurrenceDetails: http://janzen.sas.upenn.edu; catalogNumber: DHJPAR0050683; recordedBy: D.H. Janzen, W. Hallwachs & Anabelle Cordoba; individualID: DHJPAR0050683; individualCount: 1; sex: M; lifeStage: adult; preparations: pinned; otherCatalogNumbers: ACGBA3275-13, 12-SRNP-86821, BOLD:ACJ2339; **Taxon:** scientificName: Telothyria harryramirezi; phylum: Arthropoda; class: Insecta; order: Diptera; family: Tachinidae; genus: Telothyria; specificEpithet: harryramirezi; scientificNameAuthorship: Fleming & Wood, 2018; **Location:** continent: Central America; country: Costa Rica; countryCode: CR; stateProvince: Alajuela; county: Sector Rincon Rain Forest; locality: Area de Conservacion Guanacaste; verbatimLocality: Quebrada Guarumo; verbatimElevation: 400; verbatimLatitude: 10.9045; verbatimLongitude: -85.2841; verbatimCoordinateSystem: Decimal; decimalLatitude: 10.9045; decimalLongitude: -85.2841; **Identification:** identifiedBy: AJ Fleming; dateIdentified: 2018; **Event:** samplingProtocol: Reared from the larva of the Crambidae, Ategumia Solis01; verbatimEventDate: 13-Dec-2012; **Record Level:** language: en; institutionCode: CNC; collectionCode: Insects; basisOfRecord: Pinned Specimen
- **Type status:** Paratype. **Occurrence:** occurrenceDetails: http://janzen.sas.upenn.edu; catalogNumber: DHJPAR0050706; recordedBy: D.H. Janzen, W. Hallwachs & Anabelle Cordoba; individualID: DHJPAR0050706; individualCount: 1; sex: M; lifeStage: adult; preparations: pinned; otherCatalogNumbers: ACGBA3298-13, 12-SRNP-86823, BOLD:ACJ2339; **Taxon:** scientificName: Telothyria harryramirezi; phylum: Arthropoda; class: Insecta; order: Diptera; family: Tachinidae; genus: Telothyria; specificEpithet: harryramirezi; scientificNameAuthorship: Fleming & Wood, 2018; **Location:** continent: Central America; country: Costa Rica; countryCode: CR; stateProvince: Alajuela; county: Sector Rincon Rain Forest; locality: Area de Conservacion Guanacaste; verbatimLocality: Quebrada Guarumo; verbatimElevation: 400; verbatimLatitude: 10.9045; verbatimLongitude: -85.2841; verbatimCoordinateSystem: Decimal; decimalLatitude: 10.9045; decimalLongitude: -85.2841; **Identification:** identifiedBy: AJ Fleming; dateIdentified: 2018; **Event:** samplingProtocol: Reared from the larva of the Crambidae, Ategumia Solis01; verbatimEventDate: 13-Dec-2012; **Record Level:** language: en; institutionCode: CNC; collectionCode: Insects; basisOfRecord: Pinned Specimen

#### Description

**Male.** Length: 7–8 mm (Fig. 31). **Head** (Fig. 31b): frons narrow, 1/5 of head width; gena 1/12 of head height; 4–5 reclinate orbital setae uppermost reclinate orbital pair slightly convergent; anteriormost reclinate orbital subequal in length to uppermost frontal seta; ocellar setae absent; outer vertical seta absent; ocellar triangle and fronto-orbital plate dark gold; fronto-orbital plate with short blonde hairs interspersed among frontal setae; parafacial gold; facial ridge bare; palpus digitiform, apically terminating in a small bulbous club; arista brown, smoothly tapering to apical 1/8, microtrichia at most 1.5X as long as width of arista; postpedicel orange over inner surface and 60% of outer surface; postocular region behind margin of eye including gena gold tomentose; upper half of occiput gold tomentose. **Thorax** (Fig. 31a, c): brassy-gold tomentose, with two distinct outer dorsal stripes broken across suture, and two short inner stripes extending up to first postsutural dorsocentral seta; thorax covered in dense plumose blonde hairs laterally, dorsally plumose hairs mixed in with short black hairs; chaetotaxy: five postpronotal setae, basal setae arranged in a straight line; supra-alar setae 2:3; intra-alar setae 2:3; dorsocentral setae 3:3; acrostichal setae 3:3; katepisternum with three setae. Scutellum brassy-gold tomentose; two pairs of strong marginal setae (basal and subapical) and a small pair of crossed apical scutellar setae 1/8–1/10th as long as subapical scutellars; basal scutellar setae subequal in length to subapical setae; subapical setae straight; underside of scutellum bearing plumose blonde hairs below basal scutellar setae. **Legs**: foreleg ground color yellow on coxa and femur, appearing darker from tibia to tarsi; both midleg and hindleg dark brown entirely, with yellow coxae; anterior leg tibia with regular tapered fringe of equally spaced setae along basal half of anteroventral surface, and one strong posterodorsal seta. **Wings**: basicosta orange-beige; all veins bare, with only one setula at base of R_4+5_.; calypters pale white translucent with pale yellow fringe. **Abdomen** (Fig. 31a, c): ground color appearing brown-black dorsally with yellow-orange ventrolaterally; ST1+2 brown over medial 50%, with yellow ventrolaterally, extending into a longitudinal middorsal brown stripe bisected by a brown band along posterior edges of T3 and T4; T1+2–T4 with dense brassy tomentum extending over entire tergite; T5 entirely yellow, covered with gold tomentum; marginal setae present on T4 1/2 as long as those present on and T5; median discal setae absent. **Male terminalia** (Fig. 31d, e, f): Sternite 5 with a wide deeply excavated median cleft, smoothly V-shaped, margins covered in dense pollinosity; lateral lobes of sternite pointed apically, with a small group of strong setulae along outer margins; basal section of sternite 5 subequal to slightly shorter than length of apical lobes. Cerci in posterior view sharply pointed triangular sharply widening to a moderate almost rectangular shoulder along the basal section, equal in length to surstyli, fused along entire length; in lateral view, with a smooth regular downward curve along apical 2/3rds; several strong widely spaced setulae along basal 2/3rds. Surstylus in lateral view, almost equilateral along its length rounded at tip, digitiform; surstylus appearing fused with epandrium; when viewed dorsally surstyli appear slender with an inward bend. Distiphallus 3X as long as basiphallus and tubular, slightly pointed at apex.

**Female.** Unknown at this time.

#### Diagnosis

*Telothyria harryramirezi* **sp. n.** can be distinguished from all other *Telothyria* by the following combination of traits: ocellar setae absent, postpedicel orange over inner surface and 60% of outer surface, parafacial gold, thorax covered in dense plumose blonde hairs laterally, dorsally plumose hairs mixed in with short black hairs, katepisternum with three setae, legs yellow, abdominal ground color yellow-orange, and T5 yellow with gold tomentum.

#### Etymology

*Telothyria harryramirezi* **sp. n.** is named in recognition of Harry Ramirez's outstanding work on the team that conducts the caterpillar and parasite inventory from ACG’s Estación Biológica Cacao.

#### Distribution

Costa Rica, ACG, Alajuela Province, 400 m elevation.

#### Ecology

*Telothyria harryramirezi* **sp. n.** has been reared two times from a single species of Lepidoptera in the family Crambidae: *Ategumia* Solis01, in rain forest.

### Telothyria incisa

Fleming & Wood sp. n.

51372465-79FF-5ACF-ADFB-23671314A2BA

urn:lsid:zoobank.org:act:C73C9B30-EA2E-4ABE-9F88-84A6105535AB

#### Materials

- **Type status:** Holotype. **Occurrence:** occurrenceDetails: http://janzen.sas.upenn.edu; catalogNumber: DHJPAR0037586; recordedBy: D.H. Janzen, W. Hallwachs & Gloria Sihezar; individualID: DHJPAR0037586; individualCount: 1; sex: M; lifeStage: adult; preparations: pinned; otherCatalogNumbers: ASHYC4331-10, 09-SRNP-6845, BOLD:AAF0519; **Taxon:** scientificName: Telothyria incisa; phylum: Arthropoda; class: Insecta; order: Diptera; family: Tachinidae; genus: Telothyria; specificEpithet: incisa; scientificNameAuthorship: Fleming & Wood, 2018; **Location:** continent: Central America; country: Costa Rica; countryCode: CR; stateProvince: Alajuela; county: Sector San Cristobal; locality: Area de Conservacion Guanacaste; verbatimLocality: Puente Palma; verbatimElevation: 460; verbatimLatitude: 10.9163; verbatimLongitude: -85.3787; verbatimCoordinateSystem: Decimal; decimalLatitude: 10.9163; decimalLongitude: -85.3787; **Identification:** identifiedBy: AJ Fleming; dateIdentified: 2018; **Event:** samplingProtocol: Reared from the larva of the Crambidae, Herpetogramma Janzen07; verbatimEventDate: 19-Jan-2010; **Record Level:** language: en; institutionCode: CNC; collectionCode: Insects; basisOfRecord: Pinned Specimen
- **Type status:** Paratype. **Occurrence:** occurrenceDetails: http://janzen.sas.upenn.edu; catalogNumber: DHJPAR0037581; recordedBy: D.H. Janzen, W. Hallwachs & Gloria Sihezar; individualID: DHJPAR0037581; individualCount: 1; sex: M; lifeStage: adult; preparations: pinned; otherCatalogNumbers: ASHYC4326-10, 09-SRNP-6833, BOLD:AAF0519; **Taxon:** scientificName: Telothyria incisa; phylum: Arthropoda; class: Insecta; order: Diptera; family: Tachinidae; genus: Telothyria; specificEpithet: incisa; scientificNameAuthorship: Fleming & Wood, 2018; **Location:** continent: Central America; country: Costa Rica; countryCode: CR; stateProvince: Alajuela; county: Sector San Cristobal; locality: Area de Conservacion Guanacaste; verbatimLocality: Puente Palma; verbatimElevation: 460; verbatimLatitude: 10.9163; verbatimLongitude: -85.3787; verbatimCoordinateSystem: Decimal; decimalLatitude: 10.9163; decimalLongitude: -85.3787; **Identification:** identifiedBy: AJ Fleming; dateIdentified: 2018; **Event:** samplingProtocol: Reared from the larva of the Crambidae, Herpetogramma Janzen07; verbatimEventDate: 23-Jan-2010; **Record Level:** language: en; institutionCode: CNC; collectionCode: Insects; basisOfRecord: Pinned Specimen
- **Type status:** Paratype. **Occurrence:** occurrenceDetails: http://janzen.sas.upenn.edu; catalogNumber: DHJPAR0042684; recordedBy: D.H. Janzen, W. Hallwachs & Gloria Sihezar; individualID: DHJPAR0042684; individualCount: 1; sex: M; lifeStage: adult; preparations: pinned; otherCatalogNumbers: ASHYH442-11, 10-SRNP-7152, BOLD:AAF0519; **Taxon:** scientificName: Telothyria incisa; phylum: Arthropoda; class: Insecta; order: Diptera; family: Tachinidae; genus: Telothyria; specificEpithet: incisa; scientificNameAuthorship: Fleming & Wood, 2018; **Location:** continent: Central America; country: Costa Rica; countryCode: CR; stateProvince: Alajuela; county: Sector San Cristobal; locality: Area de Conservacion Guanacaste; verbatimLocality: Sendero Huerta; verbatimElevation: 527; verbatimLatitude: 10.9305; verbatimLongitude: -85.3722; verbatimCoordinateSystem: Decimal; decimalLatitude: 10.9305; decimalLongitude: -85.3722; **Identification:** identifiedBy: AJ Fleming; dateIdentified: 2018; **Event:** samplingProtocol: Reared from the larva of the Crambidae, Herpetogramma Janzen07; verbatimEventDate: 09-Jan-2011; **Record Level:** language: en; institutionCode: CNC; collectionCode: Insects; basisOfRecord: Pinned Specimen
- **Type status:** Paratype. **Occurrence:** occurrenceDetails: http://janzen.sas.upenn.edu; catalogNumber: DHJPAR0050292; recordedBy: D.H. Janzen, W. Hallwachs & Osvaldo Espinoza; individualID: DHJPAR0050292; individualCount: 1; sex: M; lifeStage: adult; preparations: pinned; otherCatalogNumbers: ACGAZ1606-12, 12-SRNP-3747, BOLD:AAF0519; **Taxon:** scientificName: Telothyria incisa; phylum: Arthropoda; class: Insecta; order: Diptera; family: Tachinidae; genus: Telothyria; specificEpithet: incisa; scientificNameAuthorship: Fleming & Wood, 2018; **Location:** continent: Central America; country: Costa Rica; countryCode: CR; stateProvince: Alajuela; county: Sector San Cristobal; locality: Area de Conservacion Guanacaste; verbatimLocality: Sendero Huerta; verbatimElevation: 527; verbatimLatitude: 10.9305; verbatimLongitude: -85.3722; verbatimCoordinateSystem: Decimal; decimalLatitude: 10.9305; decimalLongitude: -85.3722; **Identification:** identifiedBy: AJ Fleming; dateIdentified: 2018; **Event:** samplingProtocol: Reared from the larva of the Crambidae, Herpetogramma Janzen07; verbatimEventDate: 24-Sep-2012; **Record Level:** language: en; institutionCode: CNC; collectionCode: Insects; basisOfRecord: Pinned Specimen
- **Type status:** Paratype. **Occurrence:** occurrenceDetails: http://janzen.sas.upenn.edu; catalogNumber: DHJPAR0010333; recordedBy: D.H. Janzen, W. Hallwachs & Minor Carmona; individualID: DHJPAR0010333; individualCount: 1; sex: M; lifeStage: adult; preparations: pinned; otherCatalogNumbers: ASTAS164-06, 06-SRNP-42497, BOLD:AAF0519; **Taxon:** scientificName: Telothyria incisa; phylum: Arthropoda; class: Insecta; order: Diptera; family: Tachinidae; genus: Telothyria; specificEpithet: incisa; scientificNameAuthorship: Fleming & Wood, 2018; **Location:** continent: Central America; country: Costa Rica; countryCode: CR; stateProvince: Alajuela; county: Sector Rincon Rain Forest; locality: Area de Conservacion Guanacaste; verbatimLocality: Conguera; verbatimElevation: 420; verbatimLatitude: 10.9159; verbatimLongitude: -85.2663; verbatimCoordinateSystem: Decimal; decimalLatitude: 10.9159; decimalLongitude: -85.2663; **Identification:** identifiedBy: AJ Fleming; dateIdentified: 2018; **Event:** samplingProtocol: Reared from the larva of the Crambidae, Herpetogramma Janzen07; verbatimEventDate: 12-Aug-2006; **Record Level:** language: en; institutionCode: CNC; collectionCode: Insects; basisOfRecord: Pinned Specimen
- **Type status:** Paratype. **Occurrence:** occurrenceDetails: http://janzen.sas.upenn.edu; catalogNumber: DHJPAR0037282; recordedBy: D.H. Janzen, W. Hallwachs & Ricardo Calero; individualID: DHJPAR0037282; individualCount: 1; sex: F; lifeStage: adult; preparations: pinned; otherCatalogNumbers: ASHYC4027-10, 09-SRNP-73496, BOLD:AAF0519; **Taxon:** scientificName: Telothyria incisa; phylum: Arthropoda; class: Insecta; order: Diptera; family: Tachinidae; genus: Telothyria; specificEpithet: incisa; scientificNameAuthorship: Fleming & Wood, 2018; **Location:** continent: Central America; country: Costa Rica; countryCode: CR; stateProvince: Guanacaste; county: Sector Pitilla; locality: Area de Conservacion Guanacaste; verbatimLocality: Leonel; verbatimElevation: 510; verbatimLatitude: 10.9964; verbatimLongitude: -85.4019; verbatimCoordinateSystem: Decimal; decimalLatitude: 10.9964; decimalLongitude: -85.4019; **Identification:** identifiedBy: AJ Fleming; dateIdentified: 2018; **Event:** samplingProtocol: Reared from the larva of the Crambidae, Herpetogramma Janzen07; verbatimEventDate: 04-Dec-2009; **Record Level:** language: en; institutionCode: CNC; collectionCode: Insects; basisOfRecord: Pinned Specimen
- **Type status:** Paratype. **Occurrence:** occurrenceDetails: http://janzen.sas.upenn.edu; catalogNumber: DHJPAR0018612; recordedBy: D.H. Janzen, W. Hallwachs & Freyci Vargas; individualID: DHJPAR0018612; individualCount: 1; sex: F; lifeStage: adult; preparations: pinned; otherCatalogNumbers: ASTAI1259-07, 01-SRNP-5031, BOLD:AAF0519; **Taxon:** scientificName: Telothyria incisa; phylum: Arthropoda; class: Insecta; order: Diptera; family: Tachinidae; genus: Telothyria; specificEpithet: incisa; scientificNameAuthorship: Fleming & Wood, 2018; **Location:** continent: Central America; country: Costa Rica; countryCode: CR; stateProvince: Alajuela; county: Sector Rincon Rain Forest; locality: Area de Conservacion Guanacaste; verbatimLocality: Estacion Caribe; verbatimElevation: 415; verbatimLatitude: 10.9019; verbatimLongitude: -85.2749; verbatimCoordinateSystem: Decimal; decimalLatitude: 10.9019; decimalLongitude: -85.2749; **Identification:** identifiedBy: AJ Fleming; dateIdentified: 2018; **Event:** samplingProtocol: Reared from the larva of the Crambidae, Herpetogramma Janzen07; verbatimEventDate: 16-Jun-2001; **Record Level:** language: en; institutionCode: CNC; collectionCode: Insects; basisOfRecord: Pinned Specimen
- **Type status:** Paratype. **Occurrence:** occurrenceDetails: http://janzen.sas.upenn.edu; catalogNumber: DHJPAR0036576; recordedBy: D.H. Janzen, W. Hallwachs & Pablo Umana Calderon; individualID: DHJPAR0036576; individualCount: 1; sex: F; lifeStage: adult; preparations: pinned; otherCatalogNumbers: ASHYE1487-09, 09-SRNP-42172, BOLD:AAF0519; **Taxon:** scientificName: Telothyria incisa; phylum: Arthropoda; class: Insecta; order: Diptera; family: Tachinidae; genus: Telothyria; specificEpithet: incisa; scientificNameAuthorship: Fleming & Wood, 2018; **Location:** continent: Central America; country: Costa Rica; countryCode: CR; stateProvince: Alajuela; county: Sector Rincon Rain Forest; locality: Area de Conservacion Guanacaste; verbatimLocality: San Lucas; verbatimElevation: 320; verbatimLatitude: 10.9185; verbatimLongitude: -85.3034; verbatimCoordinateSystem: Decimal; decimalLatitude: 10.9185; decimalLongitude: -85.3034; **Identification:** identifiedBy: AJ Fleming; dateIdentified: 2018; **Event:** samplingProtocol: Reared from the larva of the Crambidae, Herpetogramma Janzen07; verbatimEventDate: 03-Sep-2009; **Record Level:** language: en; institutionCode: CNC; collectionCode: Insects; basisOfRecord: Pinned Specimen
- **Type status:** Paratype. **Occurrence:** occurrenceDetails: http://janzen.sas.upenn.edu; catalogNumber: DHJPAR0034442; recordedBy: D.H. Janzen, W. Hallwachs & Geovanny Lobo; individualID: DHJPAR0034442; individualCount: 1; sex: M; lifeStage: adult; preparations: pinned; otherCatalogNumbers: ASHYC1094-09, 09-SRNP-41000, BOLD:AAF0519; **Taxon:** scientificName: Telothyria incisa; phylum: Arthropoda; class: Insecta; order: Diptera; family: Tachinidae; genus: Telothyria; specificEpithet: incisa; scientificNameAuthorship: Fleming & Wood, 2018; **Location:** continent: Central America; country: Costa Rica; countryCode: CR; stateProvince: Alajuela; county: Sector Rincon Rain Forest; locality: Area de Conservacion Guanacaste; verbatimLocality: Estacion Caribe; verbatimElevation: 415; verbatimLatitude: 10.9019; verbatimLongitude: -85.2749; verbatimCoordinateSystem: Decimal; decimalLatitude: 10.9019; decimalLongitude: -85.2749; **Identification:** identifiedBy: AJ Fleming; dateIdentified: 2018; **Event:** samplingProtocol: Reared from the larva of the Crambidae, Herpetogramma Janzen07; verbatimEventDate: 06-Aug-2009; **Record Level:** language: en; institutionCode: CNC; collectionCode: Insects; basisOfRecord: Pinned Specimen
- **Type status:** Paratype. **Occurrence:** occurrenceDetails: http://janzen.sas.upenn.edu; catalogNumber: DHJPAR0037591; recordedBy: D.H. Janzen, W. Hallwachs & Gloria Sihezar; individualID: DHJPAR0037591; individualCount: 1; sex: F; lifeStage: adult; preparations: pinned; otherCatalogNumbers: ASHYC4336-10, 09-SRNP-6850, BOLD:AAF0519; **Taxon:** scientificName: Telothyria incisa; phylum: Arthropoda; class: Insecta; order: Diptera; family: Tachinidae; genus: Telothyria; specificEpithet: incisa; scientificNameAuthorship: Fleming & Wood, 2018; **Location:** continent: Central America; country: Costa Rica; countryCode: CR; stateProvince: Alajuela; county: Sector San Cristobal; locality: Area de Conservacion Guanacaste; verbatimLocality: Puente Palma; verbatimElevation: 460; verbatimLatitude: 10.9163; verbatimLongitude: -85.3787; verbatimCoordinateSystem: Decimal; decimalLatitude: 10.9163; decimalLongitude: -85.3787; **Identification:** identifiedBy: AJ Fleming; dateIdentified: 2018; **Event:** samplingProtocol: Reared from the larva of the Crambidae, Herpetogramma Janzen07; verbatimEventDate: 21-Jan-2010; **Record Level:** language: en; institutionCode: CNC; collectionCode: Insects; basisOfRecord: Pinned Specimen

#### Description

**Male.** Length: 6–9 mm (Fig. 32). **Head** (Fig. 32b): frons narrow, 1/5–1/6 of head width; gena 1/9–1/12 of head height; three reclinate orbital setae; anteriormost reclinate orbital one 1/2 times longer than uppermost frontal seta; ocellar setae absent; outer vertical seta absent; fronto-orbital plate gold on uppermost 20–30%; fronto-orbital plate with short blonde hairs interspersed among frontal setae; parafacial brilliant silver; facial ridge bare; palpus digitiform, sparsely haired along outer margin; arista orange-brown plumose, distinctly-thickened on basal 1/10, microtrichia at most 3X as long as width of arista; postpedicel orange over at most 50% of surface; postocular region behind margin of eye upper 1/3 gold, with lower 2/3 including gena silver tomentose; upper half of occiput gold tomentose. **Thorax** (Fig. 32a, c): golden tomentose, with four almost indistinct dorsal stripes, outermost pair broken across suture, reaching just beyond 3rd postsutural dorsocentral, innermost pair slightly broken across suture, only extending up to 2nd postsutural dorsocentral; thorax with plumose hairs absent from disc of scutum, with black and blonde non-plumose hairs dorsally; chaetotaxy: 4–5 postpronotal setae, basal setae arranged in a straight line; supra-alar setae 2:3; intra-alar setae 1–2:3; dorsocentral setae 4:3; acrostichal setae 4:3; katepisternum with two setae. Scutellum golden tomentose; two pairs of strong marginal setae (basal and subapical) and a small pair of crossed apical scutellar setae 1/5th as long as subapical scutellars; basal scutellar setae subequal in length to subapical setae; subapical setae straight to slightly divergent; underside of scutellum bearing regular non-plumose blonde hairs below basal scutellar setae. **Legs**: foreleg yellow ground color, midleg yellow coxa and half of femur yellow remainder of femur and tarsi and tibia brown, hindleg dark brown extending from halfway along femur to tarsi; anterior leg tibia with regular fringe of short equally spaced setae along upper half of anteroventral surface, with one posterodorsal setae. **Wings**: basicosta ivory white; all veins bare, with only 1–2 setulae at base of R_4+5_; calypters pale white translucent with a pale yellow fringe. **Abdomen** (Fig. 32a, c): ground color yellow-orange; ST1+2 brown over medial 30%, with yellow ventrolaterally, extending into a longitudinal middorsal brown stripe up to posterior edge of T4; T3 and T4 each with dense gold tomentum along anterior marginal 10–20%, thinning and extending over remainder of tergite, basal 10%, lacking tomentum creating the appearance of dark bands; T5 entirely orange with gold tomentum and a basal medial brown triangle; median marginal setae present only on T4 and T5; median discal setae absent. **Male terminalia** (Fig. 32d, e, f): Sternite 5 with a narrow and shallow median cleft, strongly v-shaped, margins lightly pollinose; lateral lobes of sternite rounded apically, with 3–4 short weak setulae; basal section of sternite 5 subequal to slightly longer than length of apical lobes, median cleft only 1/3 length of apical section. Cerci in posterior view sharply pointed triangular at most a very slight shoulder along the basal 1/3, equal in length to surstyli, fused along entire length; in lateral view, with a slight downward curve on apical 1/3; several strong widely spaced setulae along basal 2/3rds. Surstylus in lateral view, almost equilateral along its length rounded at tip, slightly pinched at midpoint appearing digitiform or clubbed; surstylus appearing fused with epandrium; when viewed dorsally surstyli appear bowed, curving inwards and then slightly diverging again at apices. Basiphallus short and slender. Distiphallus subequal in length to basiphallus and tubular, very slightly sail-shaped at apex.

**Female.** Length: 5–7 mm (Fig. 33). **Head** (Fig. 33b): as in male with the following exceptions: fronto-orbital plate 30% gold; parafacial brilliant silver; frons 1/4 of head width; two inner reclinate orbital setae; two proclinate orbital setae; outer vertical seta present. **Thorax** (Fig. 33a, c): meron without any plumose hairs only 5–10 typical meral setae. Legs: foreleg yellow ground color, midleg yellow coxa with femur, tarsi overall brown, with yellow spot on proximal end of femur, hindleg entirely dark brown extending from entire femur to tarsi; anterior leg tibia with regular fringe of short irregularly spaced setae along upper half of anteroventral surface, with 1–2 posterodorsal setae. **Abdomen** (Fig. 33a, c): ground color brown dorsally on ST1+2 and T3 with orange laterally; T4 entirely brown ground color; T5 as in male.

#### Diagnosis

*Telothyria incisa* **sp. n.** can be distinguished from all other *Telothyria* by the following combination of traits: ocellar setae absent, fronto-orbital plate mostly silver with short blonde hairs, gold on uppermost 20–30%; plumose hairs absent from disc of scutum, katepisternum with two setae, and T5 orange with a dorsomedial dark triangle, and gold tomentum. Differs from its closest congener T. peltata, by the shallow v-shaped median cleft, 1/4 length of posterior section of sternite 5.

#### Etymology

*Telothyria incisa* **sp. n.** From the Latin noun “*incisus*”, meaning "cut into", in reference to the v-shaped median cleft in sternite 5.

#### Distribution

Costa Rica, ACG, Alajuela Province, 96–560 m elevation.

#### Ecology

*Telothyria incisa* **sp. n.** has been reared 106 times from one species of Lepidoptera in the family Crambidae: *Herpetogramma* Janzen07 in rain forest.

### Telothyria manuelpereirai

Fleming & Wood sp. n.

8E9C699C-32E1-5E76-8FF8-28205BA5016D

urn:lsid:zoobank.org:act:B3943E6B-4AD0-49F7-B44B-3D1262B678F3

#### Materials

- **Type status:** Holotype. **Occurrence:** occurrenceDetails: http://janzen.sas.upenn.edu; catalogNumber: DHJPAR0011567; recordedBy: D.H. Janzen, W. Hallwachs & Minor Carmona; individualID: DHJPAR0011567; individualCount: 1; sex: M; lifeStage: adult; preparations: pinned; otherCatalogNumbers: ASTAQ954-06, 05-SRNP-42650, BOLD:AAB8712; **Taxon:** scientificName: Telothyria manuelpereirai; phylum: Arthropoda; class: Insecta; order: Diptera; family: Tachinidae; genus: Telothyria; specificEpithet: manuelpereirai; scientificNameAuthorship: Fleming & Wood, 2018; **Location:** continent: Central America; country: Costa Rica; countryCode: CR; stateProvince: Alajuela; county: Sector Rincon Rain Forest; locality: Area de Conservacion Guanacaste; verbatimLocality: Sendero Tucan; verbatimElevation: 410; verbatimLatitude: 10.9042; verbatimLongitude: -85.2712; verbatimCoordinateSystem: Decimal; decimalLatitude: 10.9042; decimalLongitude: -85.2712; **Identification:** identifiedBy: AJ Fleming; dateIdentified: 2018; **Event:** samplingProtocol: Reared from the larve of the Crambidae, Piletosoma thialisDHJ03; verbatimEventDate: 15-Oct-2005; **Record Level:** language: en; institutionCode: CNC; collectionCode: Insects; basisOfRecord: Pinned Specimen
- **Type status:** Paratype. **Occurrence:** occurrenceDetails: http://janzen.sas.upenn.edu; catalogNumber: DHJPAR0006599; recordedBy: D.H. Janzen, W. Hallwachs & Jose Perez; individualID: DHJPAR0006599; individualCount: 1; sex: F; lifeStage: adult; preparations: pinned; otherCatalogNumbers: ASTA778-06, 05-SRNP-43026, BOLD:AAB8712; **Taxon:** scientificName: Telothyria manuelpereirai; phylum: Arthropoda; class: Insecta; order: Diptera; family: Tachinidae; genus: Telothyria; specificEpithet: manuelpereirai; scientificNameAuthorship: Fleming & Wood, 2018; **Location:** continent: Central America; country: Costa Rica; countryCode: CR; stateProvince: Alajuela; county: Sector Rincon Rain Forest; locality: Area de Conservacion Guanacaste; verbatimLocality: Sendero Rincon; verbatimElevation: 430; verbatimLatitude: 10.8962; verbatimLongitude: -85.2777; verbatimCoordinateSystem: Decimal; decimalLatitude: 10.8962; decimalLongitude: -85.2777; **Identification:** identifiedBy: AJ Fleming; dateIdentified: 2018; **Event:** samplingProtocol: Reared from the larve of the Crambidae, Piletosoma thialisDHJ03; verbatimEventDate: 23-Nov-2005; **Record Level:** language: en; institutionCode: CNC; collectionCode: Insects; basisOfRecord: Pinned Specimen
- **Type status:** Paratype. **Occurrence:** occurrenceDetails: http://janzen.sas.upenn.edu; catalogNumber: DHJPAR0006648; recordedBy: D.H. Janzen, W. Hallwachs & Minor Carmona; individualID: DHJPAR0006648; individualCount: 1; sex: M; lifeStage: adult; preparations: pinned; otherCatalogNumbers: ASTA826-06, 05-SRNP-43897, BOLD:AAB8712; **Taxon:** scientificName: Telothyria manuelpereirai; phylum: Arthropoda; class: Insecta; order: Diptera; family: Tachinidae; genus: Telothyria; specificEpithet: manuelpereirai; scientificNameAuthorship: Fleming & Wood, 2018; **Location:** continent: Central America; country: Costa Rica; countryCode: CR; stateProvince: Alajuela; county: Sector Rincon Rain Forest; locality: Area de Conservacion Guanacaste; verbatimLocality: Estacion Caribe; verbatimElevation: 415; verbatimLatitude: 10.9019; verbatimLongitude: -85.2749; verbatimCoordinateSystem: Decimal; decimalLatitude: 10.9019; decimalLongitude: -85.2749; **Identification:** identifiedBy: AJ Fleming; dateIdentified: 2018; **Event:** samplingProtocol: Reared from the larve of the Crambidae, Piletosoma thialisDHJ03; verbatimEventDate: 27-Jan-2006; **Record Level:** language: en; institutionCode: CNC; collectionCode: Insects; basisOfRecord: Pinned Specimen
- **Type status:** Paratype. **Occurrence:** occurrenceDetails: http://janzen.sas.upenn.edu; catalogNumber: DHJPAR0007127; recordedBy: D.H. Janzen, W. Hallwachs & Minor Carmona; individualID: DHJPAR0007127; individualCount: 1; sex: M; lifeStage: adult; preparations: pinned; otherCatalogNumbers: ASTAV369-06, 06-SRNP-40650, BOLD:AAB8712; **Taxon:** scientificName: Telothyria manuelpereirai; phylum: Arthropoda; class: Insecta; order: Diptera; family: Tachinidae; genus: Telothyria; specificEpithet: manuelpereirai; scientificNameAuthorship: Fleming & Wood, 2018; **Location:** continent: Central America; country: Costa Rica; countryCode: CR; stateProvince: Alajuela; county: Sector Rincon Rain Forest; locality: Area de Conservacion Guanacaste; verbatimLocality: Sendero Tucan; verbatimElevation: 410; verbatimLatitude: 10.9042; verbatimLongitude: -85.2712; verbatimCoordinateSystem: Decimal; decimalLatitude: 10.9042; decimalLongitude: -85.2712; **Identification:** identifiedBy: AJ Fleming; dateIdentified: 2018; **Event:** samplingProtocol: Reared from the larve of the Crambidae, Piletosoma thialisDHJ03; verbatimEventDate: 24-Mar-2006; **Record Level:** language: en; institutionCode: CNC; collectionCode: Insects; basisOfRecord: Pinned Specimen
- **Type status:** Paratype. **Occurrence:** occurrenceDetails: http://janzen.sas.upenn.edu; catalogNumber: DHJPAR0014977; recordedBy: D.H. Janzen, W. Hallwachs & Jose Perez; individualID: DHJPAR0014977; individualCount: 1; sex: M; lifeStage: adult; preparations: pinned; otherCatalogNumbers: ASTAV668-06, 06-SRNP-41922, BOLD:AAB8712; **Taxon:** scientificName: Telothyria manuelpereirai; phylum: Arthropoda; class: Insecta; order: Diptera; family: Tachinidae; genus: Telothyria; specificEpithet: manuelpereirai; scientificNameAuthorship: Fleming & Wood, 2018; **Location:** continent: Central America; country: Costa Rica; countryCode: CR; stateProvince: Alajuela; county: Sector Rincon Rain Forest; locality: Area de Conservacion Guanacaste; verbatimLocality: Sendero Tucan; verbatimElevation: 410; verbatimLatitude: 10.9042; verbatimLongitude: -85.2712; verbatimCoordinateSystem: Decimal; decimalLatitude: 10.9042; decimalLongitude: -85.2712; **Identification:** identifiedBy: AJ Fleming; dateIdentified: 2018; **Event:** samplingProtocol: Reared from the larve of the Crambidae, Piletosoma thialisDHJ02; verbatimEventDate: 29-Jun-2006; **Record Level:** language: en; institutionCode: CNC; collectionCode: Insects; basisOfRecord: Pinned Specimen
- **Type status:** Paratype. **Occurrence:** occurrenceDetails: http://janzen.sas.upenn.edu; catalogNumber: DHJPAR0014987; recordedBy: D.H. Janzen, W. Hallwachs & Minor Carmona; individualID: DHJPAR0014987; individualCount: 1; sex: F; lifeStage: adult; preparations: pinned; otherCatalogNumbers: ASTAV678-06, 06-SRNP-42052, BOLD:AAB8712; **Taxon:** scientificName: Telothyria manuelpereirai; phylum: Arthropoda; class: Insecta; order: Diptera; family: Tachinidae; genus: Telothyria; specificEpithet: manuelpereirai; scientificNameAuthorship: Fleming & Wood, 2018; **Location:** continent: Central America; country: Costa Rica; countryCode: CR; stateProvince: Alajuela; county: Sector Rincon Rain Forest; locality: Area de Conservacion Guanacaste; verbatimLocality: Sendero Anonas; verbatimElevation: 405; verbatimLatitude: 10.9053; verbatimLongitude: -85.2788; verbatimCoordinateSystem: Decimal; decimalLatitude: 10.9053; decimalLongitude: -85.2788; **Identification:** identifiedBy: AJ Fleming; dateIdentified: 2018; **Event:** samplingProtocol: Reared from the larve of the Crambidae, Piletosoma thialisDHJ02; verbatimEventDate: 06-Jul-2006; **Record Level:** language: en; institutionCode: CNC; collectionCode: Insects; basisOfRecord: Pinned Specimen
- **Type status:** Paratype. **Occurrence:** occurrenceDetails: http://janzen.sas.upenn.edu; catalogNumber: DHJPAR0010354; recordedBy: D.H. Janzen, W. Hallwachs & Minor Carmona; individualID: DHJPAR0010354; individualCount: 1; sex: F; lifeStage: adult; preparations: pinned; otherCatalogNumbers: ASTAS185-06, 06-SRNP-42553, BOLD:AAB8712; **Taxon:** scientificName: Telothyria manuelpereirai; phylum: Arthropoda; class: Insecta; order: Diptera; family: Tachinidae; genus: Telothyria; specificEpithet: manuelpereirai; scientificNameAuthorship: Fleming & Wood, 2018; **Location:** continent: Central America; country: Costa Rica; countryCode: CR; stateProvince: Alajuela; county: Sector Rincon Rain Forest; locality: Area de Conservacion Guanacaste; verbatimLocality: Sendero Rincon; verbatimElevation: 430; verbatimLatitude: 10.8962; verbatimLongitude: -85.2777; verbatimCoordinateSystem: Decimal; decimalLatitude: 10.8962; decimalLongitude: -85.2777; **Identification:** identifiedBy: AJ Fleming; dateIdentified: 2018; **Event:** samplingProtocol: Reared from the larve of the Crambidae, Piletosoma thialisDHJ03; verbatimEventDate: 13-Aug-2006; **Record Level:** language: en; institutionCode: CNC; collectionCode: Insects; basisOfRecord: Pinned Specimen
- **Type status:** Paratype. **Occurrence:** occurrenceDetails: http://janzen.sas.upenn.edu; catalogNumber: DHJPAR0011562; recordedBy: D.H. Janzen, W. Hallwachs & Minor Carmona; individualID: DHJPAR0011562; individualCount: 1; sex: M; lifeStage: adult; preparations: pinned; otherCatalogNumbers: ASTAQ949-06, 05-SRNP-42657, BOLD:AAB8712; **Taxon:** scientificName: Telothyria manuelpereirai; phylum: Arthropoda; class: Insecta; order: Diptera; family: Tachinidae; genus: Telothyria; specificEpithet: manuelpereirai; scientificNameAuthorship: Fleming & Wood, 2018; **Location:** continent: Central America; country: Costa Rica; countryCode: CR; stateProvince: Alajuela; county: Sector Rincon Rain Forest; locality: Area de Conservacion Guanacaste; verbatimLocality: Sendero Tucan; verbatimElevation: 410; verbatimLatitude: 10.9042; verbatimLongitude: -85.2712; verbatimCoordinateSystem: Decimal; decimalLatitude: 10.9042; decimalLongitude: -85.2712; **Identification:** identifiedBy: AJ Fleming; dateIdentified: 2018; **Event:** samplingProtocol: Reared from the larve of the Crambidae, Piletosoma thialisDHJ03; verbatimEventDate: 15-Oct-2005; **Record Level:** language: en; institutionCode: CNC; collectionCode: Insects; basisOfRecord: Pinned Specimen
- **Type status:** Paratype. **Occurrence:** occurrenceDetails: http://janzen.sas.upenn.edu; catalogNumber: DHJPAR0011565; recordedBy: D.H. Janzen, W. Hallwachs & Minor Carmona; individualID: DHJPAR0011565; individualCount: 1; sex: F; lifeStage: adult; preparations: pinned; otherCatalogNumbers: ASTAQ952-06, 05-SRNP-42654, BOLD:AAB8712; **Taxon:** scientificName: Telothyria manuelpereirai; phylum: Arthropoda; class: Insecta; order: Diptera; family: Tachinidae; genus: Telothyria; specificEpithet: manuelpereirai; scientificNameAuthorship: Fleming & Wood, 2018; **Location:** continent: Central America; country: Costa Rica; countryCode: CR; stateProvince: Alajuela; county: Sector Rincon Rain Forest; locality: Area de Conservacion Guanacaste; verbatimLocality: Sendero Tucan; verbatimElevation: 410; verbatimLatitude: 10.9042; verbatimLongitude: -85.2712; verbatimCoordinateSystem: Decimal; decimalLatitude: 10.9042; decimalLongitude: -85.2712; **Identification:** identifiedBy: AJ Fleming; dateIdentified: 2018; **Event:** samplingProtocol: Reared from the larve of the Crambidae, Piletosoma thialisDHJ03; verbatimEventDate: 12-Oct-2005; **Record Level:** language: en; institutionCode: CNC; collectionCode: Insects; basisOfRecord: Pinned Specimen
- **Type status:** Paratype. **Occurrence:** occurrenceDetails: http://janzen.sas.upenn.edu; catalogNumber: DHJPAR0011566; recordedBy: D.H. Janzen, W. Hallwachs & Minor Carmona; individualID: DHJPAR0011566; individualCount: 1; sex: M; lifeStage: adult; preparations: pinned; otherCatalogNumbers: ASTAQ953-06, 05-SRNP-42649, BOLD:AAB8712; **Taxon:** scientificName: Telothyria manuelpereirai; phylum: Arthropoda; class: Insecta; order: Diptera; family: Tachinidae; genus: Telothyria; specificEpithet: manuelpereirai; scientificNameAuthorship: Fleming & Wood, 2018; **Location:** continent: Central America; country: Costa Rica; countryCode: CR; stateProvince: Alajuela; county: Sector Rincon Rain Forest; locality: Area de Conservacion Guanacaste; verbatimLocality: Sendero Tucan; verbatimElevation: 410; verbatimLatitude: 10.9042; verbatimLongitude: -85.2712; verbatimCoordinateSystem: Decimal; decimalLatitude: 10.9042; decimalLongitude: -85.2712; **Identification:** identifiedBy: AJ Fleming; dateIdentified: 2018; **Event:** samplingProtocol: Reared from the larve of the Crambidae, Piletosoma thialisDHJ03; verbatimEventDate: 15-Oct-2005; **Record Level:** language: en; institutionCode: CNC; collectionCode: Insects; basisOfRecord: Pinned Specimen
- **Type status:** Paratype. **Occurrence:** occurrenceDetails: http://janzen.sas.upenn.edu; catalogNumber: DHJPAR0006598; recordedBy: D.H. Janzen, W. Hallwachs & Jose Perez; individualID: DHJPAR0006598; individualCount: 1; sex: M; lifeStage: adult; preparations: pinned; otherCatalogNumbers: ASTA777-06, 05-SRNP-43018, BOLD:AAB8712; **Taxon:** scientificName: Telothyria manuelpereirai; phylum: Arthropoda; class: Insecta; order: Diptera; family: Tachinidae; genus: Telothyria; specificEpithet: manuelpereirai; scientificNameAuthorship: Fleming & Wood, 2018; **Location:** continent: Central America; country: Costa Rica; countryCode: CR; stateProvince: Alajuela; county: Sector Rincon Rain Forest; locality: Area de Conservacion Guanacaste; verbatimLocality: Sendero Rincon; verbatimElevation: 430; verbatimLatitude: 10.8962; verbatimLongitude: -85.2777; verbatimCoordinateSystem: Decimal; decimalLatitude: 10.8962; decimalLongitude: -85.2777; **Identification:** identifiedBy: AJ Fleming; dateIdentified: 2018; **Event:** samplingProtocol: Reared from the larve of the Crambidae, Piletosoma thialisDHJ03; verbatimEventDate: 17-Nov-2005; **Record Level:** language: en; institutionCode: CNC; collectionCode: Insects; basisOfRecord: Pinned Specimen
- **Type status:** Paratype. **Occurrence:** occurrenceDetails: http://janzen.sas.upenn.edu; catalogNumber: DHJPAR0018609; recordedBy: D.H. Janzen, W. Hallwachs & Jose Perez; individualID: DHJPAR0018609; individualCount: 1; sex: F; lifeStage: adult; preparations: pinned; otherCatalogNumbers: ASTAI1256-07, 02-SRNP-7398, BOLD:AAB8712; **Taxon:** scientificName: Telothyria manuelpereirai; phylum: Arthropoda; class: Insecta; order: Diptera; family: Tachinidae; genus: Telothyria; specificEpithet: manuelpereirai; scientificNameAuthorship: Fleming & Wood, 2018; **Location:** continent: Central America; country: Costa Rica; countryCode: CR; stateProvince: Alajuela; county: Sector Rincon Rain Forest; locality: Area de Conservacion Guanacaste; verbatimLocality: Sendero Rincon; verbatimElevation: 430; verbatimLatitude: 10.8962; verbatimLongitude: -85.2777; verbatimCoordinateSystem: Decimal; decimalLatitude: 10.8962; decimalLongitude: -85.2777; **Identification:** identifiedBy: AJ Fleming; dateIdentified: 2018; **Event:** samplingProtocol: Reared from the larve of the Crambidae, Piletosoma thialisDHJ02; verbatimEventDate: 03-Jul-2002; **Record Level:** language: en; institutionCode: CNC; collectionCode: Insects; basisOfRecord: Pinned Specimen
- **Type status:** Paratype. **Occurrence:** occurrenceDetails: http://janzen.sas.upenn.edu; catalogNumber: DHJPAR0018610; recordedBy: D.H. Janzen, W. Hallwachs & Jose Perez; individualID: DHJPAR0018610; individualCount: 1; sex: F; lifeStage: adult; preparations: pinned; otherCatalogNumbers: ASTAI1257-07, 02-SRNP-7399, BOLD:AAB8712; **Taxon:** scientificName: Telothyria manuelpereirai; phylum: Arthropoda; class: Insecta; order: Diptera; family: Tachinidae; genus: Telothyria; specificEpithet: manuelpereirai; scientificNameAuthorship: Fleming & Wood, 2018; **Location:** continent: Central America; country: Costa Rica; countryCode: CR; stateProvince: Alajuela; county: Sector Rincon Rain Forest; locality: Area de Conservacion Guanacaste; verbatimLocality: Sendero Rincon; verbatimElevation: 430; verbatimLatitude: 10.8962; verbatimLongitude: -85.2777; verbatimCoordinateSystem: Decimal; decimalLatitude: 10.8962; decimalLongitude: -85.2777; **Identification:** identifiedBy: AJ Fleming; dateIdentified: 2018; **Event:** samplingProtocol: Reared from the larve of the Crambidae, Piletosoma thialisDHJ02; verbatimEventDate: 04-Jul-2002; **Record Level:** language: en; institutionCode: CNC; collectionCode: Insects; basisOfRecord: Pinned Specimen
- **Type status:** Paratype. **Occurrence:** occurrenceDetails: http://janzen.sas.upenn.edu; catalogNumber: DHJPAR0018611; recordedBy: D.H. Janzen, W. Hallwachs & Jose Perez; individualID: DHJPAR0018611; individualCount: 1; sex: F; lifeStage: adult; preparations: pinned; otherCatalogNumbers: ASTAI1258-07, 02-SRNP-7401, BOLD:AAB8712; **Taxon:** scientificName: Telothyria manuelpereirai; phylum: Arthropoda; class: Insecta; order: Diptera; family: Tachinidae; genus: Telothyria; specificEpithet: manuelpereirai; scientificNameAuthorship: Fleming & Wood, 2018; **Location:** continent: Central America; country: Costa Rica; countryCode: CR; stateProvince: Alajuela; county: Sector Rincon Rain Forest; locality: Area de Conservacion Guanacaste; verbatimLocality: Sendero Rincon; verbatimElevation: 430; verbatimLatitude: 10.8962; verbatimLongitude: -85.2777; verbatimCoordinateSystem: Decimal; decimalLatitude: 10.8962; decimalLongitude: -85.2777; **Identification:** identifiedBy: AJ Fleming; dateIdentified: 2018; **Event:** samplingProtocol: Reared from the larve of the Crambidae, Piletosoma thialisDHJ02; verbatimEventDate: 03-Jul-2002; **Record Level:** language: en; institutionCode: CNC; collectionCode: Insects; basisOfRecord: Pinned Specimen
- **Type status:** Paratype. **Occurrence:** occurrenceDetails: http://janzen.sas.upenn.edu; catalogNumber: DHJPAR0035703; recordedBy: D.H. Janzen, W. Hallwachs & Jose Perez; individualID: DHJPAR0035703; individualCount: 1; sex: M; lifeStage: adult; preparations: pinned; otherCatalogNumbers: ASHYD1084-09, 09-SRNP-41306, BOLD:AAB8712; **Taxon:** scientificName: Telothyria manuelpereirai; phylum: Arthropoda; class: Insecta; order: Diptera; family: Tachinidae; genus: Telothyria; specificEpithet: manuelpereirai; scientificNameAuthorship: Fleming & Wood, 2018; **Location:** continent: Central America; country: Costa Rica; countryCode: CR; stateProvince: Alajuela; county: Sector Rincon Rain Forest; locality: Area de Conservacion Guanacaste; verbatimLocality: Estacion Caribe; verbatimElevation: 415; verbatimLatitude: 10.9019; verbatimLongitude: -85.2749; verbatimCoordinateSystem: Decimal; decimalLatitude: 10.9019; decimalLongitude: -85.2749; **Identification:** identifiedBy: AJ Fleming; dateIdentified: 2018; **Event:** samplingProtocol: Reared from the larve of the Crambidae, Piletosoma thialis; verbatimEventDate: 14-Jul-2009; **Record Level:** language: en; institutionCode: CNC; collectionCode: Insects; basisOfRecord: Pinned Specimen
- **Type status:** Paratype. **Occurrence:** occurrenceDetails: http://janzen.sas.upenn.edu; catalogNumber: DHJPAR0035816; recordedBy: D.H. Janzen, W. Hallwachs & Noe Castillo; individualID: DHJPAR0035816; individualCount: 1; sex: F; lifeStage: adult; preparations: pinned; otherCatalogNumbers: ASHYD1197-09, 09-SRNP-69212, BOLD:AAB8712; **Taxon:** scientificName: Telothyria manuelpereirai; phylum: Arthropoda; class: Insecta; order: Diptera; family: Tachinidae; genus: Telothyria; specificEpithet: manuelpereirai; scientificNameAuthorship: Fleming & Wood, 2018; **Location:** continent: Central America; country: Costa Rica; countryCode: CR; stateProvince: Alajuela; county: Sector Rincon Rain Forest; locality: Area de Conservacion Guanacaste; verbatimLocality: Jacobo; verbatimElevation: 461; verbatimLatitude: 10.9408; verbatimLongitude: -85.3177; verbatimCoordinateSystem: Decimal; decimalLatitude: 10.9408; decimalLongitude: -85.3177; **Identification:** identifiedBy: AJ Fleming; dateIdentified: 2018; **Event:** samplingProtocol: Reared from the larve of the Crambidae, Piletosoma thialisDHJ02; verbatimEventDate: 19-Jul-2009; **Record Level:** language: en; institutionCode: CNC; collectionCode: Insects; basisOfRecord: Pinned Specimen
- **Type status:** Paratype. **Occurrence:** occurrenceDetails: http://janzen.sas.upenn.edu; catalogNumber: DHJPAR0035819; recordedBy: D.H. Janzen, W. Hallwachs & Noe Castillo; individualID: DHJPAR0035819; individualCount: 1; sex: F; lifeStage: adult; preparations: pinned; otherCatalogNumbers: ASHYD1200-09, 09-SRNP-69246, BOLD:AAB8712; **Taxon:** scientificName: Telothyria manuelpereirai; phylum: Arthropoda; class: Insecta; order: Diptera; family: Tachinidae; genus: Telothyria; specificEpithet: manuelpereirai; scientificNameAuthorship: Fleming & Wood, 2018; **Location:** continent: Central America; country: Costa Rica; countryCode: CR; stateProvince: Alajuela; county: Sector Rincon Rain Forest; locality: Area de Conservacion Guanacaste; verbatimLocality: Jacobo; verbatimElevation: 461; verbatimLatitude: 10.9408; verbatimLongitude: -85.3177; verbatimCoordinateSystem: Decimal; decimalLatitude: 10.9408; decimalLongitude: -85.3177; **Identification:** identifiedBy: AJ Fleming; dateIdentified: 2018; **Event:** samplingProtocol: Reared from the larve of the Crambidae, Piletosoma thialisDHJ03; verbatimEventDate: 18-Jul-2009; **Record Level:** language: en; institutionCode: CNC; collectionCode: Insects; basisOfRecord: Pinned Specimen
- **Type status:** Paratype. **Occurrence:** occurrenceDetails: http://janzen.sas.upenn.edu; catalogNumber: DHJPAR0040183; recordedBy: D.H. Janzen, W. Hallwachs & Ricardo Calero; individualID: DHJPAR0040183; individualCount: 1; sex: M; lifeStage: adult; preparations: pinned; otherCatalogNumbers: ASHYE2350-11, 10-SRNP-71645, BOLD:AAB8712; **Taxon:** scientificName: Telothyria manuelpereirai; phylum: Arthropoda; class: Insecta; order: Diptera; family: Tachinidae; genus: Telothyria; specificEpithet: manuelpereirai; scientificNameAuthorship: Fleming & Wood, 2018; **Location:** continent: Central America; country: Costa Rica; countryCode: CR; stateProvince: Guanacaste; county: Sector Pitilla; locality: Area de Conservacion Guanacaste; verbatimLocality: Charia; verbatimElevation: 530; verbatimLatitude: 10.9934; verbatimLongitude: -85.4027; verbatimCoordinateSystem: Decimal; decimalLatitude: 10.9934; decimalLongitude: -85.4027; **Identification:** identifiedBy: AJ Fleming; dateIdentified: 2018; **Event:** samplingProtocol: Reared from the larve of the Crambidae, Piletosoma thialis; verbatimEventDate: 09-Jul-2010; **Record Level:** language: en; institutionCode: CNC; collectionCode: Insects; basisOfRecord: Pinned Specimen
- **Type status:** Paratype. **Occurrence:** occurrenceDetails: http://janzen.sas.upenn.edu; catalogNumber: DHJPAR0040201; recordedBy: D.H. Janzen, W. Hallwachs & Ricardo Calero; individualID: DHJPAR0040201; individualCount: 1; sex: M; lifeStage: adult; preparations: pinned; otherCatalogNumbers: ASHYE2368-11, 10-SRNP-71639, BOLD:AAB8712; **Taxon:** scientificName: Telothyria manuelpereirai; phylum: Arthropoda; class: Insecta; order: Diptera; family: Tachinidae; genus: Telothyria; specificEpithet: manuelpereirai; scientificNameAuthorship: Fleming & Wood, 2018; **Location:** continent: Central America; country: Costa Rica; countryCode: CR; stateProvince: Guanacaste; county: Sector Pitilla; locality: Area de Conservacion Guanacaste; verbatimLocality: Charia; verbatimElevation: 530; verbatimLatitude: 10.9934; verbatimLongitude: -85.4027; verbatimCoordinateSystem: Decimal; decimalLatitude: 10.9934; decimalLongitude: -85.4027; **Identification:** identifiedBy: AJ Fleming; dateIdentified: 2018; **Event:** samplingProtocol: Reared from the larve of the Crambidae, Piletosoma thialis; verbatimEventDate: 27-Jun-2010; **Record Level:** language: en; institutionCode: CNC; collectionCode: Insects; basisOfRecord: Pinned Specimen

#### Description

**Male.** Length: 7–10 mm (Fig. 34). **Head** (Fig. 34b): frons narrow, 1/5 of head width; gena 1/8 of head height; three reclinate orbital setae; anteriormost reclinate orbital subequal to uppermost frontal seta; ocellar setae present but so small as to appear absent; outer vertical seta absent; fronto-orbital plate over 90% silver, with brassy tinge at most covering ocellar triangle and adjacent area; fronto-orbital plate with short blonde hairs interspersed among frontal setae; parafacial pale silver; facial ridge bare; palpus with a slight spatulate club at apex, sparsely haired along outer margin; arista orange-brown smoothly tapered, minutely plumose microtrichia at most as long as width of arista; postpedicel orange over 50% of surface; postocular region behind margin of eye upper 1/3 gold, with lower 2/3 including gena silver tomentose; upper 1/3 of occiput gold tomentose. **Thorax** (Fig. 34a, c): golden tomentose, with four distinct dorsal stripes; entire thorax covered in dense plumose blonde hairs, hairs extending to cover scutellum; chaetotaxy: 5–6 postpronotal setae, basal setae arranged in a straight line; supra-alar setae 2:3; intra-alar setae 2:3; dorsocentral setae 3:3; acrostichal setae 3:3; katepisternum with two setae. Scutellum golden tomentose; two pairs of strong marginal setae (basal and subapical) and a small pair of crossed apical scutellar setae 1/8–1/10th as long as subapical scutellars; basal scutellar setae subequal in length to subapical setae; subapical setae straight; underside of scutellum bearing plumose blonde hairs below basal scutellar setae. **Legs**: foreleg with yellow ground color; midleg yellow coxa and femur with brown tibia and tarsal segments; hindleg yellow ground color on proximal 50% of femur, transitioning to dark brown distally, tibia and tarsal segments; anterior leg tibia with regular fringe of equally spaced setae along anteroventral surface, and two widely spaced posterodorsal setae. **Wings**: basicosta bright ivory white; all veins bare, with only 1–2 setulae at base of R_4+5_; calypters pale white translucent with narrow beige fringes. **Abdomen** (Fig. 34a, c): ground color yellow-orange; ST1+2 yellow over more than 50% of tergite, with dark brown ground color medially along the middorsal depression, extending into a longitudinal middorsal brown stripe up to posterior edge of T4; ST1+2 with golden plumose hairs present on ventral surface; T3 and T4 each with dense gold tomentum along anterior marginal 10%, thinning and extending over remainder of tergite; T5 entirely orange with gold tomentum; one pair of median marginal setae weak almost undifferentiated on T3; row of marginal setae present only on T4 and T5; median discal setae absent. **Male terminalia** (Fig. 34d, e, f): Sternite 5 with a wide deeply excavated median cleft, smoothly V-shaped, margins covered in dense pollinosity; lateral lobes of sternite pointed apically, with a small group of strong setulae along outer margins; basal section of sternite 5 subequal to slightly shorter than length of apical lobes. Cerci in posterior view sharply pointed and slender, abruptly widening to a moderate almost rectangular shoulder along the basal section, equal in length to surstyli, fused along entire length; in lateral view, with a smooth regular downward curve along apical 2/3rds; several strong widely spaced setulae along basal 1/3rd. Surstylus in lateral view, almost equilateral along its length rounded at tip, digitiform; surstylus appearing fused with epandrium; when viewed dorsally surstyli appear slender with an inward bend. Distiphallus 2X as long as basiphallus and tubular, slightly pointed at apex.

**Female.** Length: 5–9 mm (Fig. 35). **Head** (Fig. 35b): as in male with the following exceptions: fronto-orbital plate 90% silver; parafacial brilliant silver; frons 1/5 of head width; three inner reclinate orbital setae; two proclinate orbital setae; and outer vertical setae present. **Thorax** (Fig. 35a, c): katepisternum with three setae; meron densely covered in plumose hairs as in male but with the addition of 2–4 typical meral setae. Legs: colored as in male. **Abdomen** (Fig. 35a, c): ground color brown dorsally on ST1+2 and T3 with large patches of orange ventrolaterally; T4 entirely brown ground color; T5 entirely orange with gold tomentum; T3 with one strong pair of median marginal setae and T4, T5 with a complete row of marginal setae; T4 bearing two weak discal setae scarcely distinguishable from surrounding hairs.

#### Diagnosis

*Telothyria manuelpereirai* **sp. n.** can be distinguished from all other *Telothyria* by the following combination of traits: ocellar setae present but so small as to appear absent, postpedicel orange over 50% of surface, entire thorax covered in dense plumose blonde hairs, hairs extending to cover scutellum, katepisternum with two setae in males and three setae in females, and T5 yellow with silver tomentum.

#### Etymology

*Telothyria manuelpereirai* **sp. n.** is named in recognition of Manuel Pereira's outstanding work on the team that conducts the caterpillar and parasite inventory from ACG’s Estación Biológica Cacao.

#### Distribution

Costa Rica, ACG, Alajuela and Guanacaste Provinces, 410–530 m elevation.

#### Ecology

*Telothyria manuelpereirai* **sp. n.** has been reared 20 times from three species of Lepidoptera in the family Crambidae: *Piletosoma thialis* Dyar, 1914, *Piletosoma thialis*DHJ02, and *Piletosoma thialis*DHJ03, in rain forest.

### Telothyria obscura

Fleming & Wood sp. n.

C5DE3ADD-0278-5BFE-8B58-1AE5B06B0390

urn:lsid:zoobank.org:act:163E5D17-65C3-41C1-A211-83ED9C4C6F0D

#### Materials

- **Type status:** Holotype. **Occurrence:** catalogNumber: CNC618905; recordedBy: D.M. Wood; individualID: CNC618905; individualCount: 1; sex: M; lifeStage: adult; preparations: pinned; **Taxon:** scientificName: Telothyria obscura; phylum: Arthropoda; class: Insecta; order: Diptera; family: Tachinidae; genus: Telothyria; specificEpithet: obscura; scientificNameAuthorship: Fleming & Wood, 2019; **Location:** continent: Central America; country: Costa Rica; countryCode: CR; stateProvince: Puntarenas; verbatimLocality: Monteverde; verbatimElevation: 1799; **Identification:** identifiedBy: AJ Fleming; dateIdentified: 2019; **Event:** samplingProtocol: Hand collected; verbatimEventDate: 20-Aug-1991; **Record Level:** language: en; institutionCode: CNC; collectionCode: Insects; basisOfRecord: Pinned Specimen
- **Type status:** Paratype. **Occurrence:** catalogNumber: CNC618892; recordedBy: D.M. Wood; individualID: CNC618892; sex: M; lifeStage: adult; preparations: pinned; **Taxon:** scientificName: Telothyria obscura; phylum: Arthropoda; class: Insecta; order: Diptera; family: Tachinidae; genus: Telothyria; specificEpithet: obscura; scientificNameAuthorship: Fleming & Wood, 2019; **Location:** continent: Central America; country: Costa Rica; countryCode: CR; stateProvince: Puntarenas; verbatimLocality: Monteverde Cerro; verbatimElevation: 1800; **Identification:** identifiedBy: AJ Fleming; dateIdentified: 2019; **Event:** samplingProtocol: Hand collected; verbatimEventDate: 22-30-Aug-1996; **Record Level:** language: en; institutionCode: CNC; collectionCode: Insects; basisOfRecord: Pinned Specimen
- **Type status:** Paratype. **Occurrence:** recordedBy: D.M. Wood; sex: M; lifeStage: adult; preparations: pinned; **Taxon:** scientificName: Telothyria obscura; phylum: Arthropoda; class: Insecta; order: Diptera; family: Tachinidae; genus: Telothyria; specificEpithet: obscura; scientificNameAuthorship: Fleming & Wood, 2019; **Location:** continent: Central America; country: Costa Rica; countryCode: CR; stateProvince: Puntarenas; verbatimLocality: Monteverde; verbatimElevation: 1799; **Identification:** identifiedBy: AJ Fleming; dateIdentified: 2019; **Event:** samplingProtocol: Hand collected; verbatimEventDate: 20-Aug-1991; **Record Level:** language: en; institutionCode: CNC; collectionCode: Insects; basisOfRecord: Pinned Specimen
- **Type status:** Paratype. **Occurrence:** recordedBy: D.M. Wood; sex: M; lifeStage: adult; preparations: pinned; **Location:** continent: Central America; country: Costa Rica; countryCode: CR; stateProvince: Puntarenas; verbatimLocality: Monteverde; verbatimElevation: 1799; **Identification:** identifiedBy: AJ Fleming; dateIdentified: 2019; **Event:** samplingProtocol: Hand collected; verbatimEventDate: 20-Aug-1991; **Record Level:** language: en; institutionCode: CNC; collectionCode: Insects; basisOfRecord: Pinned Specimen

#### Description

**Male.** Length: 5–8 mm (Fig. 36). **Head** (Fig. 36b): frons wide almost 1/5 of head width; gena 1/10 of head height; three reclinate orbital setae; anteriormost reclinate orbital seta 1.25X longer than uppermost frontal seta; ocellar setae absent; outer vertical seta absent; fronto-orbital plate gold on upper 30%, brilliant silver on remainder; fronto-orbital plate densely covered with both pale blonde and light reddish-brown hairs interspersed among frontal setae; parafacial pale brilliant silver; facial ridge bare; palpus short yellow and digitiform with slight upward turn apically, sparsely haired along outer margin; arista brown, smoothly tapered, microtrichia at most 2X as long as width of arista; pedicel orange, postpedicel with slight orange apex, adjacent to pedicel; postocular region behind margin of eye upper half gold, with lower half including gena silver tomentose; upper half of occiput pale gold tomentose, lower half silver tomentose. **Thorax** (Fig. 36a, c): dark brownish ground color with brown-bronze tomentum lightening to almost gold color along lateral edges of scutum, with four dorsal stripes, thick and evident, innermost pair ending at 1st postsutural dorsocentral, outermost pair slightly broken across suture, plus one extra dorsal stripe dorsocentrally ending at suture; thorax densely covered in plumose blonde hairs only along lateral surfaces; chaetotaxy: 4–5 postpronotal setae, basal setae arranged in a straight line; supra-alar setae 2:3; intra-alar setae 2:2; dorsocentral setae 3:3; acrostichal setae 3:3; katepisternum with three setae. Scutellum dark brown with slight bronze-gold tomentum only along apex; two pairs of strong marginal setae (basal and subapical) and a small pair of crossed apical scutellar setae 1/5th as long as subapical scutellars; basal scutellar setae subequal in length to subapical setae; subapical setae straight; underside of scutellum bearing predominantly plumose blonde hairs with few interspersed regular non-plumose black hairs below basal scutellar setae. **Legs**: all legs with an overall light reddish-brown ground color throughout with silver tomentum on posterodorsal surfaces, tibia yellow ground color with dense black hairs giving them an overall dark appearance, tarsal segments appearing dark brown; anterior leg tibia with irregularly sized tapered fringe of equally spaced setae along basal half of anterodorsal surface, with one posterodorsal setae. **Wings**: basicosta beige, brown basally; all veins bare, and very slightly infuscate, with one setula at base of R_4+5_; calypters infuscate brown translucent with white fringe densely populated with short translucente microsetulae only visible under certain angles of light. **Abdomen** (Fig. 36a, c): ground color dark brown dorsally, T1+2–T4 with yellow ventrolaterally and T5 brown with orange apically; T4 with a complete unbroken band of dark ground color along posterior edge; T3–T5 with brassy-brown tomentu throughout and dense gold tomentum along anterior margin of tergites, marginal tomentum broken along midline; median marginal setae present only on T4 and T5; median discal setae absent. **Terminalia** (Fig. 36d, e, f): Sternite 5 with a wide deeply separated median cleft, widely V-shaped, margins tomentose; lateral lobes of sternite subtriangular apically, outer margins covered in strong setae; basal section of sternite 5 subequal to length of apical lobes. Cerci in posterior view sharply pointed rectangular, equal in length to surstyli, fused along entire length; evenly tapering with medial shoulder absent. In lateral view cerci, with a strong downward bend, along apical 1/3, and several strong widely spaced setae along basal 2/3rds. Surstylus in lateral view broad and leaf-shaped, pointed at tip; fused with epandrium; when viewed dorsally surstyli appear robust and straight with a very slight club apically. Basiphallus short and stout and stout, distiphallus subequal to in length to basiphallus, weakly tapering apically.

**Female.** Unknown at this time.

#### Diagnosis

*Telothyria obscura* **sp. n.** can be distinguished from all other *Telothyria* by the following combination of traits: ocellar setae absent, fronto-orbital plate gold on upper 30%, brilliant silver on remainder, parafacial brilliant silver, postpedicel more than 50% black, only orange adjacent to pedicel, plumose hairs absent on disc of scutum, thorax with four thoracic stripes plus one extra presutural stripe dorsomedially, and three katepisternal setae, abdominal ground color orange with a brownish-black middorsal stripe occupying almost entire dorsal surface of tergites, abdomen brassy-brown tomentose throughout, with dark orange lateroventrally from ST1+2–T5, and T5 orange apically with brassy-brown sheen of tomentum. *Telothyria obscura* differs from *Telothyria omissa* by the presence of median marginal setae on T4.

#### Etymology

*Telothyria obscura* **sp. n.** From the Latin adjective, “*obscurus*” meaning dark or dim, in reference to the darkened nature of the dorsal surface of the thorax and abdomen.

#### Distribution

Costa Rica, Puntarenas Province, Monteverde 1799–1800 m elevation.

#### Ecology

Specimens hand collected, four times from 1800 m, further ecology not available.

### Telothyria omissa

Fleming & Wood sp. n.

394CDDAC-6FE7-5AC5-AAE0-B4E0609076EA

urn:lsid:zoobank.org:act:1B70E2AA-F774-41BF-9FAD-E3C967324A18

#### Materials

- **Type status:** Holotype. **Occurrence:** catalogNumber: CNC618906; recordedBy: D.M. Wood; individualID: CNC618906; individualCount: 1; sex: M; lifeStage: adult; preparations: pinned; **Taxon:** scientificName: Telothyria omissa; phylum: Arthropoda; class: Insecta; order: Diptera; family: Tachinidae; genus: Telothyria; specificEpithet: omissa; scientificNameAuthorship: Fleming & Wood, 2019; **Location:** continent: Central America; country: Mexico; countryCode: MX; stateProvince: Chiapas; verbatimLocality: 6 km SE of Ocosingo; verbatimElevation: 1400; **Identification:** identifiedBy: AJ Fleming; dateIdentified: 2019; **Event:** samplingProtocol: Hand collected; verbatimEventDate: 20-Aug-1992; **Record Level:** language: en; institutionCode: CNC; collectionCode: Insects; basisOfRecord: Pinned Specimen

#### Description

**Male.** Length: 7 mm (Fig. 37). **Head** (Fig. 37b): frons narrow, 1/5 of head width; gena 1/12 of head height; three reclinate orbital setae; anteriormost reclinate orbital almost equal to uppermost frontal seta; ocellar setae absent; outer vertical seta absent; fronto-orbital plate brassy-gray throughout; fronto-orbital plate with short blonde hairs interspersed among frontal setae; parafacial brilliant silver; facial ridge bare; palpus short digitiform with slight club apically, sparsely haired along outer margin, slightly denser apically giving apex a darkened tone relative to rest of palpus; arista brown, basally orange, smoothly tapered, with microtrichia at most as long as width of arista; postpedicel only 40% orange, directly adjacent to pedicel; postocular region behind margin of eye upper half gold, with lower half including gena silver tomentose; upper half of occiput brassy-silver tomentose, remainder silver. **Thorax** (Fig. 37a, c): dark brown ground color, with gold tomentum, with four distinct dorsal stripes, outer pair almost unbroken across suture, and inner pair extending almost to second postsutural dorsocentral seta; thorax laterally covered in dense plumose blonde hairs, dorsally covered in long dark hairs; chaetotaxy: 5 postpronotal setae, basal setae arranged in a straight line; supra-alar setae 2:3; intra-alar setae 1:2; dorsocentral setae 3:3; acrostichal setae 3:3; katepisternum with three setae. Scutellum dark with light gold color along posterior margin; two pairs of strong marginal setae (basal and subapical) and a small pair of crossed apical scutellar setae 1/5th as long as subapical scutellars; basal scutellar setae subequal in length to subapical setae; subapical setae straight; underside of scutellum bearing plumose blonde hairs below basal scutellar setae. **Legs**: all legs with an overall light reddish-brown ground color throughout with silver tomentum on posterodorsal surfaces, tibia yellow ground color with dense black hairs giving them an overall dark appearance, tarsal segments appearing dark brown; anterior leg tibia with regular fringe of equally spaced setae along anteroventral surface, and one posterodorsal seta. **Wings**: basicosta beige; all veins bare, with only 1–2 setulae at base of R_4+5_; calypters pale cinereous translucent, with a narrow yellowish fringe. **Abdomen** (Fig. 37a, c): ground color dark yellow laterally, with dark brown dorsocentrally and dark brown ventrally; ST1+2 brown medially over central 40%, ST1+2–T5 yellow laterally; T3–T5 with dense sheen of light gold tomentum extending over entire tergite appearing to have a gold sheen when viewed with the naked eye; T5 with orange apically; strong lateral marginal setae on T3; marginal setae absent from T4, row of marginal setae on T5; median discal setae absent. **Male terminalia**: not examined.

**Female.** Unknown at this time.

#### Diagnosis

*Telothyria omissa* **sp. n.** can be distinguished from all other *Telothyria* by the following combination of traits: ocellar setae absent, fronto-orbital plate brassy-gray, parafacial brilliant silver, postpedicel orange, thorax with four thoracic stripes and three katepisternal setae, abdominal ground color orange with a brownish middorsal stripe, with dark orange lateroventrally from ST1+2–T5, plumose hairs on thorax absent dorsally, and T5 orange apically with gold sheen of tomentum. *Telothyria omissa* differs from *Telothyria obscura* by the absence of median marginal setae on T4, and the underside of scutellum bearing only plumose blonde hairs below basal scutellar setae.

#### Etymology

*Telothyria omissa* **sp. n.** From the Latin adjective, “*omissus*” meaning lacking, in reference to the lack median marginal setae on T3 and T4.

#### Distribution

Mexico, Chiapas, Ocosingo 6km SW, 1400 m elevation.

#### Ecology

Specimens hand collected once at 1400 m, further ecology not available.

### Telothyria osvaldoespinozai

Fleming & Wood sp. n.

43B0E727-2943-5354-B743-2260F3B23DDD

urn:lsid:zoobank.org:act:8FE092AB-77D3-460A-8806-9BF7C1497AF7

#### Materials

- **Type status:** Holotype. **Occurrence:** occurrenceDetails: http://janzen.sas.upenn.edu; catalogNumber: DHJPAR0045657; recordedBy: D.H. Janzen, W. Hallwachs & Cirilo Umana; individualID: DHJPAR0045657; individualCount: 1; sex: M; lifeStage: adult; preparations: pinned; otherCatalogNumbers: ACGAZ846-11, 11-SRNP-67582, BOLD:ABU7495; **Taxon:** scientificName: Telothyria osvaldoespinozai; phylum: Arthropoda; class: Insecta; order: Diptera; family: Tachinidae; genus: Telothyria; specificEpithet: osvaldoespinozai; scientificNameAuthorship: Fleming & Wood, 2018; **Location:** continent: Central America; country: Costa Rica; countryCode: CR; stateProvince: Alajuela; county: Sector Rincon Rain Forest; locality: Area de Conservacion Guanacaste; verbatimLocality: Palomo; verbatimElevation: 96; verbatimLatitude: 10.9619; verbatimLongitude: -85.2804; verbatimCoordinateSystem: Decimal; decimalLatitude: 10.9619; decimalLongitude: -85.2804; **Identification:** identifiedBy: AJ Fleming; dateIdentified: 2018; **Event:** samplingProtocol: Reared from the larva of the Crambidae, Herpetogramma phaeopteralis; verbatimEventDate: 02-Sep-2011; **Record Level:** language: en; institutionCode: CNC; collectionCode: Insects; basisOfRecord: Pinned Specimen

#### Description

**Male.** Length: 6 mm (Fig. 38). **Head** (Fig. 38a, b): frons narrow, 1/5 of head width; gena 1/10 of head height; four reclinate orbital setae uppermost reclinate orbital pair slightly convergent; anteriormost reclinate orbital shorter than uppermost frontal seta; ocellar setae absent; outer vertical seta absent; ocellar triangle and fronto-orbital plate pale brassy-gold; fronto-orbital plate with short blonde hairs interspersed among frontal setae; parafacial dull gray-silver; facial ridge bare; palpus digitiform, apically terminating in a small bulbous club; arista brown, smoothly tapering to apical 1/8, microtrichia at most 1.5X as long as width of arista; postpedicel orange over at most 30% of surface; postocular region behind margin of eye upper half gold, with lower half including gena silver tomentose; upper half of occiput gold tomentose. **Thorax** (Fig. 38a, c): brassy-gold tomentose, with two distinct outer dorsal stripes, and two short inner stripes; thorax covered in dense plumose blonde hairs laterally, absent dorsally; chaetotaxy: five postpronotal setae, basal setae arranged in a straight line; supra-alar setae 2:3; intra-alar setae 2:2; dorsocentral setae 3:3; acrostichal setae 3:3; katepisternum with three setae. Scutellum brassy-gold tomentose; two pairs of strong marginal setae (basal and subapical) and a small pair of crossed apical scutellar setae 1/8–1/10th as long as subapical scutellars; basal scutellar setae subequal in length to subapical setae; subapical setae straight; underside of scutellum bearing plumose blonde hairs below basal scutellar setae. **Legs**: foreleg brown coxa and proximal half of femur, yellow with ground color extending from distal half of femur, tarsal segments darkened by vestiture of microsetulae; both midleg and hindleg with yellow coxa, and remainder dark brown entirely; anterior leg tibia with smoothly tapering fringe of equally spaced setae along anteroventral surface, and two posterodorsal setae. **Wings**: basicosta ivory/beige; all veins bare, with only one setula at base of R_4+5_; calypters pale white translucent with narrow pale yellow fringe. **Abdomen** (Fig. 38a, c): ground color yellow-orange; ST1+2 brown over medial 30%, with yellow ventrolaterally, extending into a longitudinal middorsal brown stripe bisected by a brown band along posterior edges of T3 and T4; T1+2–T4 with pale silver tomentum extending over entire tergite, anterior margin of tergites beige tomentose; T5 entirely yellow, bearing some beige colored tomentum; marginal setae present on T4 1/2 as long as those present on and T5; median discal setae absent. **Male terminalia**: not examined.

**Female.** Unknown at this time.

#### Diagnosis

*Telothyria osvaldoespinozai* **sp. n.** can be distinguished from all other *Telothyria* by the following combination of traits: ocellar setae absent, plumose hairs absent from disc of scutum, katepisternum with three setae, two postsutural intra-alar setae, underside of scutellum bearing plumose blonde hairs below basal scutellar setae, and T5 yellow with silver tomentum.

#### Etymology

*Telothyria osvaldoespinozai* **sp. n.** is named in recognition of Osvaldo Espinoza’s outstanding work on the team that conducts the caterpillar and parasite inventory from ACG’s Estación Biológica San Gerardo.

#### Distribution

Costa Rica, ACG, Alajuela Province, 96 m elevation.

#### Ecology

*Telothyria osvaldoespinozai* **sp. n.** has been reared once from a single species of Lepidoptera in the family Crambidae: *Herpetogramma phaeopteralis* (Guenée, 1854), in rain forest.

### Telothyria peltata

Fleming & Wood sp. n.

222596FE-1326-5B13-A7F5-750184DA284D

urn:lsid:zoobank.org:act:ED8375CB-4D25-4CCB-BA99-BD3EAFE3C62A

#### Materials

- **Type status:** Holotype. **Occurrence:** occurrenceDetails: http://janzen.sas.upenn.edu; catalogNumber: DHJPAR0050298; recordedBy: D.H. Janzen, W. Hallwachs & Gloria Sihezar; individualID: DHJPAR0050298; individualCount: 1; sex: M; lifeStage: adult; preparations: pinned; otherCatalogNumbers: ACGAZ1612-12, 12-SRNP-3715,; **Taxon:** scientificName: Telothyria peltata; phylum: Arthropoda; class: Insecta; order: Diptera; family: Tachinidae; genus: Telothyria; specificEpithet: peltata; scientificNameAuthorship: Fleming & Wood, 2018; **Location:** continent: Central America; country: Costa Rica; countryCode: CR; stateProvince: Alajuela; county: Sector San Cristobal; locality: Area de Conservacion Guanacaste; verbatimLocality: Sendero Huerta; verbatimElevation: 527; verbatimLatitude: 10.9305; verbatimLongitude: -85.3722; verbatimCoordinateSystem: Decimal; decimalLatitude: 10.9305; decimalLongitude: -85.3722; **Identification:** identifiedBy: AJ Fleming; dateIdentified: 2018; **Event:** samplingProtocol: Reared from the larva of the Crambidae, Herpetogramma Janzen07; verbatimEventDate: 30-Sep-2012; **Record Level:** language: en; institutionCode: CNC; collectionCode: Insects; basisOfRecord: Pinned Specimen
- **Type status:** Paratype. **Occurrence:** occurrenceDetails: http://janzen.sas.upenn.edu; catalogNumber: DHJPAR0050636; recordedBy: D.H. Janzen, W. Hallwachs & Edwin Apu; individualID: DHJPAR0050636; individualCount: 1; sex: M; lifeStage: adult; preparations: pinned; otherCatalogNumbers: ACGBA3228-13, 12-SRNP-81895, BOLD:AAZ2421; **Taxon:** scientificName: Telothyria peltata; phylum: Arthropoda; class: Insecta; order: Diptera; family: Tachinidae; genus: Telothyria; specificEpithet: peltata; scientificNameAuthorship: Fleming & Wood, 2018; **Location:** continent: Central America; country: Costa Rica; countryCode: CR; stateProvince: Alajuela; county: Sector Rincon Rain Forest; locality: Area de Conservacion Guanacaste; verbatimLocality: Jacobo; verbatimElevation: 461; verbatimLatitude: 10.9408; verbatimLongitude: -85.3177; verbatimCoordinateSystem: Decimal; decimalLatitude: 10.9408; decimalLongitude: -85.3177; **Identification:** identifiedBy: AJ Fleming; dateIdentified: 2018; **Event:** samplingProtocol: Reared from the larva of the Crambidae, Herpetogramma Janzen07; verbatimEventDate: 06-Dec-2012; **Record Level:** language: en; institutionCode: CNC; collectionCode: Insects; basisOfRecord: Pinned Specimen
- **Type status:** Paratype. **Occurrence:** occurrenceDetails: http://janzen.sas.upenn.edu; catalogNumber: DHJPAR0049621; recordedBy: D.H. Janzen, W. Hallwachs & Calixto Moraga; individualID: DHJPAR0049621; individualCount: 1; sex: F; lifeStage: adult; preparations: pinned; otherCatalogNumbers: ASHYB2415-12, 12-SRNP-30992,; **Taxon:** scientificName: Telothyria peltata; phylum: Arthropoda; class: Insecta; order: Diptera; family: Tachinidae; genus: Telothyria; specificEpithet: peltata; scientificNameAuthorship: Fleming & Wood, 2018; **Location:** continent: Central America; country: Costa Rica; countryCode: CR; stateProvince: Guanacaste; county: Sector Pitilla; locality: Area de Conservacion Guanacaste; verbatimLocality: Sendero Carica; verbatimElevation: 660; verbatimLatitude: 10.9928; verbatimLongitude: -85.4294; verbatimCoordinateSystem: Decimal; decimalLatitude: 10.9928; decimalLongitude: -85.4294; **Identification:** identifiedBy: AJ Fleming; dateIdentified: 2018; **Event:** samplingProtocol: Reared from the larva of the Crambidae, Herpetogramma Janzen07; verbatimEventDate: 07-Jul-2012; **Record Level:** language: en; institutionCode: CNC; collectionCode: Insects; basisOfRecord: Pinned Specimen
- **Type status:** Paratype. **Occurrence:** occurrenceDetails: http://janzen.sas.upenn.edu; catalogNumber: DHJPAR0055866; recordedBy: D.H. Janzen, W. Hallwachs & Gloria Sihezar; individualID: DHJPAR0055866; individualCount: 1; sex: F; lifeStage: adult; preparations: pinned; otherCatalogNumbers: ASHYH2598-14, 14-SRNP-2839, BOLD:AAZ2421; **Taxon:** scientificName: Telothyria peltata; phylum: Arthropoda; class: Insecta; order: Diptera; family: Tachinidae; genus: Telothyria; specificEpithet: peltata; scientificNameAuthorship: Fleming & Wood, 2018; **Location:** continent: Central America; country: Costa Rica; countryCode: CR; stateProvince: Alajuela; county: Sector Rincon Rain Forest; locality: Area de Conservacion Guanacaste; verbatimLocality: Camino Albergue Oscar; verbatimElevation: 560; verbatimLatitude: 10.8774; verbatimLongitude: -85.3236; verbatimCoordinateSystem: Decimal; decimalLatitude: 10.8774; decimalLongitude: -85.3236; **Identification:** identifiedBy: AJ Fleming; dateIdentified: 2018; **Event:** samplingProtocol: Reared from the larva of the Crambidae, Herpetogramma Janzen07; verbatimEventDate: 29-Jun-2014; **Record Level:** language: en; institutionCode: CNC; collectionCode: Insects; basisOfRecord: Pinned Specimen
- **Type status:** Paratype. **Occurrence:** occurrenceDetails: http://janzen.sas.upenn.edu; catalogNumber: DHJPAR0010228; recordedBy: D.H. Janzen, W. Hallwachs & Jose Perez; individualID: DHJPAR0010228; individualCount: 1; sex: F; lifeStage: adult; preparations: pinned; otherCatalogNumbers: ASTAV754-06, 06-SRNP-41511,; **Taxon:** scientificName: Telothyria peltata; phylum: Arthropoda; class: Insecta; order: Diptera; family: Tachinidae; genus: Telothyria; specificEpithet: peltata; scientificNameAuthorship: Fleming & Wood, 2018; **Location:** continent: Central America; country: Costa Rica; countryCode: CR; stateProvince: Alajuela; county: Sector Rincon Rain Forest; locality: Area de Conservacion Guanacaste; verbatimLocality: Finca Aurita; verbatimElevation: 460; verbatimLatitude: 10.8841; verbatimLongitude: -85.2573; verbatimCoordinateSystem: Decimal; decimalLatitude: 10.8841; decimalLongitude: -85.2573; **Identification:** identifiedBy: AJ Fleming; dateIdentified: 2018; **Event:** samplingProtocol: Reared from the larva of the Crambidae, Herpetogramma Janzen07; verbatimEventDate: 01-Jun-2006; **Record Level:** language: en; institutionCode: CNC; collectionCode: Insects; basisOfRecord: Pinned Specimen
- **Type status:** Paratype. **Occurrence:** occurrenceDetails: http://janzen.sas.upenn.edu; catalogNumber: DHJPAR0046467; recordedBy: D.H. Janzen, W. Hallwachs & Keiner Aragon; individualID: DHJPAR0046467; individualCount: 1; sex: F; lifeStage: adult; preparations: pinned; otherCatalogNumbers: ACGBA640-12, 11-SRNP-68225, BOLD:AAF0519; **Taxon:** scientificName: Telothyria peltata; phylum: Arthropoda; class: Insecta; order: Diptera; family: Tachinidae; genus: Telothyria; specificEpithet: peltata; scientificNameAuthorship: Fleming & Wood, 2018; **Location:** continent: Central America; country: Costa Rica; countryCode: CR; stateProvince: Alajuela; county: Sector Rincon Rain Forest; locality: Area de Conservacion Guanacaste; verbatimLocality: Palomo; verbatimElevation: 96; verbatimLatitude: 10.9619; verbatimLongitude: -85.2804; verbatimCoordinateSystem: Decimal; decimalLatitude: 10.9619; decimalLongitude: -85.2804; **Identification:** identifiedBy: AJ Fleming; dateIdentified: 2018; **Event:** samplingProtocol: Reared from the larva of the Crambidae, Herpetogramma Janzen07; verbatimEventDate: 14-Jan-2012; **Record Level:** language: en; institutionCode: CNC; collectionCode: Insects; basisOfRecord: Pinned Specimen
- **Type status:** Paratype. **Occurrence:** occurrenceDetails: http://janzen.sas.upenn.edu; catalogNumber: DHJPAR0037498; recordedBy: D.H. Janzen, W. Hallwachs & Elda Araya; individualID: DHJPAR0037498; individualCount: 1; sex: M; lifeStage: adult; preparations: pinned; otherCatalogNumbers: ASHYC4243-10, 09-SRNP-7111, BOLD:AAZ2421; **Taxon:** scientificName: Telothyria peltata; phylum: Arthropoda; class: Insecta; order: Diptera; family: Tachinidae; genus: Telothyria; specificEpithet: peltata; scientificNameAuthorship: Fleming & Wood, 2018; **Location:** continent: Central America; country: Costa Rica; countryCode: CR; stateProvince: Alajuela; county: Sector San Cristobal; locality: Area de Conservacion Guanacaste; verbatimLocality: Puente Palma; verbatimElevation: 460; verbatimLatitude: 10.9163; verbatimLongitude: -85.3787; verbatimCoordinateSystem: Decimal; decimalLatitude: 10.9163; decimalLongitude: -85.3787; **Identification:** identifiedBy: AJ Fleming; dateIdentified: 2018; **Event:** samplingProtocol: Reared from the larva of the Crambidae, Herpetogramma Janzen07; verbatimEventDate: 01-Feb-2010; **Record Level:** language: en; institutionCode: CNC; collectionCode: Insects; basisOfRecord: Pinned Specimen
- **Type status:** Paratype. **Occurrence:** occurrenceDetails: http://janzen.sas.upenn.edu; catalogNumber: DHJPAR0040931; recordedBy: D.H. Janzen, W. Hallwachs & Anabelle Cordoba; individualID: DHJPAR0040931; individualCount: 1; sex: M; lifeStage: adult; preparations: pinned; otherCatalogNumbers: ASHYF846-11, 10-SRNP-43830,; **Taxon:** scientificName: Telothyria peltata; phylum: Arthropoda; class: Insecta; order: Diptera; family: Tachinidae; genus: Telothyria; specificEpithet: peltata; scientificNameAuthorship: Fleming & Wood, 2018; **Location:** continent: Central America; country: Costa Rica; countryCode: CR; stateProvince: Alajuela; county: Sector Rincon Rain Forest; locality: Area de Conservacion Guanacaste; verbatimLocality: San Lucas; verbatimElevation: 320; verbatimLatitude: 10.9185; verbatimLongitude: -85.3034; verbatimCoordinateSystem: Decimal; decimalLatitude: 10.9185; decimalLongitude: -85.3034; **Identification:** identifiedBy: AJ Fleming; dateIdentified: 2018; **Event:** samplingProtocol: Reared from the larva of the Crambidae, Herpetogramma Janzen07; verbatimEventDate: 22-Nov-2010; **Record Level:** language: en; institutionCode: CNC; collectionCode: Insects; basisOfRecord: Pinned Specimen

#### Description

**Male.** Length: 5–9 mm (Fig. 39). **Head** (Fig. 39b): frons narrow, 1/6 of head width; gena 1/9 of head height; three reclinate orbital setae; anteriormost reclinate orbital one 1/2 times longer than uppermost frontal seta; ocellar setae present but so small as to appear absent; outer vertical seta absent; fronto-orbital plate gold on uppermost 30%, ocellar triangle concolorous and contiguous with gold vertex; fronto-orbital plate with short blonde hairs interspersed among frontal setae; parafacial brilliant silver; facial ridge bare; palpus digitiform, sparsely haired along outer margin; arista orange-brown plumose, distinctly-thickened on basal 1/10, microtrichia at most 3X as long as width of arista; postpedicel orange over at most 50% of surface; postocular region behind margin of eye upper half gold, with lower half including gena silver tomentose; upper half of occiput gold tomentose. **Thorax** (Fig. 39a, c): golden tomentose, with four distinct dorsal stripes; thorax covered in dense plumose blonde hairs laterally, with blonde non-plumose hairs dorsally; chaetotaxy: 4–5 postpronotal setae, basal setae arranged in a straight line; supra-alar setae 2:3; intra-alar setae 1–2:3; dorsocentral setae 3:3; acrostichal setae 3:3; katepisternum with two setae. Scutellum golden tomentose; two pairs of strong marginal setae (basal and subapical) and a small pair of crossed apical scutellar setae 1/5th as long as subapical scutellars; basal scutellar setae subequal in length to subapical setae; subapical setae straight to slightly divergent; underside of scutellum bearing regular non-plumose blonde hairs below basal scutellar setae. **Legs**: foreleg yellow ground color, midleg yellow coxa and half of femur yellow, remainder of femur and tarsi and tibia brown, hindleg dark brown extending from halfway along femur to tarsi; anterior leg tibia with regular fringe of short equally spaced setae along upper half of anteroventral surface, with one posterodorsal setae. **Wings**: basicosta ivory white; all veins bare, with only 1–2 setulae at base of R_4+5_; calypters pale white translucent with a pale yellow fringe. **Abdomen** (Fig. 39a, c): ground color yellow-orange; ST1+2 brown over medial 30%, with yellow ventrolaterally, extending into a longitudinal middorsal brown stripe up to posterior edge of T4; T3 and T4 with dense gold tomentum along anterior marginal 10%, thinning and extending over remainder of tergite; T5 entirely orange with gold tomentum and a basal medial brown triangle; median marginal setae present only on T4 and T5; median discal setae absent. **Male terminalia** (Fig. 39d, e, f): Sternite 5 with a narrow and shallow almost slit-like median cleft, lobes almost touching at midline, margins lightly pollinose; lateral lobes of sternite rounded apically, with 3–4 short weak setulae; basal section of sternite 5 subequal to slightly longer than length of apical lobes, median cleft only 1/4 length of apical section. Cerci in posterior view sharply pointed triangular slightly widening midlength to a slight rectangular shoulder along the basal section, equal in length to surstyli, fused along entire length; in lateral view, with a strong downward curve on apical 1/3; several strong widely spaced setulae along basal 2/3rds. Surstylus in lateral view, almost equilateral along its length rounded at tip, slightly pinched at midpoint appearing digitiform or clubbed; surstylus appearing fused with epandrium; when viewed dorsally surstyli appear straight, almost parallel at apices. Distiphallus 1.5X as long as basiphallus and tubular, slightly pointed at apex.

**Female.** Length: 5–7 mm (Fig. 40). **Head** (Fig. 40b): as in male with the following exceptions: fronto-orbital plate 30% gold; parafacial brilliant silver; frons 1/4 of head width; two inner reclinate orbital setae; two proclinate orbital setae; outer vertical seta present. **Thorax** (Fig. 40a, c): katepisternum with two setae; meron without any plumose hairs only 5–6 typical meral setae. Legs: foreleg yellow ground color, midleg yellow ground color on coxa, with brown overtones on femur, tarsi and tibia, hindleg with yellow basally darkening to brown apically up to dark brown tarsi; anterior leg tibia with regular fringe of short irregularly spaced setae along upper half of anteroventral surface, with 1–2 posterodorsal setae. **Abdomen** (Fig. 40a, c): ground color brown dorsally on ST1+2 and T3 with orange laterally; T4 entirely brown ground color; T5 as in male.

#### Diagnosis

*Telothyria peltata* **sp. n.** can be distinguished from all other *Telothyria* by the following combination of traits: ocellar setae minimal but present, postpedicel mostly maroon-black with orange only directly adjacent to pedicel, plumose hairs absent from thorax dorsally, katepisternum with two setae, and T5 yellow with silver tomentum, underside of scutellum bearing regular non-plumose blonde hairs below basal scutellar setae. Differs from *T. incisa* in the male terminalia where sternite 5 has an almost slit-like median cleft, lobes almost touching at midline, 1/4 length of anterior plate section of sternite 5.

#### Etymology

*Telothyria peltata* **sp. n.** From the Greek noun “*pelta*”, meaning "small shield", in reference to the shield-like shape of sternite 5.

#### Distribution

Costa Rica, ACG, Alajuela and Guanacaste Provinces, 460–560 m elevation.

#### Ecology

*Telothyria peltata* **sp. n.** has been reared 12 times from one species of Lepidoptera in the family Crambidae: *Herpetogramma* Janzen07 in rain forest.

### Telothyria relicta

van der Wulp, 1890

87B9EA83-6BD7-5CE6-986B-C3B69E497D99

Telothyria relicta van der Wulp, 1890: 171. Holotype female (BMNH). Type locality: Mexico, Veracruz, Atoyac.

#### Materials

- **Type status:** Other material. **Occurrence:** occurrenceDetails: http://janzen.sas.upenn.edu; catalogNumber: DHJPAR0006608; recordedBy: D.H. Janzen, W. Hallwachs & Elieth Cantillano; individualID: DHJPAR0006608; individualCount: 1; sex: F; lifeStage: adult; preparations: pinned; otherCatalogNumbers: ASTA787-06, 05-SRNP-24705, BOLD:AAG0819; **Taxon:** scientificName: Telothyria relicta; phylum: Arthropoda; class: Insecta; order: Diptera; family: Tachinidae; genus: Telothyria; specificEpithet: relicta; scientificNameAuthorship: van der Wulp, 1890; **Location:** continent: Central America; country: Costa Rica; countryCode: CR; stateProvince: Guanacaste; county: Sector Del Oro; locality: Area de Conservacion Guanacaste; verbatimLocality: Quebrada Lajosa; verbatimElevation: 400; verbatimLatitude: 11.0331; verbatimLongitude: -85.4288; verbatimCoordinateSystem: Decimal; decimalLatitude: 11.0331; decimalLongitude: -85.4288; **Identification:** identifiedBy: AJ Fleming; dateIdentified: 2018; **Event:** samplingProtocol: Reared from the larva of the Crambidae, Desmia tages; verbatimEventDate: 15-Nov-2005; **Record Level:** language: en; institutionCode: CNC; collectionCode: Insects; basisOfRecord: Pinned Specimen
- **Type status:** Other material. **Occurrence:** occurrenceDetails: http://janzen.sas.upenn.edu; catalogNumber: DHJPAR0039296; recordedBy: D.H. Janzen, W. Hallwachs & Gloria Sihezar; individualID: DHJPAR0039296; individualCount: 1; sex: M; lifeStage: adult; preparations: pinned; otherCatalogNumbers: ASTAV859-10, 10-SRNP-2636, BOLD:AAG0819; **Taxon:** scientificName: Telothyria relicta; phylum: Arthropoda; class: Insecta; order: Diptera; family: Tachinidae; genus: Telothyria; specificEpithet: relicta; scientificNameAuthorship: van der Wulp, 1890; **Location:** continent: Central America; country: Costa Rica; countryCode: CR; stateProvince: Alajuela; county: Sector Rincon Rain Forest; locality: Area de Conservacion Guanacaste; verbatimLocality: Camino Albergue Oscar; verbatimElevation: 560; verbatimLatitude: 10.8774; verbatimLongitude: -85.3236; verbatimCoordinateSystem: Decimal; decimalLatitude: 10.8774; decimalLongitude: -85.3236; **Identification:** identifiedBy: AJ Fleming; dateIdentified: 2018; **Event:** samplingProtocol: Reared from the larva of the Crambidae, Desmia benealisDHJ03; verbatimEventDate: 13-Jun-2010; **Record Level:** language: en; institutionCode: CNC; collectionCode: Insects; basisOfRecord: Pinned Specimen
- **Type status:** Other material. **Occurrence:** occurrenceDetails: http://janzen.sas.upenn.edu; catalogNumber: DHJPAR0042608; recordedBy: D.H. Janzen, W. Hallwachs & Elda Araya; individualID: DHJPAR0042608; individualCount: 1; sex: F; lifeStage: adult; preparations: pinned; otherCatalogNumbers: ASHYH366-11, 11-SRNP-2016, BOLD:AAG0819; **Taxon:** scientificName: Telothyria relicta; phylum: Arthropoda; class: Insecta; order: Diptera; family: Tachinidae; genus: Telothyria; specificEpithet: relicta; scientificNameAuthorship: van der Wulp, 1890; **Location:** continent: Central America; country: Costa Rica; countryCode: CR; stateProvince: Alajuela; county: Sector Rincon Rain Forest; locality: Area de Conservacion Guanacaste; verbatimLocality: Camino Albergue Oscar; verbatimElevation: 560; verbatimLatitude: 10.8774; verbatimLongitude: -85.3236; verbatimCoordinateSystem: Decimal; decimalLatitude: 10.8774; decimalLongitude: -85.3236; **Identification:** identifiedBy: AJ Fleming; dateIdentified: 2018; **Event:** samplingProtocol: Reared from the larva of the Crambidae, Desmia benealisDHJ03; verbatimEventDate: 08-Jun-2011; **Record Level:** language: en; institutionCode: CNC; collectionCode: Insects; basisOfRecord: Pinned Specimen
- **Type status:** Other material. **Occurrence:** occurrenceDetails: http://janzen.sas.upenn.edu; catalogNumber: DHJPAR0052069; recordedBy: D.H. Janzen, W. Hallwachs & Osvaldo Espinoza; individualID: DHJPAR0052069; individualCount: 1; sex: F; lifeStage: adult; preparations: pinned; otherCatalogNumbers: ASHYH1181-13, 13-SRNP-2909, BOLD:AAG0819; **Taxon:** scientificName: Telothyria relicta; phylum: Arthropoda; class: Insecta; order: Diptera; family: Tachinidae; genus: Telothyria; specificEpithet: relicta; scientificNameAuthorship: van der Wulp, 1890; **Location:** continent: Central America; country: Costa Rica; countryCode: CR; stateProvince: Alajuela; county: Sector San Cristobal; locality: Area de Conservacion Guanacaste; verbatimLocality: Sendero Huerta; verbatimElevation: 527; verbatimLatitude: 10.9305; verbatimLongitude: -85.3722; verbatimCoordinateSystem: Decimal; decimalLatitude: 10.9305; decimalLongitude: -85.3722; **Identification:** identifiedBy: AJ Fleming; dateIdentified: 2018; **Event:** samplingProtocol: Reared from the larva of the Crambidae, Desmia benealisDHJ03; verbatimEventDate: 27-Jun-2013; **Record Level:** language: en; institutionCode: CNC; collectionCode: Insects; basisOfRecord: Pinned Specimen
- **Type status:** Other material. **Occurrence:** occurrenceDetails: http://janzen.sas.upenn.edu; catalogNumber: DHJPAR0050575; recordedBy: D.H. Janzen, W. Hallwachs & Cirilo Umana; individualID: DHJPAR0050575; individualCount: 1; sex: M; lifeStage: adult; preparations: pinned; otherCatalogNumbers: ACGBA3167-13, 12-SRNP-77019, BOLD:AAG0819; **Taxon:** scientificName: Telothyria relicta; phylum: Arthropoda; class: Insecta; order: Diptera; family: Tachinidae; genus: Telothyria; specificEpithet: relicta; scientificNameAuthorship: van der Wulp, 1890; **Location:** continent: Central America; country: Costa Rica; countryCode: CR; stateProvince: Alajuela; county: Sector Rincon Rain Forest; locality: Area de Conservacion Guanacaste; verbatimLocality: Finca Esmeralda; verbatimElevation: 123; verbatimLatitude: 10.9355; verbatimLongitude: -85.2531; verbatimCoordinateSystem: Decimal; decimalLatitude: 10.9355; decimalLongitude: -85.2531; **Identification:** identifiedBy: AJ Fleming; dateIdentified: 2018; **Event:** samplingProtocol: Reared from the larva of the Crambidae, Desmia benealisDHJ03; verbatimEventDate: 19-Oct-2012; **Record Level:** language: en; institutionCode: CNC; collectionCode: Insects; basisOfRecord: Pinned Specimen
- **Type status:** Other material. **Occurrence:** occurrenceDetails: http://janzen.sas.upenn.edu; catalogNumber: DHJPAR0050630; recordedBy: D.H. Janzen, W. Hallwachs & Mercedes Moraga; individualID: DHJPAR0050630; individualCount: 1; sex: F; lifeStage: adult; preparations: pinned; otherCatalogNumbers: ACGBA3222-13, 12-SRNP-77646, BOLD:AAG0819; **Taxon:** scientificName: Telothyria relicta; phylum: Arthropoda; class: Insecta; order: Diptera; family: Tachinidae; genus: Telothyria; specificEpithet: relicta; scientificNameAuthorship: van der Wulp, 1890; **Location:** continent: Central America; country: Costa Rica; countryCode: CR; stateProvince: Alajuela; county: Sector Rincon Rain Forest; locality: Area de Conservacion Guanacaste; verbatimLocality: Finca Esmeralda; verbatimElevation: 123; verbatimLatitude: 10.9355; verbatimLongitude: -85.2531; verbatimCoordinateSystem: Decimal; decimalLatitude: 10.9355; decimalLongitude: -85.2531; **Identification:** identifiedBy: AJ Fleming; dateIdentified: 2018; **Event:** samplingProtocol: Reared from the larva of the Crambidae, Desmia benealisDHJ03; verbatimEventDate: 01-Dec-2012; **Record Level:** language: en; institutionCode: CNC; collectionCode: Insects; basisOfRecord: Pinned Specimen
- **Type status:** Other material. **Occurrence:** occurrenceDetails: http://janzen.sas.upenn.edu; catalogNumber: DHJPAR0051641; recordedBy: D.H. Janzen, W. Hallwachs & Gloria Sihezar; individualID: DHJPAR0051641; individualCount: 1; sex: F; lifeStage: adult; preparations: pinned; otherCatalogNumbers: ACGBA4233-13, 13-SRNP-758, BOLD:AAG0819; **Taxon:** scientificName: Telothyria relicta; phylum: Arthropoda; class: Insecta; order: Diptera; family: Tachinidae; genus: Telothyria; specificEpithet: relicta; scientificNameAuthorship: van der Wulp, 1890; **Location:** continent: Central America; country: Costa Rica; countryCode: CR; stateProvince: Alajuela; county: Sector San Cristobal; locality: Area de Conservacion Guanacaste; verbatimLocality: Sendero Huerta; verbatimElevation: 527; verbatimLatitude: 10.9305; verbatimLongitude: -85.3722; verbatimCoordinateSystem: Decimal; decimalLatitude: 10.9305; decimalLongitude: -85.3722; **Identification:** identifiedBy: AJ Fleming; dateIdentified: 2018; **Event:** samplingProtocol: Reared from the larva of the Crambidae, Desmia ploralisDHJ10; verbatimEventDate: 11-Mar-2013; **Record Level:** language: en; institutionCode: CNC; collectionCode: Insects; basisOfRecord: Pinned Specimen
- **Type status:** Other material. **Occurrence:** occurrenceDetails: http://janzen.sas.upenn.edu; catalogNumber: DHJPAR0062393; recordedBy: D.H. Janzen, W. Hallwachs & Cirilo Umana; individualID: DHJPAR0062393; individualCount: 1; sex: F; lifeStage: adult; preparations: pinned; otherCatalogNumbers: ACGBA8697-18, 17-SRNP-76419, BOLD:AAG0819; **Taxon:** scientificName: Telothyria relicta; phylum: Arthropoda; class: Insecta; order: Diptera; family: Tachinidae; genus: Telothyria; specificEpithet: relicta; scientificNameAuthorship: van der Wulp, 1890; **Location:** continent: Central America; country: Costa Rica; countryCode: CR; stateProvince: Alajuela; county: Sector Rincon Rain Forest; locality: Area de Conservacion Guanacaste; verbatimLocality: Quebrada Bambu; verbatimElevation: 109; verbatimLatitude: 10.9301; verbatimLongitude: -85.2521; verbatimCoordinateSystem: Decimal; decimalLatitude: 10.9301; decimalLongitude: -85.2521; **Identification:** identifiedBy: AJ Fleming; dateIdentified: 2018; **Event:** samplingProtocol: Reared from the larva of the Crambidae, same as 04-SRNP-56093; verbatimEventDate: 07-Dec-2017; **Record Level:** language: en; institutionCode: CNC; collectionCode: Insects; basisOfRecord: Pinned Specimen

#### Description

**Male.** Length: 7–10 mm (Fig. 41). **Head** (Fig. 41b): frons narrow, 1/6 of head width; gena 1/8 of head height; two reclinate orbital setae; anteriormost reclinate orbital four times longer than uppermost frontal seta; ocellar setae present but so small as to appear absent; outer vertical seta absent; fronto-orbital plate brassy gold, ocellar triangle slightly darker than vertex; fronto-orbital plate with short blonde hairs interspersed among frontal setae; parafacial pale, with a very light silver tomentosity visible under certain angles; facial ridge bare; palpus with a slight spatulate club at apex, sparsely haired along outer margin; arista orange-brown smoothly tapered, microtrichia at most as long as width of arista; postpedicel orange over more than 50% of surface; postocular region behind margin of eye upper 3/4 gold, with lower 1/4 including gena silver tomentose; upper half of occiput gold tomentose. **Thorax** (Fig. 41a, c): golden tomentose, with four distinct dorsal stripes; entire thorax covered in dense plumose blonde hairs; chaetotaxy: 6–7 postpronotal setae, basal setae arranged in a straight line; supra-alar setae 2:3; intra-alar setae 2:3; dorsocentral setae 3:3; acrostichal setae 3:3; katepisternum with two setae. Scutellum ranging from golden tomentose; two pairs of strong marginal setae (basal and subapical) and a small pair of crossed apical scutellar setae 1/8–1/10th as long as subapical scutellars; basal scutellar setae subequal in length to subapical setae; subapical setae straight; underside of scutellum bearing plumose blonde hairs below basal scutellar setae. **Legs**: foreleg with yellow ground color throughout, tibia and tarsal segments appearing darker due to hair covering; midleg with yellow coxa and femur, tibia light yellow-brown appearing darker due to hair covering and dark brown tarsal segments; hindleg with yellow coxa and proximal 1/3 of femur, almost entirely dark brown extending from distal 2/3 of femur to tarsal segments; anterior leg tibia with regular fringe of equally spaced setae along anteroventral surface, with two strong posterodorsal setae. **Wings**: basicosta pale ivory, darkening to brown basally; dorsally with 1–2 setulae at base of R_4+5_; calypters pale white translucent, fringe only slightly more opaque than remainder of calypter. **Abdomen** (Fig. 41a, c): ground color yellow-orange; ST1+2 brown over 50%, with yellow ventrolaterally, extending into a longitudinal middorsal brown stripe up to posterior edge of T4; T3 and T4 each with dense gold tomentum along anterior marginal 10%, thinning and extending over remainder of tergite; T5 entirely orange with gold tomentum; median marginal setae present only on T4 and T5; median discal setae absent. **Male terminalia** (Fig. 41d, e, f): Sternite 5 with a wide deeply excavated median cleft, smoothly V-shaped, margins covered in dense pollinosity; lateral lobes of sternite pointed apically, with a small group of strong setulae along outer margins; basal section of sternite 5 subequal to slightly shorter than length of apical lobes. Cerci in posterior view sharply pointed and slender, abruptly widening to a moderate almost rectangular shoulder along the basal section, equal in length to surstyli, fused along entire length; in lateral view, with a smooth regular downward curve along apical 2/3rds; several strong widely spaced setulae along basal 1/3rd. Surstylus in lateral view, almost equilateral along its length rounded at tip, digitiform; surstylus appearing fused with epandrium; when viewed dorsally surstyli appear slender with an inward bend. Distiphallus 2X as long as basiphallus and tubular, slightly pointed at apex.

**Female.** Length: 5–9 mm (Fig. 42). **Head** (Fig. 42b): as in male with the following exceptions: fronto-orbital plate 50% gold; parafacial brilliant silver; frons 1/5 of head width; three inner reclinate orbital setae; two proclinate orbital setae; outer vertical seta present; postocular region behind margin of eye upper 1/4 gold, with lower 3/4 including gena silver tomentose; upper half of occiput gold tomentose. **Thorax** (Fig. 42a, c): katepisternum with three setae; meron densely covered in plumose hairs as in male but with the addition of 2–4 typical meral setae. Legs: foreleg with yellow ground color throughout; midleg with yellow coxa and proximal 1/5 of femur with brown tibia and tarsal segments; hindleg with yellow coxa, proximal 1/3 of femur yellow and remainder almost entirely dark brown extending from femur to tarsal segments. Wings: as in male except ventrally R_4+5_ bearing 2–7 setae. **Abdomen** (Fig. 42a, c): ground color brown on ST1+2 and T3 with small patches of orange laterally; T4 entirely brown ground color; T5 entirely orange with gold tomentum; T3 with one pair of median marginal setae and T4, T5 with a complete row of marginal setae.

#### Diagnosis

*Telothyria relicta* can be distinguished from all other *Telothyria* by the following combination of traits: ocellar setae present but so small as to appear absent, postpedicel mostly orange, parafacial gold, entire thorax covered in dense plumose blonde hairs, katepisternum with two setae, underside of scutellum bearing plumose blonde hairs below basal scutellar setae, legs yellow, abdominal ground color yellow-orange, and T5 entirely orange with gold tomentum.

#### Distribution

Costa Rica, ACG, Alajuela Province, 123–560 m elevation.

#### Ecology

Within the ACG inventor, *Telothyria relicta* has been reared seven times from three species of Lepidoptera in the family Crambidae: *Desmia tages* (Cramer, 1777), *Desmia benealis*DHJ03, and *Desmia ploralis*DHJ10 in rain forest and dry–rain lowland intergrade.

### Telothyria ricardocaleroi

Fleming & Wood sp. n.

930CB975-7D5E-5ED6-894B-D6082E16BB1C

urn:lsid:zoobank.org:act:5F6A0320-560D-4B31-8D13-37587E049F3E

#### Materials

- **Type status:** Holotype. **Occurrence:** occurrenceDetails: http://janzen.sas.upenn.edu; catalogNumber: DHJPAR0054151; recordedBy: D.H. Janzen, W. Hallwachs & Anabelle Cordoba; individualID: DHJPAR0054151; individualCount: 1; sex: M; lifeStage: adult; preparations: pinned; otherCatalogNumbers: 13-SRNP-47166, BOLD:ACJ2245; **Taxon:** scientificName: Telothyria ricardocaleroi; phylum: Arthropoda; class: Insecta; order: Diptera; family: Tachinidae; genus: Telothyria; specificEpithet: ricardocaleroi; scientificNameAuthorship: Fleming & Wood, 2018; **Location:** continent: Central America; country: Costa Rica; countryCode: CR; stateProvince: Alajuela; county: Sector Rincon Rain Forest; locality: Area de Conservacion Guanacaste; verbatimLocality: Sendero Juntas; verbatimElevation: 400; verbatimLatitude: 10.906610; verbatimLongitude: -85.287840; verbatimCoordinateSystem: Decimal; decimalLatitude: 10.90661; decimalLongitude: -85.28784; **Identification:** identifiedBy: AJ Fleming; dateIdentified: 2018; **Event:** samplingProtocol: Reared from the larva of the Crambidae, Neoleucinodes Janzen02; verbatimEventDate: 20-Jan-2014; **Record Level:** language: en; institutionCode: CNC; collectionCode: Insects; basisOfRecord: Pinned Specimen
- **Type status:** Paratype. **Occurrence:** occurrenceDetails: http://janzen.sas.upenn.edu; catalogNumber: DHJPAR0050686; recordedBy: D.H. Janzen, W. Hallwachs & Anabelle Cordoba; individualID: DHJPAR0050686; individualCount: 1; sex: F; lifeStage: adult; preparations: pinned; otherCatalogNumbers: ACGBA3278-13, 12-SRNP-86017, BOLD:ACJ2245; **Taxon:** scientificName: Telothyria ricardocaleroi; phylum: Arthropoda; class: Insecta; order: Diptera; family: Tachinidae; genus: Telothyria; specificEpithet: ricardocaleroi; scientificNameAuthorship: Fleming & Wood, 2018; **Location:** continent: Central America; country: Costa Rica; countryCode: CR; stateProvince: Alajuela; county: Sector Rincon Rain Forest; locality: Area de Conservacion Guanacaste; verbatimLocality: Sendero Juntas; verbatimElevation: 400; verbatimLatitude: 10.9066; verbatimLongitude: -85.2878; verbatimCoordinateSystem: Decimal; decimalLatitude: 10.9066; decimalLongitude: -85.2878; **Identification:** identifiedBy: AJ Fleming; dateIdentified: 2018; **Event:** samplingProtocol: Reared from the larva of the Crambidae, Neoleucinodes Janzen02; verbatimEventDate: 08-Nov-2012; **Record Level:** language: en; institutionCode: CNC; collectionCode: Insects; basisOfRecord: Pinned Specimen
- **Type status:** Paratype. **Occurrence:** occurrenceDetails: http://janzen.sas.upenn.edu; catalogNumber: DHJPAR0057064; recordedBy: D.H. Janzen, W. Hallwachs & Pablo Umana Calderon; individualID: DHJPAR0057064; individualCount: 1; sex: F; lifeStage: adult; preparations: pinned; otherCatalogNumbers: ACGBA4974-15, 13-SRNP-47125, BOLD:ACJ2245; **Taxon:** scientificName: Telothyria ricardocaleroi; phylum: Arthropoda; class: Insecta; order: Diptera; family: Tachinidae; genus: Telothyria; specificEpithet: ricardocaleroi; scientificNameAuthorship: Fleming & Wood, 2018; **Location:** continent: Central America; country: Costa Rica; countryCode: CR; stateProvince: Alajuela; county: Sector Rincon Rain Forest; locality: Area de Conservacion Guanacaste; verbatimLocality: Sendero Juntas; verbatimElevation: 400; verbatimLatitude: 10.9066; verbatimLongitude: -85.2878; verbatimCoordinateSystem: Decimal; decimalLatitude: 10.9066; decimalLongitude: -85.2878; **Identification:** identifiedBy: AJ Fleming; dateIdentified: 2018; **Event:** samplingProtocol: Reared from the larva of the Crambidae, Neoleucinodes Janzen02; verbatimEventDate: 19-Jan-2014; **Record Level:** language: en; institutionCode: CNC; collectionCode: Insects; basisOfRecord: Pinned Specimen
- **Type status:** Paratype. **Occurrence:** occurrenceDetails: http://janzen.sas.upenn.edu; catalogNumber: DHJPAR0057087; recordedBy: D.H. Janzen, W. Hallwachs & Pablo Umana Calderon; individualID: DHJPAR0057087; individualCount: 1; sex: M; lifeStage: adult; preparations: pinned; otherCatalogNumbers: ACGBA4997-15, 14-SRNP-67339, BOLD:ACJ2245; **Taxon:** scientificName: Telothyria ricardocaleroi; phylum: Arthropoda; class: Insecta; order: Diptera; family: Tachinidae; genus: Telothyria; specificEpithet: ricardocaleroi; scientificNameAuthorship: Fleming & Wood, 2018; **Location:** continent: Central America; country: Costa Rica; countryCode: CR; stateProvince: Alajuela; county: Sector Rincon Rain Forest; locality: Area de Conservacion Guanacaste; verbatimLocality: Vado Rio Francia; verbatimElevation: 400; verbatimLatitude: 10.9009; verbatimLongitude: -85.2891; verbatimCoordinateSystem: Decimal; decimalLatitude: 10.9009; decimalLongitude: -85.2891; **Identification:** identifiedBy: AJ Fleming; dateIdentified: 2018; **Event:** samplingProtocol: Reared from the larva of the Crambidae, Neoleucinodes Janzen02; verbatimEventDate: 22-Jan-2015; **Record Level:** language: en; institutionCode: CNC; collectionCode: Insects; basisOfRecord: Pinned Specimen
- **Type status:** Paratype. **Occurrence:** occurrenceDetails: http://janzen.sas.upenn.edu; catalogNumber: DHJPAR0057089; recordedBy: D.H. Janzen, W. Hallwachs & Jose Perez; individualID: DHJPAR0057089; individualCount: 1; sex: M; lifeStage: adult; preparations: pinned; otherCatalogNumbers: ACGBA4999-15, 14-SRNP-67353, BOLD:ACJ2245; **Taxon:** scientificName: Telothyria ricardocaleroi; phylum: Arthropoda; class: Insecta; order: Diptera; family: Tachinidae; genus: Telothyria; specificEpithet: ricardocaleroi; scientificNameAuthorship: Fleming & Wood, 2018; **Location:** continent: Central America; country: Costa Rica; countryCode: CR; stateProvince: Alajuela; county: Sector Rincon Rain Forest; locality: Area de Conservacion Guanacaste; verbatimLocality: Vado Rio Francia; verbatimElevation: 400; verbatimLatitude: 10.9009; verbatimLongitude: -85.2891; verbatimCoordinateSystem: Decimal; decimalLatitude: 10.9009; decimalLongitude: -85.2891; **Identification:** identifiedBy: AJ Fleming; dateIdentified: 2018; **Event:** samplingProtocol: Reared from the larva of the Crambidae, Neoleucinodes Janzen02; verbatimEventDate: 23-Jan-2015; **Record Level:** language: en; institutionCode: CNC; collectionCode: Insects; basisOfRecord: Pinned Specimen
- **Type status:** Paratype. **Occurrence:** occurrenceDetails: http://janzen.sas.upenn.edu; catalogNumber: DHJPAR0054145; recordedBy: D.H. Janzen, W. Hallwachs & Pablo Umana Calderon; individualID: DHJPAR0054145; individualCount: 1; sex: M; lifeStage: adult; preparations: pinned; otherCatalogNumbers: 13-SRNP-47123, BOLD:ACJ2245; **Taxon:** scientificName: Telothyria ricardocaleroi; phylum: Arthropoda; class: Insecta; order: Diptera; family: Tachinidae; genus: Telothyria; specificEpithet: ricardocaleroi; scientificNameAuthorship: Fleming & Wood, 2018; **Location:** continent: Central America; country: Costa Rica; countryCode: CR; stateProvince: Alajuela; county: Sector Rincon Rain Forest; locality: Area de Conservacion Guanacaste; verbatimLocality: Sendero Juntas; verbatimElevation: 400; verbatimLatitude: 10.906610; verbatimLongitude: -85.287840; verbatimCoordinateSystem: Decimal; decimalLatitude: 10.90661; decimalLongitude: -85.28784; **Identification:** identifiedBy: AJ Fleming; dateIdentified: 2018; **Event:** samplingProtocol: Reared from the larva of the Crambidae, Neoleucinodes Janzen02; verbatimEventDate: 21-Jan-2014; **Record Level:** language: en; institutionCode: CNC; collectionCode: Insects; basisOfRecord: Pinned Specimen
- **Type status:** Paratype. **Occurrence:** occurrenceDetails: http://janzen.sas.upenn.edu; catalogNumber: DHJPAR0050551; recordedBy: D.H. Janzen, W. Hallwachs & Keiner Aragon; individualID: DHJPAR0050551; individualCount: 1; sex: F; lifeStage: adult; preparations: pinned; otherCatalogNumbers: ACGBA3143-13, 12-SRNP-68632, BOLD:ACJ2245; **Taxon:** scientificName: Telothyria ricardocaleroi; phylum: Arthropoda; class: Insecta; order: Diptera; family: Tachinidae; genus: Telothyria; specificEpithet: ricardocaleroi; scientificNameAuthorship: Fleming & Wood, 2018; **Location:** continent: Central America; country: Costa Rica; countryCode: CR; stateProvince: Alajuela; county: Sector Rincon Rain Forest; locality: Area de Conservacion Guanacaste; verbatimLocality: Palomo; verbatimElevation: 96; verbatimLatitude: 10.9619; verbatimLongitude: -85.2804; verbatimCoordinateSystem: Decimal; decimalLatitude: 10.9619; decimalLongitude: -85.2804; **Identification:** identifiedBy: AJ Fleming; dateIdentified: 2018; **Event:** samplingProtocol: Reared from the larva of the Crambidae, Neoleucinodes Janzen02; verbatimEventDate: 06-Nov-2012; **Record Level:** language: en; institutionCode: CNC; collectionCode: Insects; basisOfRecord: Pinned Specimen

#### Description

**Male.** Length: 6 mm (Fig. 43). **Head** (Fig. 43b): frons narrow, 1/5 of head width; gena less than 1/12 of head height; four reclinate orbital setae; anteriormost reclinate orbital shorter than uppermost frontal seta; ocellar setae absent; outer vertical absent; fronto-orbital plate pale silver with a slight brassy-gold tinge at level of ocellar triangle, ocellar triangle concolorous with surrounding fronto-orbital plate; fronto-orbital plate with short blonde hairs interspersed among frontal setae; parafacial brilliant silver; facial ridge bare; palpus narrow and filiform, sparsely haired; arista brown, slight orange tinge basally, smoothly tapering to apical 1/8, microtrichia at most 1X as long as width of arista; postpedicel orange over most of its surface slightly darkening along apical 50%; postocular region behind margin of eye upper 1/2–2/3 gold, with lower 1/2–1/3 including gena silver tomentose; upper half of occiput gold tomentose. **Thorax** (Fig. 43a, c): brassy-gold tomentose, with four distinct dorsal stripes, inner two broken along suture; thorax covered in dense black hairs dorsally, and plumose blonde hairs laterally; chaetotaxy: five postpronotal setae, basal setae arranged in a straight line; supra-alar setae 2:3; intra-alar setae 2:3; dorsocentral setae 3:3; acrostichal setae 4:3; katepisternum with three setae. Scutellum brassy-gold tomentose, darkened basally; two pairs of strong marginal setae (basal and subapical) and a small pair of crossed apical scutellar setae 1/8–1/10th as long as subapical scutellars; basal scutellar setae subequal in length to subapical setae; subapical setae straight; underside of scutellum bearing predominantly regular non-plumose black hairs sometime with few interspersed plumose blonde hairs below basal scutellar setae. **Legs**: foreleg with yellow ground color throughout; midleg and hindleg bearing yellow coxae with dark yellow-brown femur, tibia, and tarsal segments; anterior leg tibia with regular tapered fringe of equally spaced setae along basal half of anteroventral surface, and one strong posterodorsal seta. **Wings**: basicosta pale ivory white; all veins bare, with only one setula at base of R_4+5_; calypters pale white translucent with pale yellow fringes. **Abdomen** (Fig. 43a, c): ground color yellow appearing darkened to brown-black dorsally, with yellow ventrolaterally; entire abdomen covered in dense gold tomentum; T5 entirely black-maroon with only a slightly yellow apex, covered with silver tomentum; median marginal setae present only on T4 and T5, those present on T4 drastically reduced compared to those on T5; median discal setae absent. **Male terminalia** (Fig. 43d, e, f): Sternite 5 with a wide deeply separated median cleft, widely V-shaped, margins tomentose; lateral lobes of sternite subtriangular apically, outer margins covered in strong setae; basal section of sternite 5 2.5X longer than length of apical lobes. Cerci in posterior view sharply pointed triangular, equal in length to surstyli, fused along entire length; basal shoulder weakly developed almost absent. In lateral view with a strong downward curve on apical 1/3; several strong widely spaced setae along basal 2/3rds. Surstylus in lateral view, almost subrectangular along its length rounded at tip, slightly pinched at midpoint appearing digitiform; surstylus appearing fused with epandrium; when viewed dorsally surstyli appear robust and straight with a very slight club apically. Distiphallus subequal to in length to basiphallus, weakly tapering apically.

**Female.** Length: 4–5 mm (Fig. 44). **Head** (Fig. 44b): as in male with the following exceptions: fronto-orbital plate pale brassy gold over upper 70%; frons 1/3 of head width; gena 1/6 of head height; three inner reclinate orbital setae; three proclinate orbital setae; outer vertical seta present; palpus apically oar-shaped and distinctly upturned; postocular region behind margin of eye upper 1/3 gold, with lower 2/3 including gena silver tomentose. **Thorax** (Fig. 44a, c): katepisternum with three setae; meron plumose hairs as well as 6–8 typical meral setae. Legs: anterior leg, with blotchy darkened charcoal-black patches on yellow ground color; midleg and hindleg as in male; anterior tibia with regular tapered fringe of equally spaced setae along basal 1/3 of anteroventral surface, often only 3–4 setae, one almost anterodorsal seta and one strong posterodorsal seta. **Abdomen** (Fig. 44a, c): ST1+2 and T3 50% brown dorsally, with yellow-orange lateroventrally, T4 entirely brown, and T5 yellow-orange entirely.

#### Diagnosis

*Telothyria ricardocaleroi* **sp. n.** can be distinguished from all other *Telothyria* by the following combination of traits: ocellar setae absent, fronto-orbital plate pale silver with a slight brassy-gold tinge at level of ocellar triangle, ocellar triangle concolorous with surrounding fronto-orbital plate, plumose hairs absent from disc of scutum, thorax gold tomentose dorsally, katepisternum with three setae, black setulae on underside of scutellum, legs yellow, abdominal ground color yellow-orange, and T5 black-maroon with silver tomentum.

#### Etymology

*Telothyria ricardocaleroi* **sp. n.** is named in recognition of Ricardo Calero's outstanding work on the team that conducts the caterpillar and parasite inventory from ACG’s Estación Biológica Quica.

#### Distribution

Costa Rica, ACG, Alajuela Province, 96–400 m elevation.

#### Ecology

*Telothyria ricardocaleroi* **sp. n.** has been reared eight times from a single species of Lepidoptera in the family Crambidae: *Neoleucinodes* Janzen02, in rain forest.

## Identification Keys

### Key to the *Telothyria* van der Wulp, of the Mesoamerican region

**Table:** 

1	Plumose blonde hairs present on disc of scutum as well as both lateral and dorsal surfaces of thorax (Fig. 45)	2
–	Plumose blonde hairs absent from disc of scutum (Fig. 46), in abundance laterally on thorax	19
2	Two katepisternal setae	3
–	Three katepisternal setae	8
3	Two postsutural intra-alar setae	4
–	Three postsutural intra-alar setae	6
4	Fore leg reddish-maroon; abdominal tergite 5 (T5) dark brown ground color covered in gold tomentum	*trinitatis* (Thompson)
–	Fore leg yellow; T5 yellow or orange ground color, with or without gold or beige tomentum, that is sparse apicodorsally	5
5	Facial ridge bearing short blonde setulae on lowest 1/5 (Fig. 25); scutellum entirely covered with plumose hairs; basicosta ivory white (Fig. 48b)	* fimbriata * **sp. n.**
–	Facial ridge bare; scutellum with plumose hairs along anterior margins only, disc of scutellum with only short black setulae; basicosta orange (Fig. 48c)	* auranticrus * **sp. n.**
6	Median marginal setae present on ST1+2, and T3; wing infuscate; calypters brassy brown	* bicuspidata * **sp. n.**
–	Median marginal setae absent on ST1+2, and T3; wing clear; calypters white translucent	7
7	Fronto-orbital plate over 90% silver, slightly darkened surrounding ocellar triangle	*manuelpereirai***sp. n.** in part males
–	Fronto-orbital plate brassy-gold, concolorous around ocellar triangle	*relicta* van der Wulp in part males
8	Frons widened, brilliant silver throughout; narrow frontal vitta, almost obliterated by fronto-orbital plate (Fig. 27b)	9
–	Frons narrow, not as above, either brilliant silver or gold; frontal vitta well defined and prominent	10
9	Tibia of fore leg with two posterodorsal setae, and no anteroventral fringe; abdominal ground color orange dorsally with median dark stripe, T5 more than 1/2 orange (Fig. 49a)	*frontalis* (Townsend, 1939)
–	Tibia of fore leg with one posterodorsal seta, and an anteroventral fringe; abdominal ground color dark brown dorsally with yellow laterally, T5 orange only apically (Fig. 49b)	* fulgida * **sp. n.**
10	Median marginal setae present on T3	11
–	Median marginal setae absent on T3	12
11	Abdomen ground color brown on ST1+2 and T3 with small orange patches ventrolaterally; basal 1/3 of hind femur yellow, contrasting with dark brown apical 2/3rds	*relicta* van der Wulp in part female
–	Abdomen ground color brown dorsally on ST1+2 and T3 with large patches of orange ventrolaterally; T5 entirely orange with gold tomentum; proximal 1/2 of femur yellow, basal 1/2 dark brown;	*manuelpereirai***sp. n.** in part female
12	Basicosta either brown or pale orange–beige; ground color of hind femur dark	13
–	Basicosta ivory white; ground color of hind femur partly or entirely yellow	14
13	Ocellar setae present but weak; basicosta creamy orange–beige	* harryramirezi * **sp. n.**
–	Ocellar setae absent; basicosta uniformly dark brown	* duniagarciae * **sp. n.**
14	Two postsutural intra-alar setae	15
–	Three postsutural intra-alar setae	16
15	Four postsutural dorsocentral setae; hind femur over 2/3 yellow	* auriolus * **sp. n.**
–	Three postsutural dorsocentral setae; hind femur up to 1/2 yellow	* erythropyga * **sp. n.**
16	Hind femur with entirely yellow ground color, with no dark coloration	*variegata* (Fabricius)
–	Hind femur yellow ground color basally changing to dark brown on at least apical 1/3	17
17	Postocular region behind margin of eye distinctly silver along upper half of eye; T5 all dark brown with only minimal orange along apical 10% of tergite	*rufostriata* van der Wulp
–	Postocular region behind margin of eye distinctly gold along upper half of eye; T5 mostly orange with brown along basal 10% of tergite	18
18	Fronto-orbital plate deep gold throughout; inner thoracic-vitta reaching up to but not beyond insertion of first postsutural dorsocentral seta; medial 30% of ST1+2 brown, with yellow-orange ventrolaterally, females orange abdomen with dorsomedial brown stripe (Fig. 47b)	* alexanderi * **sp. n.**
–	Fronto-orbital plate pale gold or pale yellow with a sheen; inner thoracic-vitta reaching up to halfway between the insertion of first postsutural and second postsutural dorsocentral setae; medial 50% of ST1+2 brown with yellow-orange ventrolaterally, females brown dorsally with orange ventrolaterally (Fig. 47a)	* aidani * **sp. n.**
19	Basicosta brown or black (Fig. 48a)	20
–	Basicosta ivory or beige	26
20	Thorax with brassy or gold tomentum throughout, including both lateral and dorsal surfaces	21
–	Thorax with distinctly grey or silver tomentum	24
21	Thorax black in ground color with pale brassy tomentum, 5 bold prominent dorsal stripes; thorax with spot of brown plumose hairs laterally on anepisternum; abdomen bright orange, slightly darkened mid-dorsally (Fig. 11a, c)	* clavata * **sp. n.**
–	Thorax black in ground color with gold tomentum, either 4 dorsal stripes or dorsal stripe indistinct except under certain angles of light; plumose hairs of thorax all yellow; abdomen not bright orange	22
22	Basicosta beige apically turning to brown basally, abdomen dark reddish maroon, with distinctly reddish T5, all legs dark reddish-brown ground color	*rufopygata* (Bigot)
–	Basicosta brown throughout, abdomen not dark reddish-brown ground color, legs with light ground color either yellow or reddish orange.	23
23	Ocellar setae lateroclinate; frontal vitta with wide branches surrounding ocellar triangle; ground color of legs reddish orange	*major* (Thompson)
–	Ocellar setae absent; frontal vitta tapering and evanescent around ocellar triangle; legs yellow ground color can appear light brown	*illucens* van der Wulp
24	Ground color of abdomen ST1+2, and T3 mostly orange, bisected by a mid-dorsal brown stripe extending to entirely brown T4 and T5 (Figs 29a, 30a)	* grisea * **sp. n.**
–	Ground color of abdomen mostly maroon lacking any orange dorsally (Fig. 14b, c)	25
25	Ground color of abdomen entirely maroon with no orange present	*cupreiventris* van der Wulp
–	Ground color of abdomen mostly maroon or dark with orange evident along lateral surfaces	* cristata * **sp. n.**
26	Katepisternal setae 2; postpedicel mostly orange (some brown or dark orange may be present) (Figs 32b, 39b)	27
–	Katepisternal setae 3; postpedicel more than 50% black or dark colored with orange only confined to base directly adjacent to pedicel (Figs 20, 38)	28
27	Sternite 5 with a narrow and shallow almost slit-like median cleft, only 1/4 length of apical section; cercus in posterior view slightly widening at midlength to creating a slight rectangular shoulder along basal section (Fig. 39e, f)	* peltata * **sp. n.**
–	Sternite 5 with a narrow and shallow strongly v-shaped median cleft, cleft up to 1/3 length of apical section; cerci in posterior view very narrowly widened with almost no shoulder present along basal 1/3 (Fig. 32e, f)	* incisa * **sp. n.**
28	Abdominal T4 lacking median marginal setae, only two lateral marginal setae present	* omissa * **sp. n.**
–	Abdominal T4 with median marginal setae these can appear short and stout or reduced	29
29	With four wide dorsal stripes, plus one extra presutural stripe dorsomedially; thoracic tomentum mostly bronze-brown with gold along lateral margins of scutum; abdomen brassy brown tomentose throughout	* obscura * **sp. n.**
–	With only four dorsal stripes; thoracic tomentum silver grey or pale gold; abdomen ranging from silver tomentose to pale beige/gold tomentose throughout	30
30	Posterodorsal surface of anterior tibia bearing two strong setae (Fig. 49a)	31
–	Posterodorsal surface of anterior tibia bearing one strong seta (Fig. 49b)	32
31	Scutum with 2 pairs of sharply delineated narrow dorsal stripes; two postsutural intra-alars	* osvaldoespinozai * **sp. n.**
–	Scutum with two wide and diffuse dorsal stripes; three postsutural intra-alars	*placida* van der Wulp
32	Ocellar setae hair-like but present	33
–	Ocellar setae absent	35
33	Gena entirely silver tomentose	* duvalierbricenoi * **sp. n.**
–	Posterior half of gena gold tomentose	34
34	Coxae on all legs bright orange contrasting with femora	* diniamartinezae * **sp. n.**
–	Coxae on all legs dark reddish-brown concolorous with remainder of leg	*insularis* Curran
35	Black setulae present on underside of scutellum	* ricardocaleroi * **sp. n.**
–	Only plumose yellow setulae present on underside of scutellum	36
36	Abdominal ST1+2 predominantly brown throughout (dorsally and ventrally)	* carolinacanoae * **sp. n.**
–	Abdominal ST1+2 at least with orange ground color ventrally	37
37	Gena gold tomentose	* gloriasihezarae * **sp. n.**
–	Gena entirely silver tomentose	38
38	Midleg with yellow coxa, femur, tibia and tarsal segments dark brown with silver tomentum; T4 dark brown dorsally, with yellow ventrolaterally	* eldaarayae * **sp. n.**
–	Midleg with yellow coxa, femur, tibia and tarsal segments dark orange with silver tomentum only on dorsal surface; T4 dark maroon dorsally, orange ventrolaterally	*minor* (Thompson)

The area covered by this key is confined to the Mesoamerican biogeographical region between the Isthmus of Tehuantepec in Southern Mexico (northern limit), south to the Colombian border with Panama, and inclusive of the Antilles. Our key is based primarily on male specimens but can be applied to females as well.

## Discussion

**Figure 50.**
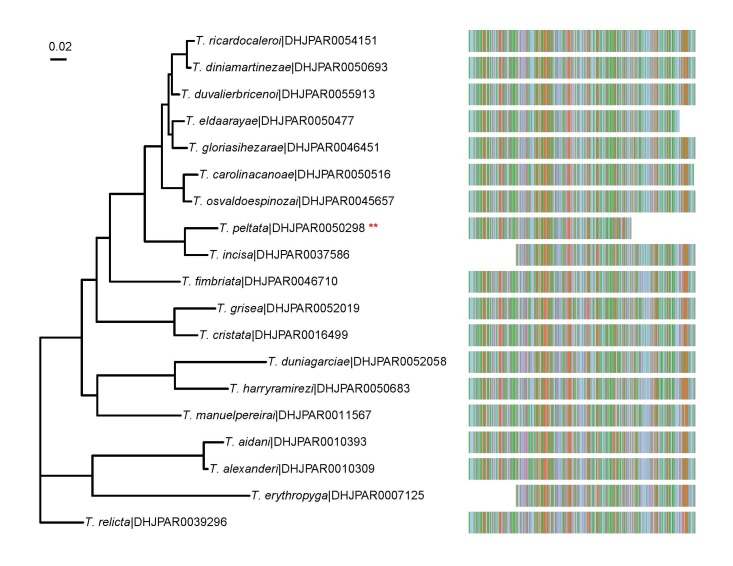
An unrooted phylogenetic tree for nineteen species of ACG *Telothyria* inferred by using the Maximum Likelihood (ML) method based on the General Time Reversible model (Nei and Kumar 2000) conducted in MEGA X (Kumar et al. 2018) and presented using the ggtree package (Yu et al. 2016) in R (Team 2019). Tip labels are species names and the DHJPAR accession for the holotype and are associated with an image of the DNA barcode (with coloured bars representing the A (green), T (blue), C (purple) or G (red). Exceptions to this pattern are first for *T. relicta* (where the holotype has not been sequenced) and for *T. peltata* (marked with two red asterisks) where the DNA barcode is not known and what has been included in this ML tree is pseudogene amplicon that has been amplified for all three known samples of this species.

In the process of species determination, sequences derived from ACG specimens provided one example (*T. peltata* - marked with two red asterisks in Fig. 50) where the DNA barcode remains unknown. From each of the three currently known specimens the PCR amplification has produced a sequence with a single base pair deletion midway through the barcode region – a signature of a pseudogene since all bases after that position would be out of frame. Although *T. peltata* sequences are unlikely to be coding DNA, we included this pseudogene amplicon in our ML tree as that has been amplified for all three known samples of this species, but it is important to note that the species description for *T. peltata* is based only on the morphological differences.

## Supplementary Material

XML Treatment for Telothyria

XML Treatment for Telothyria aidani

XML Treatment for Telothyria alexanderi

XML Treatment for Telothyria auranticrus

XML Treatment for Telothyria auriolus

XML Treatment for Telothyria bicuspidata

XML Treatment for Telothyria carolinacanoae

XML Treatment for Telothyria clavata

XML Treatment for Telothyria cristata

XML Treatment for Telothyria cupreiventris

XML Treatment for Telothyria diniamartinezae

XML Treatment for Telothyria duniagarciae

XML Treatment for Telothyria duvalierbricenoi

XML Treatment for Telothyria eldaarayae

XML Treatment for Telothyria erythropyga

XML Treatment for Telothyria fimbriata

XML Treatment for Telothyria fulgida

XML Treatment for Telothyria gloriasihezarae

XML Treatment for Telothyria grisea

XML Treatment for Telothyria harryramirezi

XML Treatment for Telothyria incisa

XML Treatment for Telothyria manuelpereirai

XML Treatment for Telothyria obscura

XML Treatment for Telothyria omissa

XML Treatment for Telothyria osvaldoespinozai

XML Treatment for Telothyria peltata

XML Treatment for Telothyria relicta

XML Treatment for Telothyria ricardocaleroi

## Figures and Tables

**Figure 2a.**
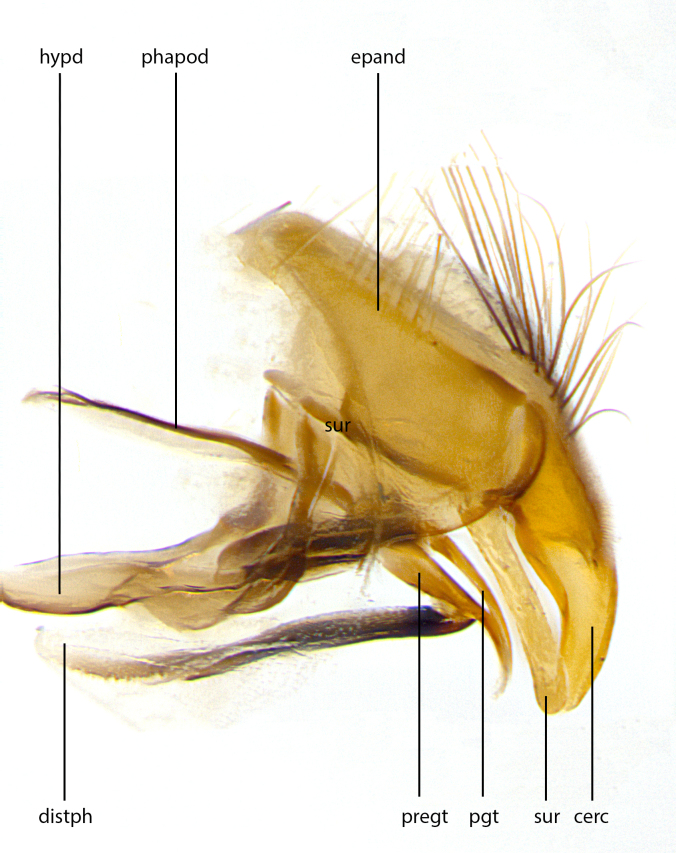
abbreviations: cerc = cercus; distph = distiphallus; epand = epandrium; epiph = epiphallus; hypd = hypandrium; phapod = phalloapodeme; pgt = postgonite; pregt = pregonite; sur = surstylus.

**Figure 2b.**
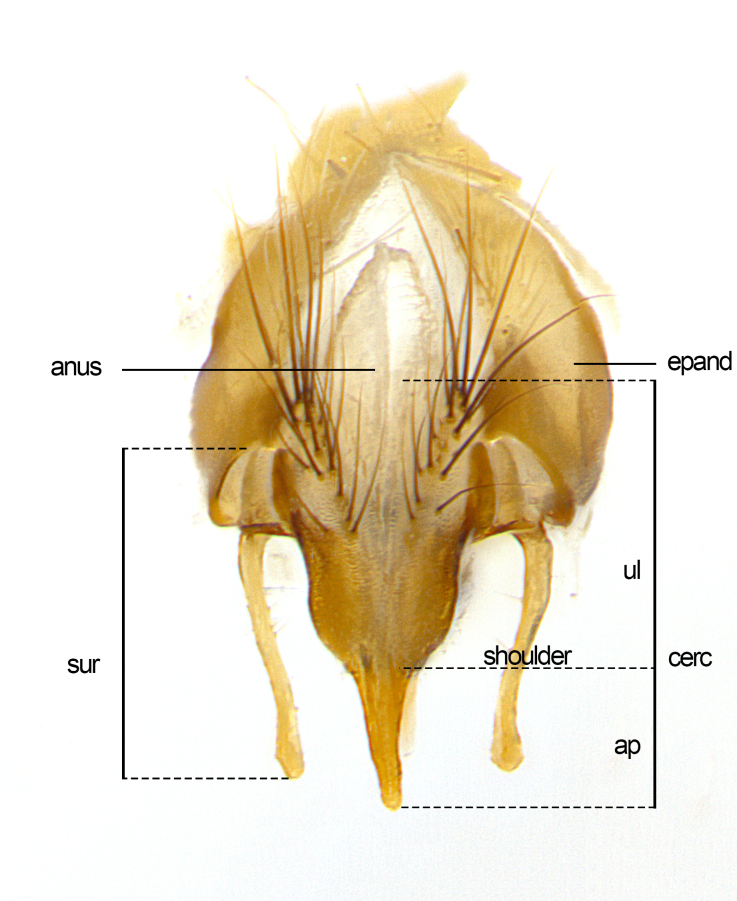
abbreviations for sections measured: anus = anal operculum; ap = apical section of cercus; epand = epandrium; ul = upper lobe of cercus; shoulder = shoulder point between apical section and upper lobe of cercus; sur = surstylus.

**Figure 2c.**
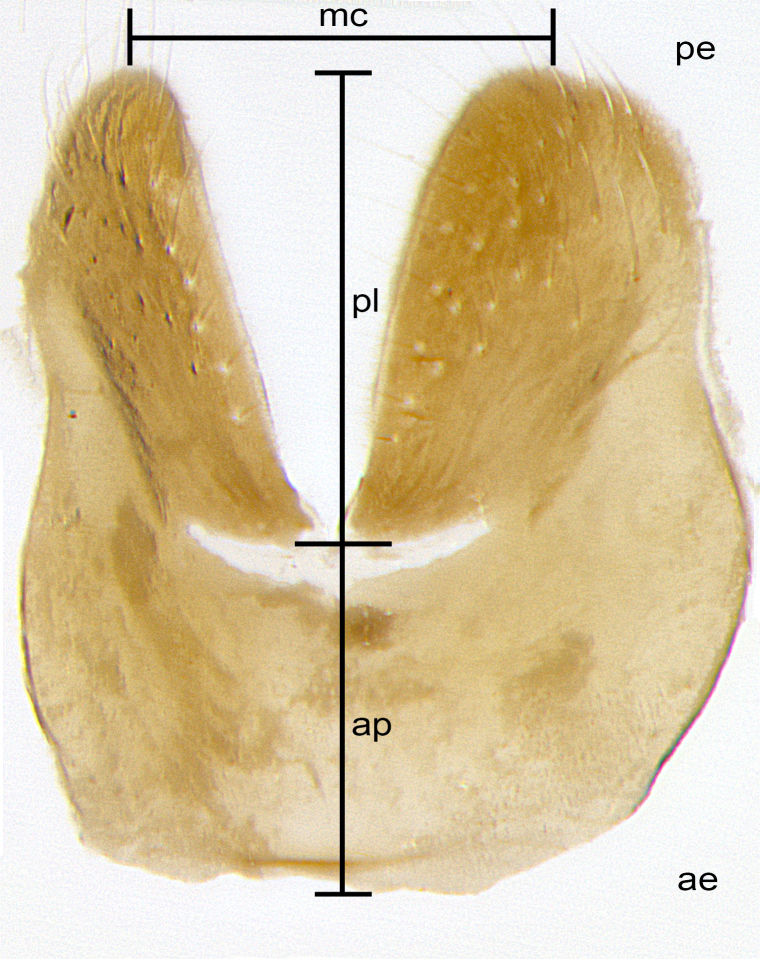
abbreviations: ae = anterior edge; ap = anterior plate; mc = median cleft; pe = posterior edge; pl = posterior lobes.

**Figure 2d.**
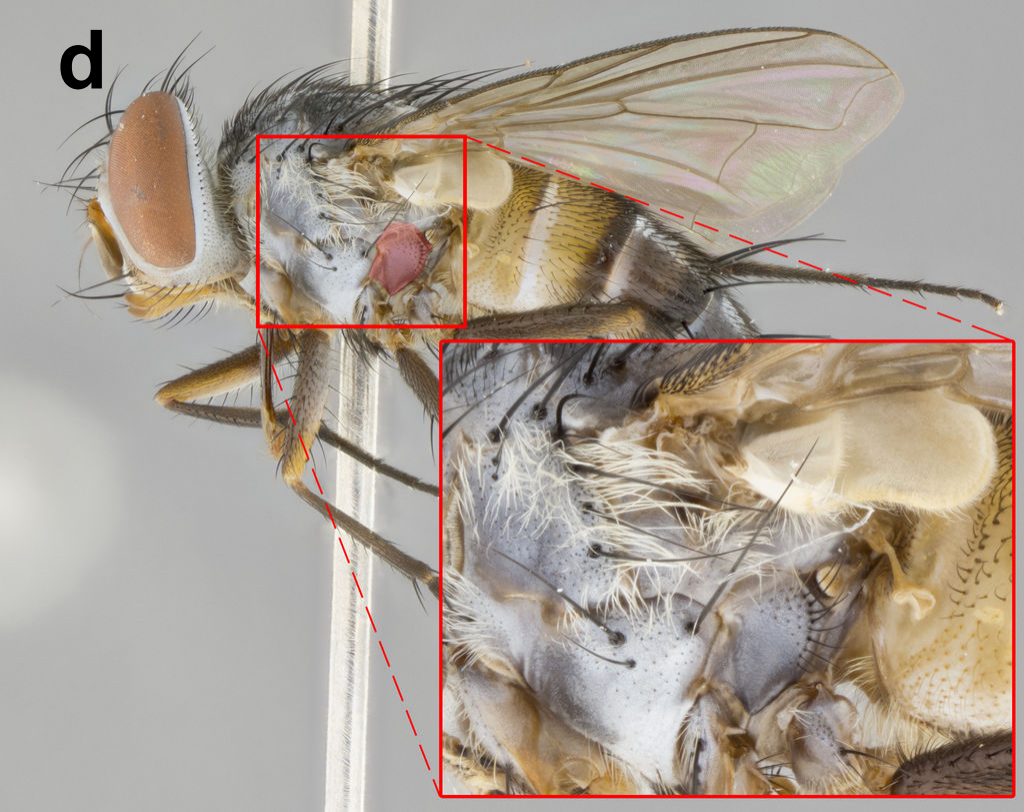
lateral habitus of *Telothyria grisea* **sp. n.** female (voucher n. DHJPAR0038741) inset detailing the presence of "normal" meral setae.

**Figure 2e.**
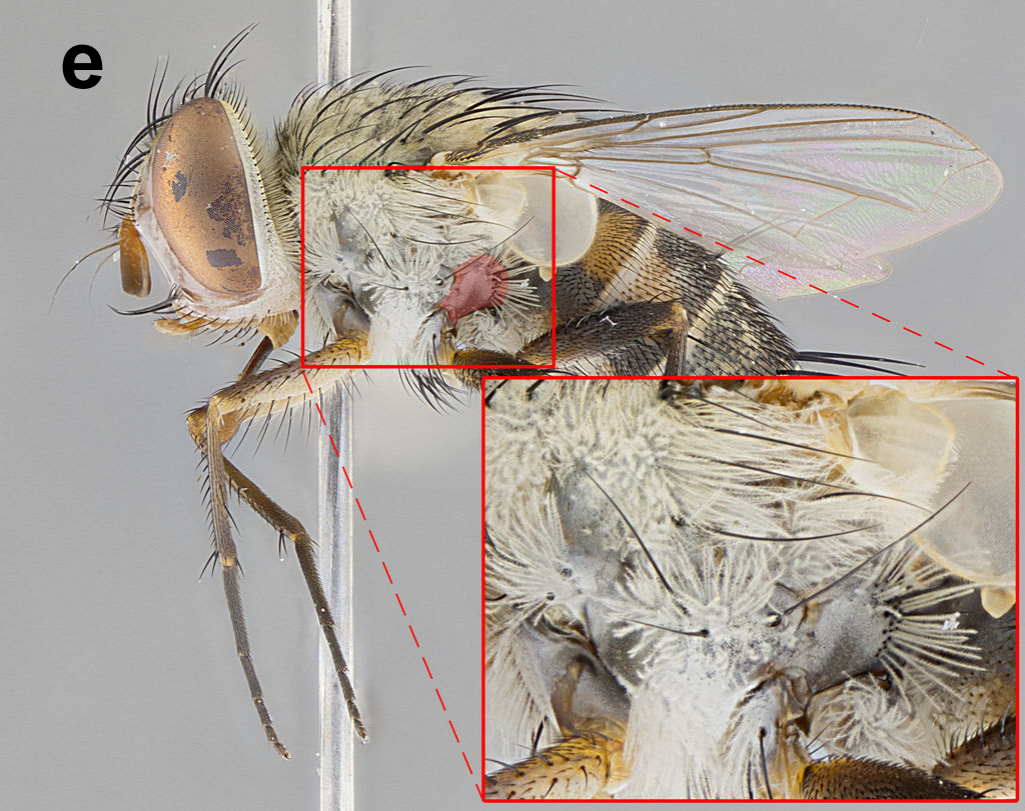
lateral habitus of *Telothyria relicta* female (voucher n. DHJPAR0050630) inset detailing the presence of plumose meral setae.

**Figure 2f.**
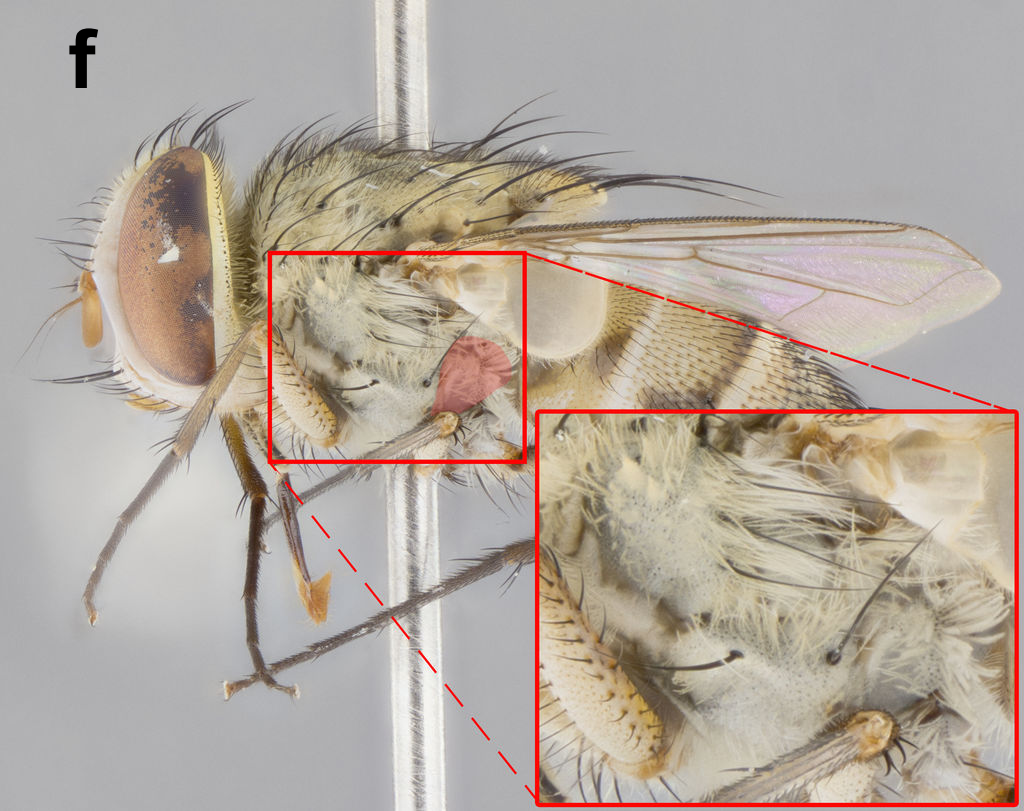
lateral habitus of *Telothyria peltata* **sp. n.** male (voucher n. DHJPAR0050298) inset detailing the presence of plumose meral setae typical of all the males of the genus.

**Figure 3a.**
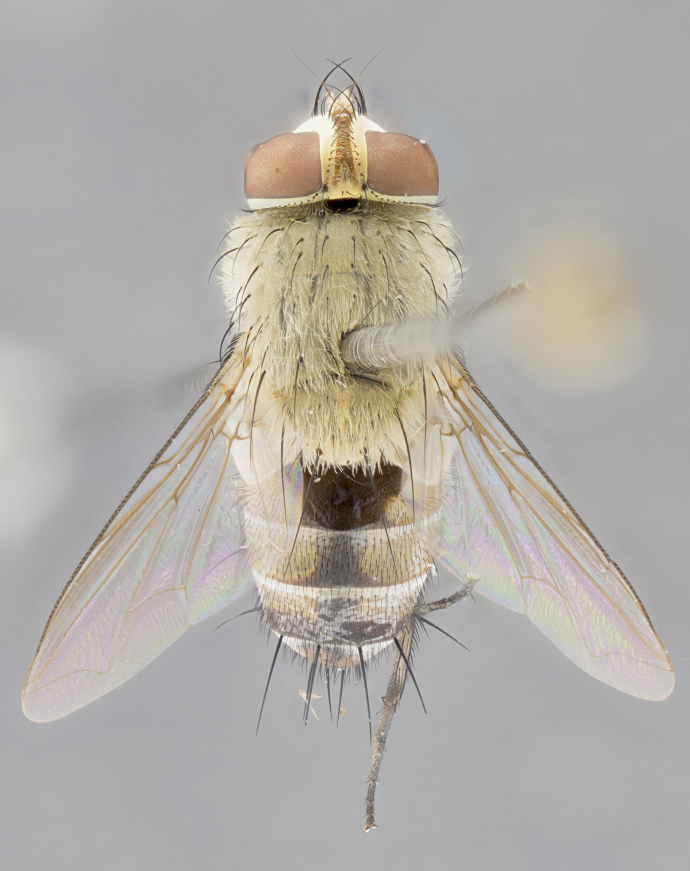
dorsal view.

**Figure 3b.**
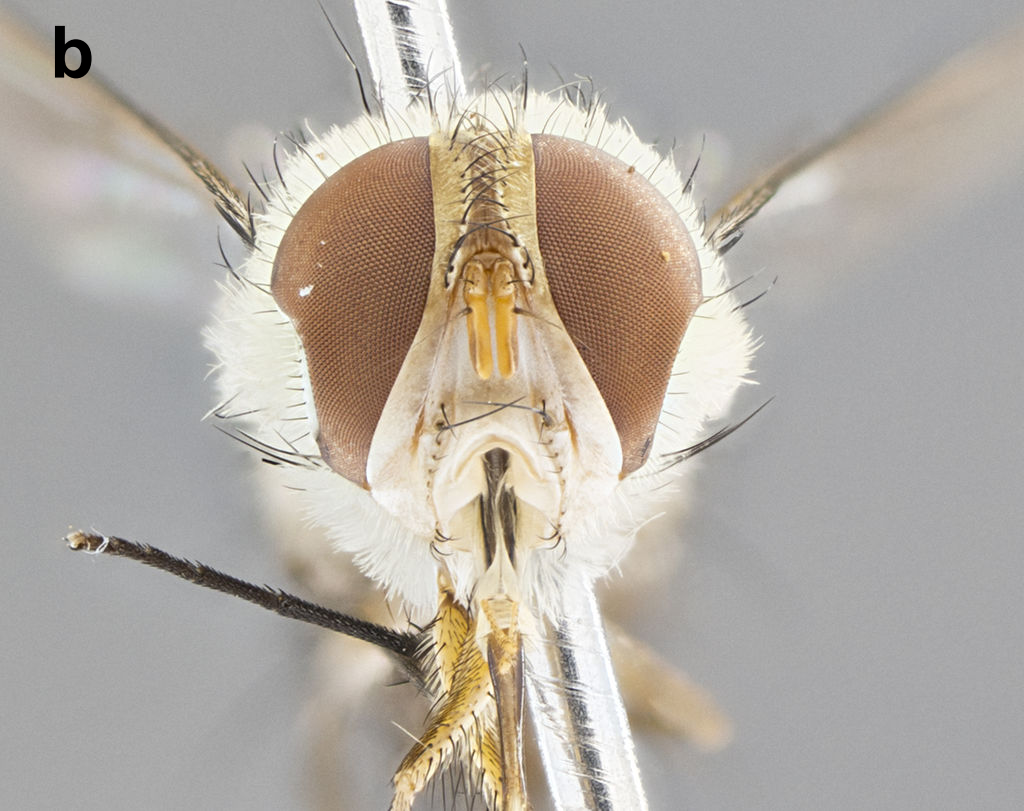
frontal view.

**Figure 3c.**
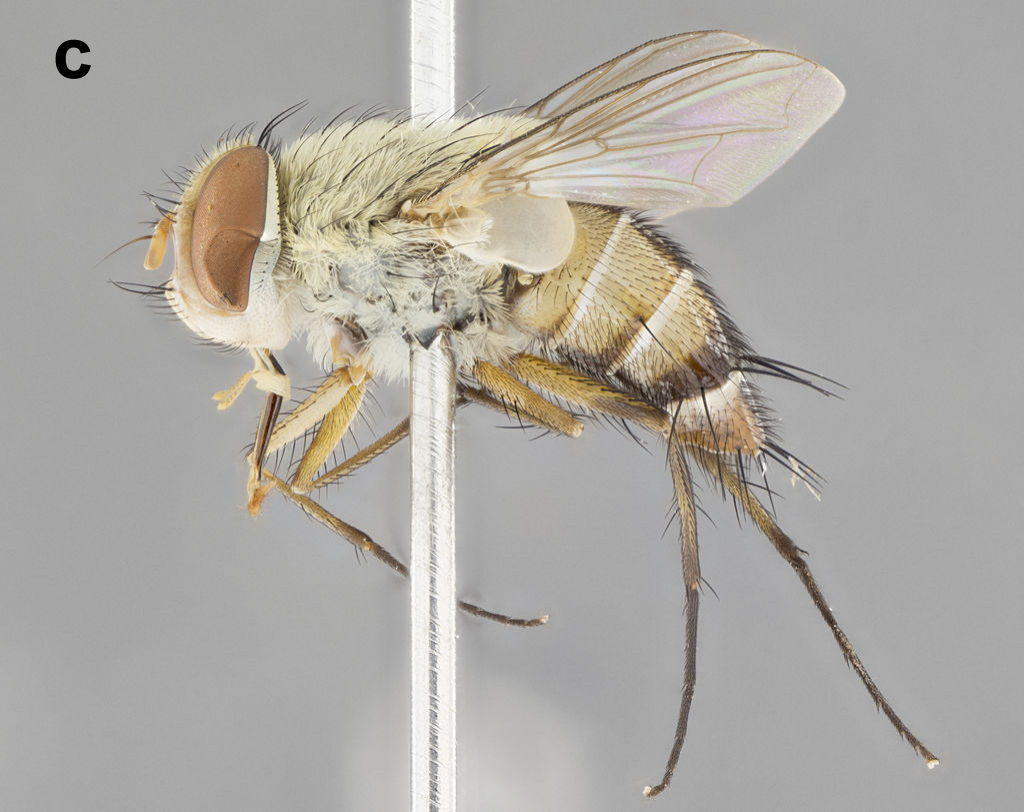
lateral view.

**Figure 3d.**
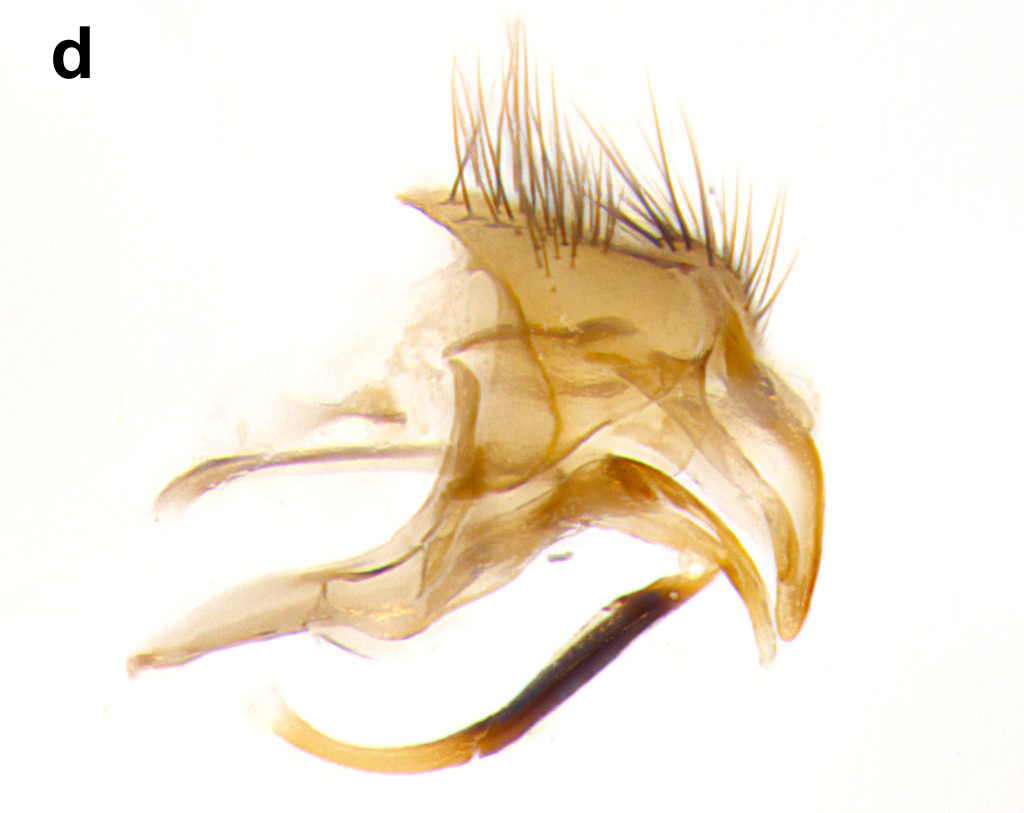
caudal view.

**Figure 3e.**
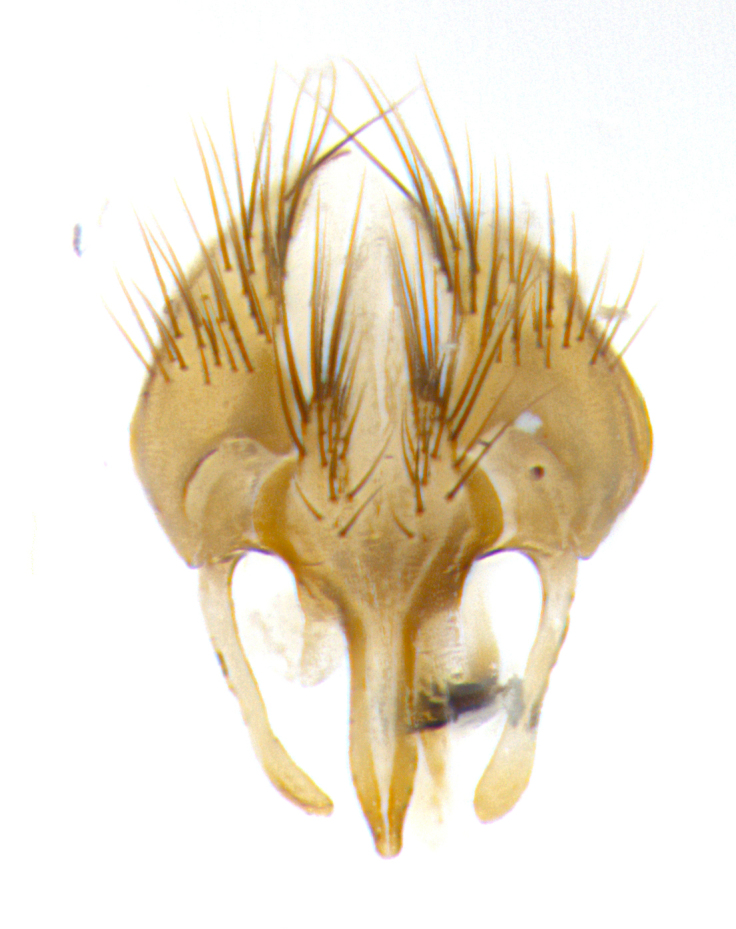
lateral view.

**Figure 3f.**
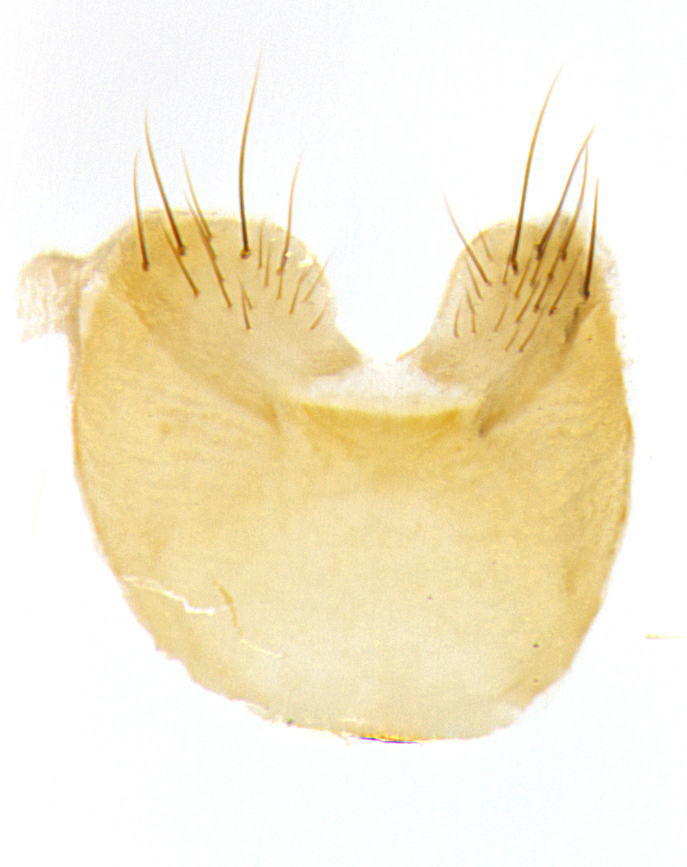
sternite 5, ventral view.

**Figure 4a.**
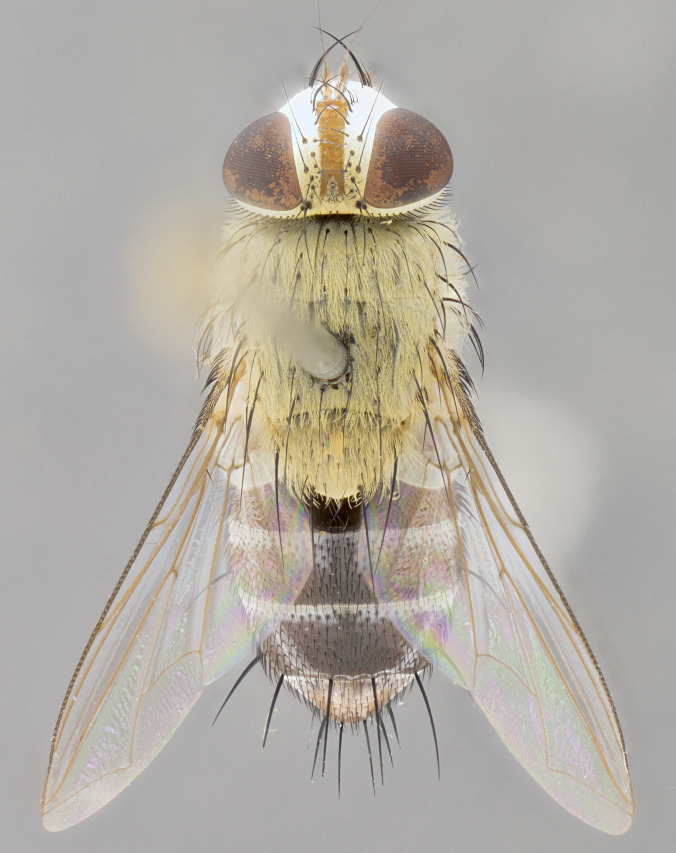
dorsal view.

**Figure 4b.**
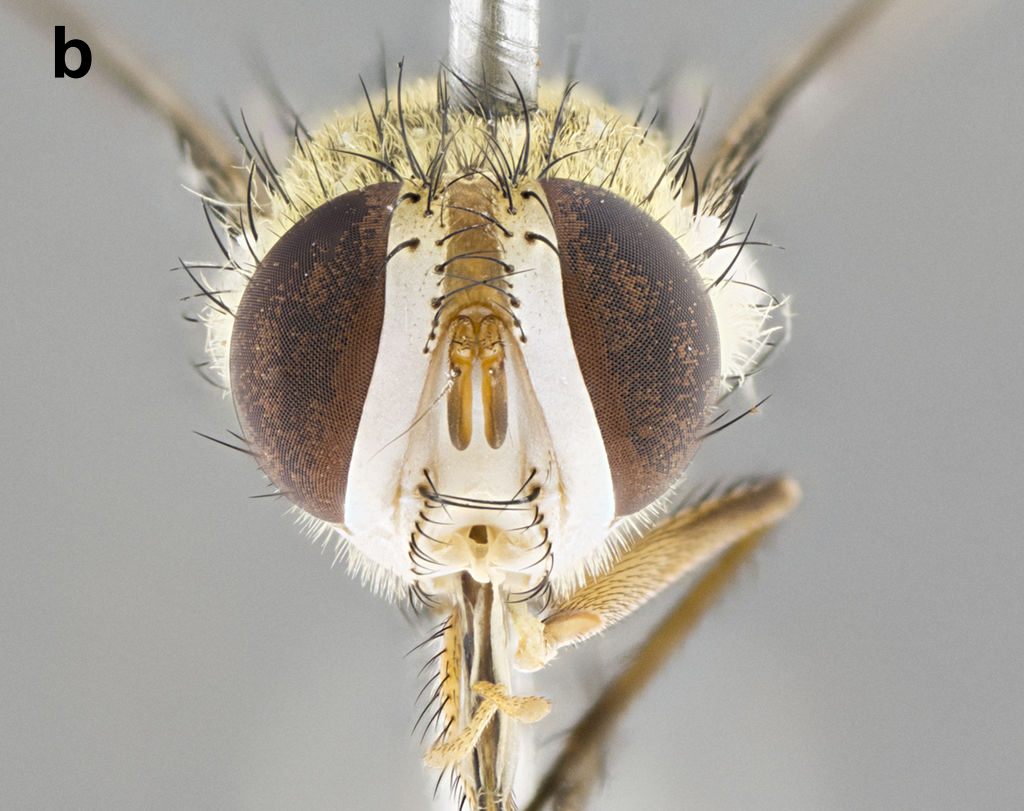
frontal view.

**Figure 4c.**
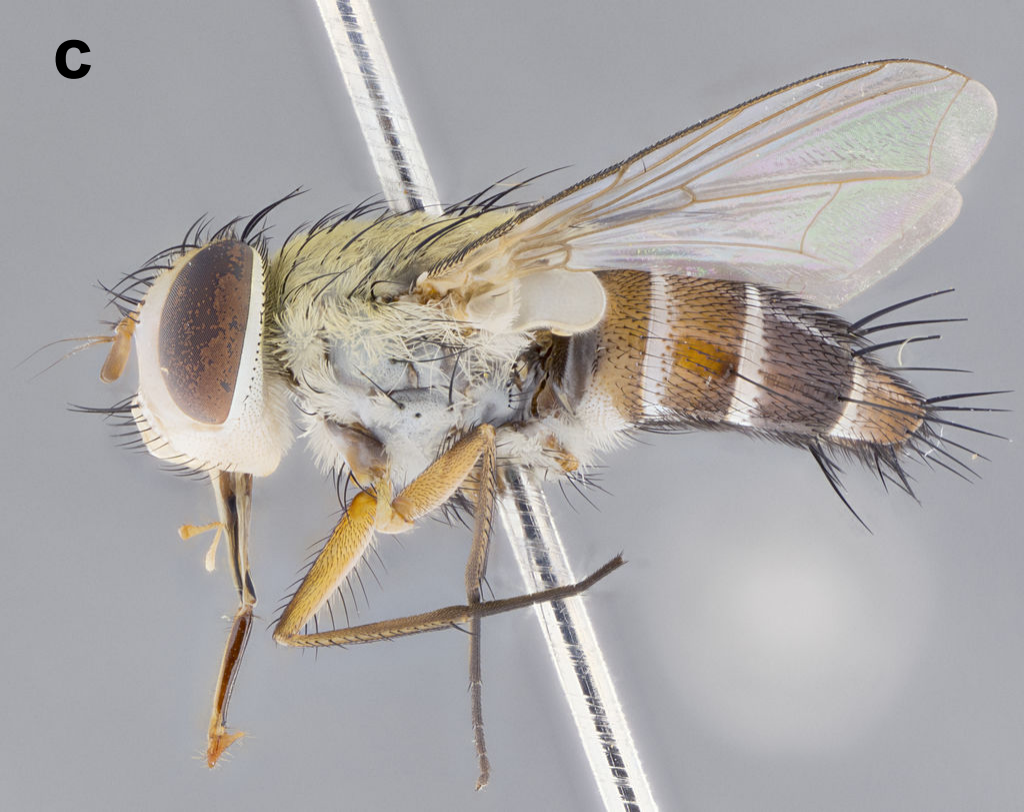
lateral view.

**Figure 5a.**
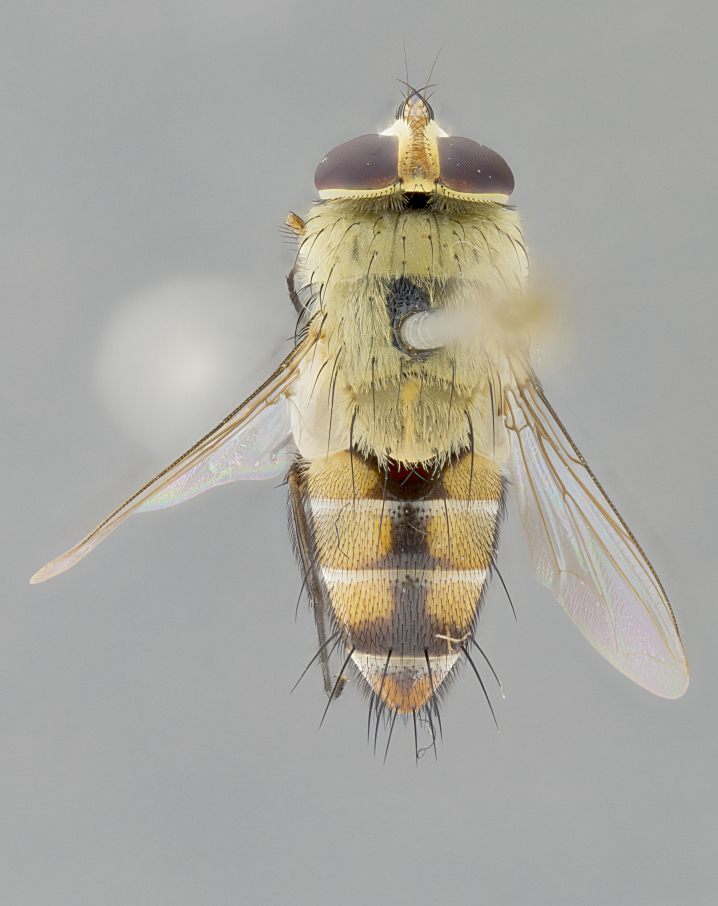
dorsal view.

**Figure 5b.**
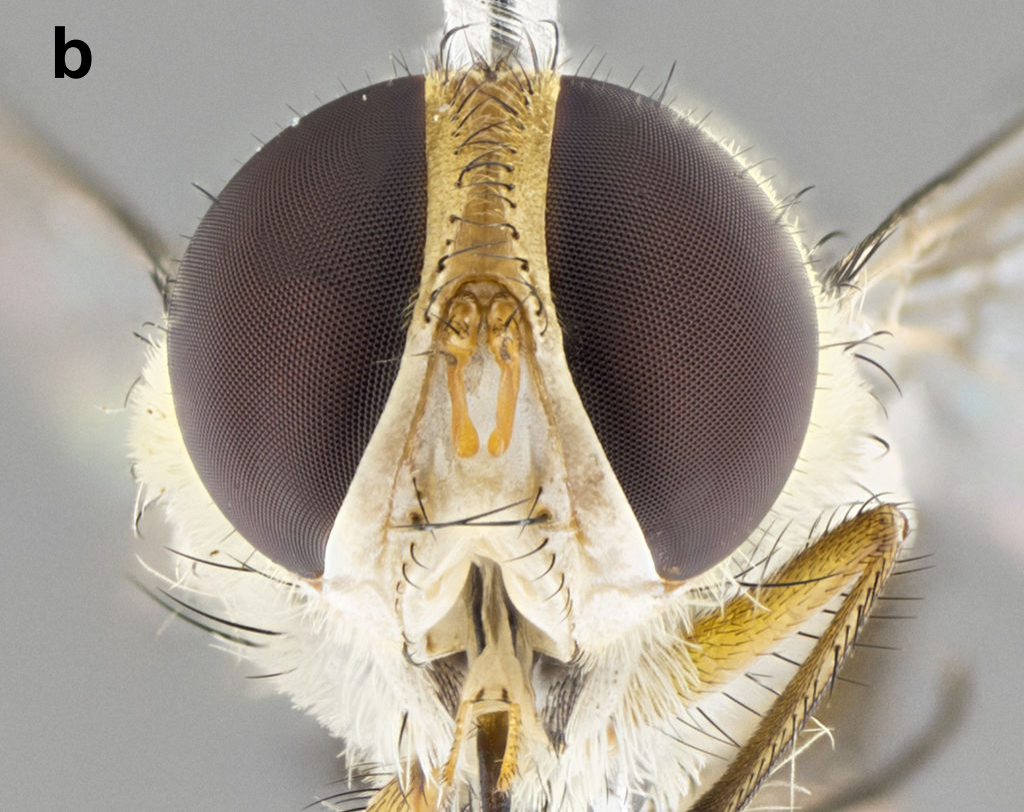
frontal view.

**Figure 5c.**
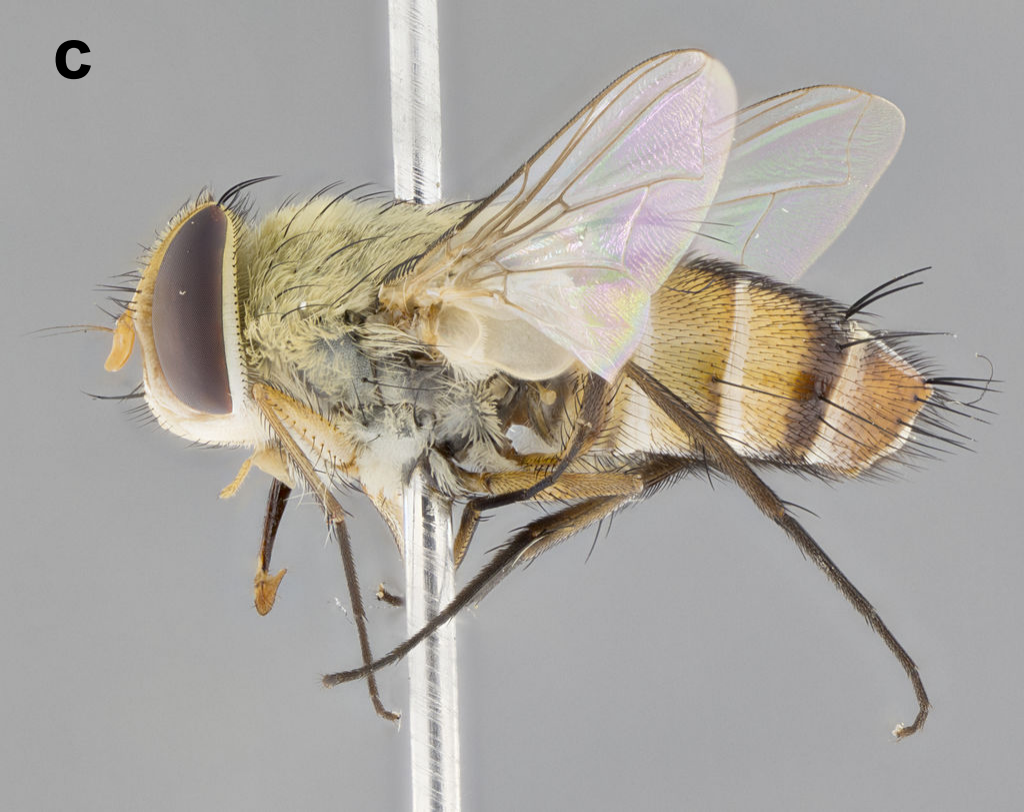
lateral view.

**Figure 6a.**
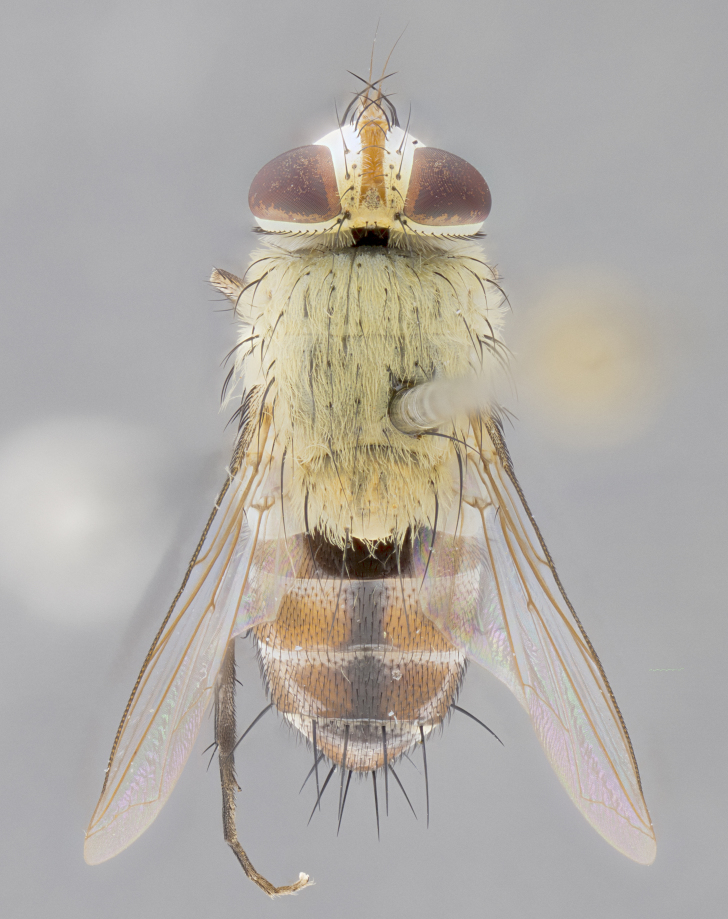
dorsal view.

**Figure 6b.**
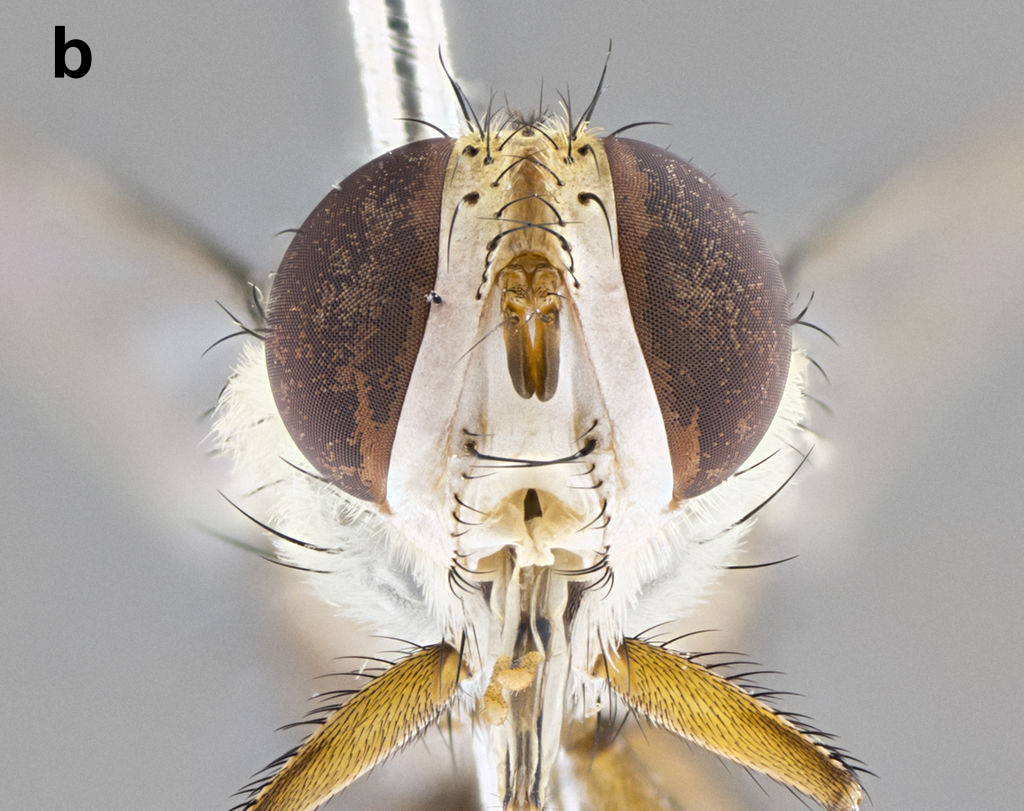
frontal view.

**Figure 6c.**
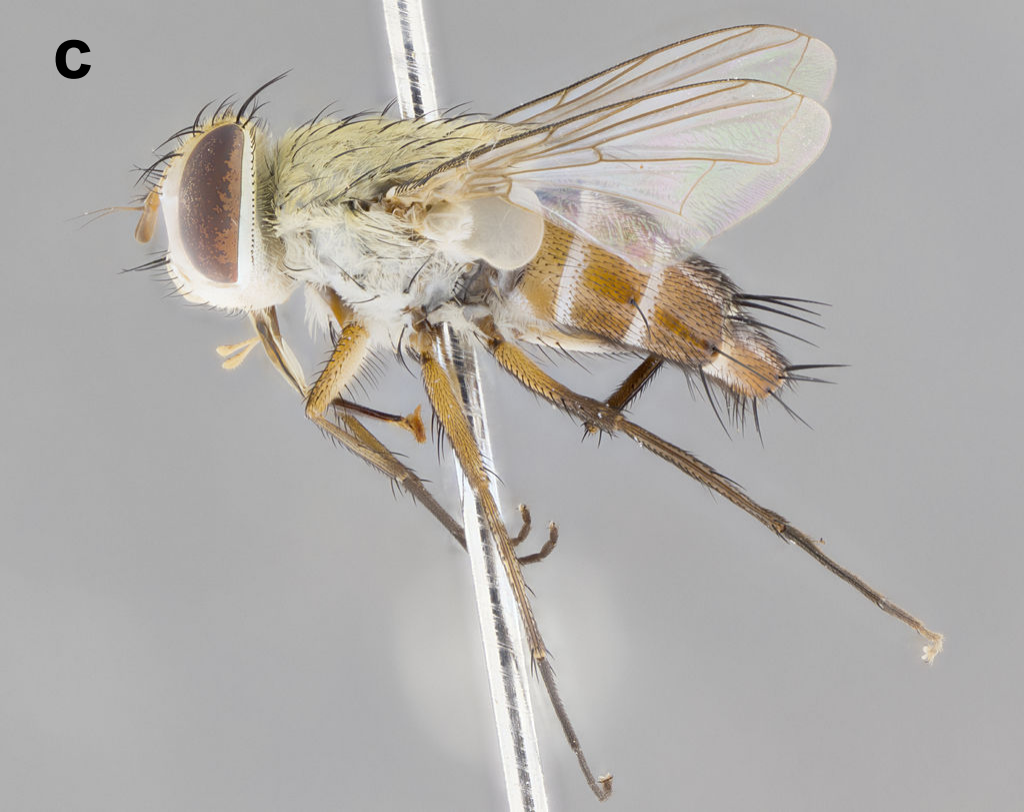
lateral view.

**Figure 7a.**
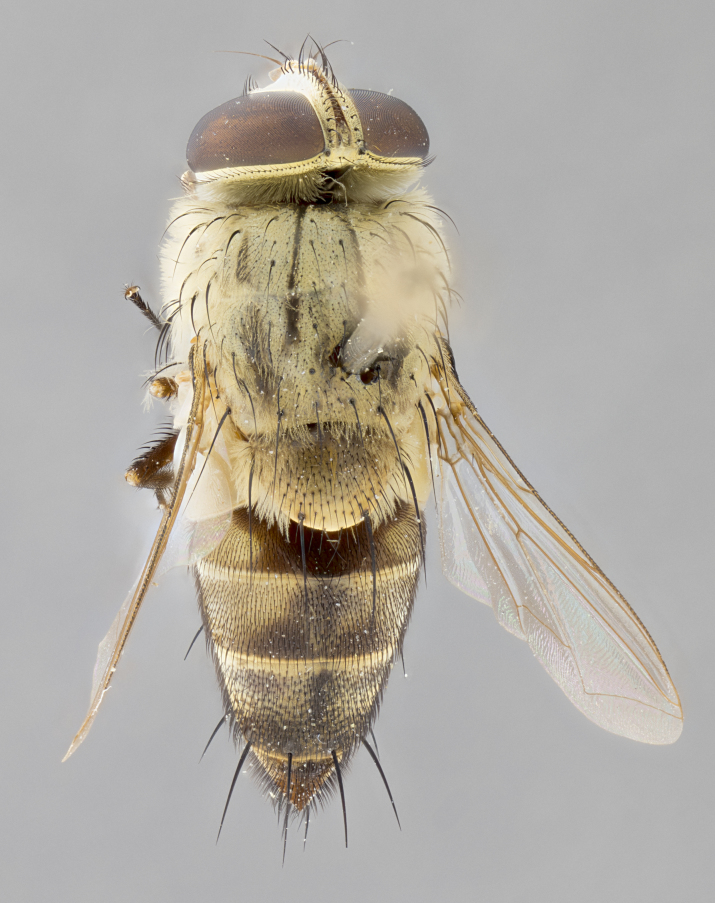
dorsal view.

**Figure 7b.**
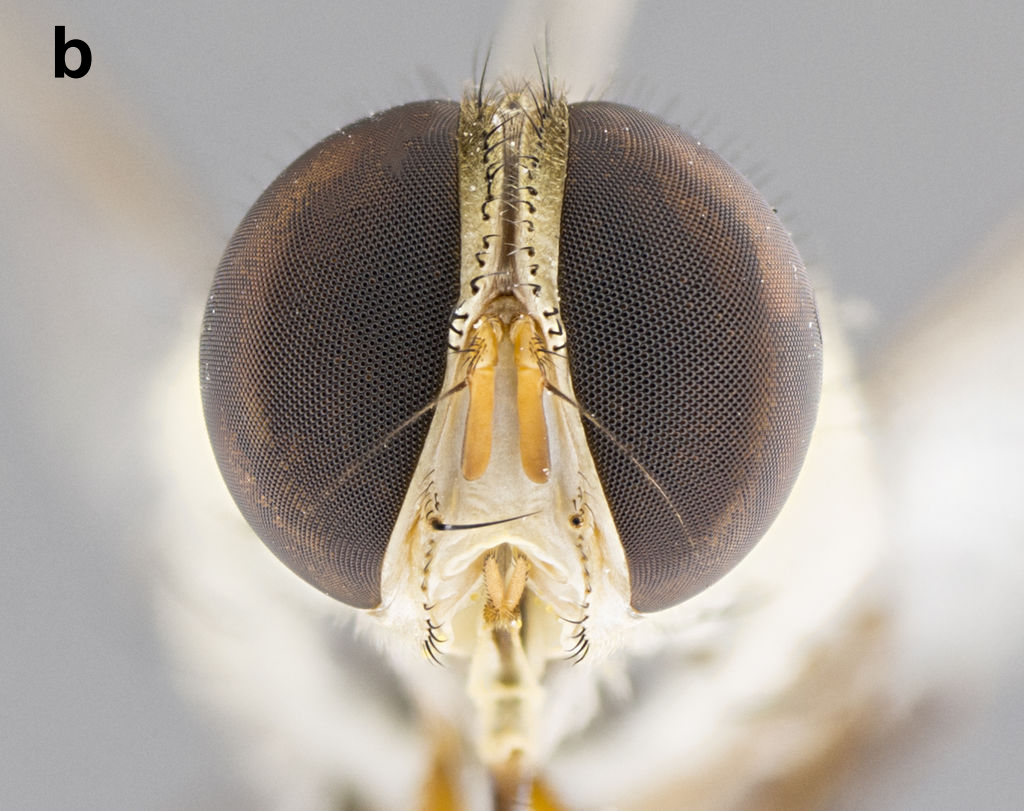
frontal view.

**Figure 7c.**
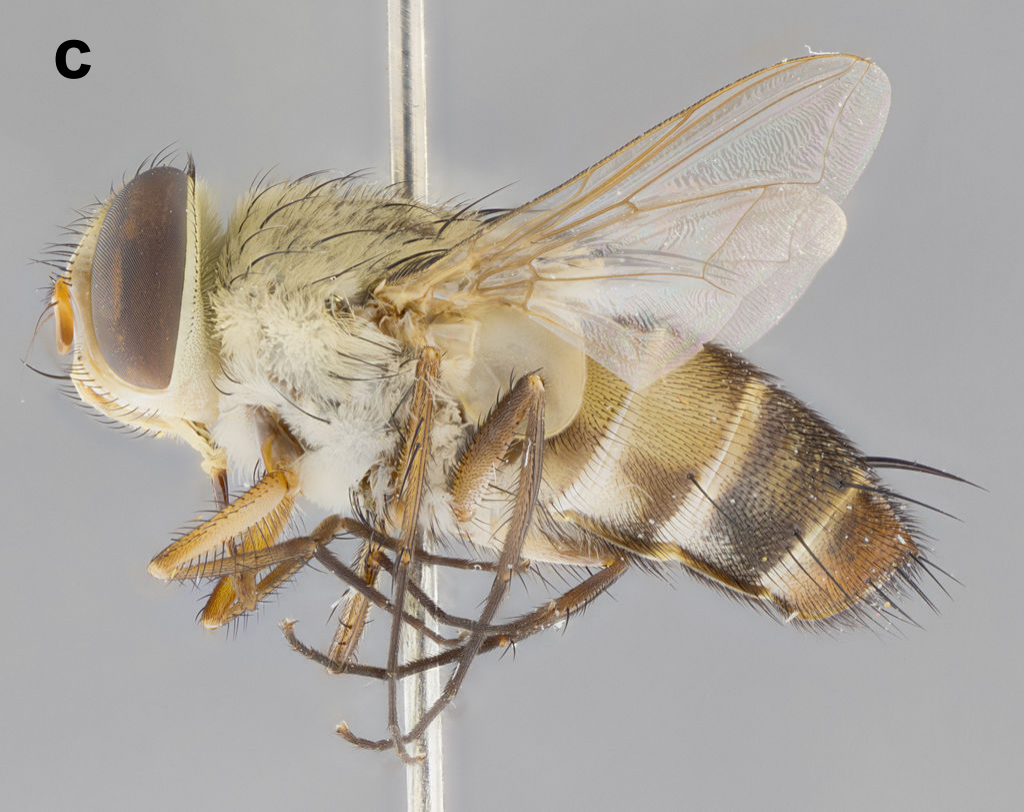
lateral view.

**Figure 7d.**
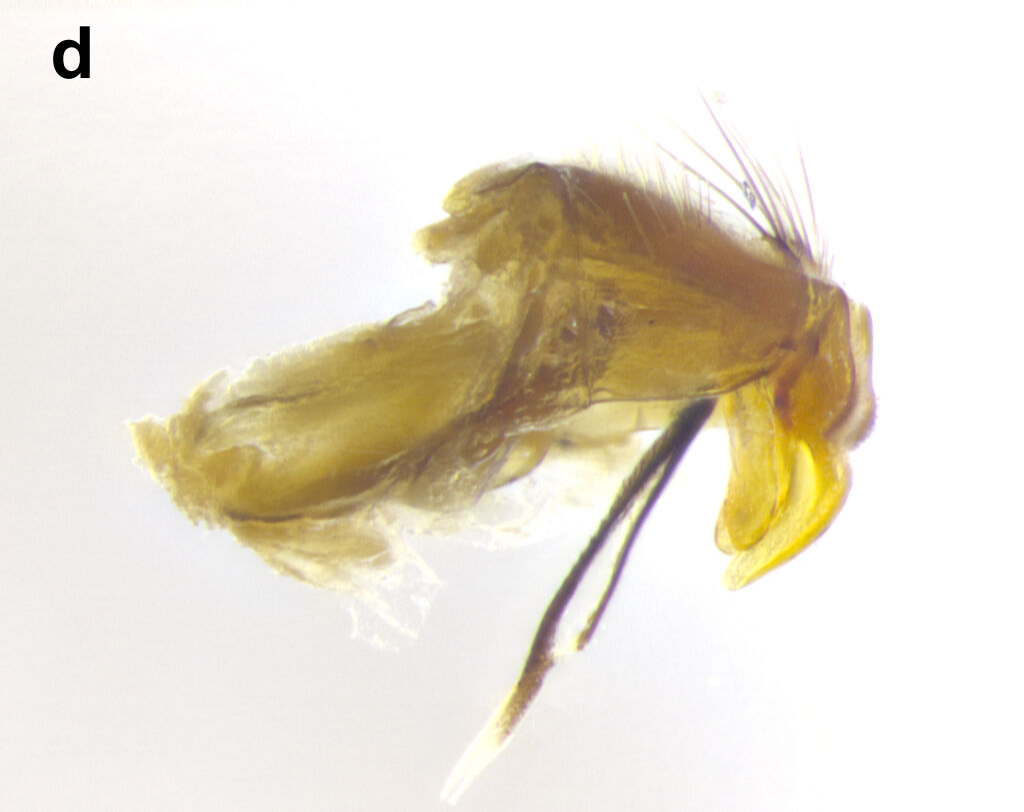
caudal view.

**Figure 7e.**
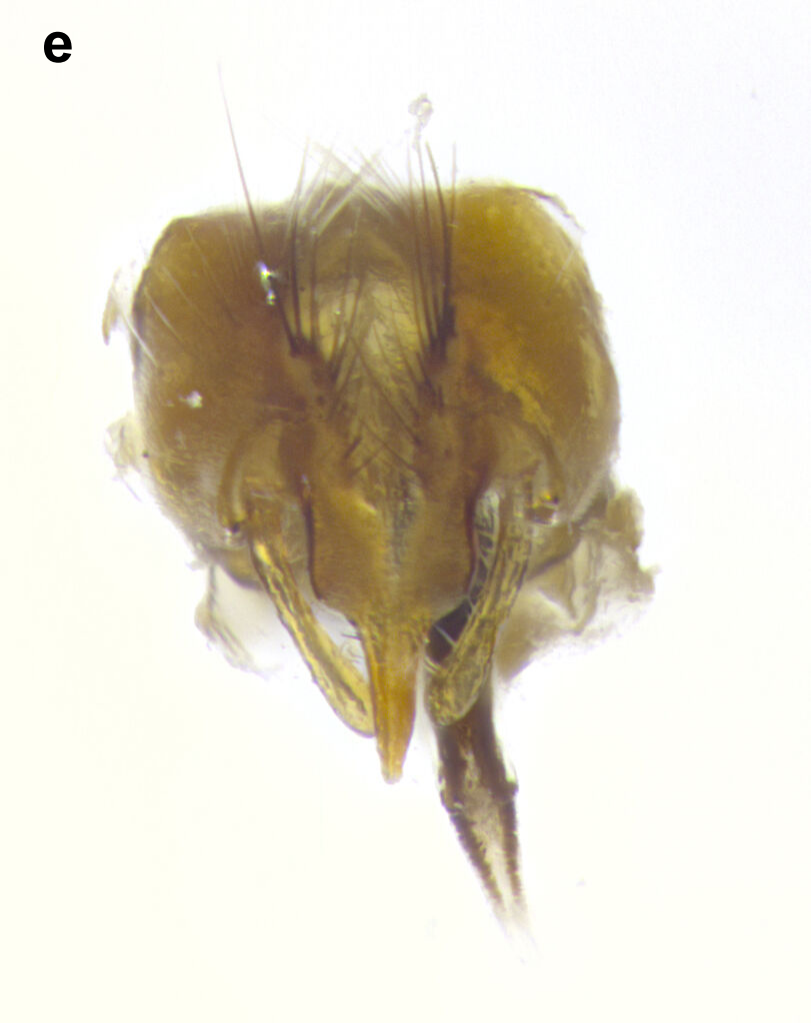
lateral view.

**Figure 7f.**
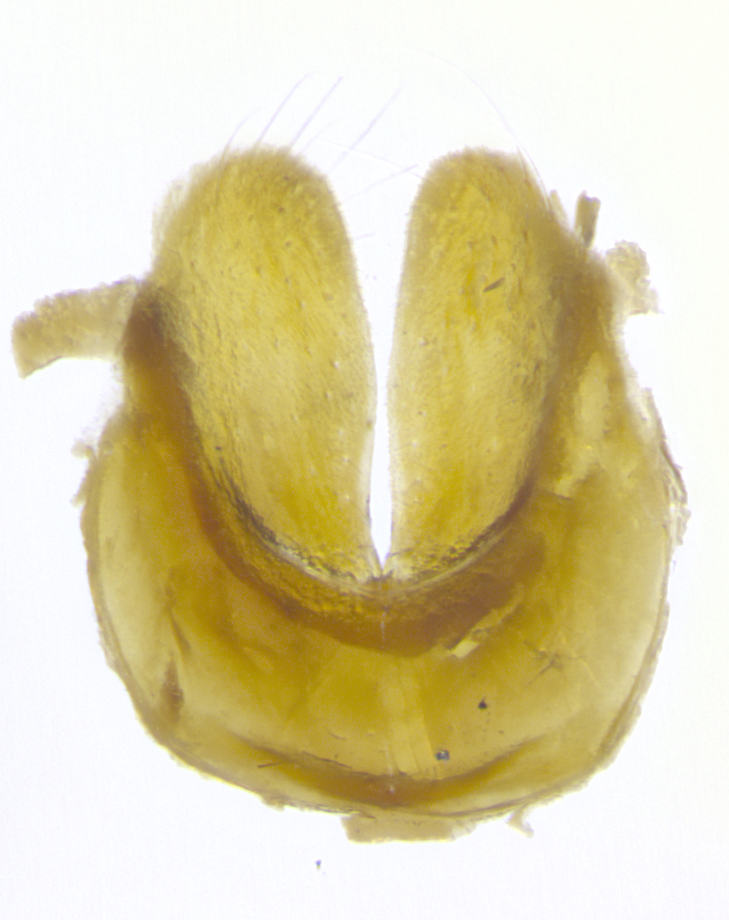
Sternite 5, ventral view.

**Figure 8a.**
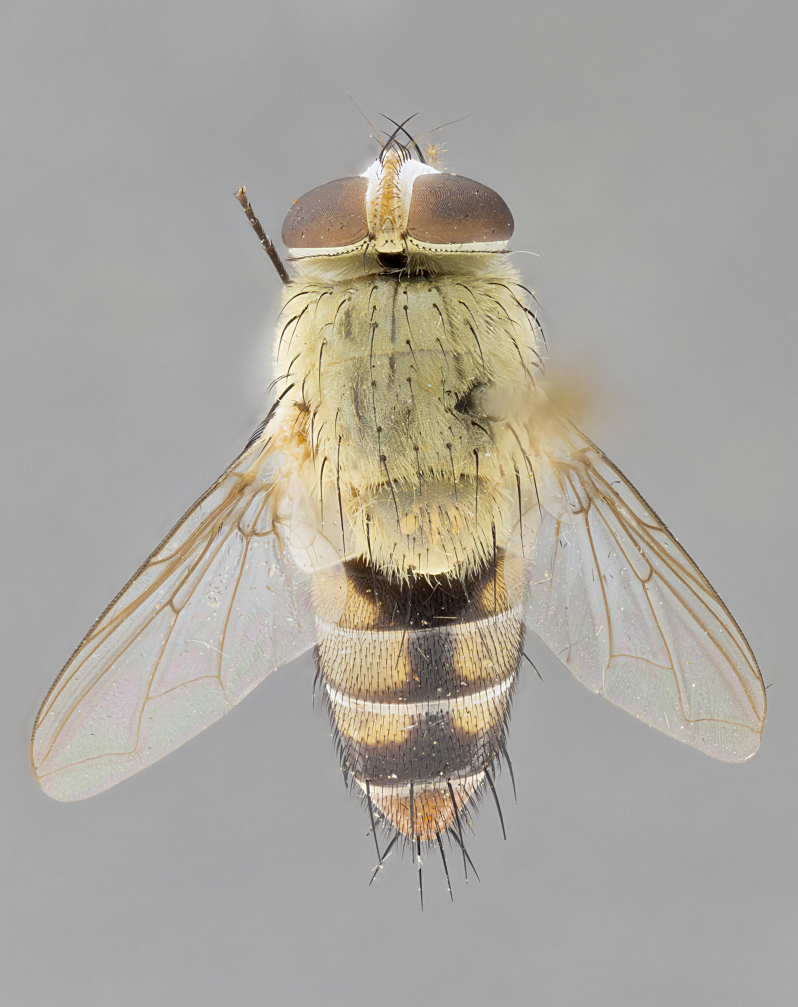
dorsal view.

**Figure 8b.**
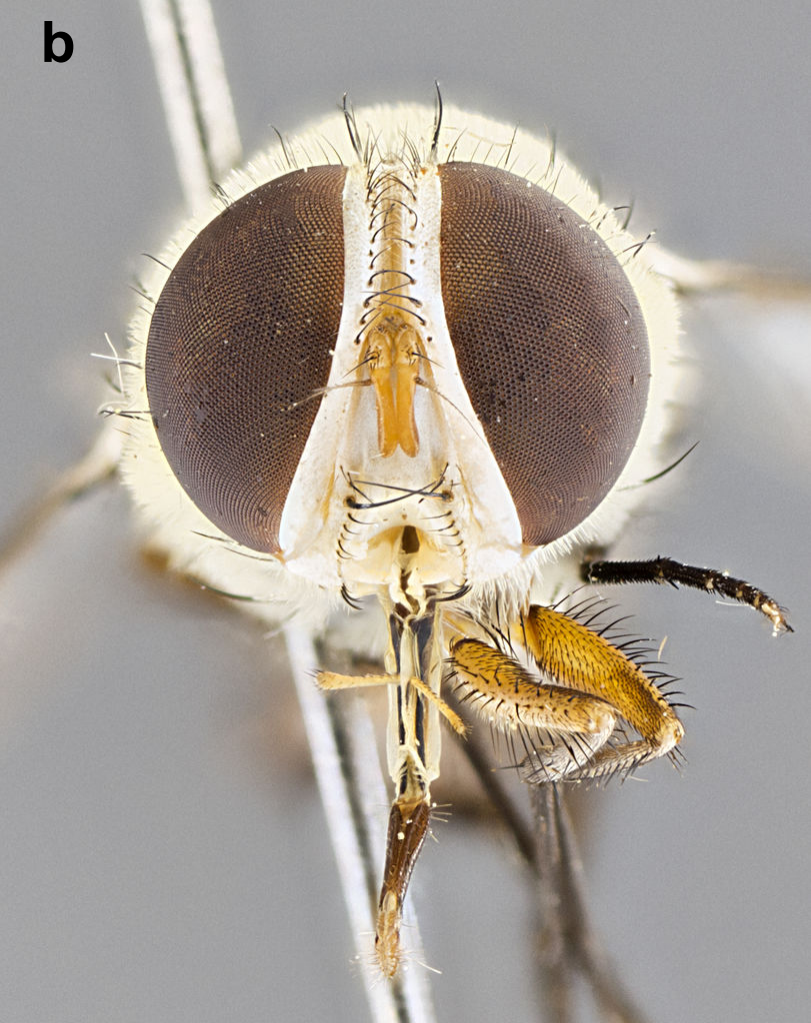
frontal view.

**Figure 8c.**
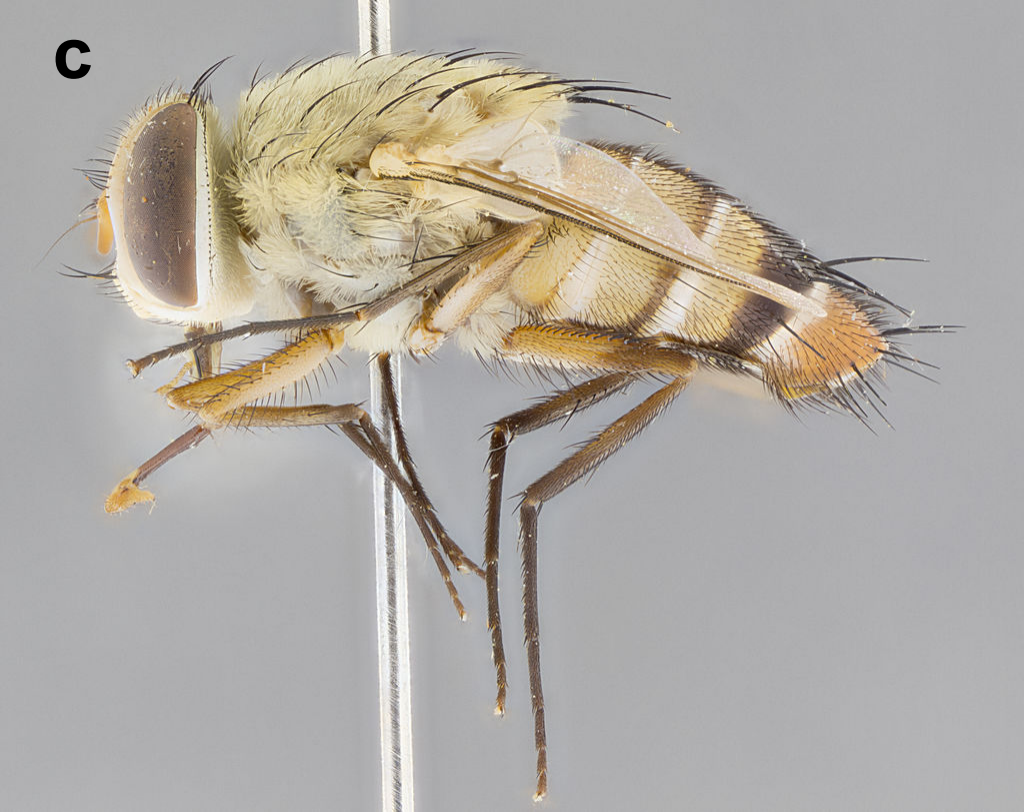
lateral view.

**Figure 9a.**
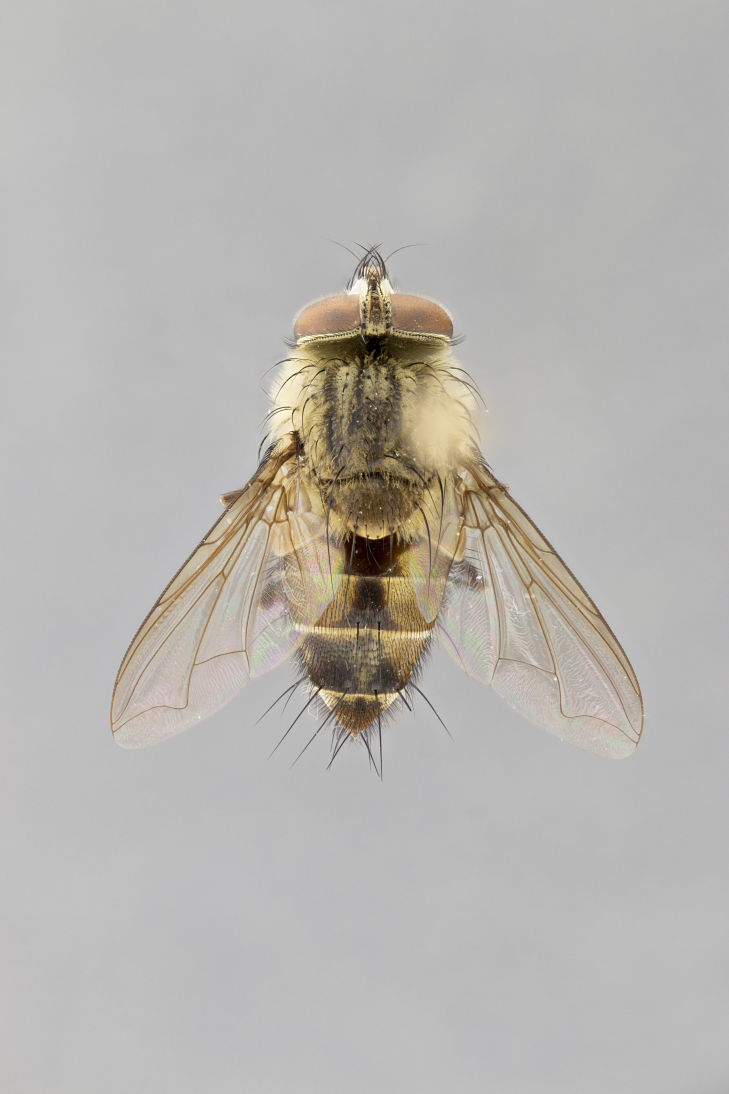
dorsal view.

**Figure 9b.**
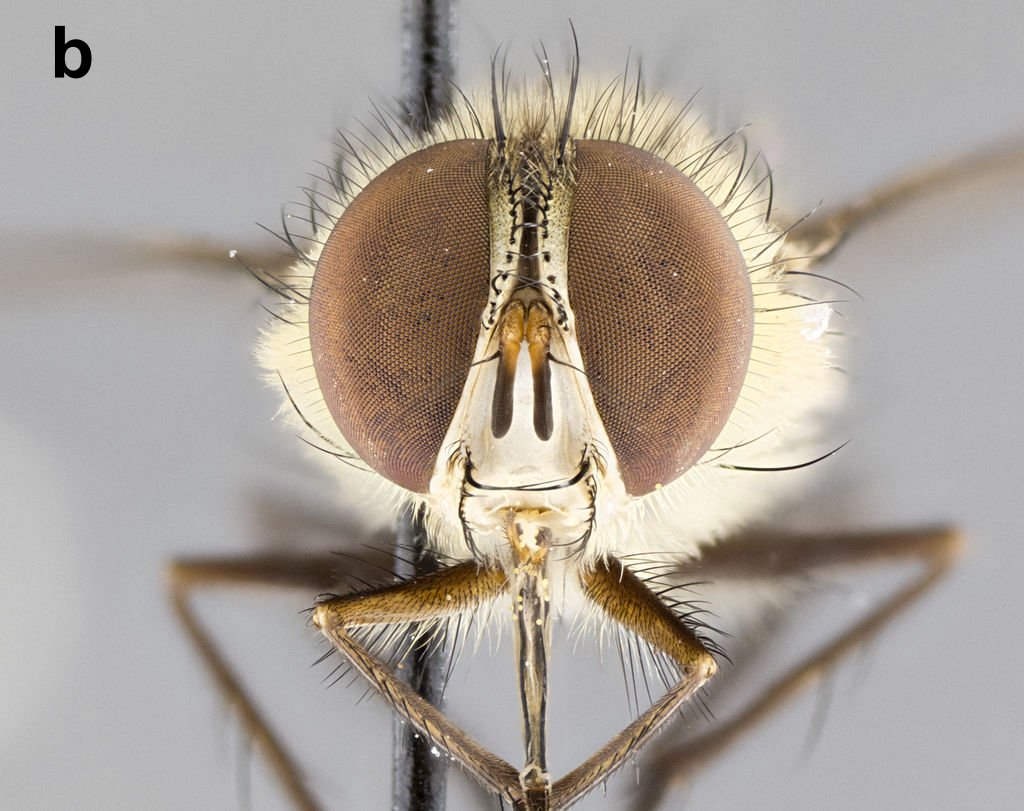
frontal view.

**Figure 9c.**
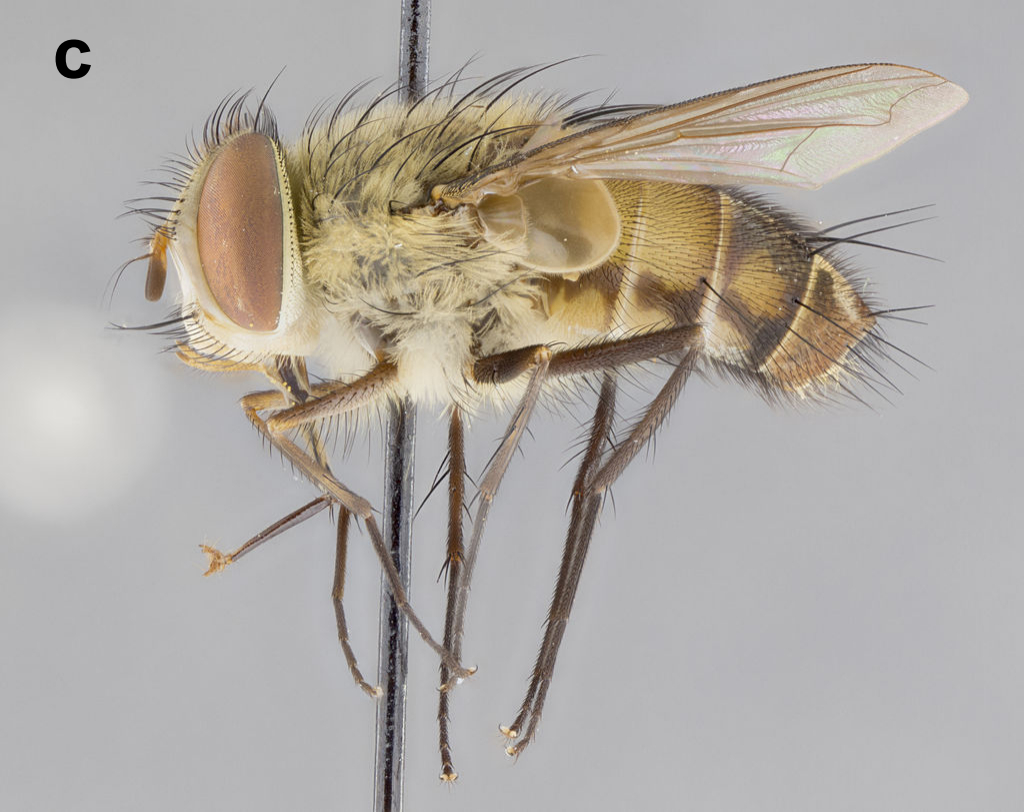
lateral view.

**Figure 9d.**
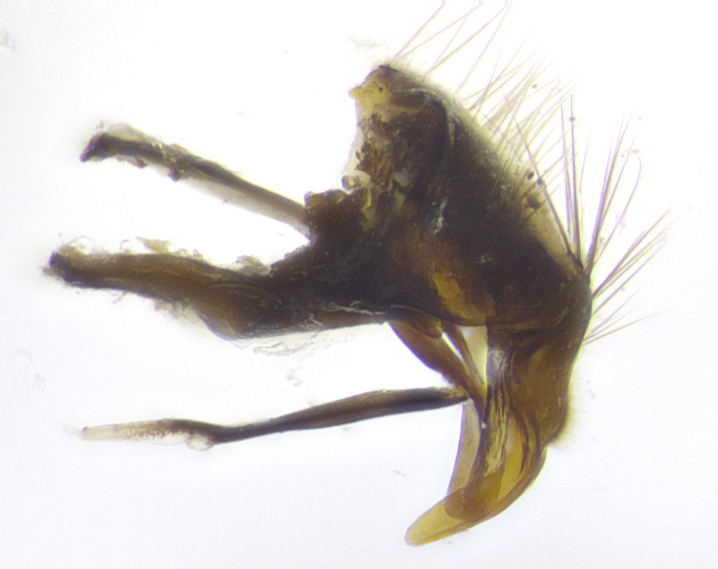
caudal view.

**Figure 9e.**
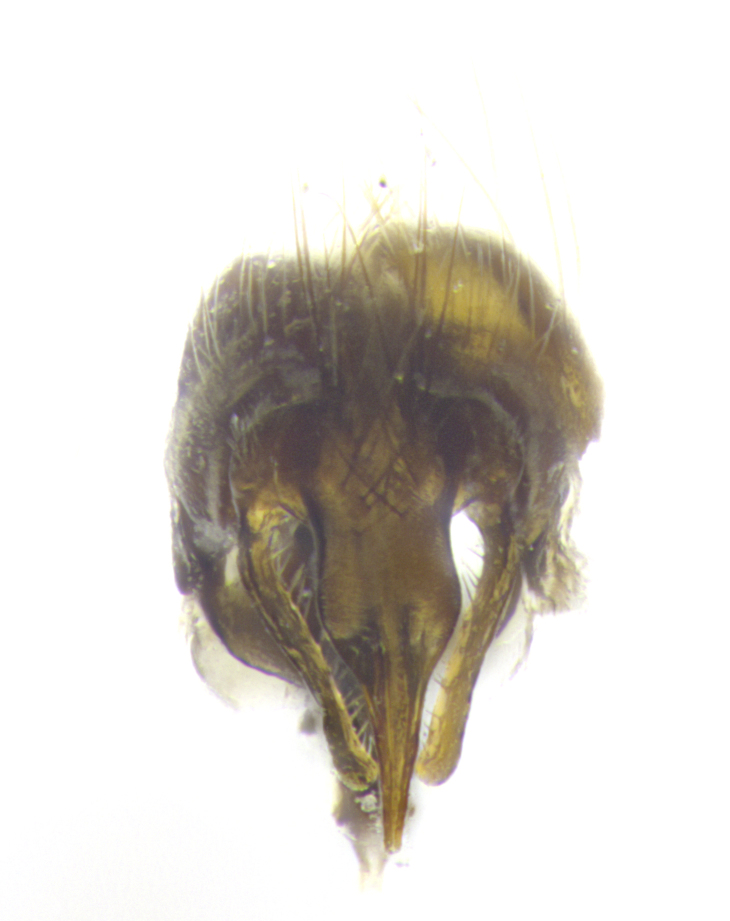
lateral view.

**Figure 9f.**
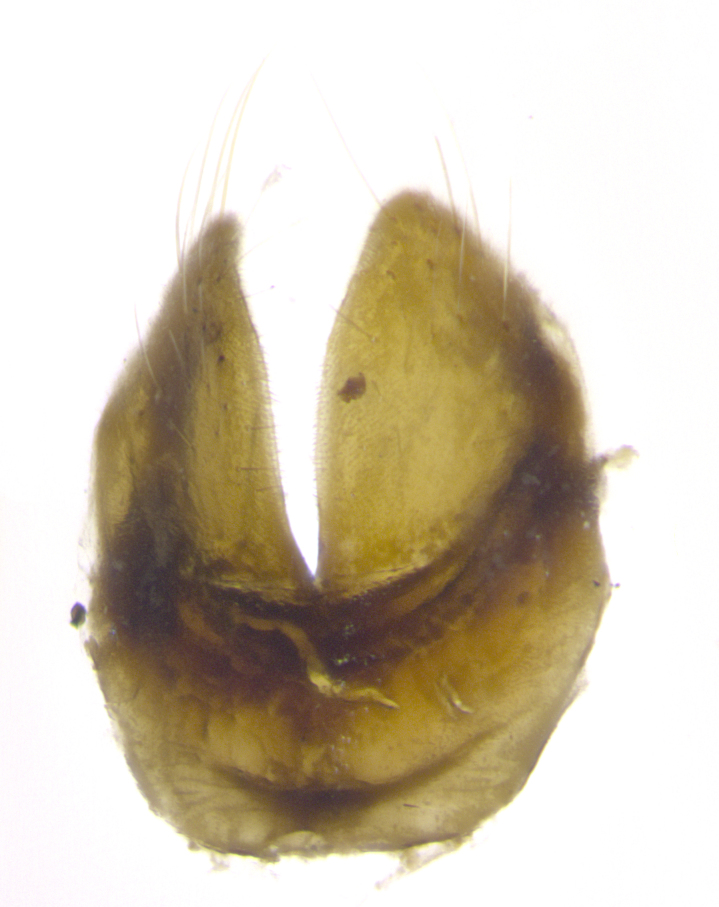
sternite 5,ventral view.

**Figure 10a.**
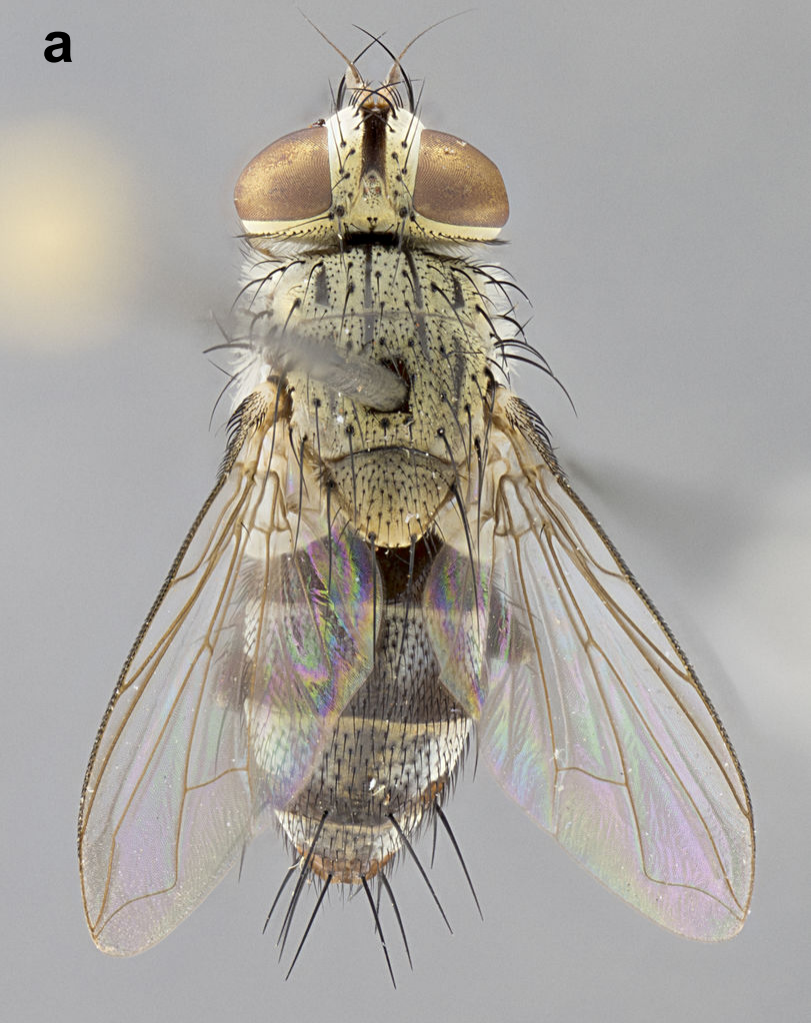
dorsal view.

**Figure 10b.**
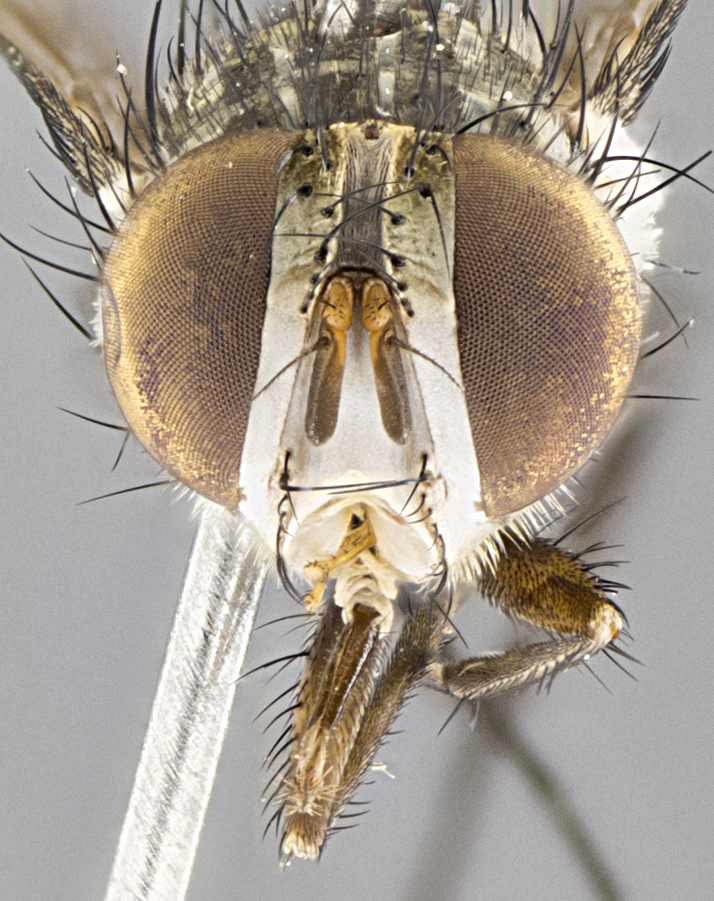
frontal view.

**Figure 10c.**
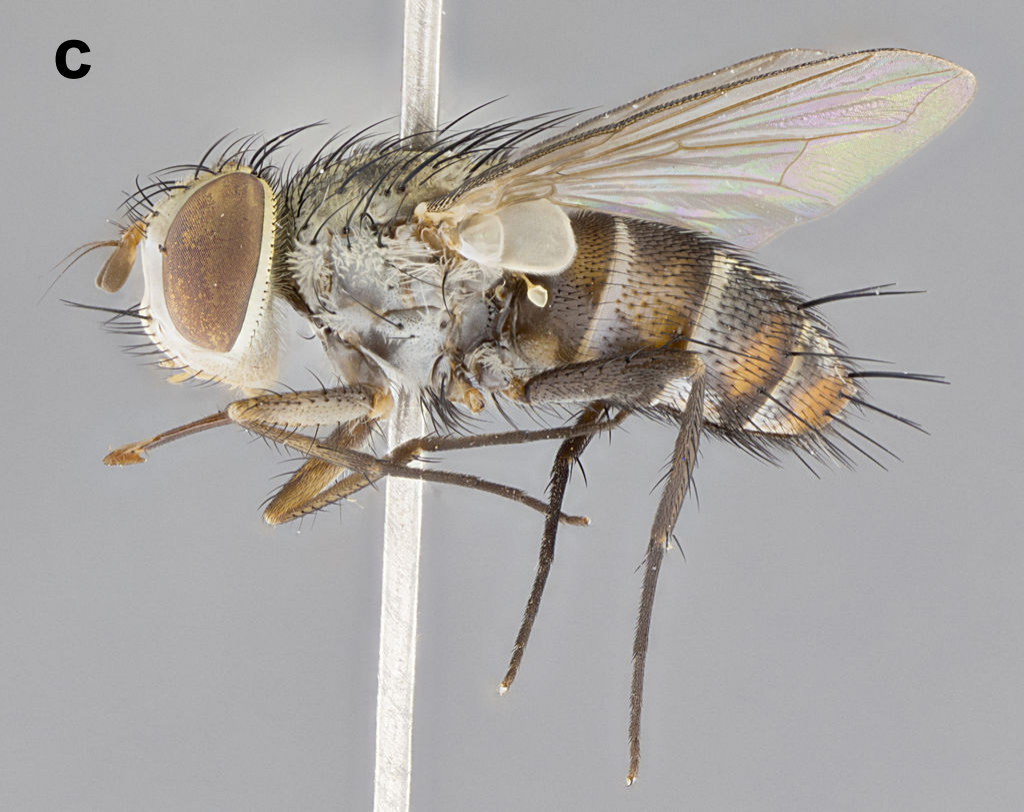
lateral view.

**Figure 11a.**
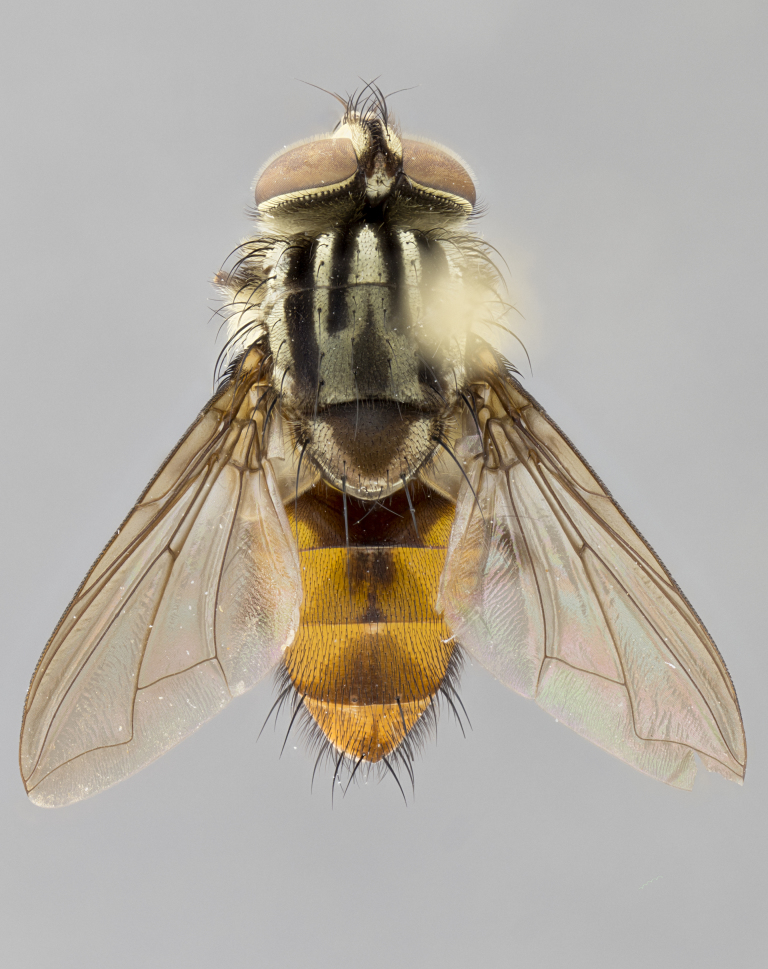
dorsal view.

**Figure 11b.**
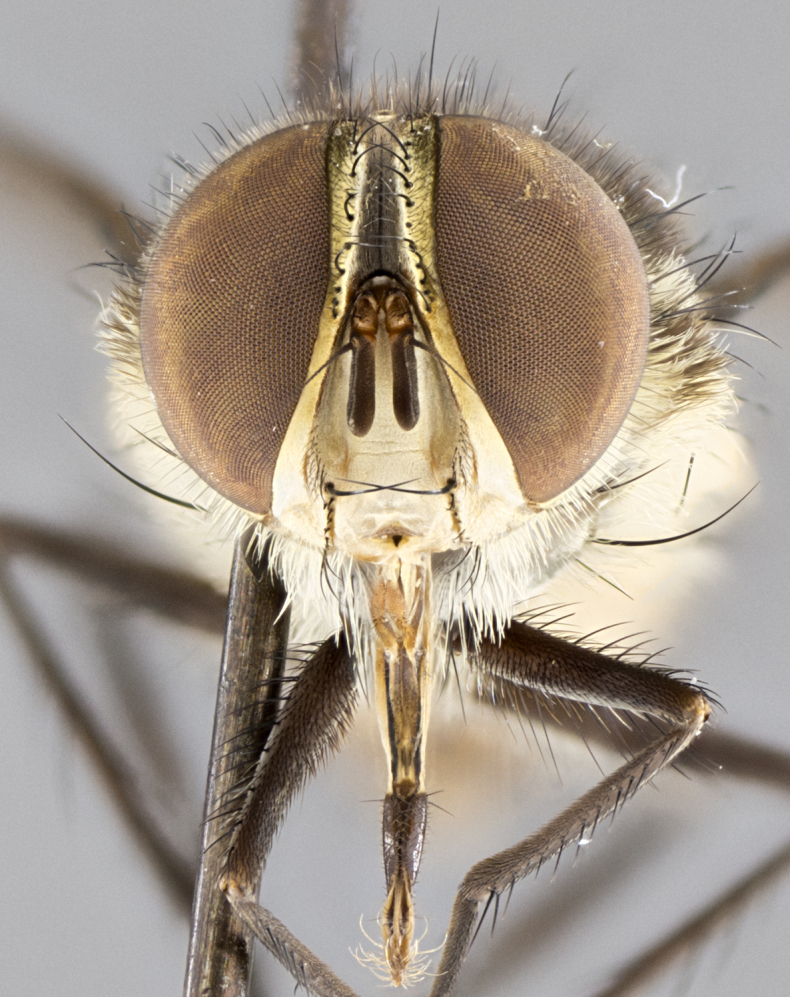
frontal view.

**Figure 11c.**
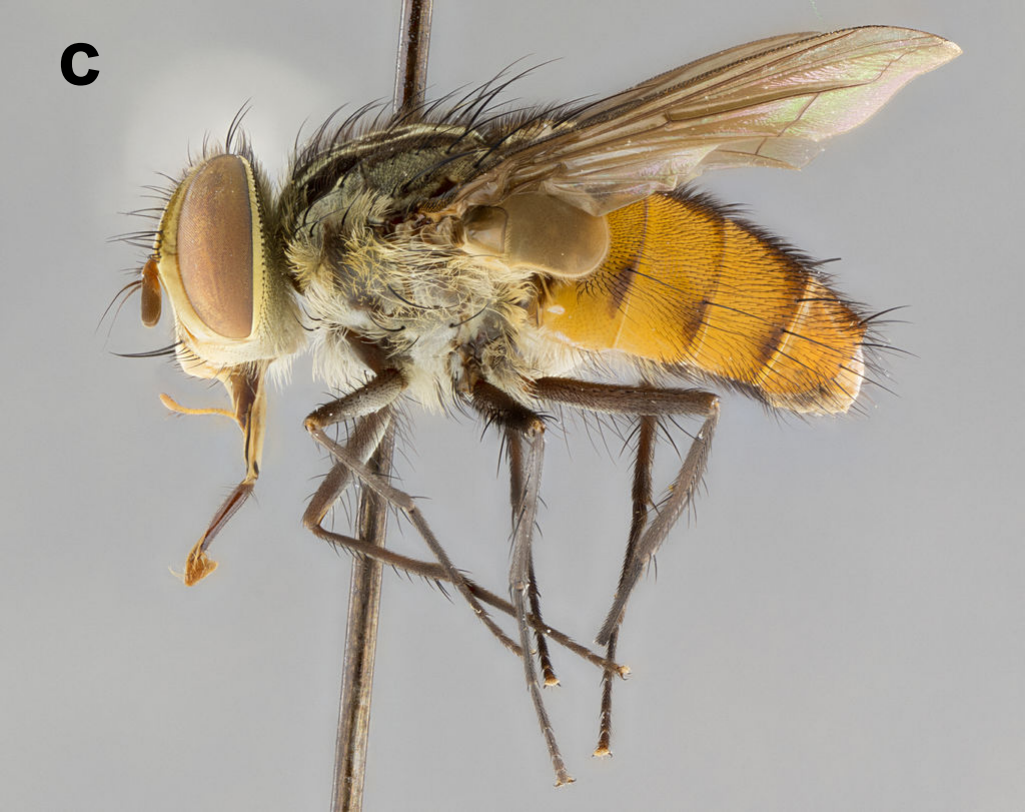
lateral view.

**Figure 11d.**
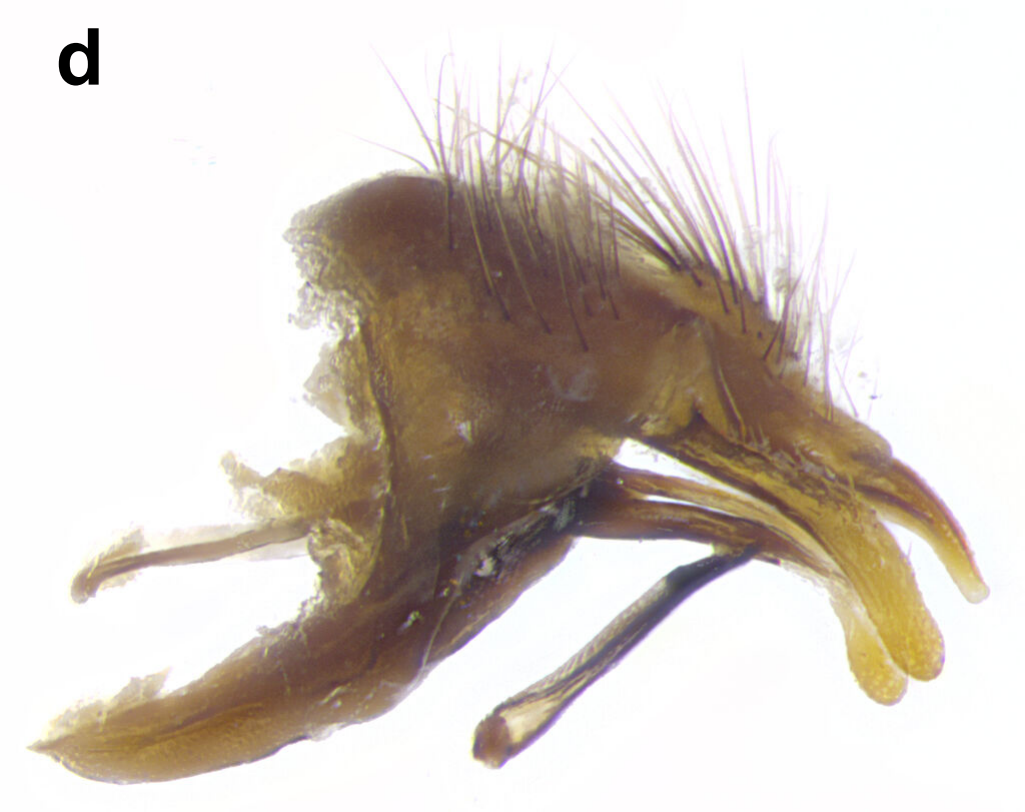
caudal view.

**Figure 11e.**
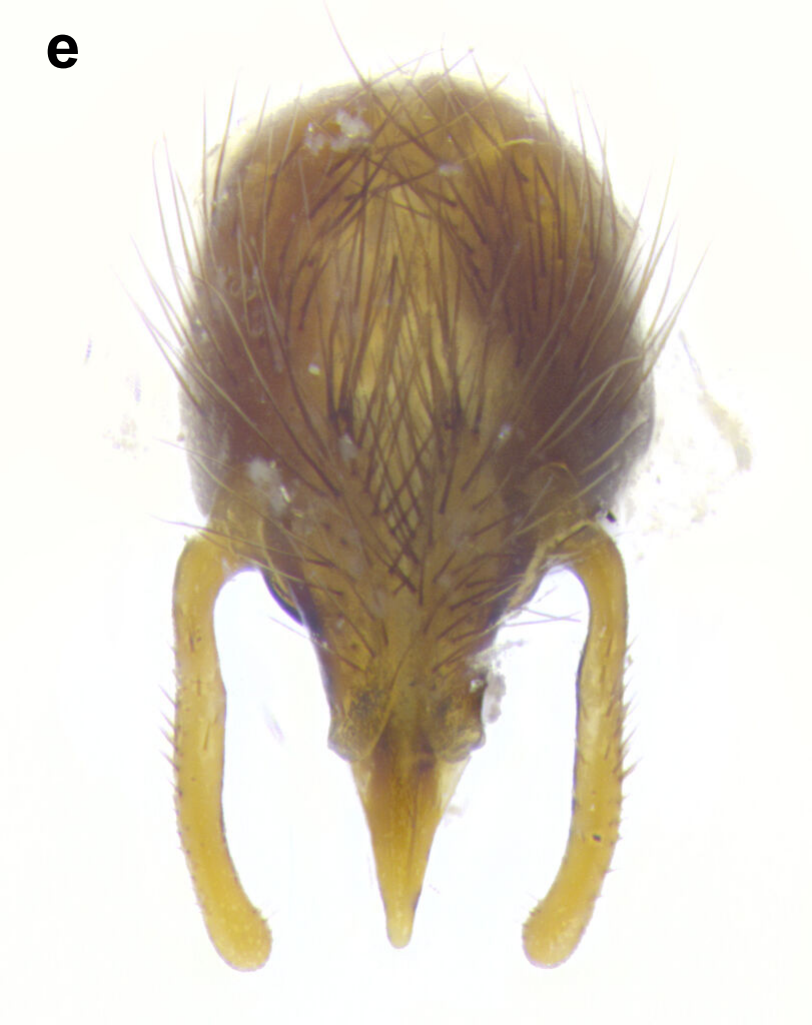
lateral view.

**Figure 11f.**
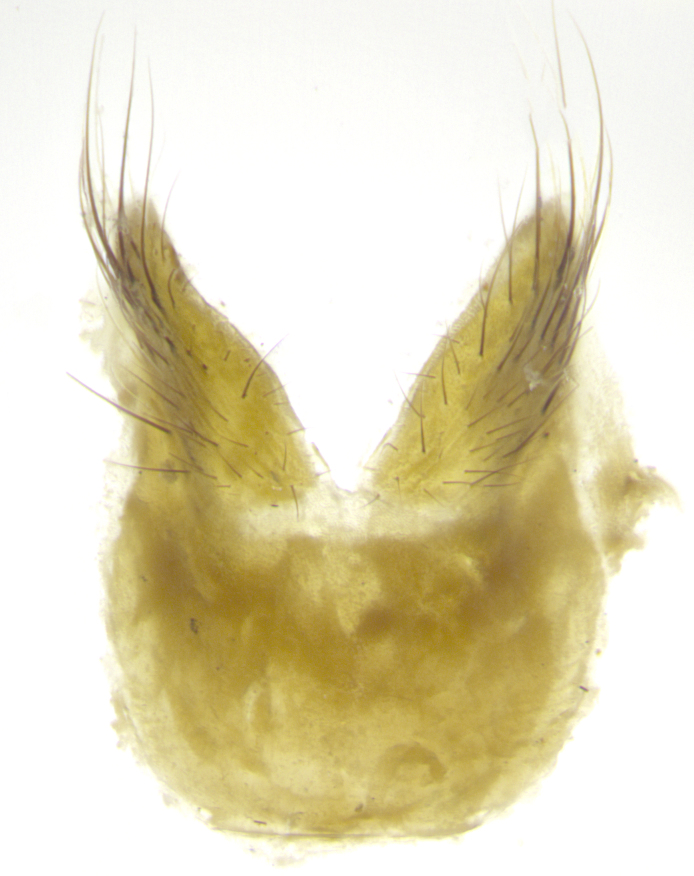
sternite 5, ventral view.

**Figure 12a.**
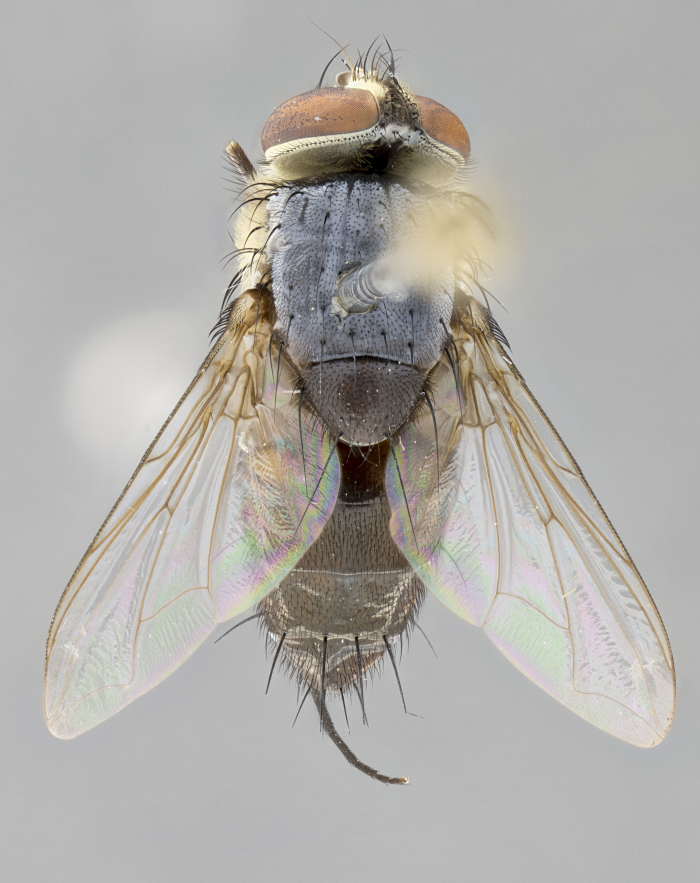
dorsal view.

**Figure 12b.**
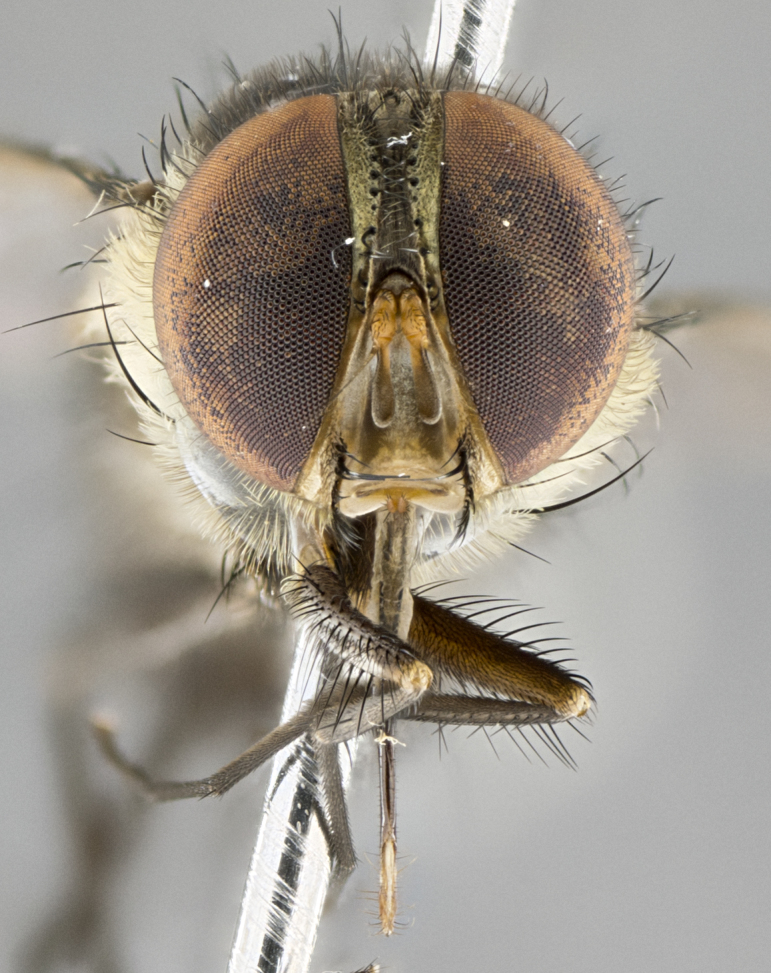
frontal view.

**Figure 12c.**
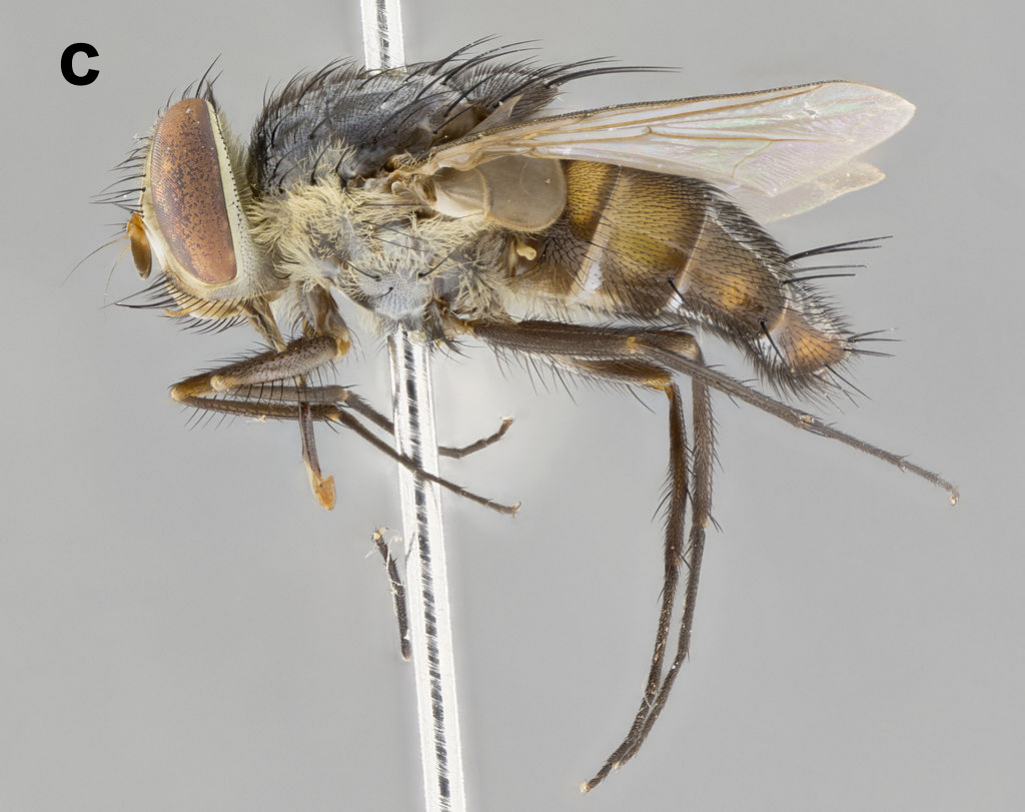
lateral view.

**Figure 12d.**
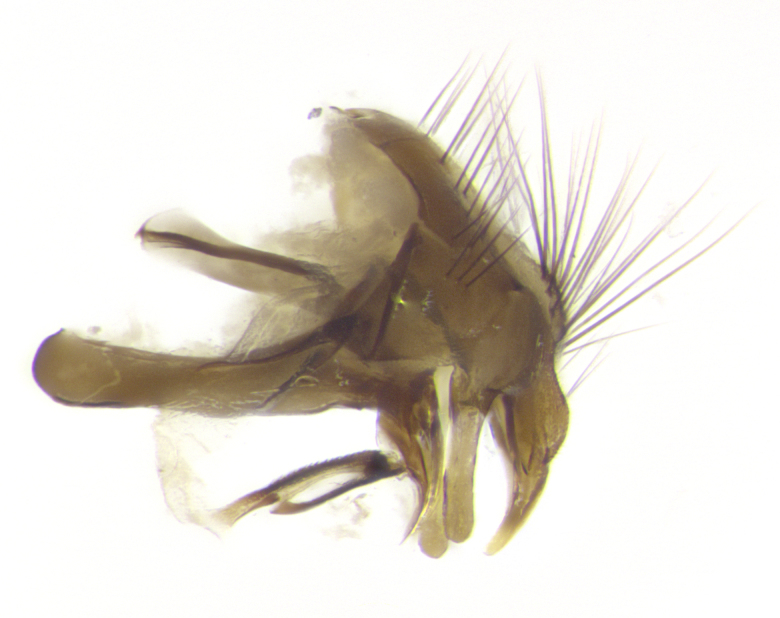
lateral view.

**Figure 12e.**
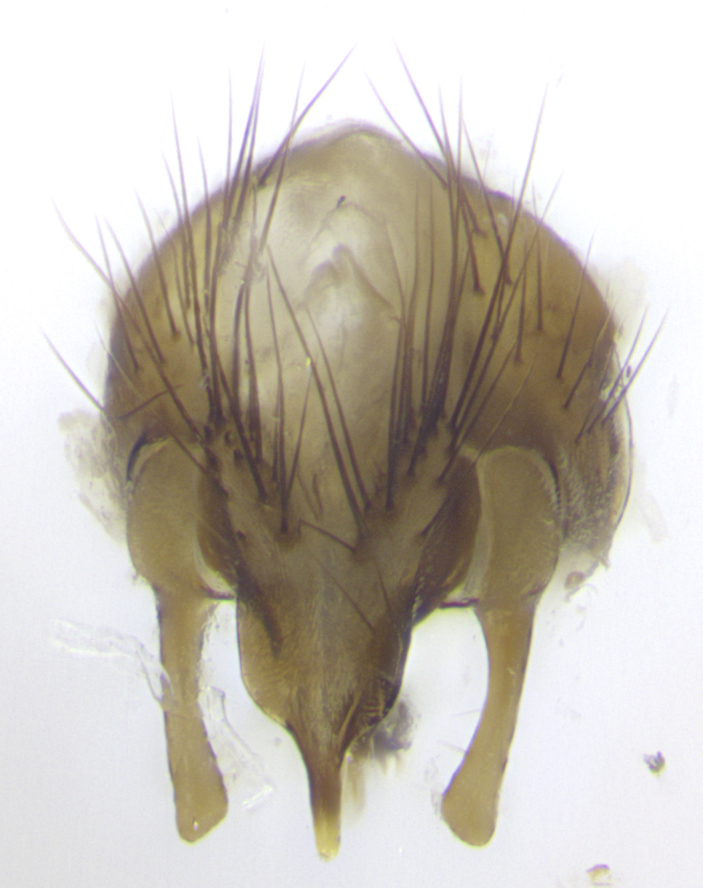
caudal view.

**Figure 12f.**
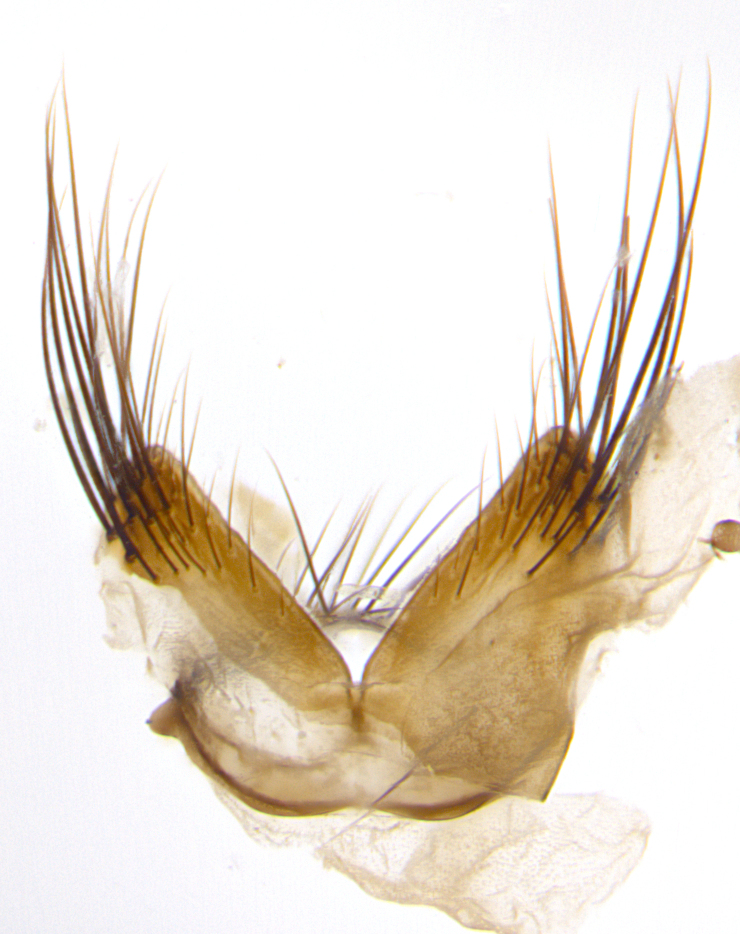
sternite 5, ventral view.

**Figure 13a.**
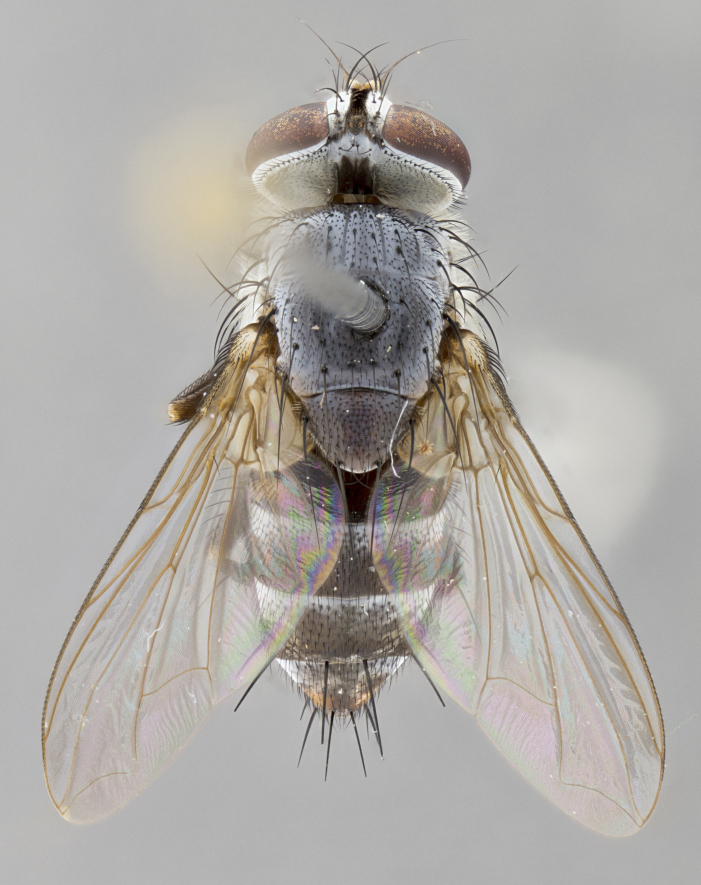
dorsal view

**Figure 13b.**
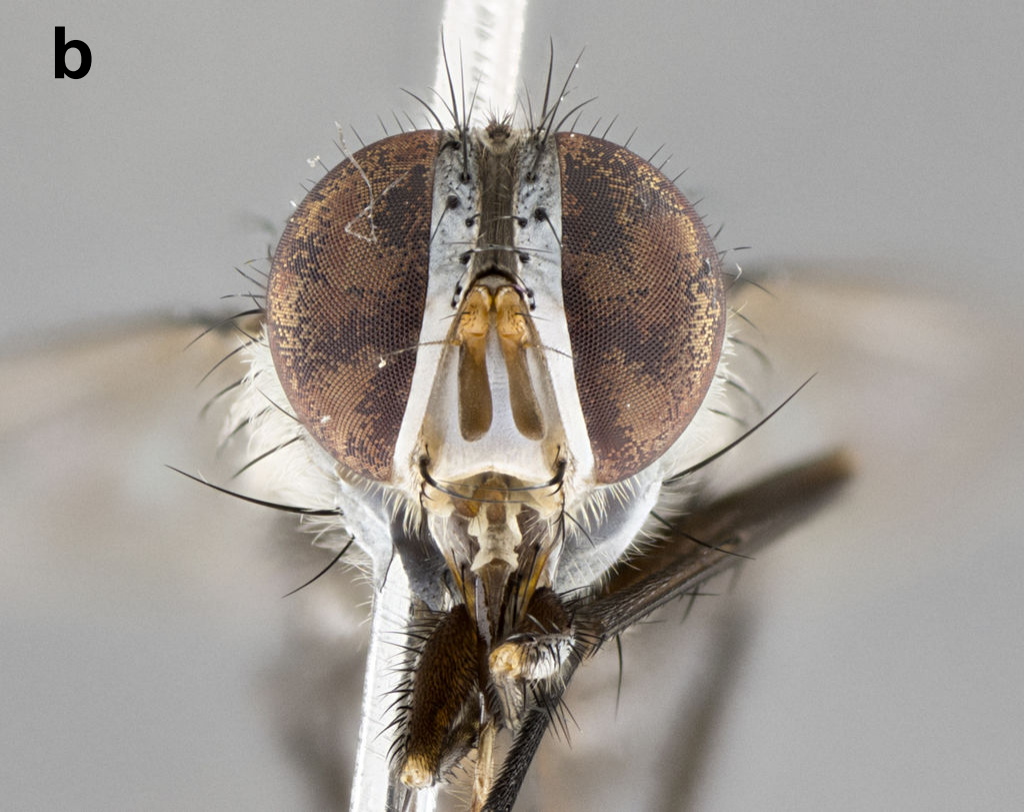
frontal view

**Figure 13c.**
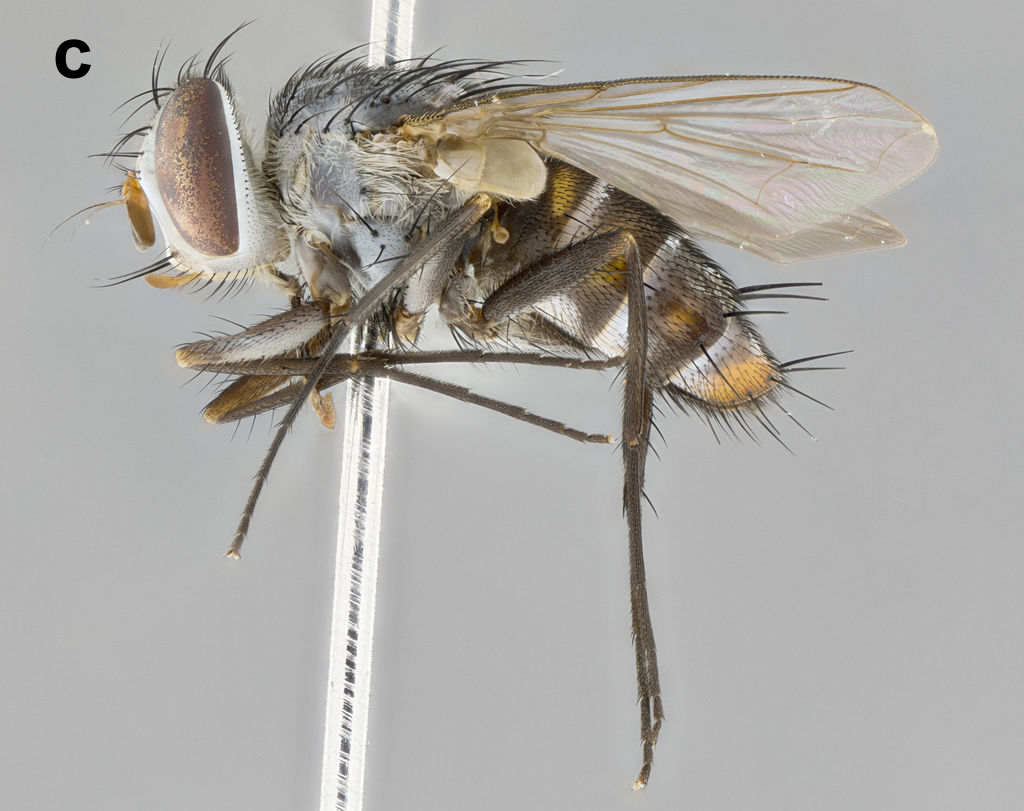
lateral view

**Figure 14a.**
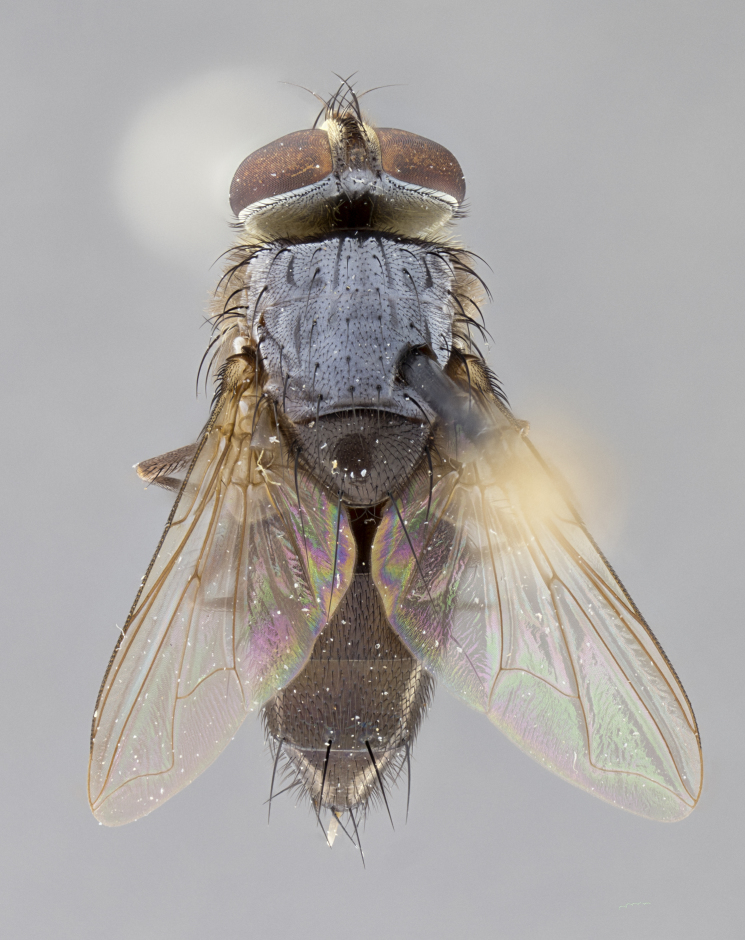
dorsal view.

**Figure 14b.**
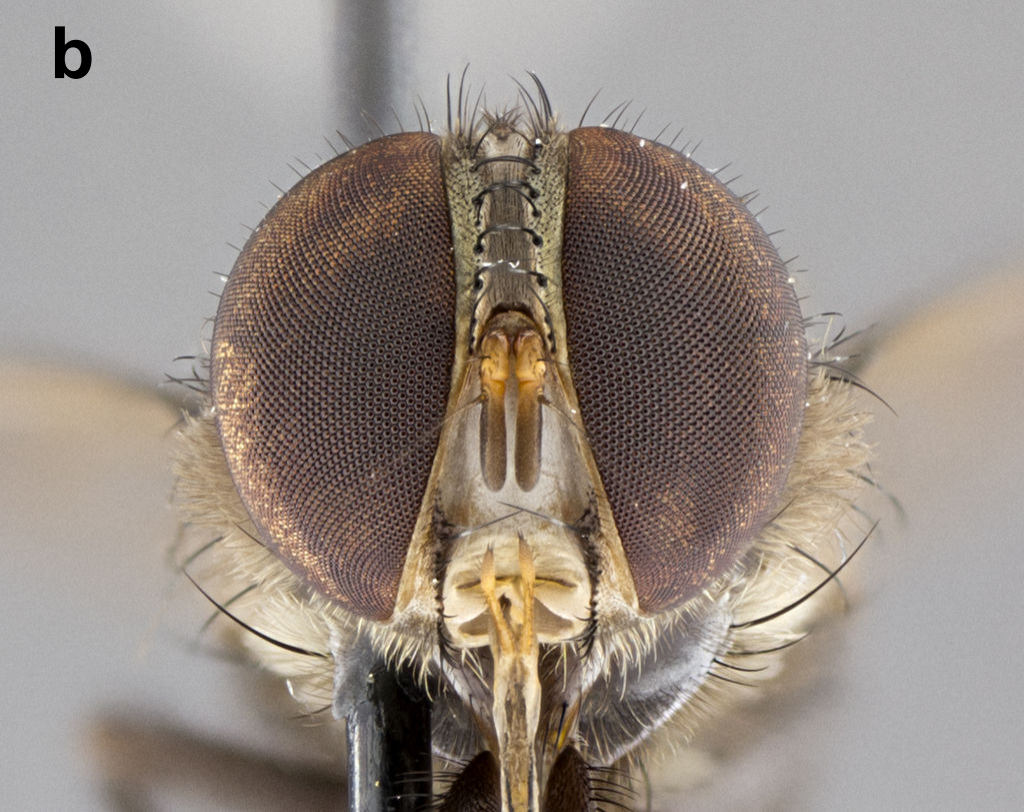
frontal view.

**Figure 14c.**
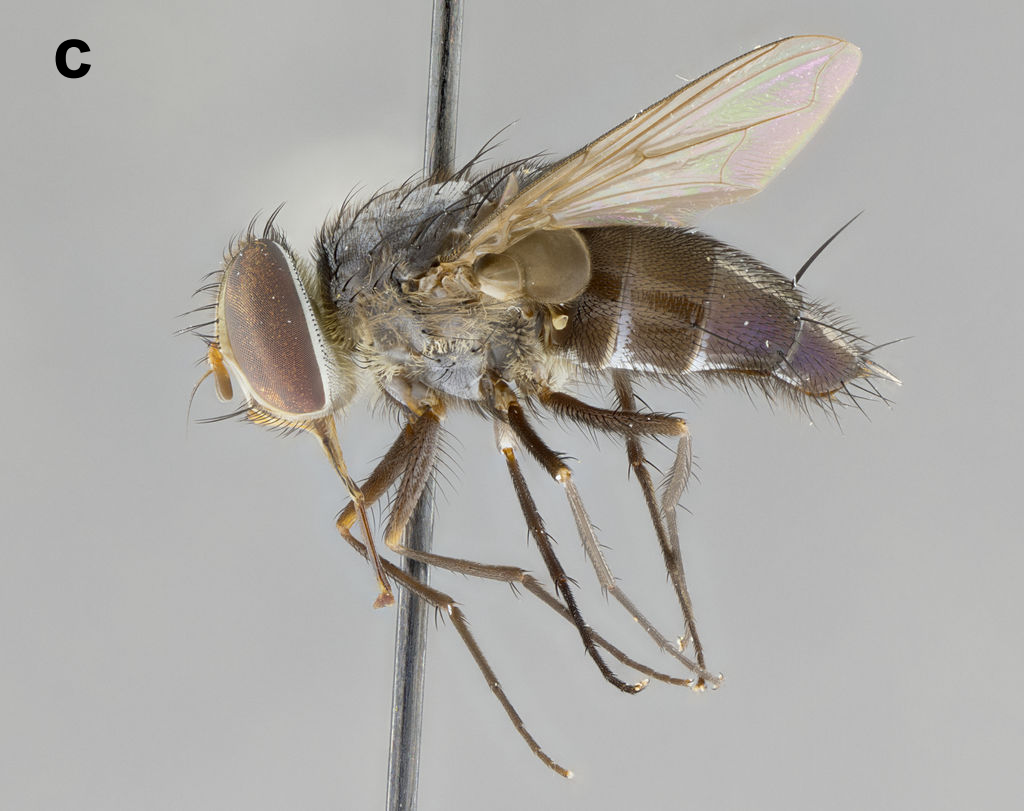
lateral view.

**Figure 15a.**
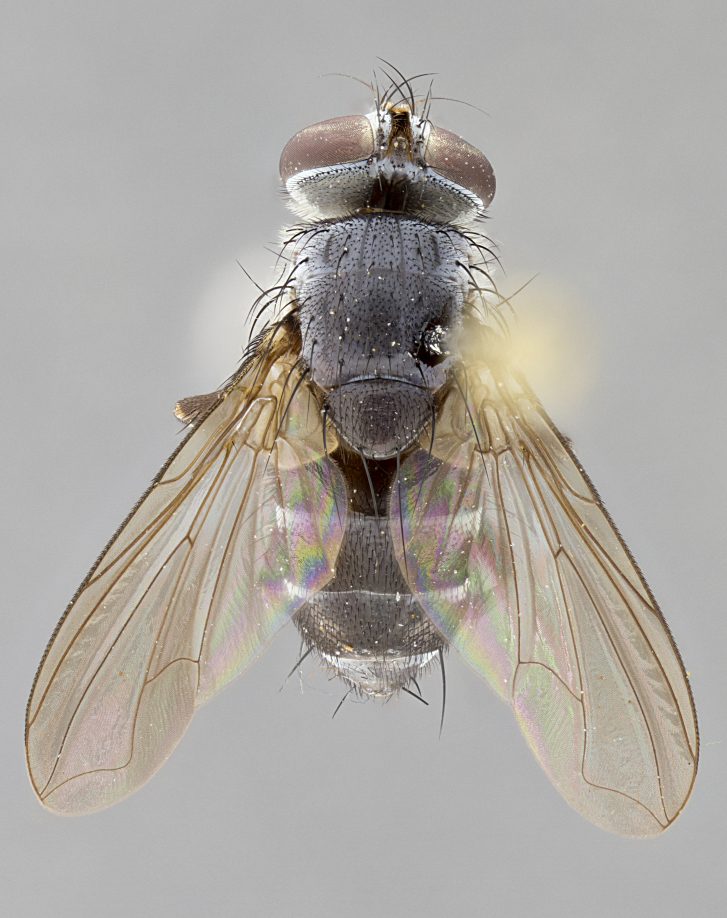
dorsal view.

**Figure 15b.**
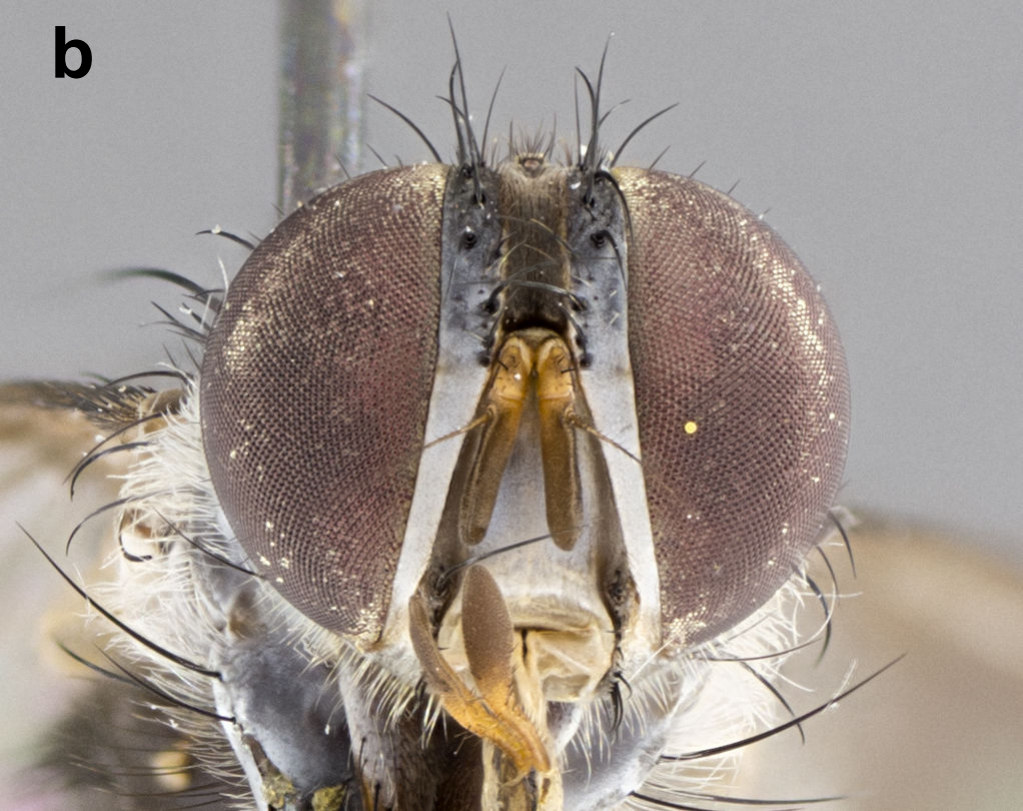
frontal view.

**Figure 15c.**
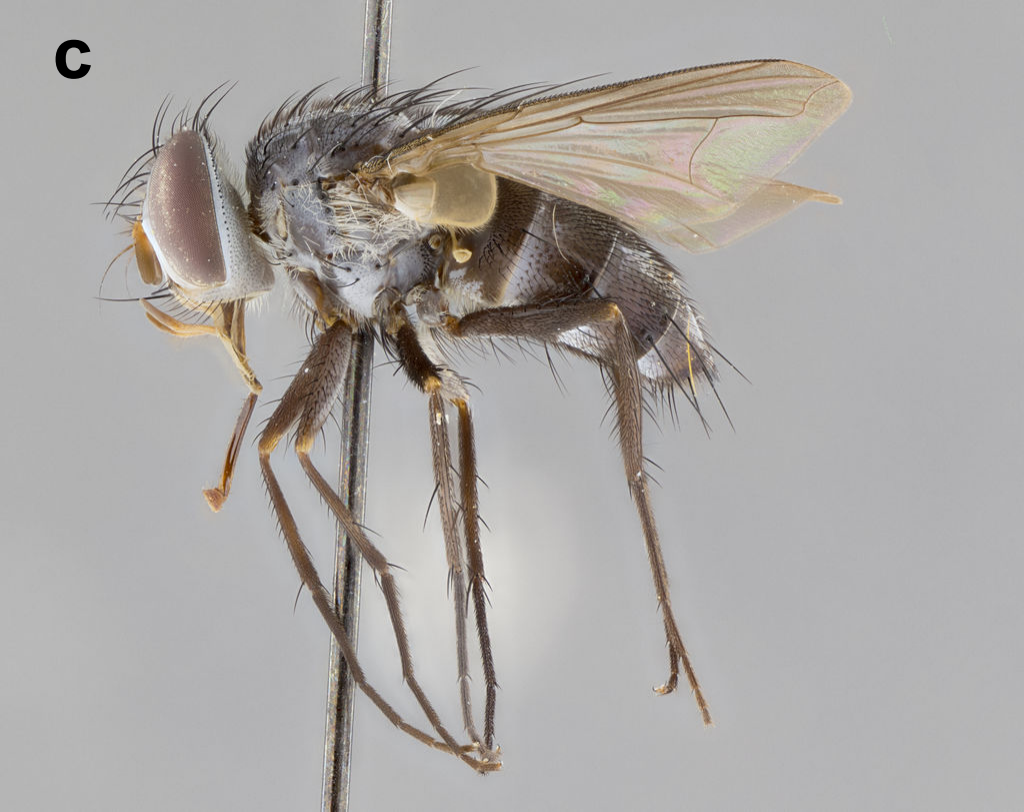
lateral view.

**Figure 16a.**
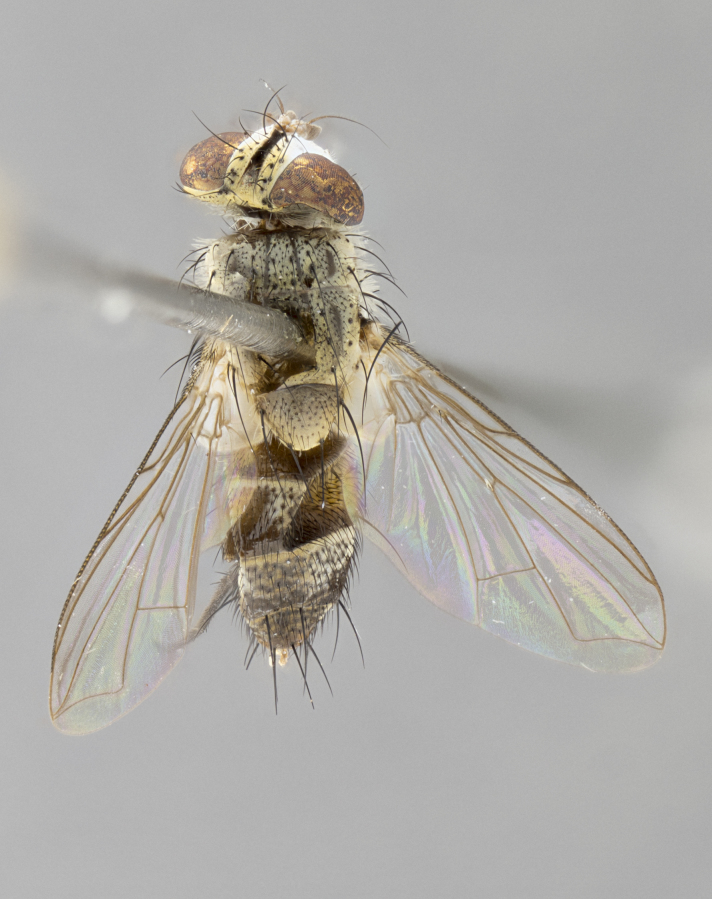
dorsal view.

**Figure 16b.**
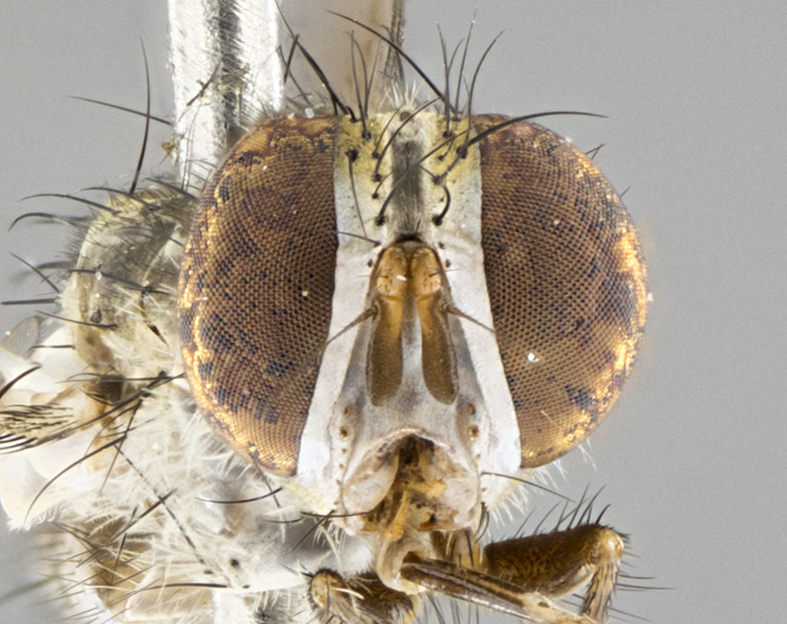
frontal view.

**Figure 16c.**
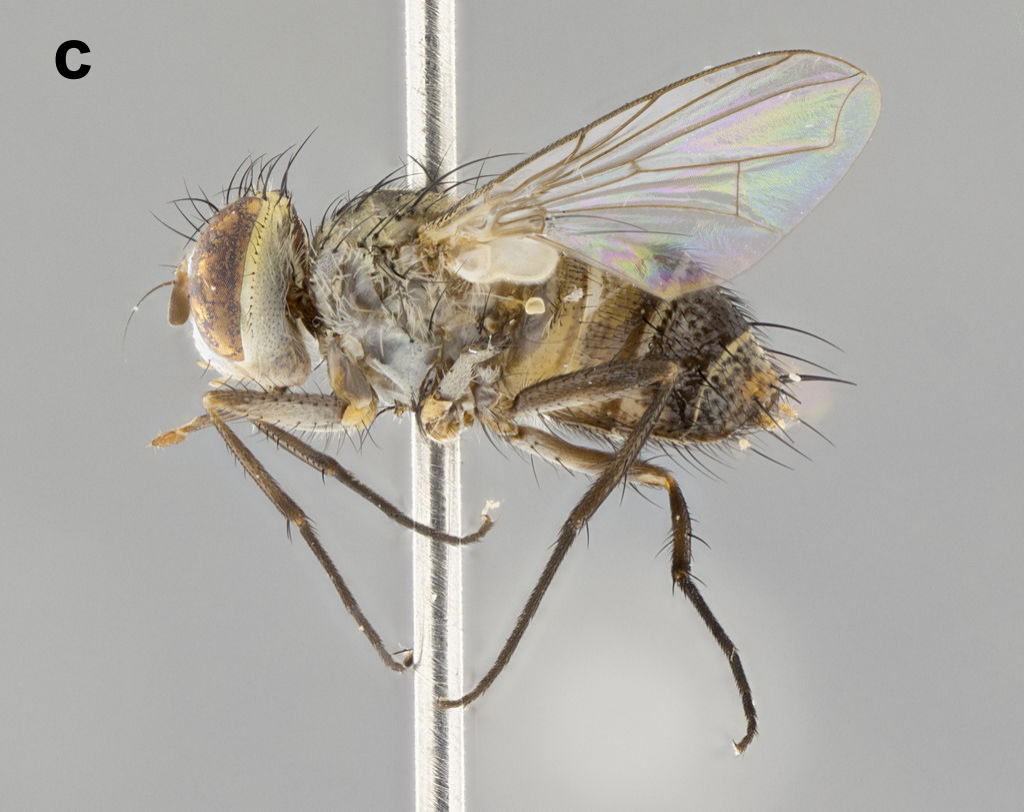
lateral view.

**Figure 17a.**
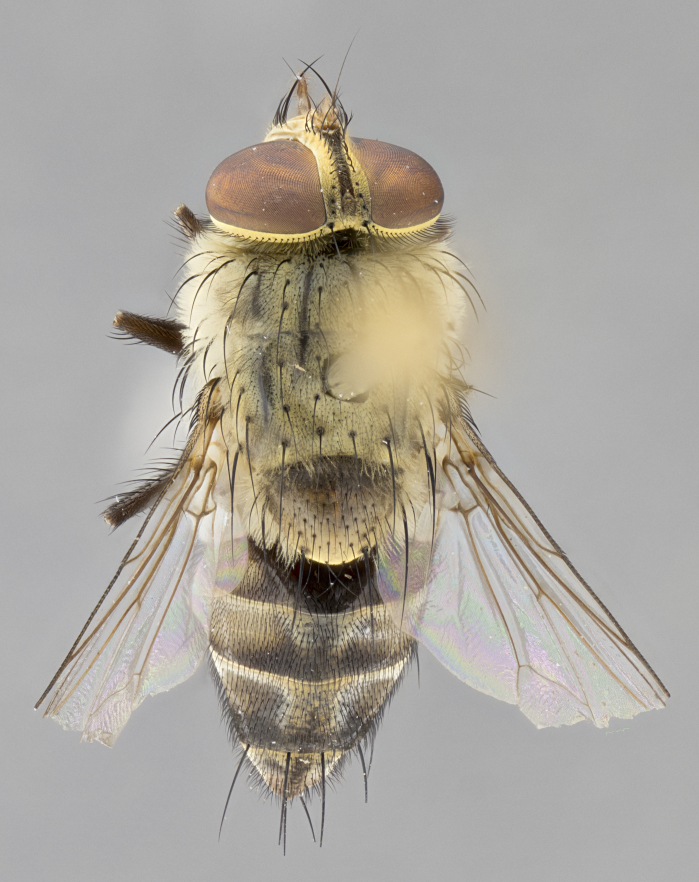
dorsal view.

**Figure 17b.**
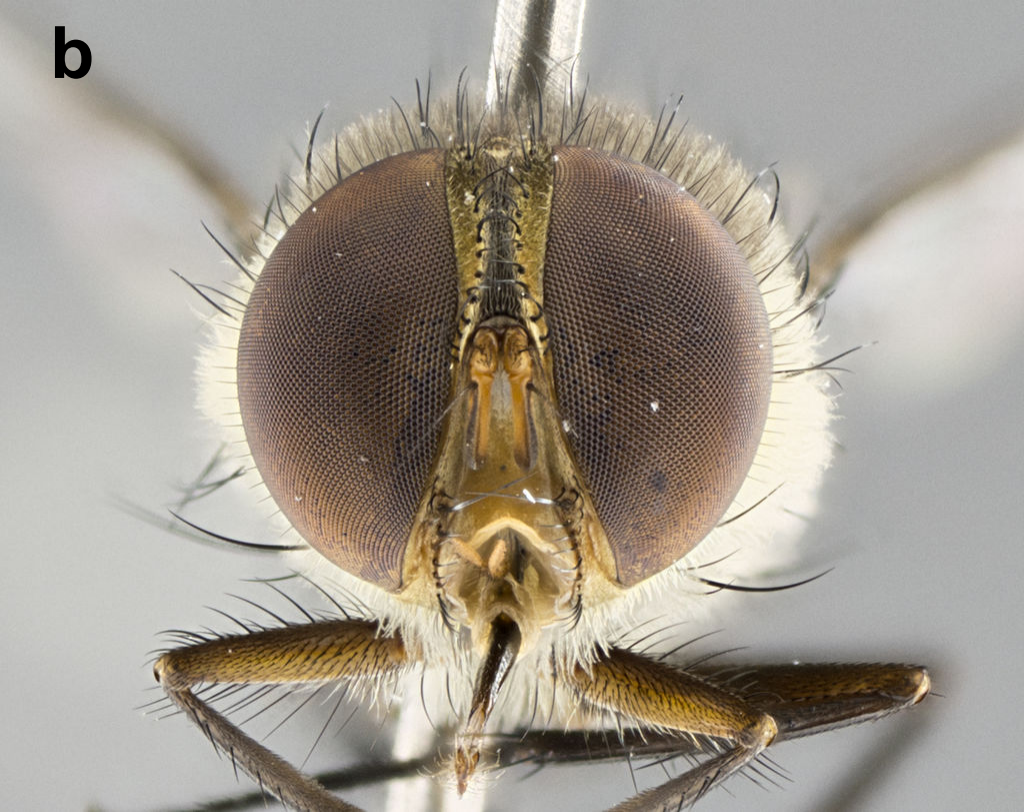
frontal view.

**Figure 17c.**
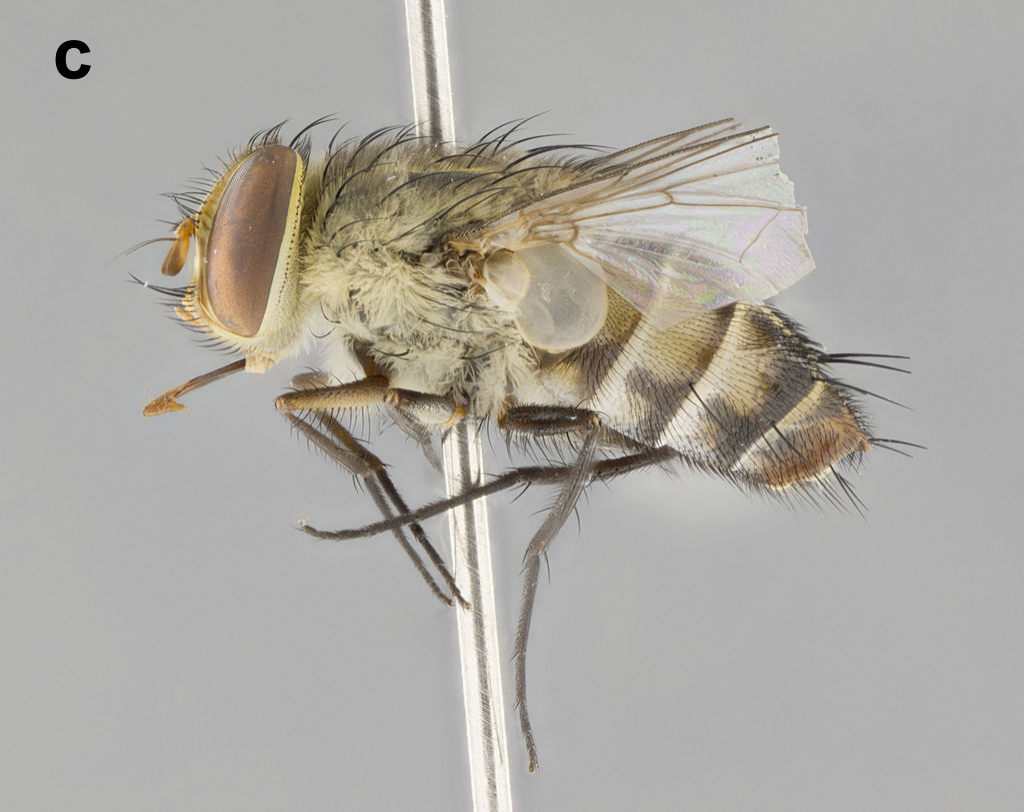
lateral view.

**Figure 18a.**
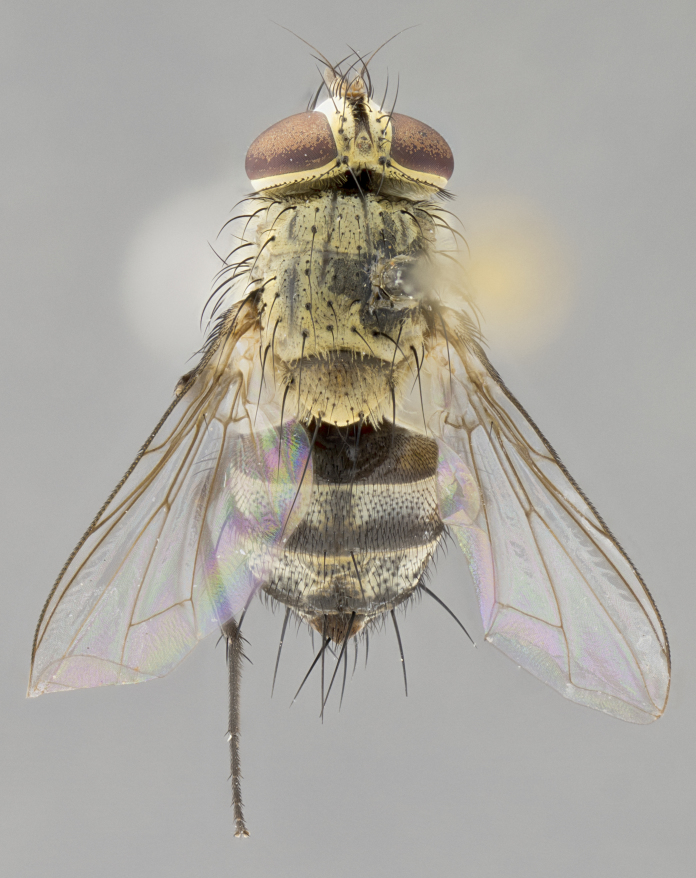
dorsal view.

**Figure 18b.**
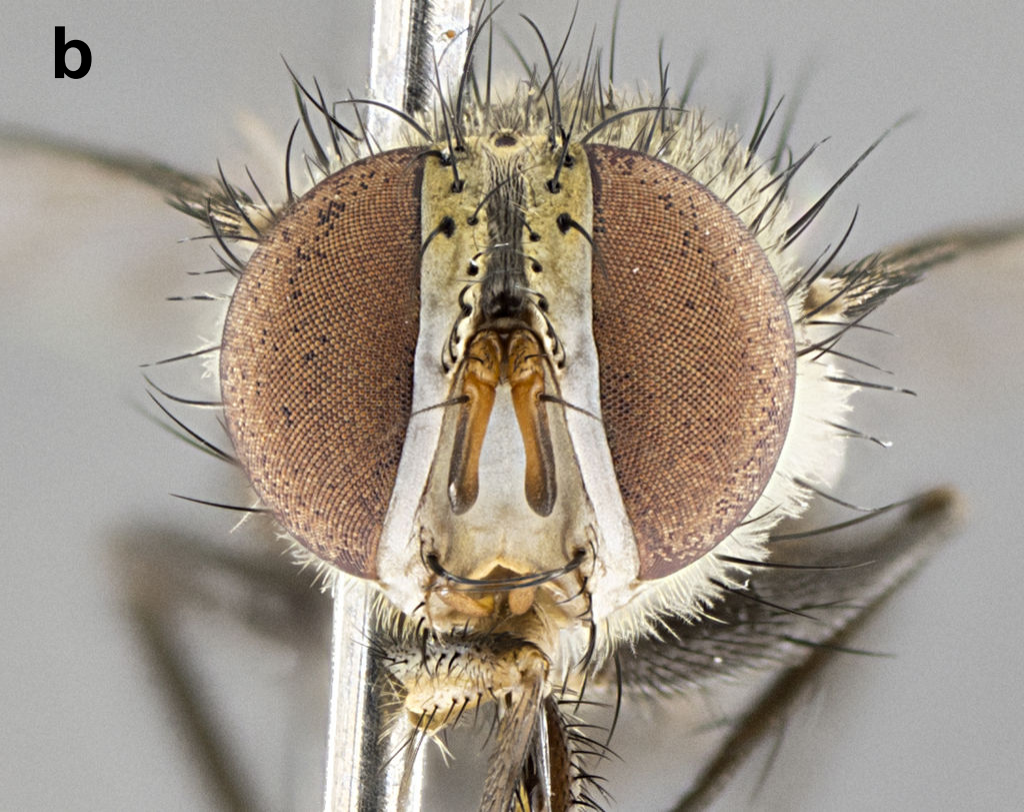
frontal view.

**Figure 18c.**
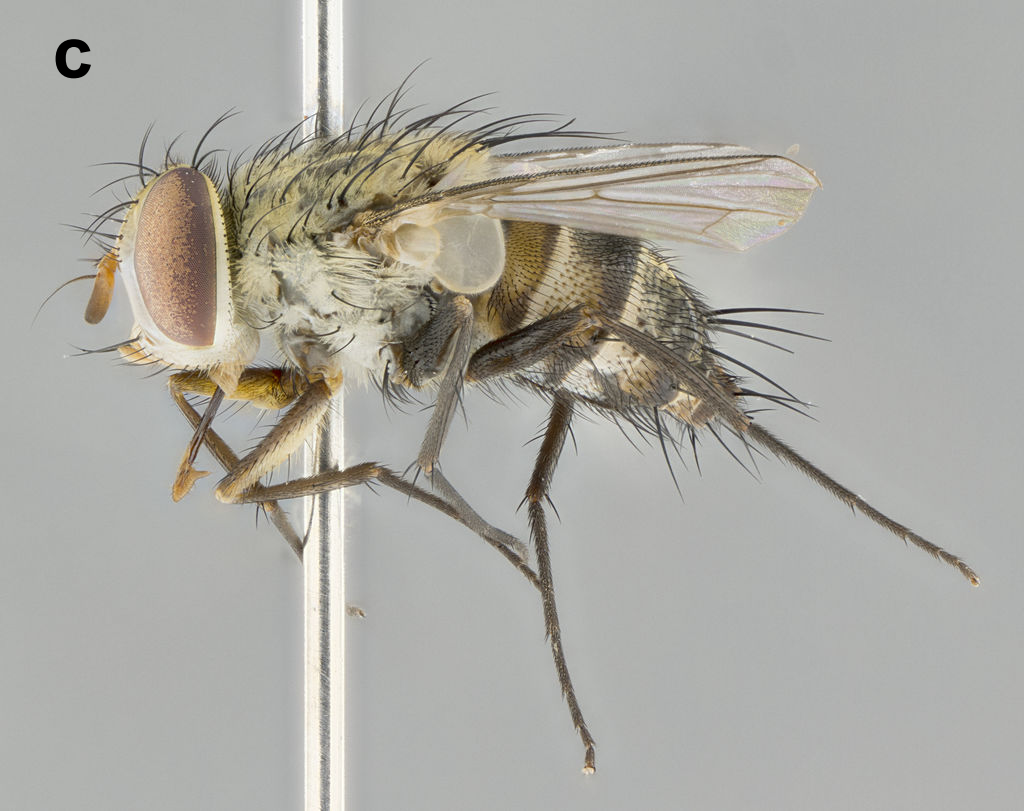
lateral view.

**Figure 19a.**
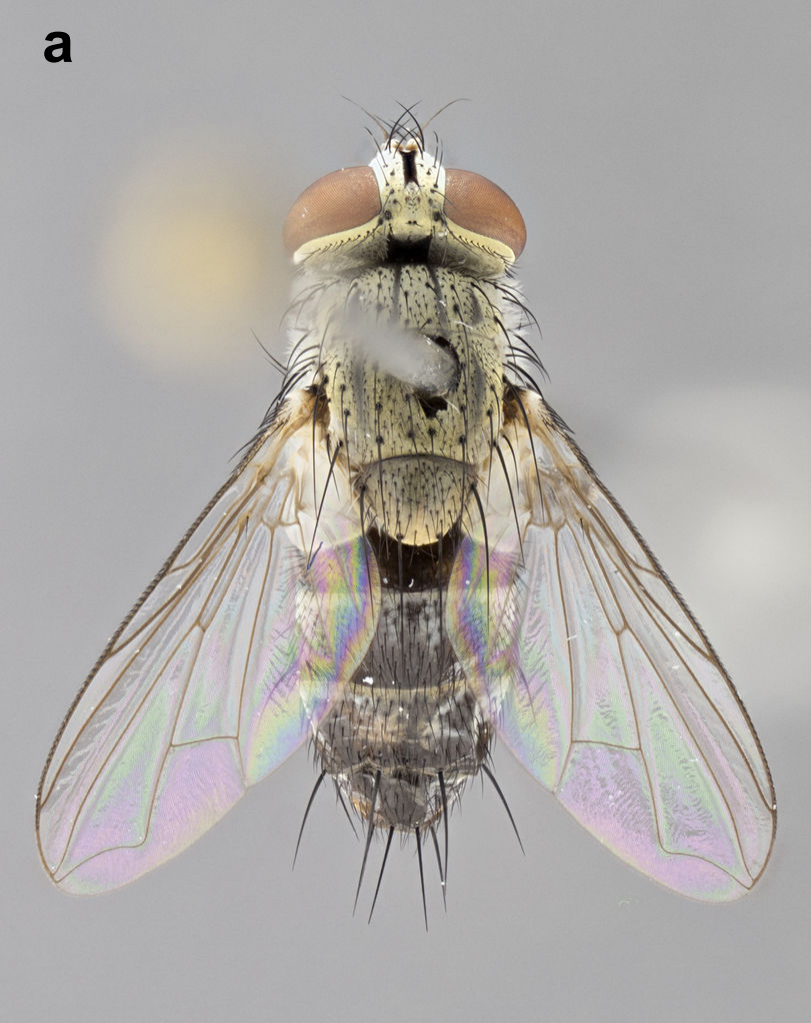
dorsal view.

**Figure 19b.**
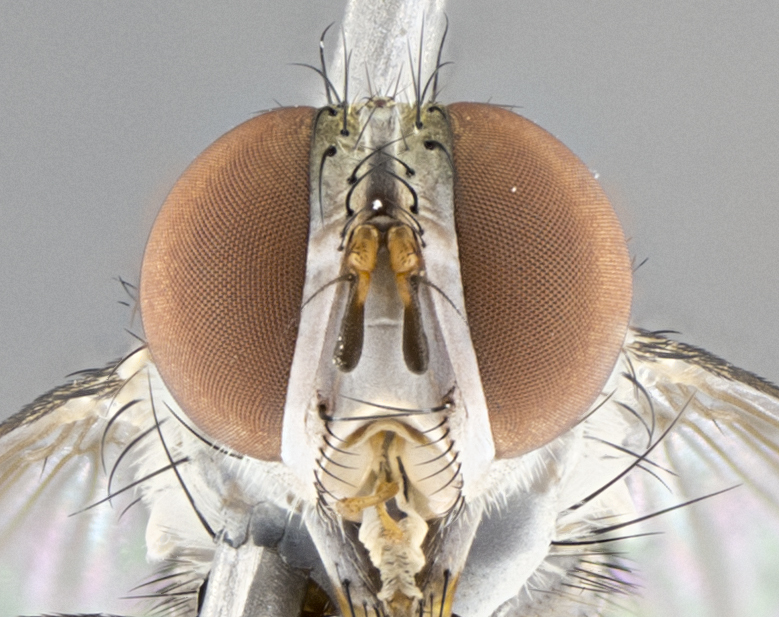
frontal view.

**Figure 19c.**
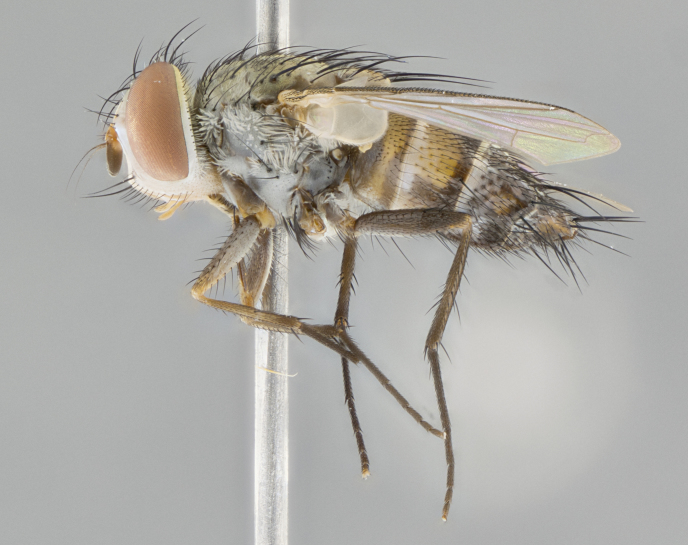
lateral view.

**Figure 20a.**
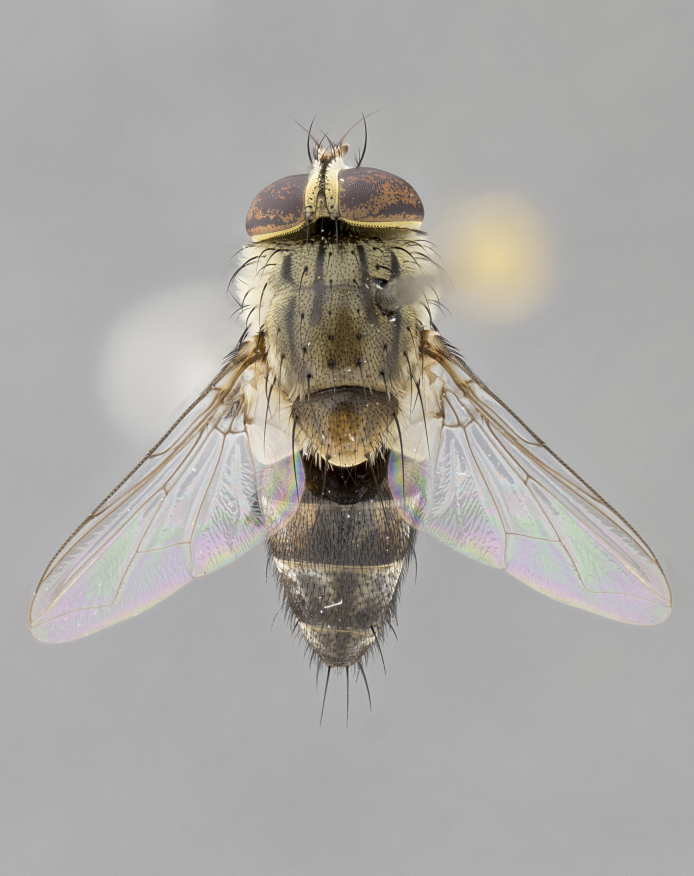
dorsal view.

**Figure 20b.**
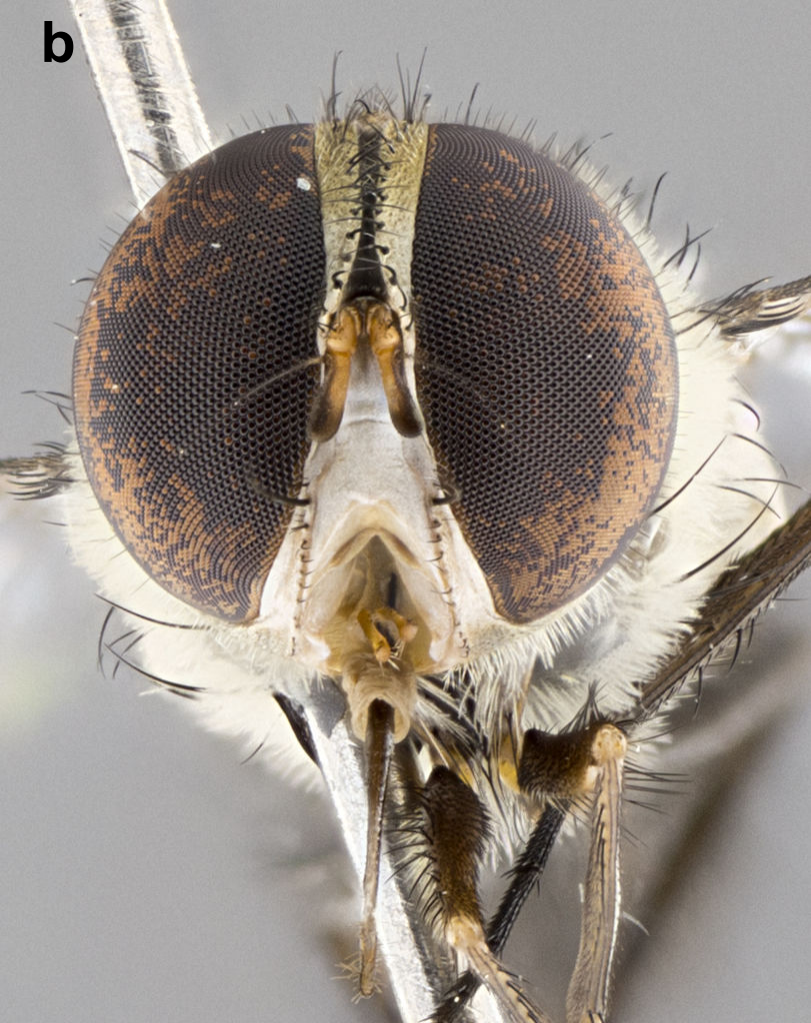
frontal view.

**Figure 20c.**
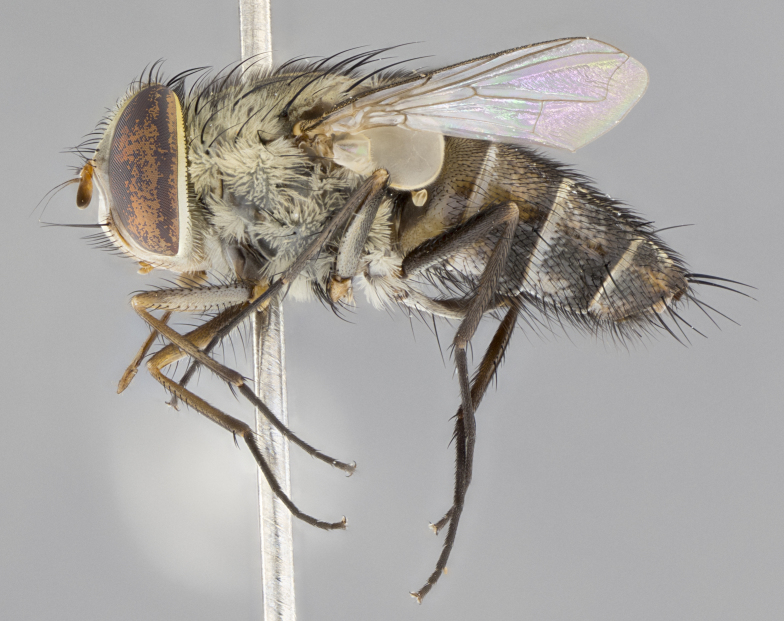
lateral view.

**Figure 20d.**
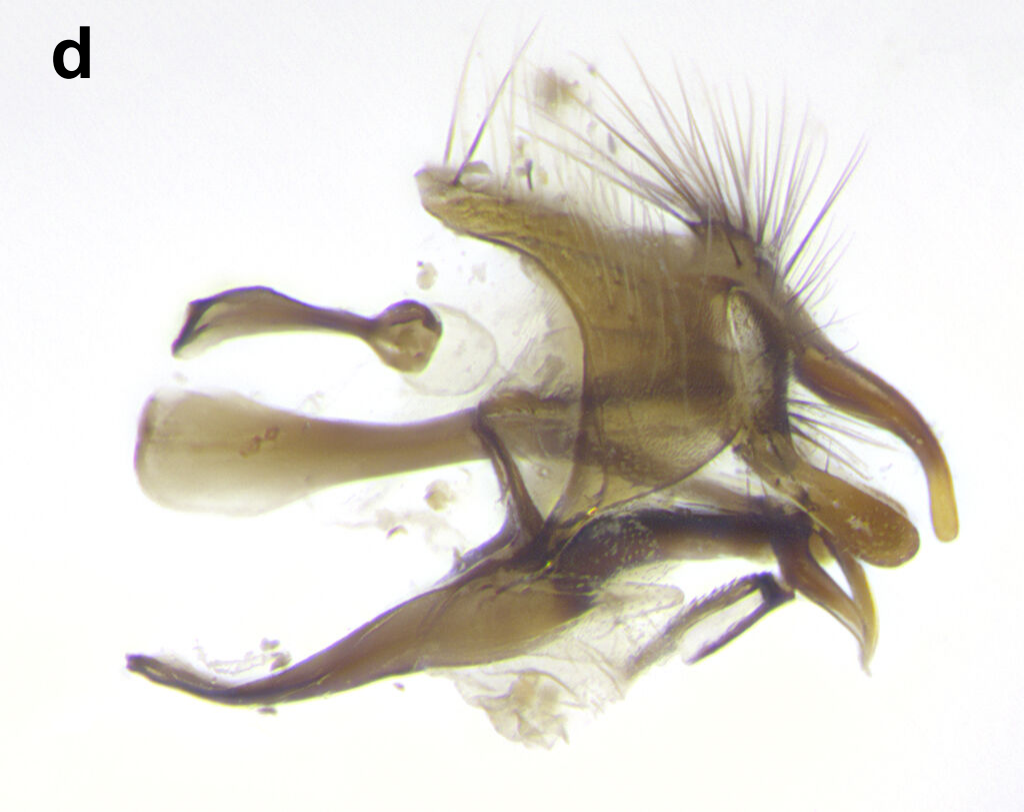
lateral view.

**Figure 20e.**
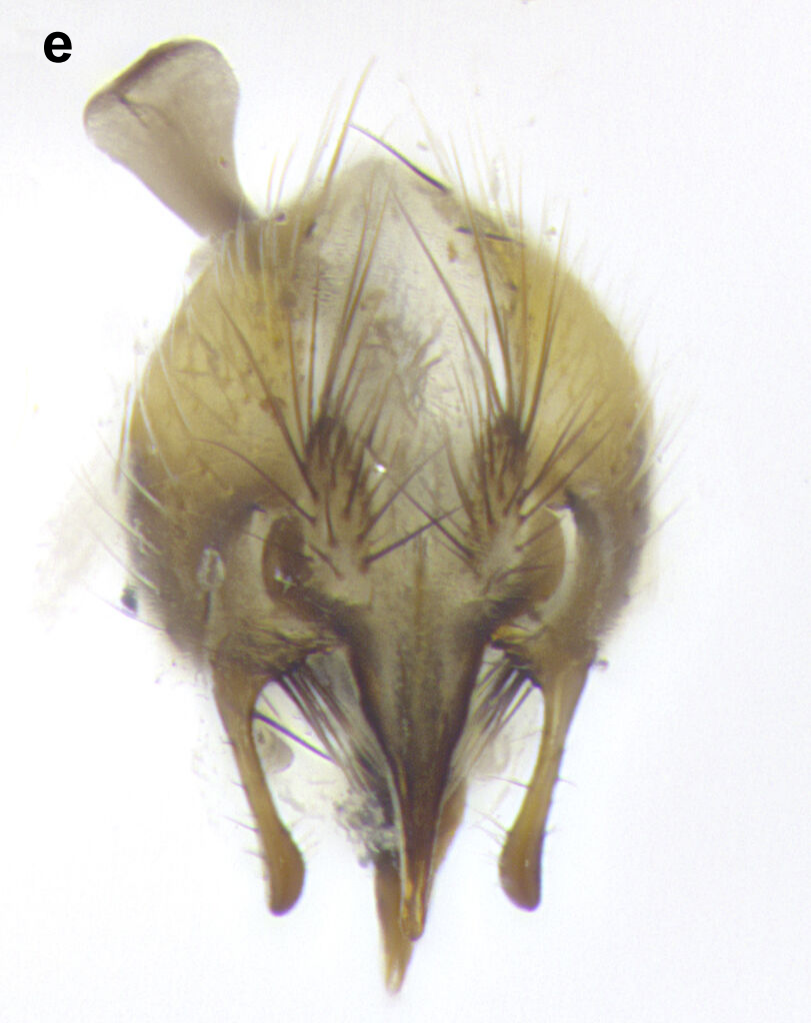
caudal view.

**Figure 20f.**
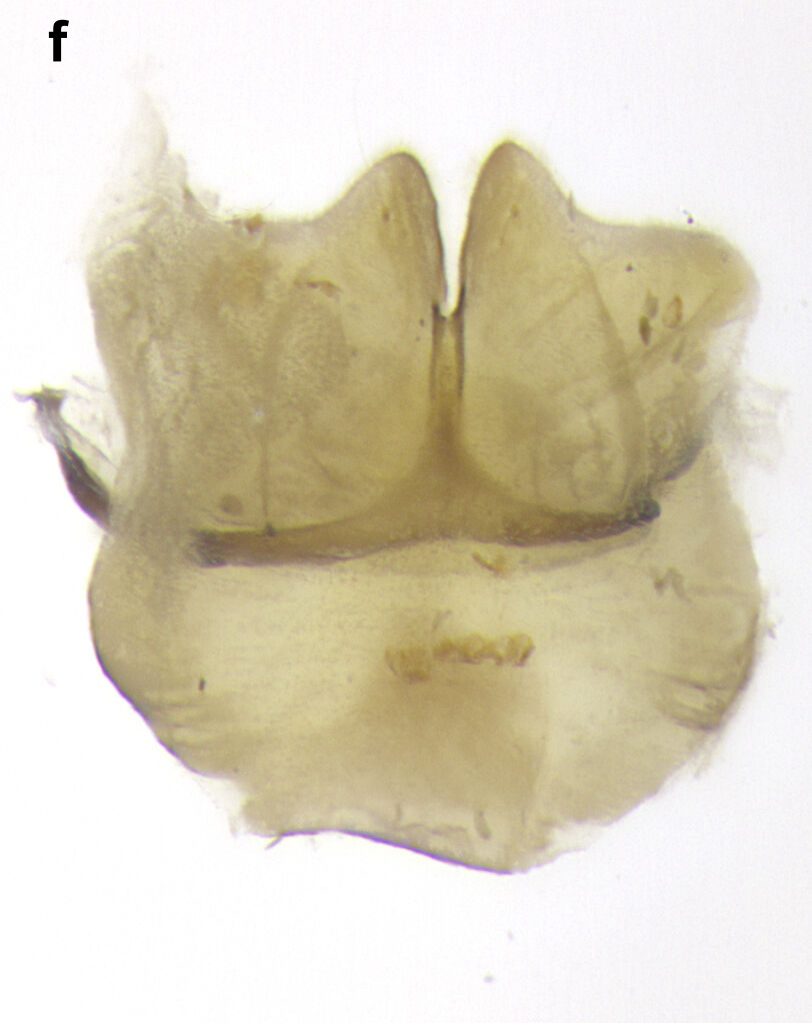
sternite 5, ventral view.

**Figure 21a.**
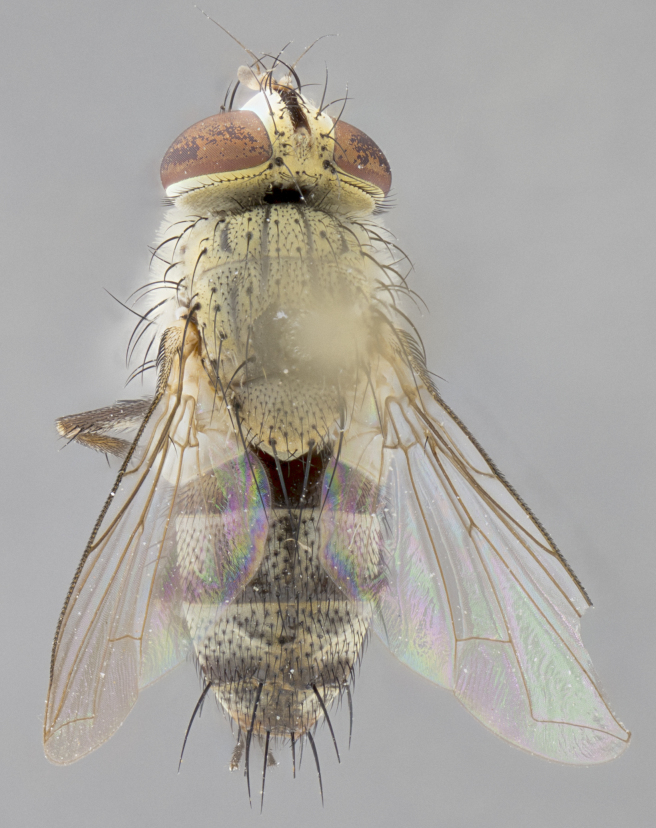
dorsal view.

**Figure 21b.**
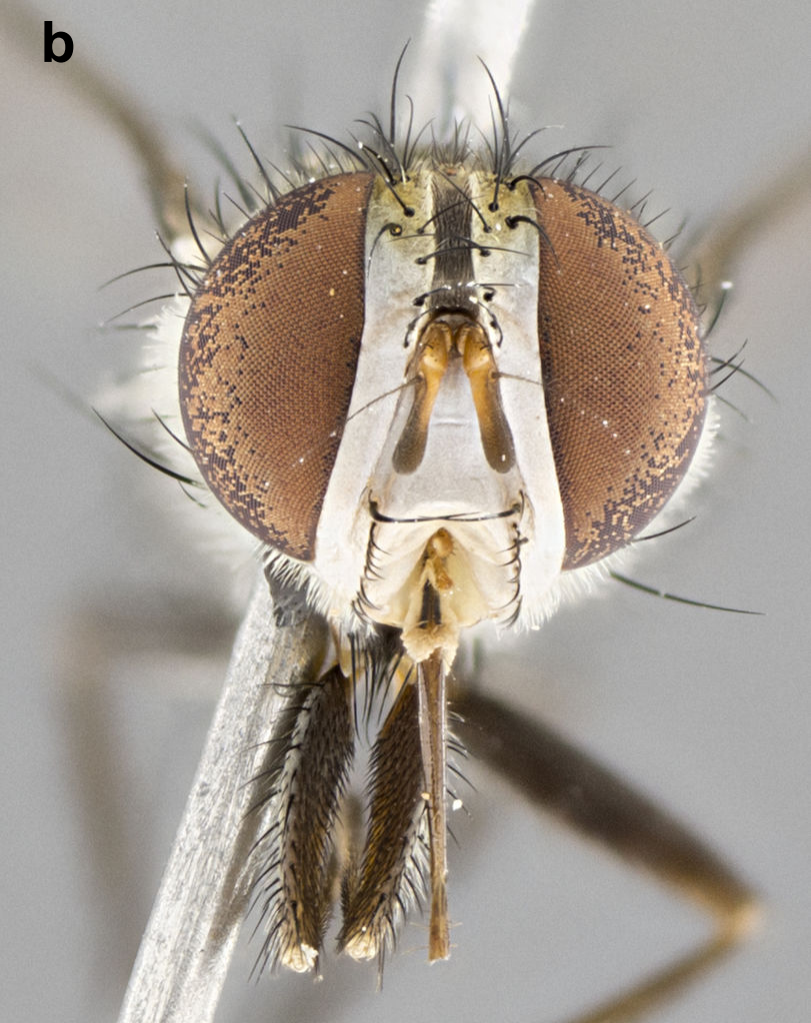
frontal view.

**Figure 21c.**
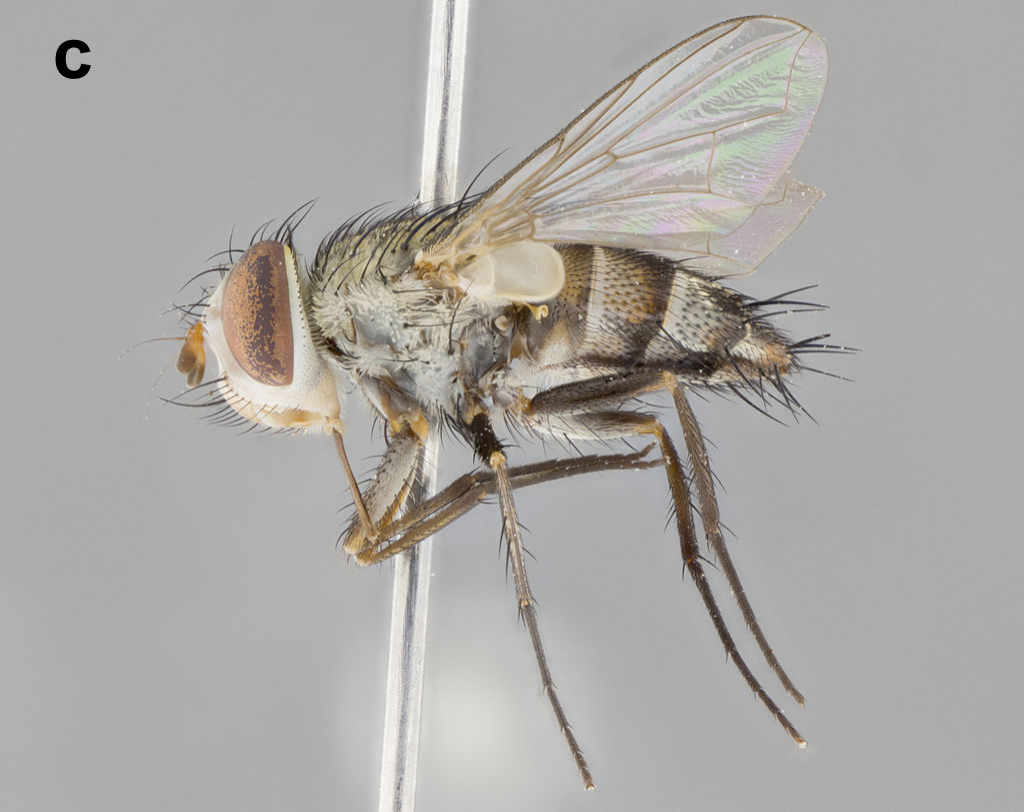
lateral view.

**Figure 22a.**
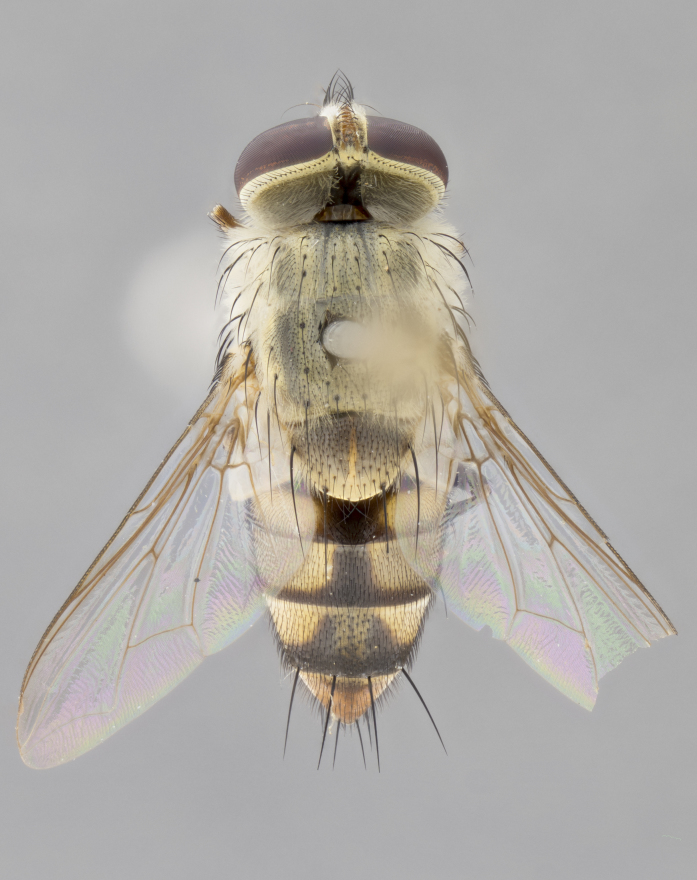
dorsal view.

**Figure 22b.**
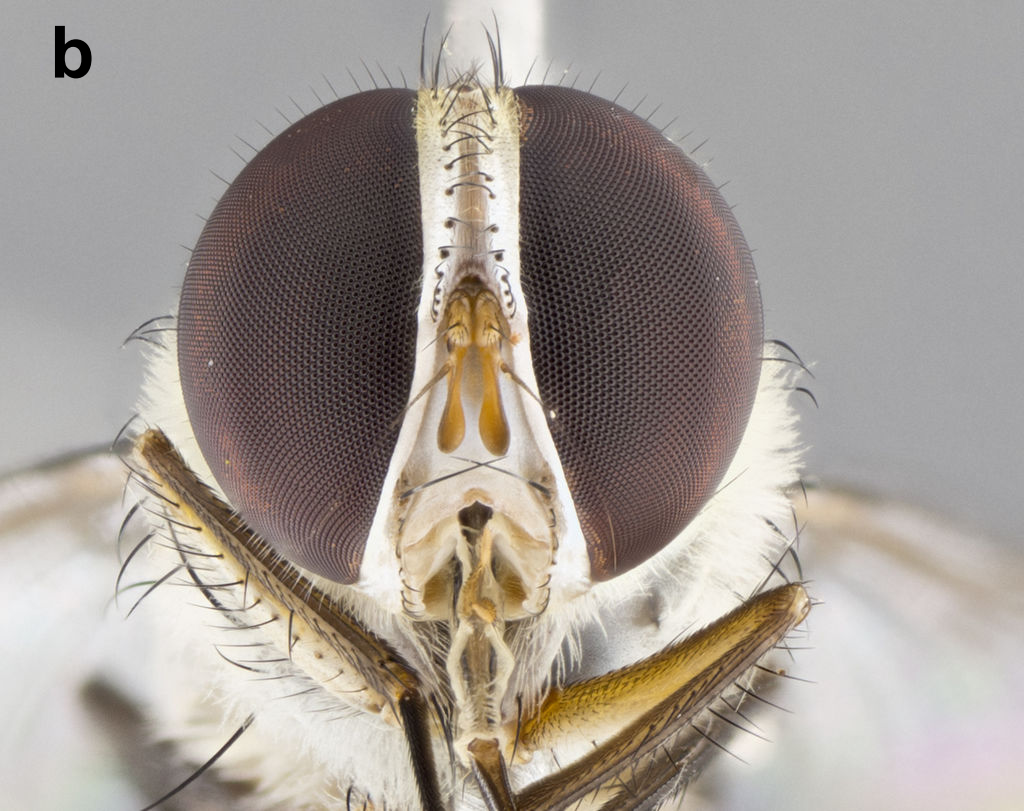
frontal view.

**Figure 22c.**
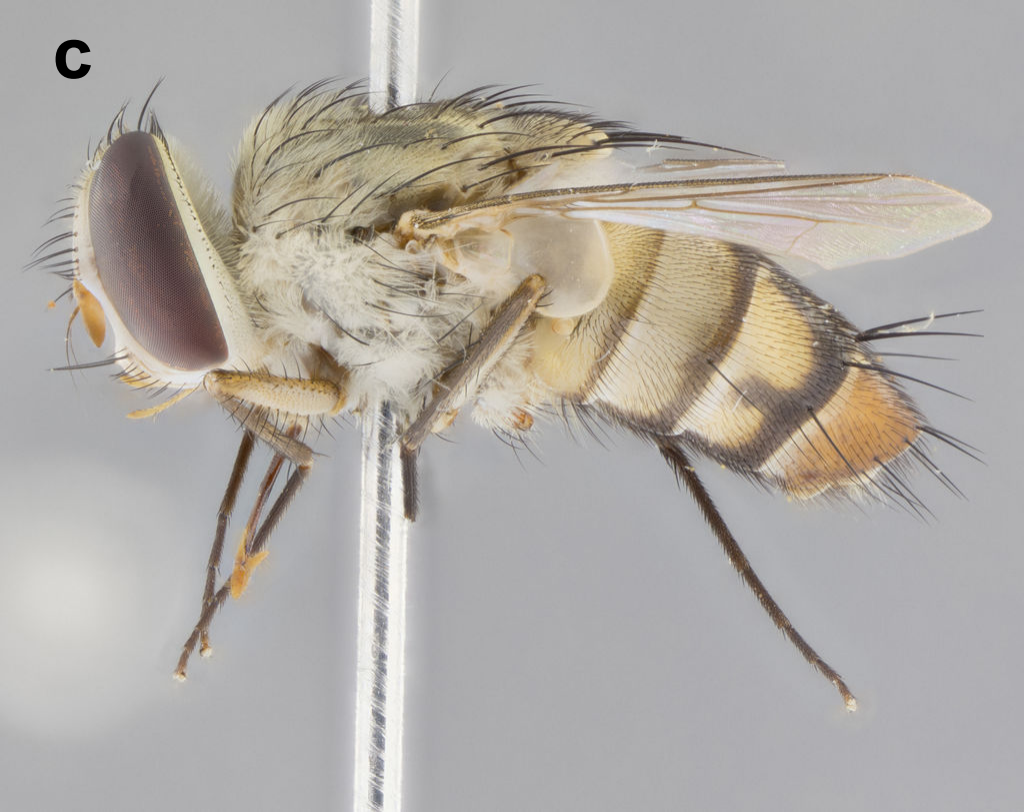
lateral view.

**Figure 22d.**
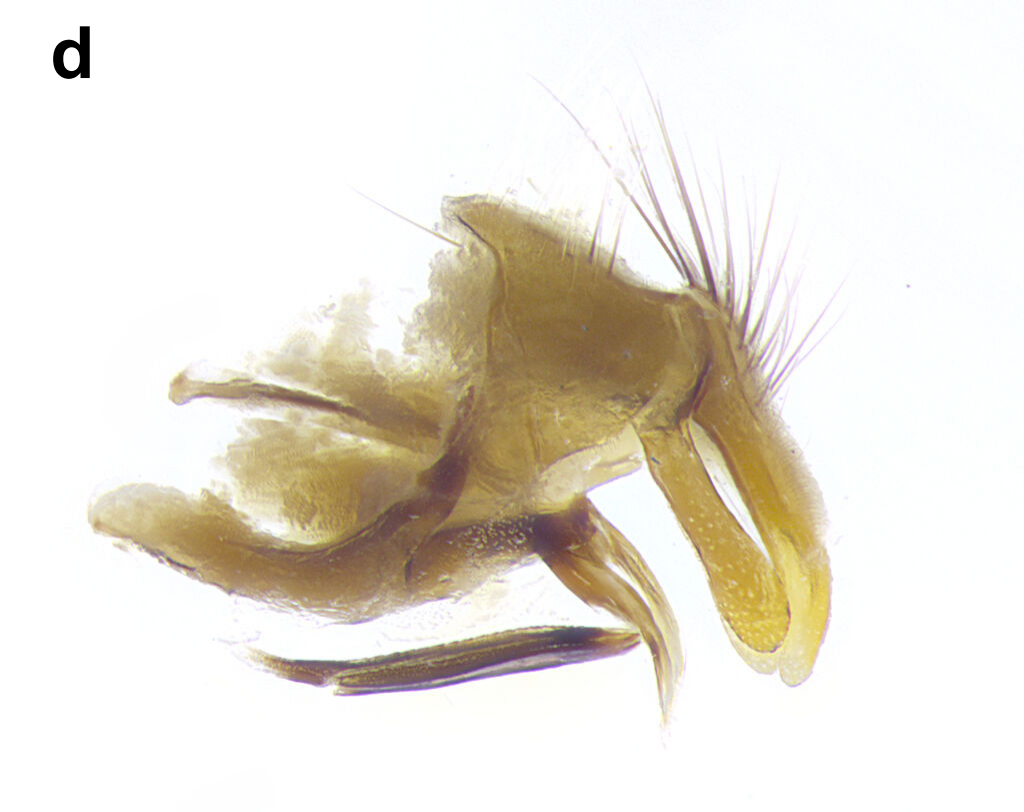
caudal view.

**Figure 22e.**
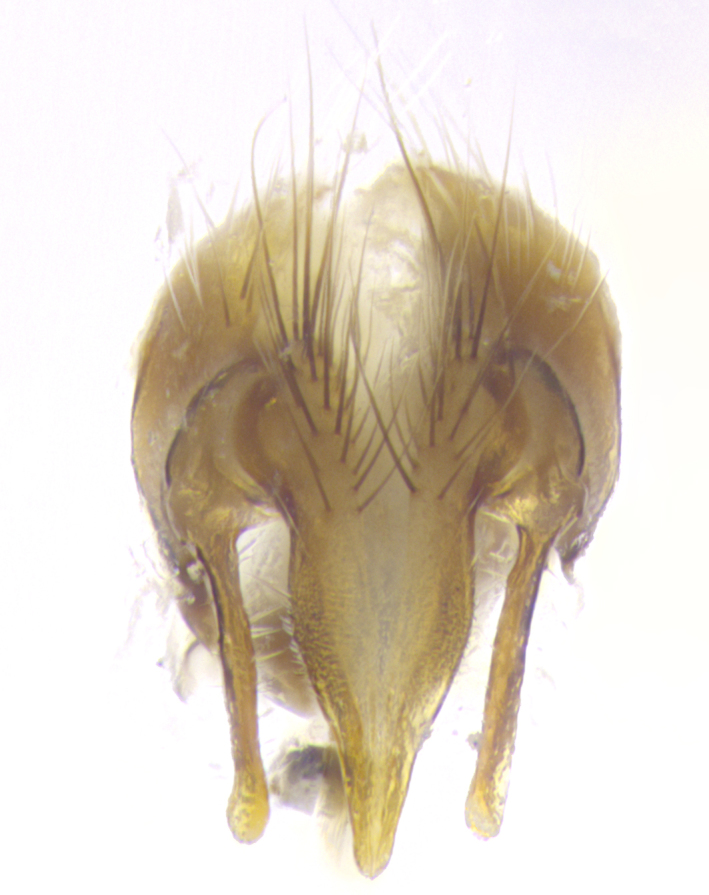
lateral view.

**Figure 22f.**
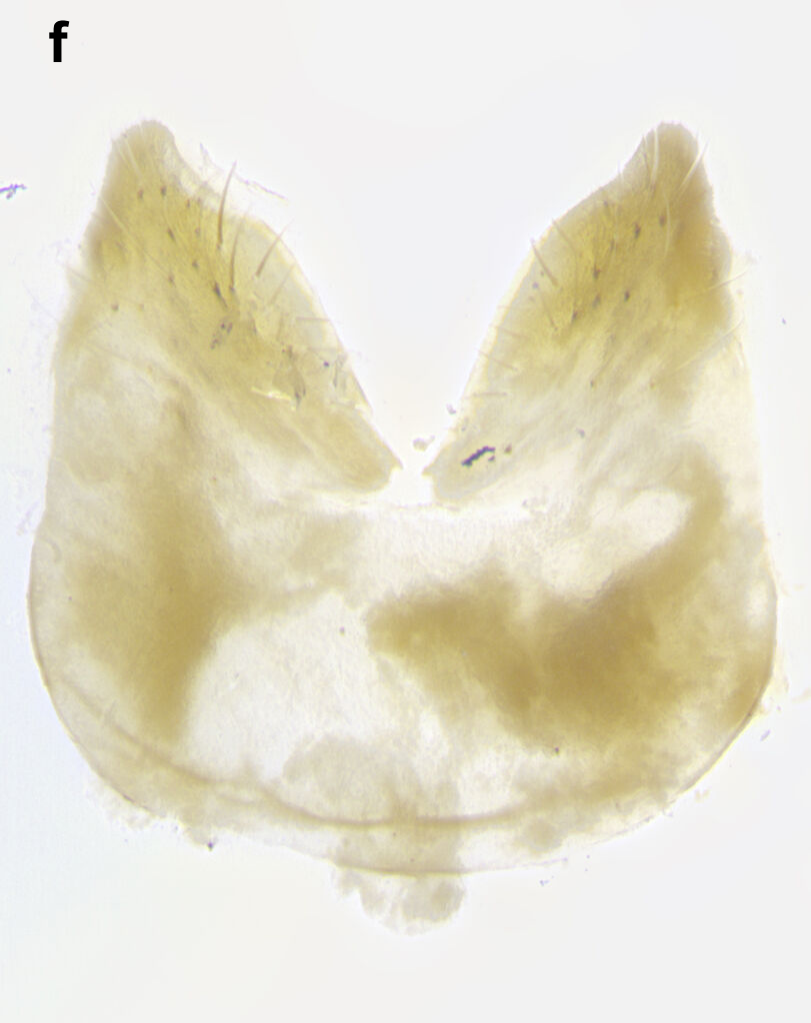
sternite 5, ventral view.

**Figure 23a.**
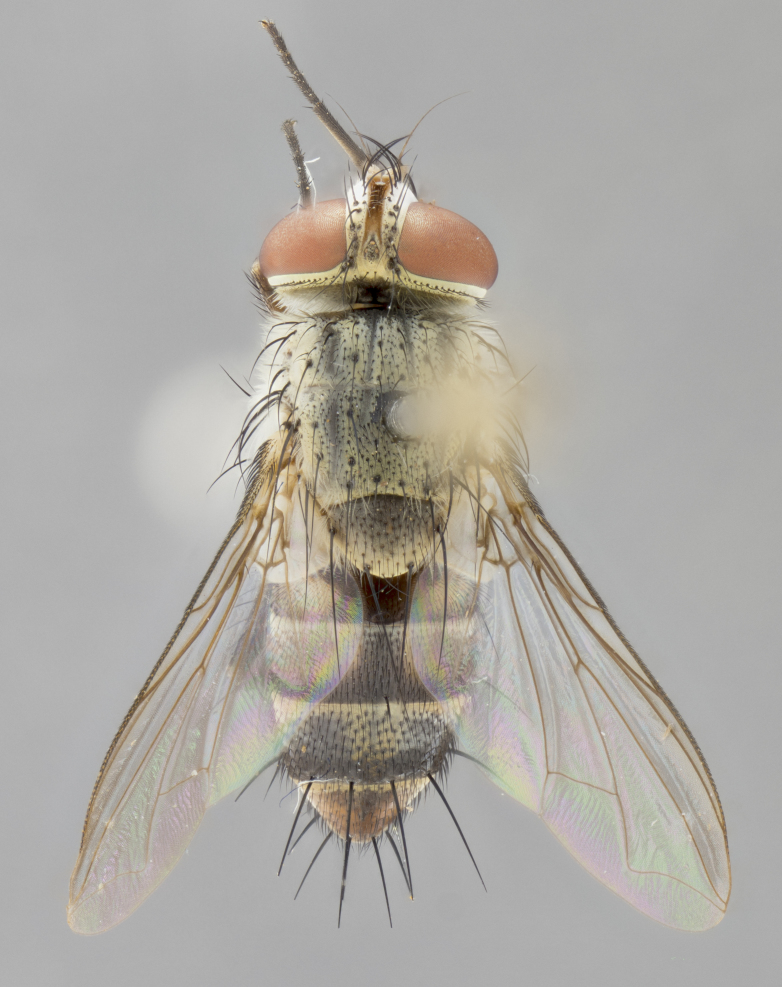
dorsal view.

**Figure 23b.**
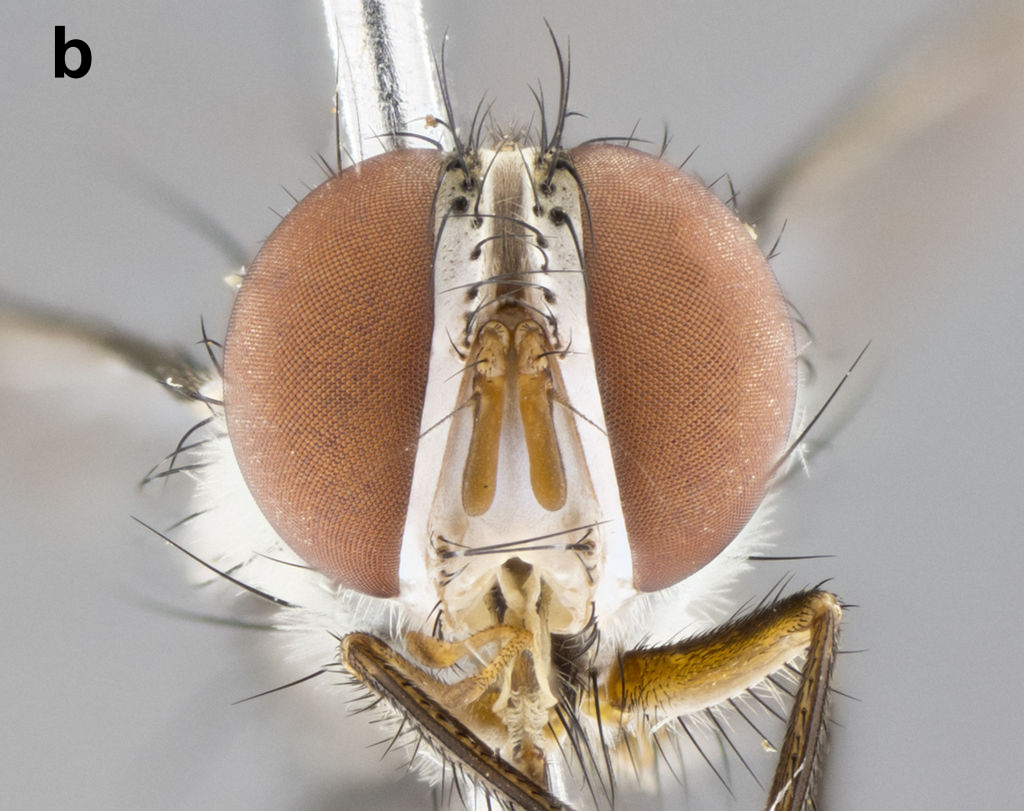
frontal view.

**Figure 23c.**
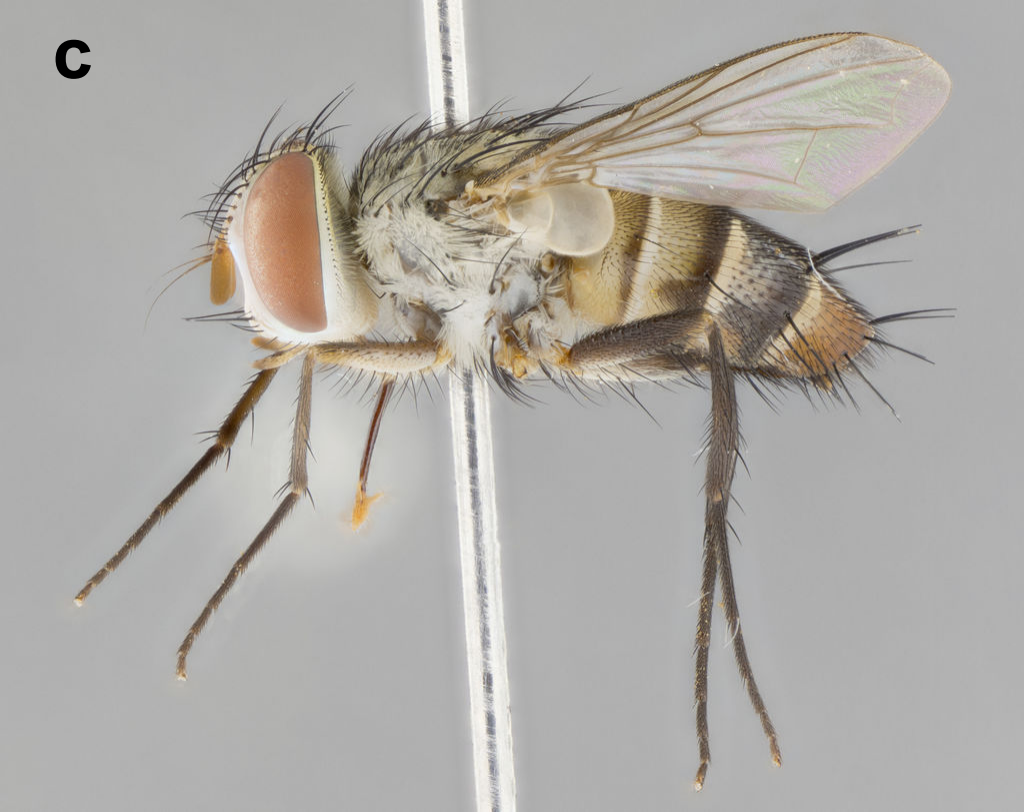
lateral view.

**Figure 24a.**
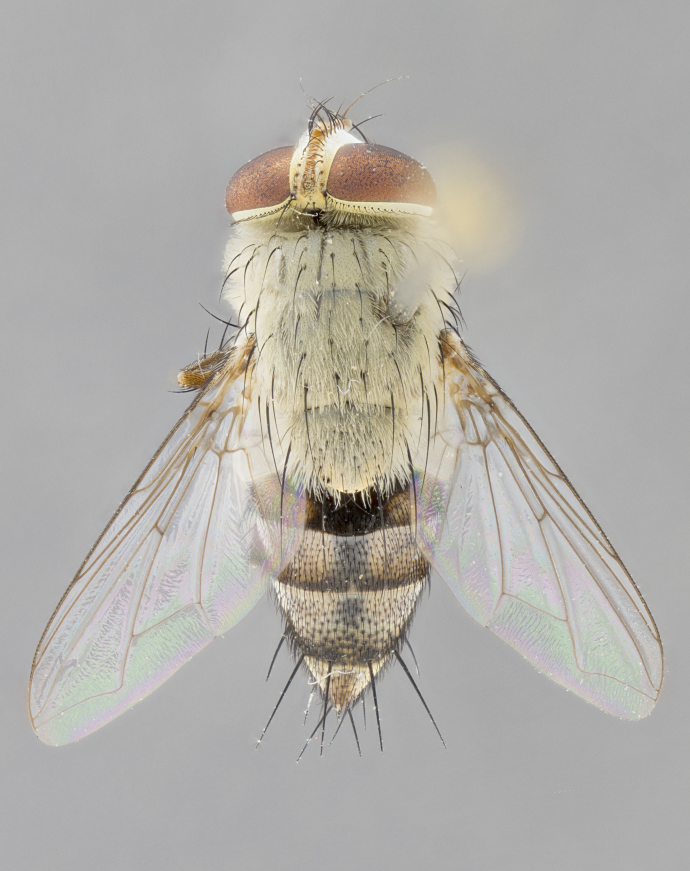
dorsal view.

**Figure 24b.**
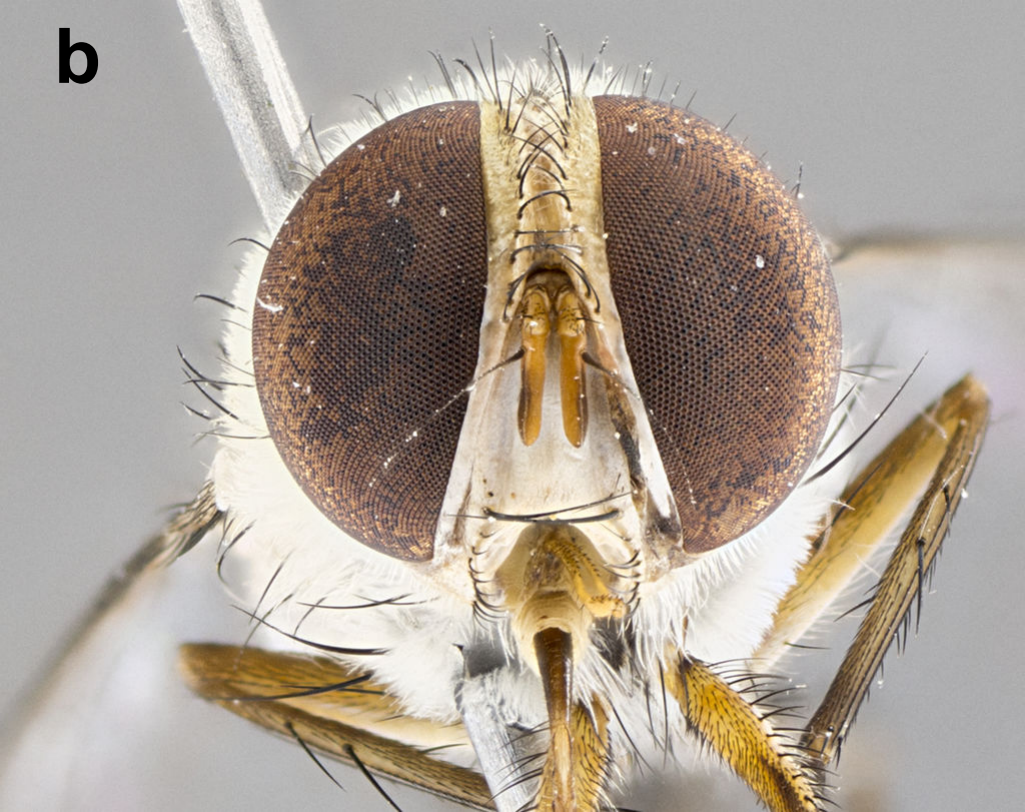
frontal view.

**Figure 24c.**
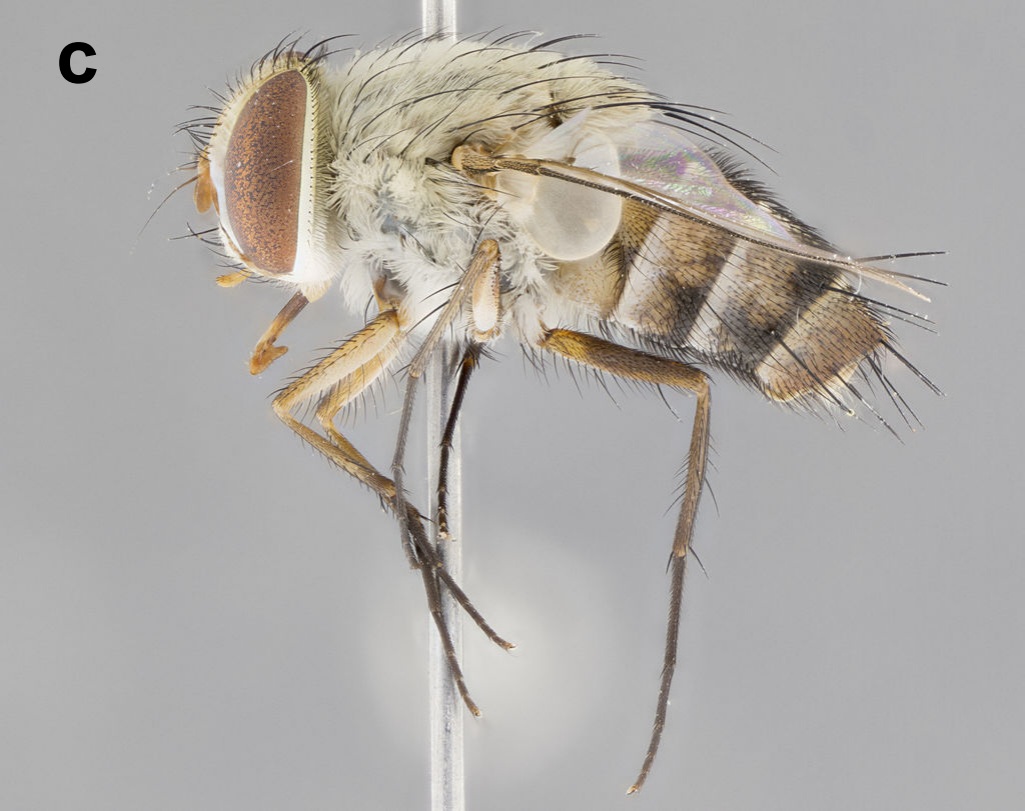
lateral view.

**Figure 24d.**
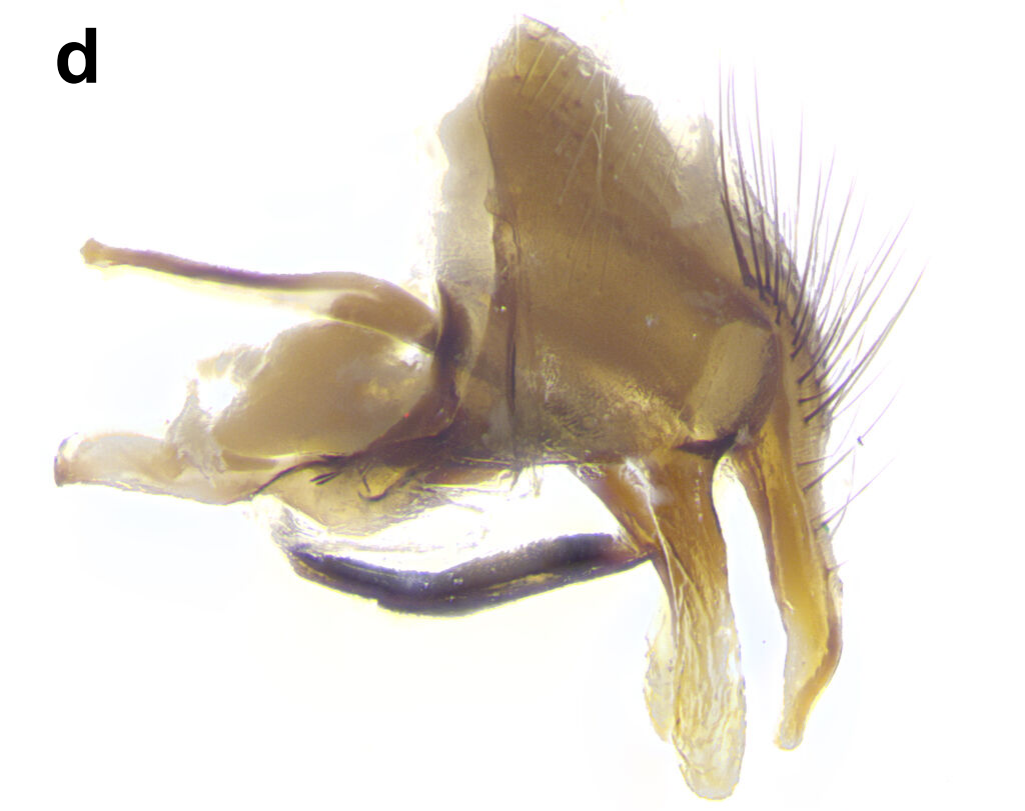
lateral view.

**Figure 24e.**
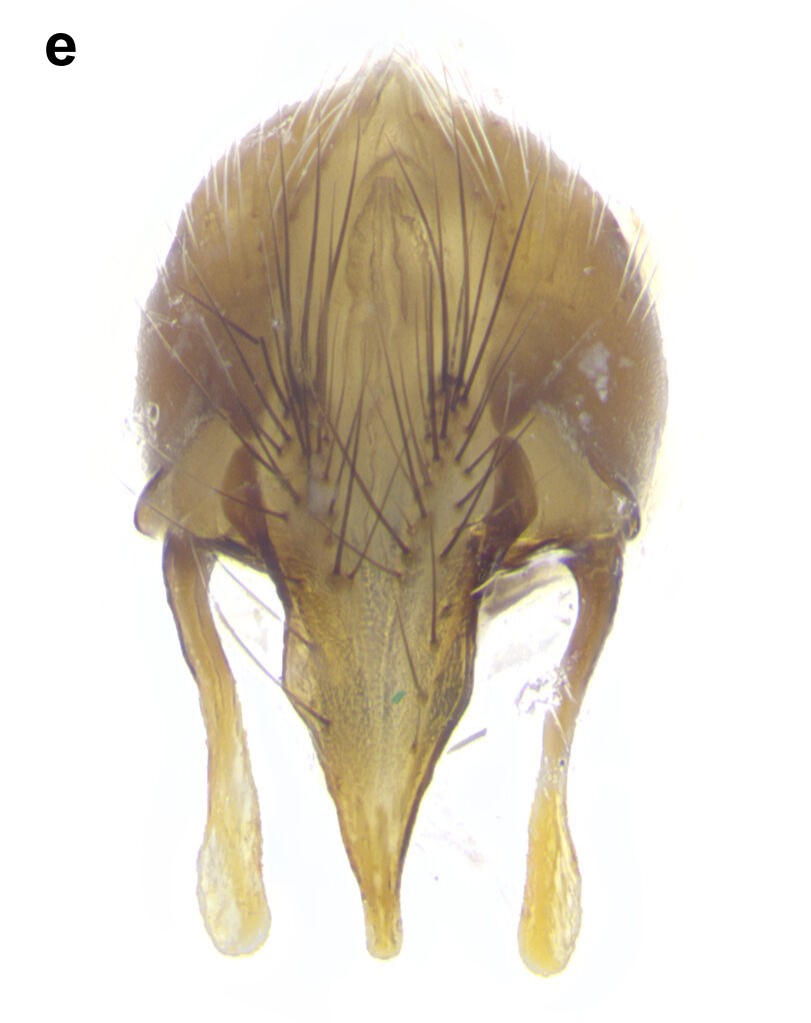
caudal view.

**Figure 24f.**
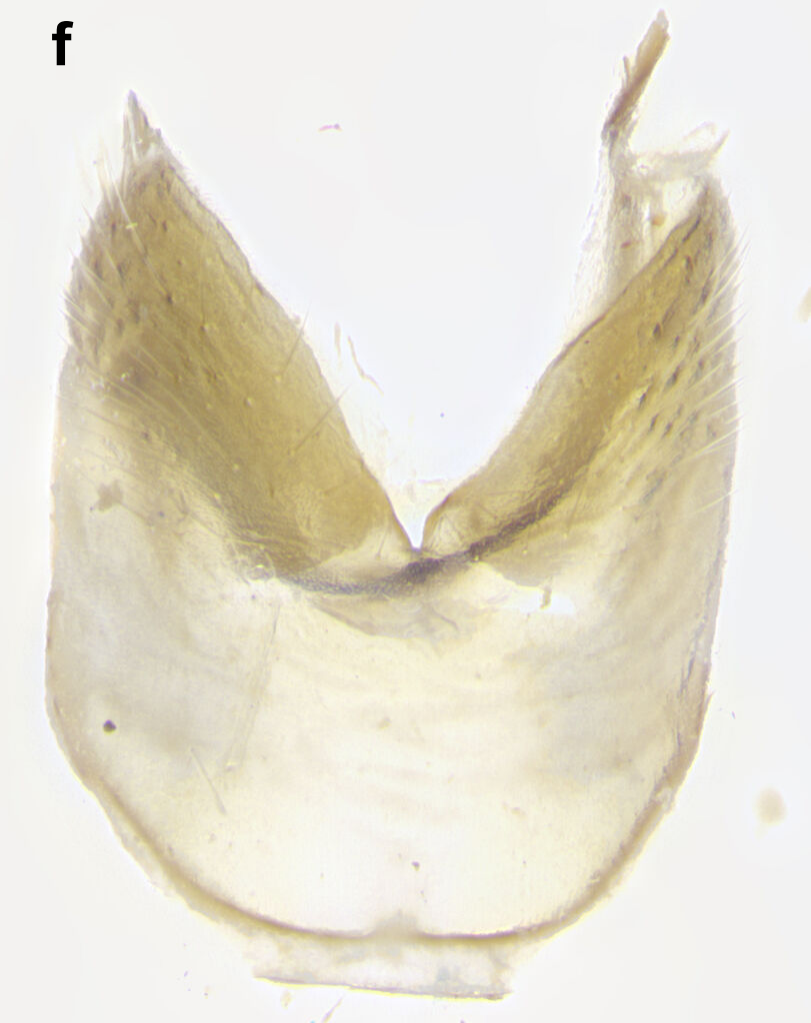
sternite 5, ventral view.

**Figure 26a.**
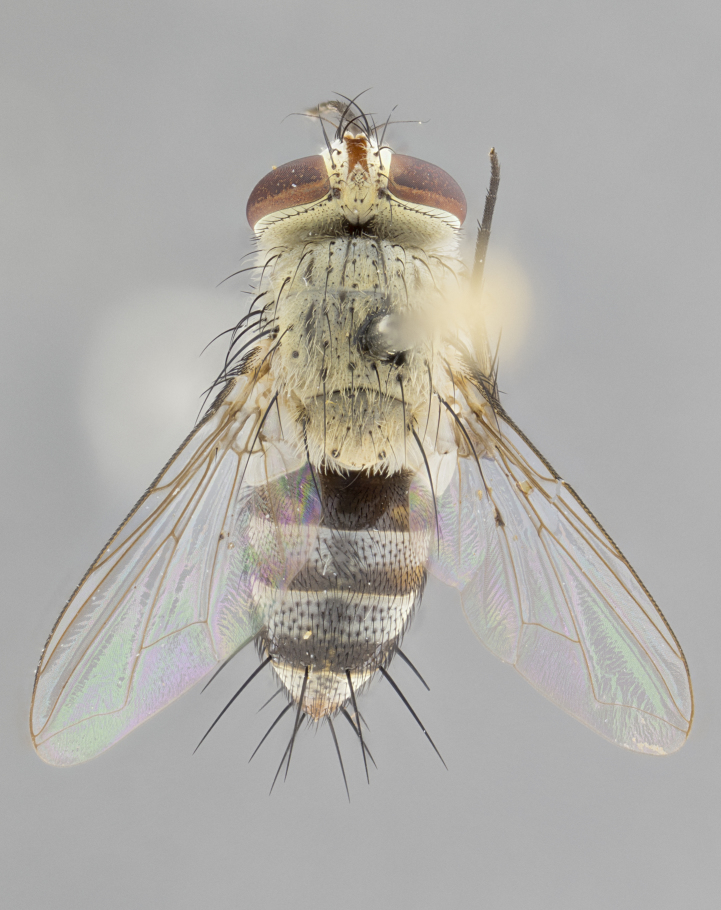
dorsal view.

**Figure 26b.**
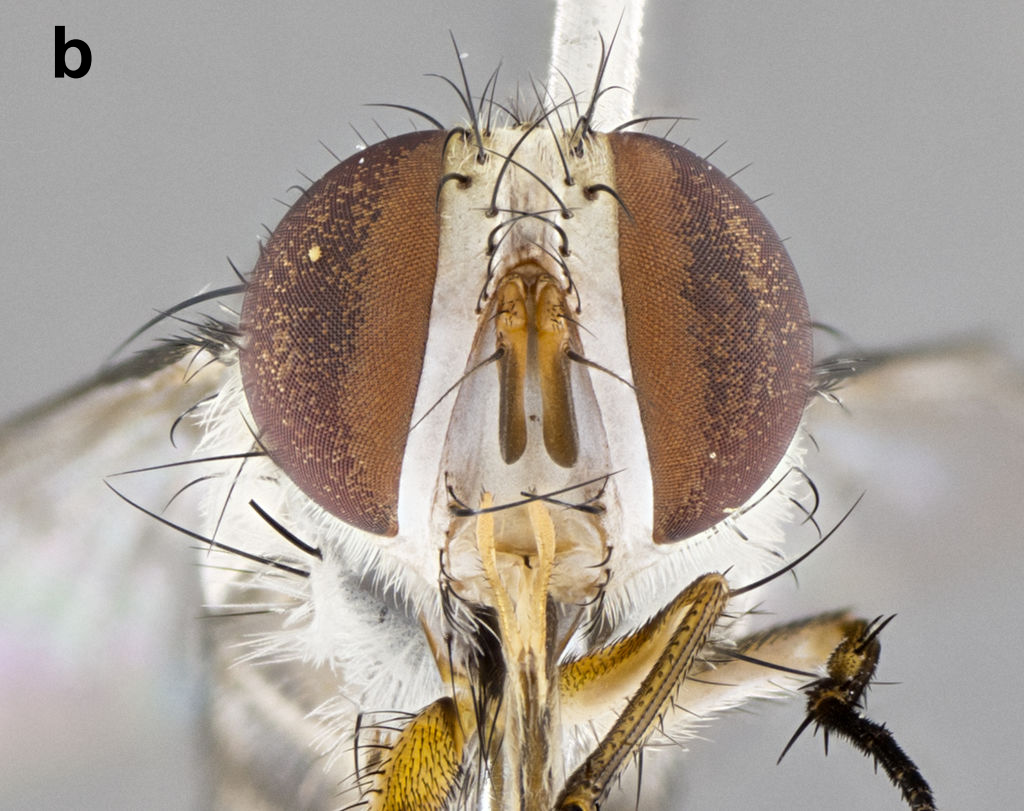
frontal view.

**Figure 26c.**
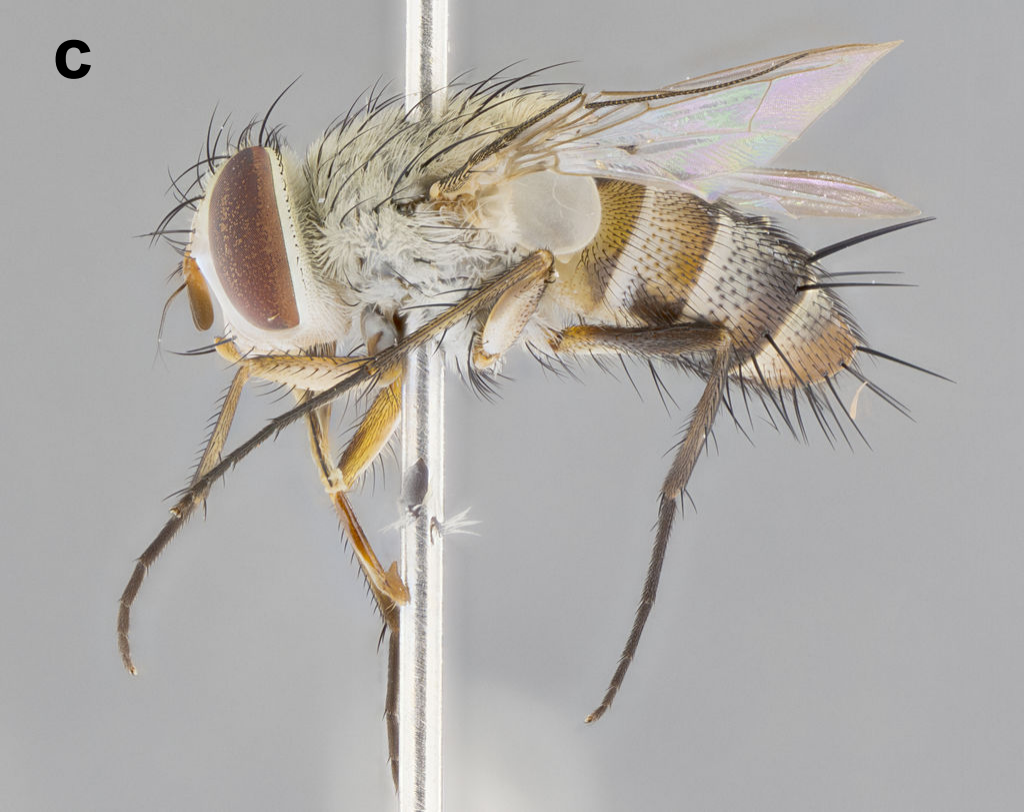
lateral view.

**Figure 27a.**
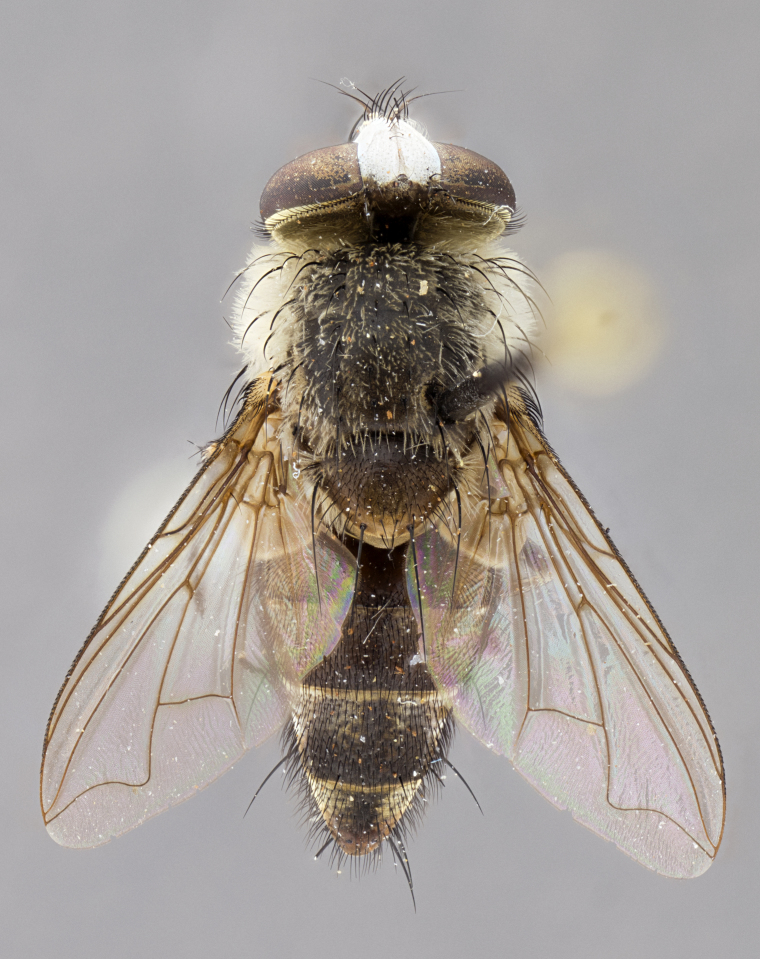
dorsal view.

**Figure 27b.**
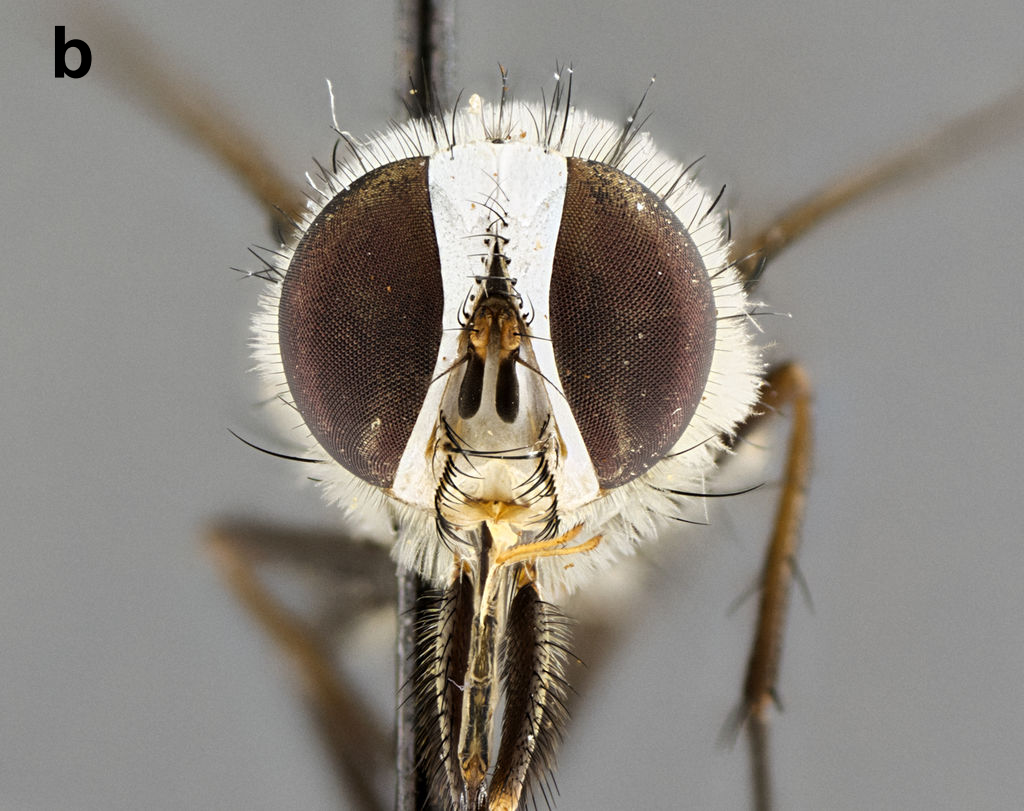
frontal view.

**Figure 27c.**
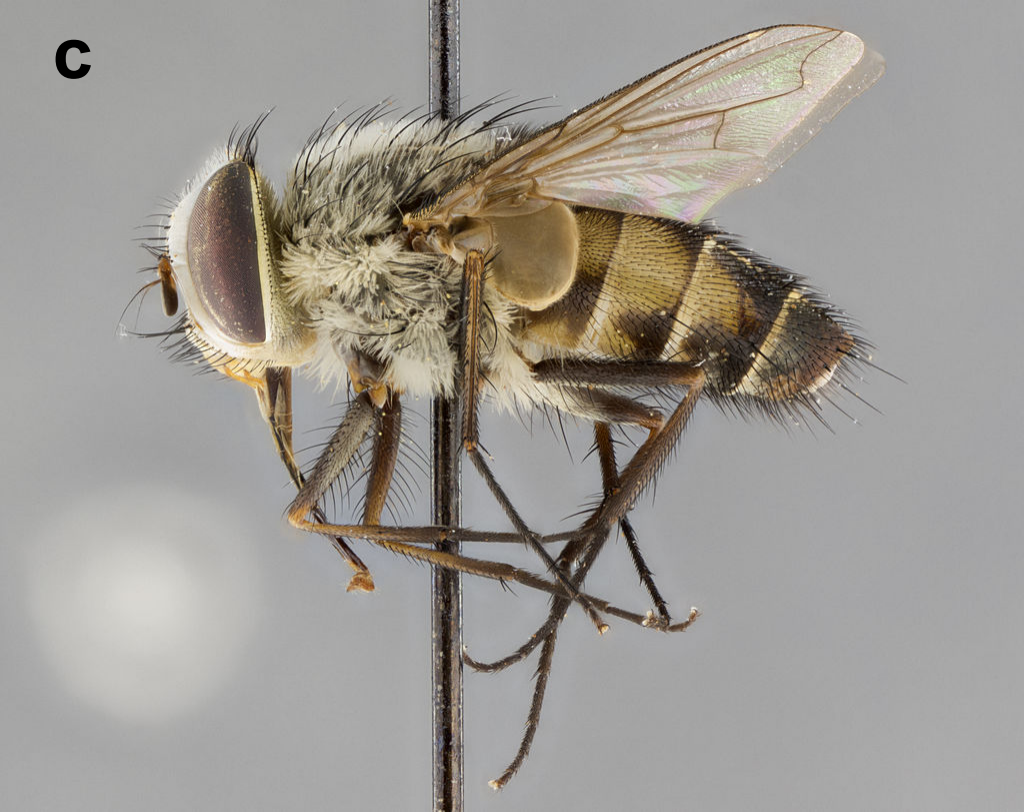
lateral view.

**Figure 27d.**
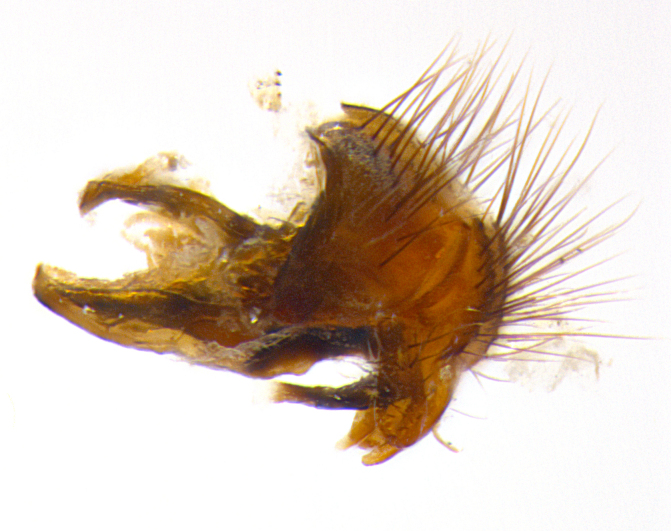
lateral view.

**Figure 27e.**
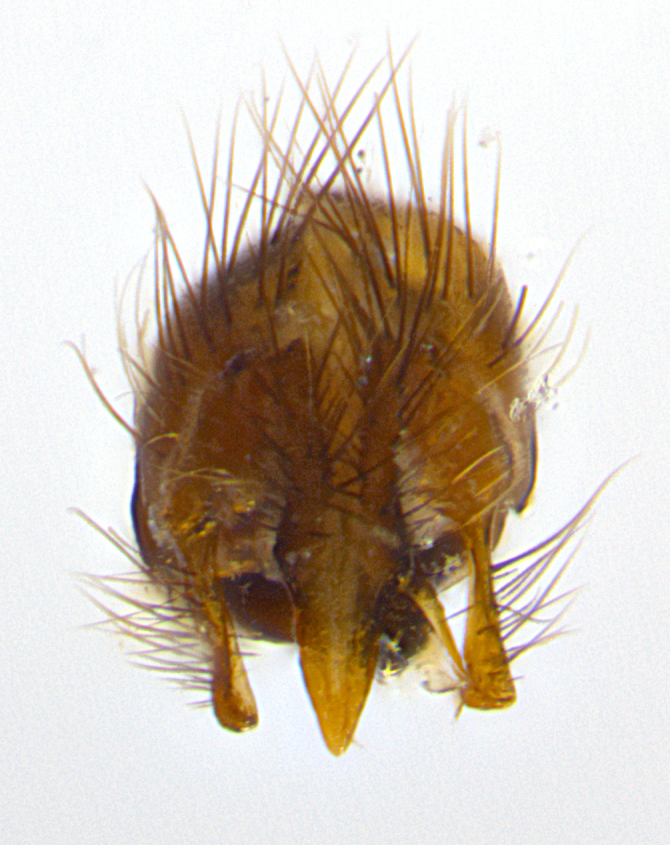
caudal view.

**Figure 27f.**
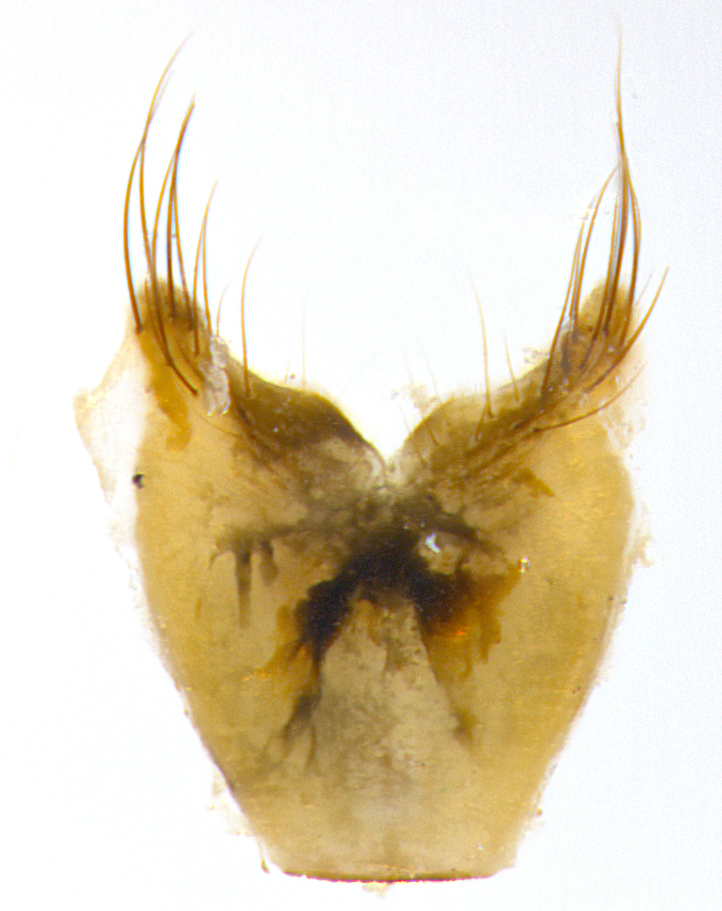
sternite 5, ventral view.

**Figure 28a.**
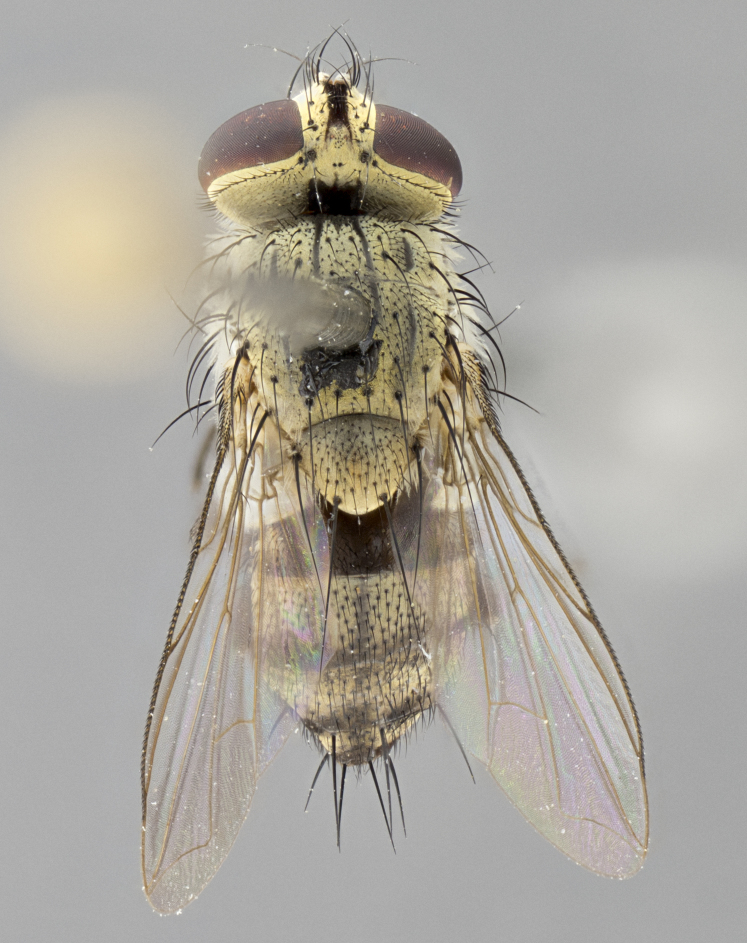
dorsal view.

**Figure 28b.**
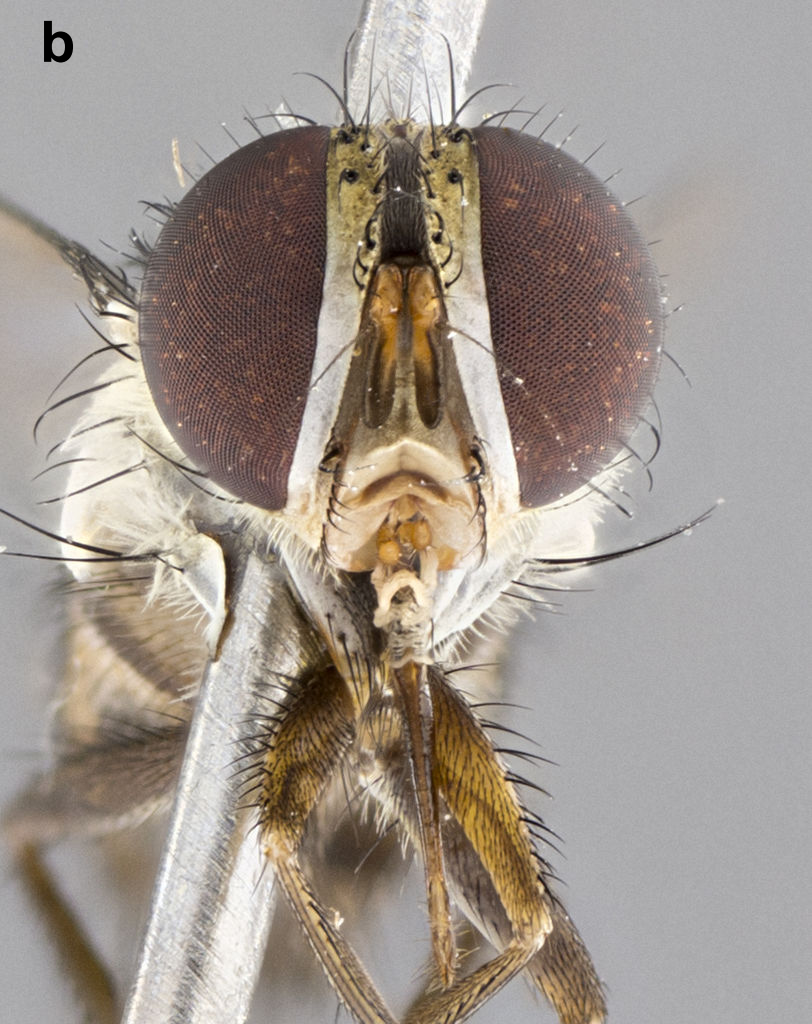
frontal view.

**Figure 28c.**
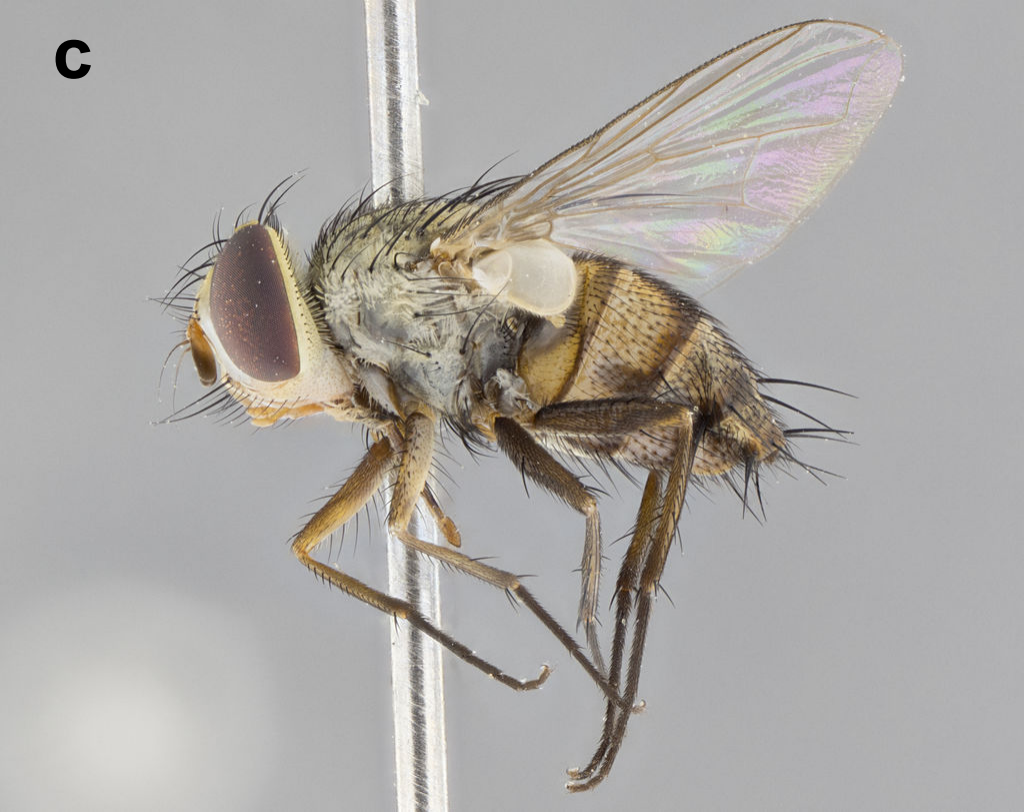
lateral view.

**Figure 29a.**
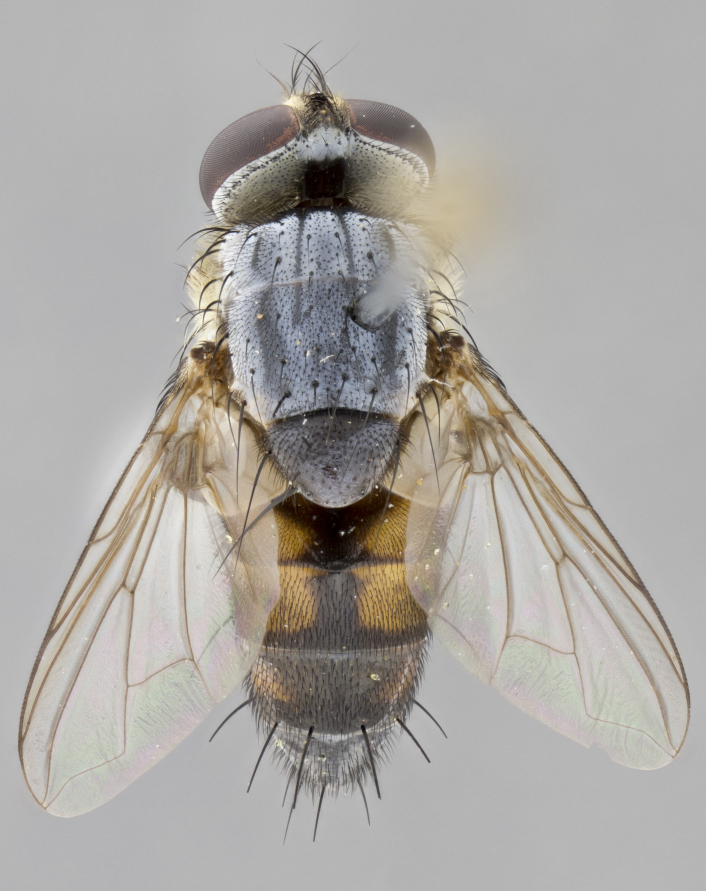
dorsal view.

**Figure 29b.**
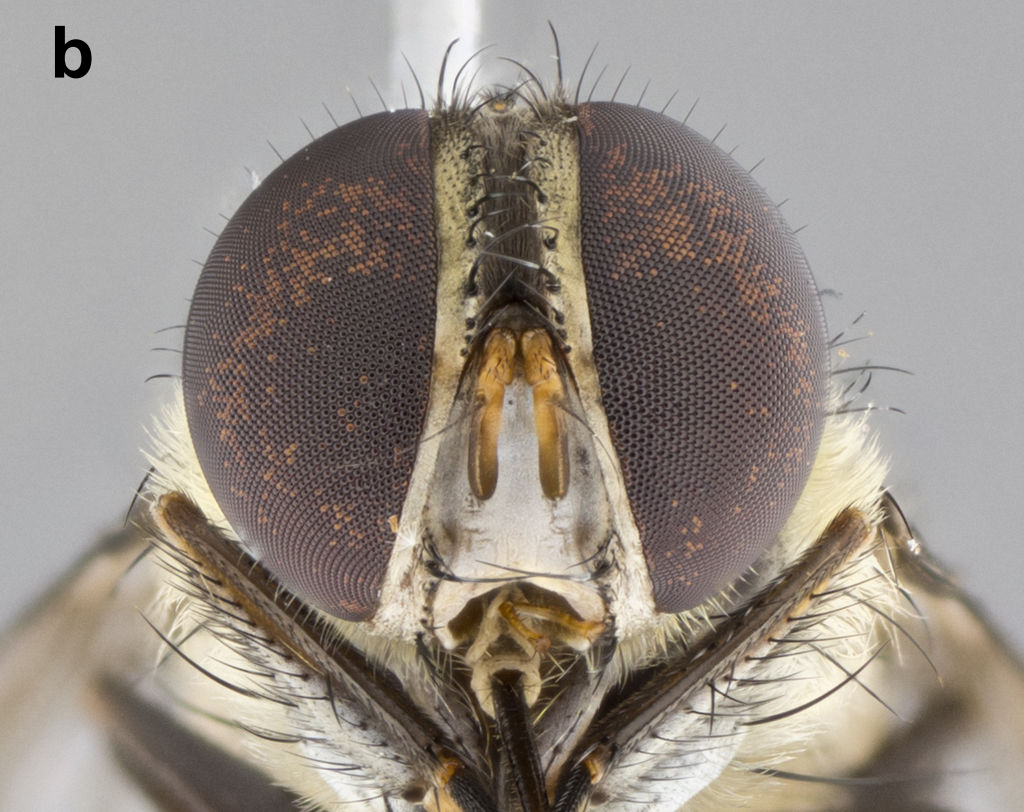
frontal view.

**Figure 29c.**
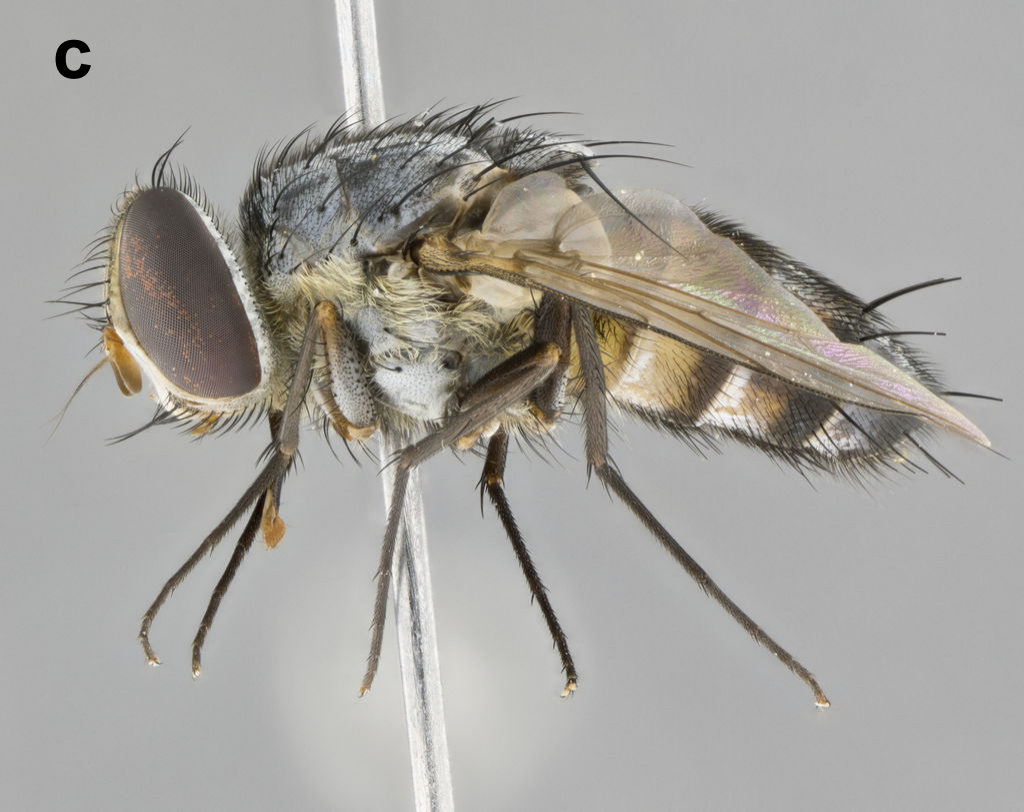
lateral view.

**Figure 30a.**
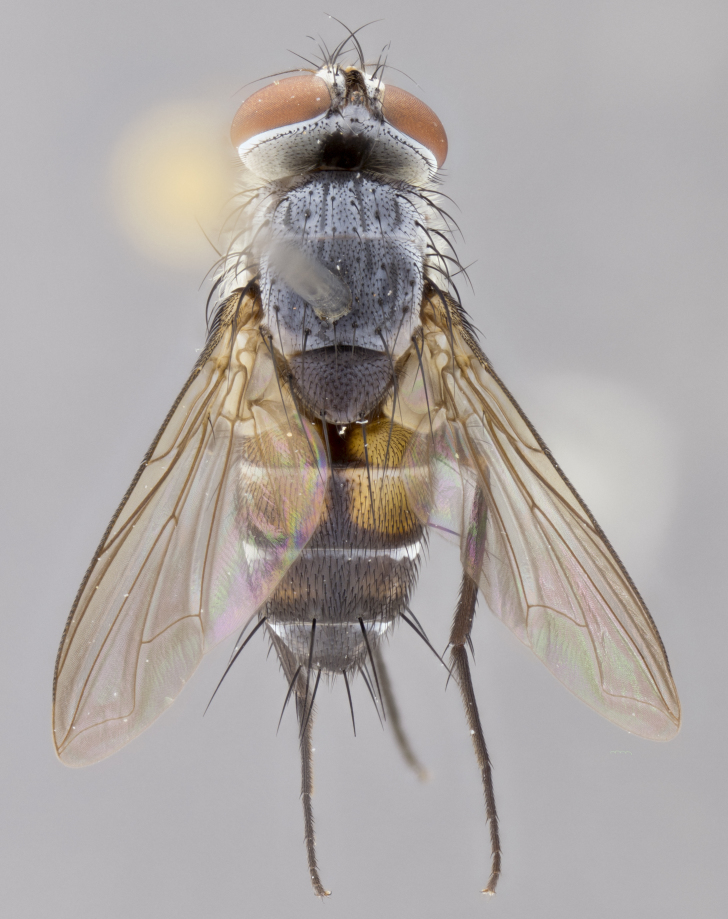
dorsal view.

**Figure 30b.**
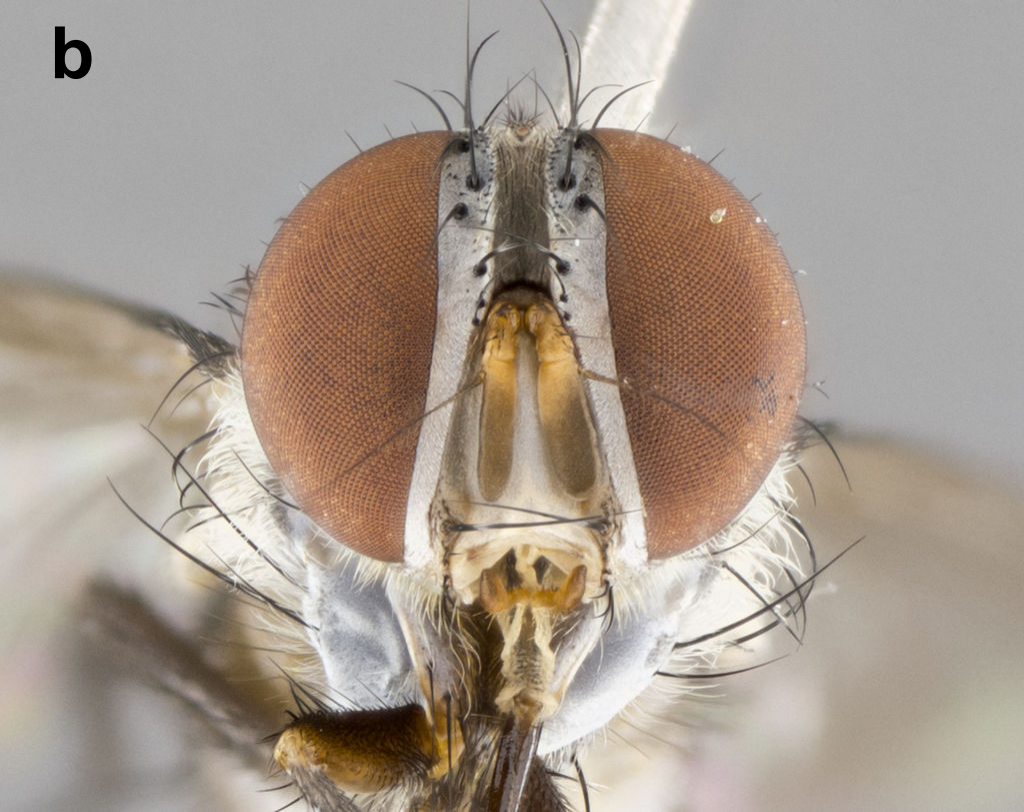
frontal view.

**Figure 30c.**
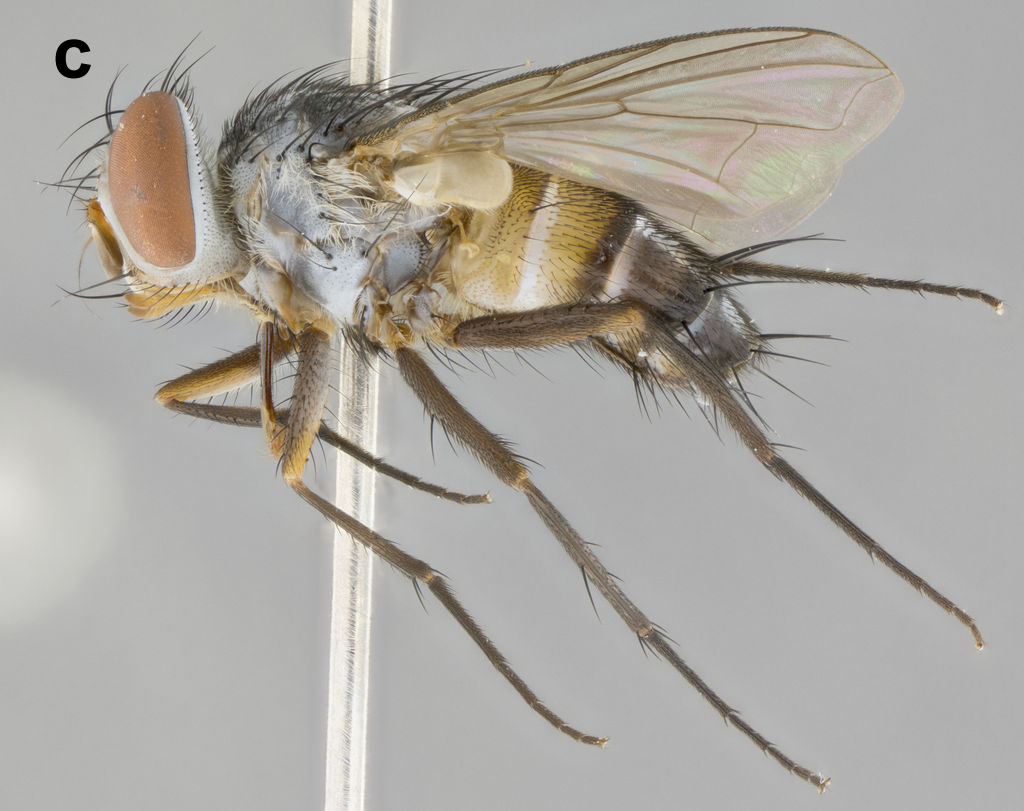
lateral view.

**Figure 31a.**
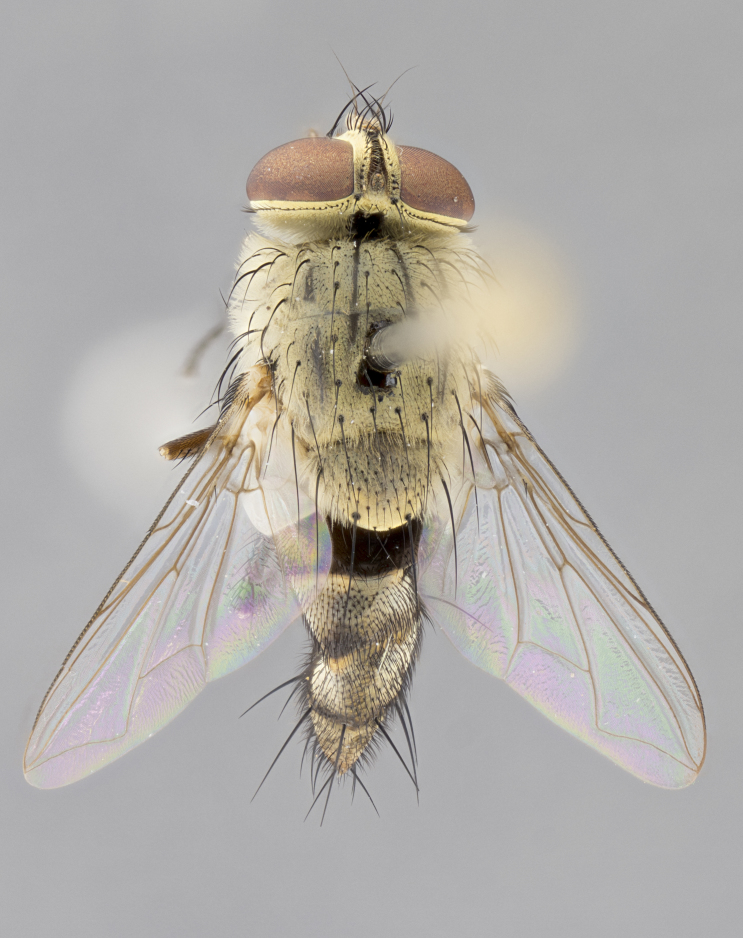
dorsal view.

**Figure 31b.**
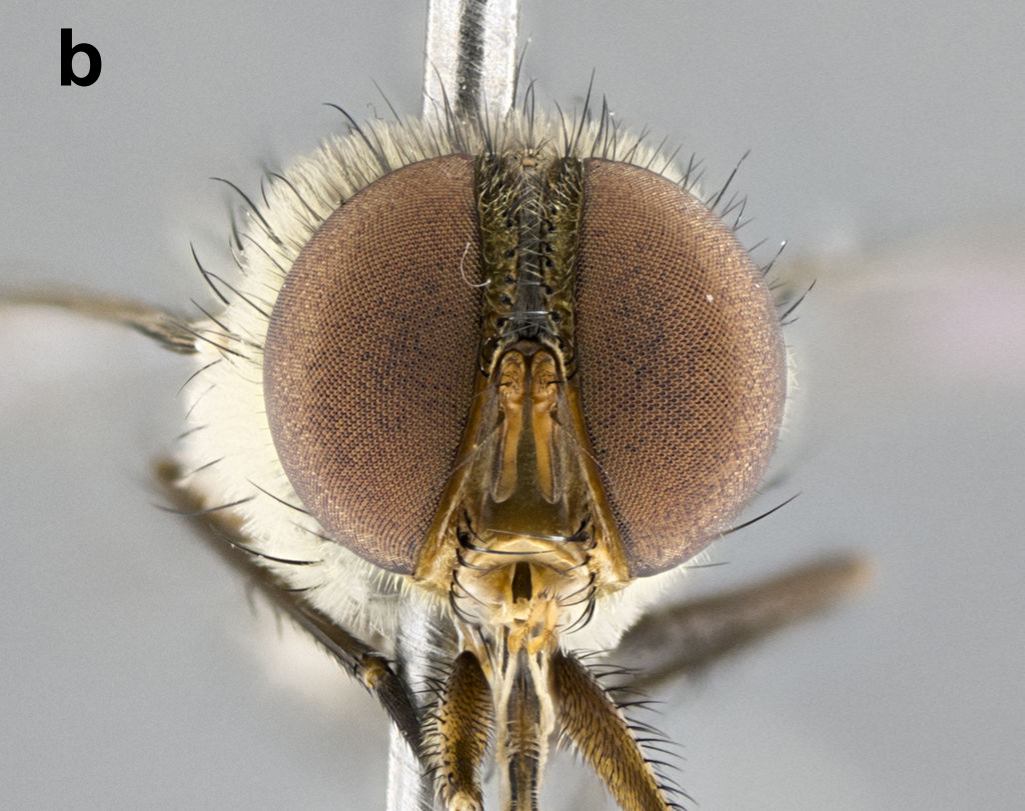
frontal view.

**Figure 31c.**
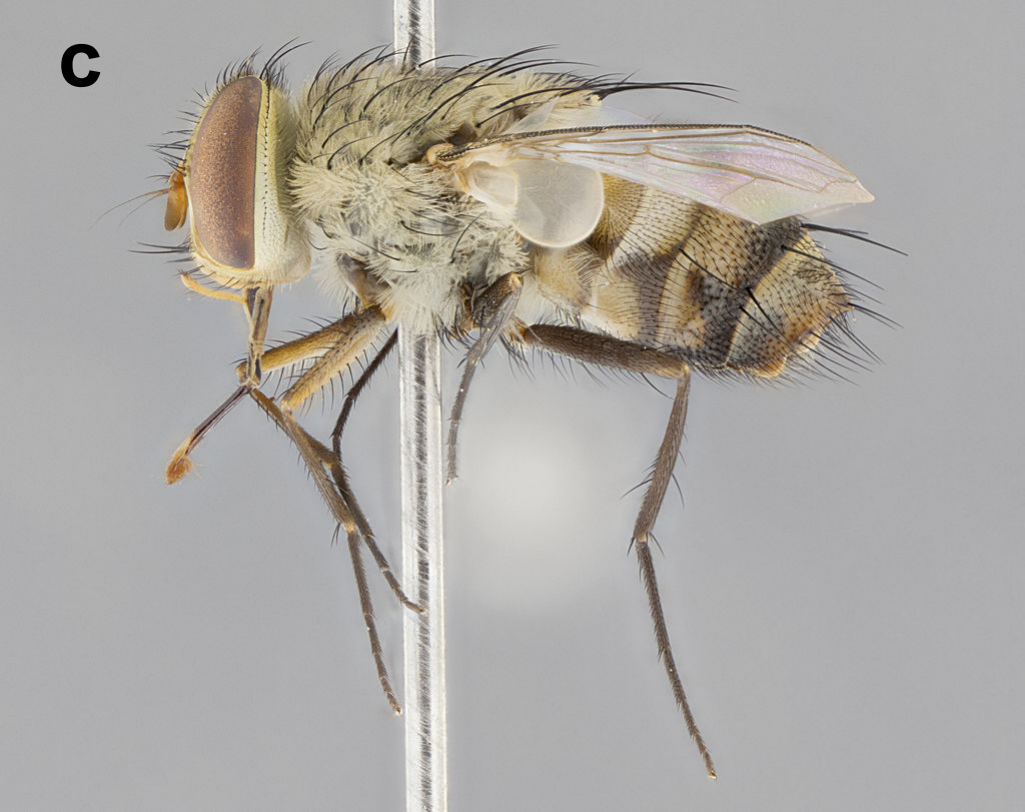
lateral view.

**Figure 31d.**
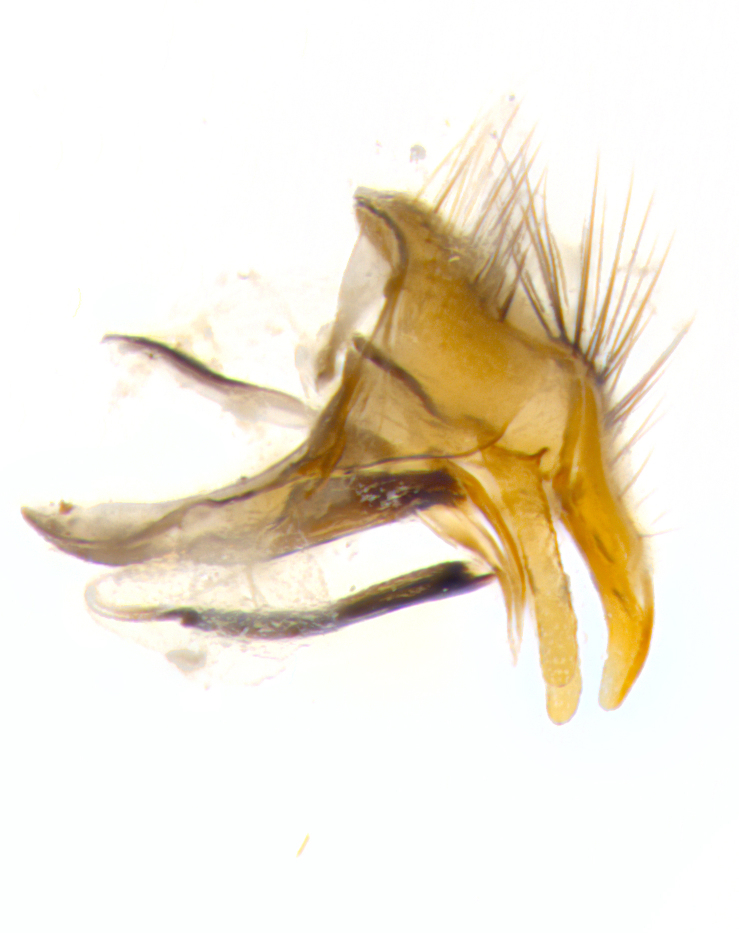
caudal view.

**Figure 31e.**
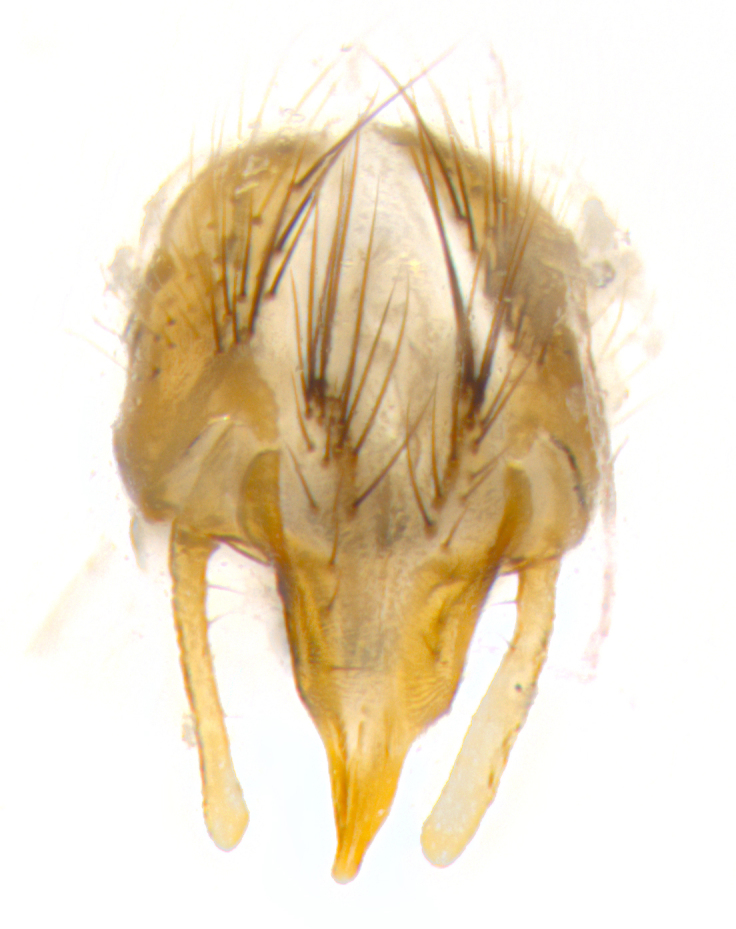
lateral view.

**Figure 31f.**
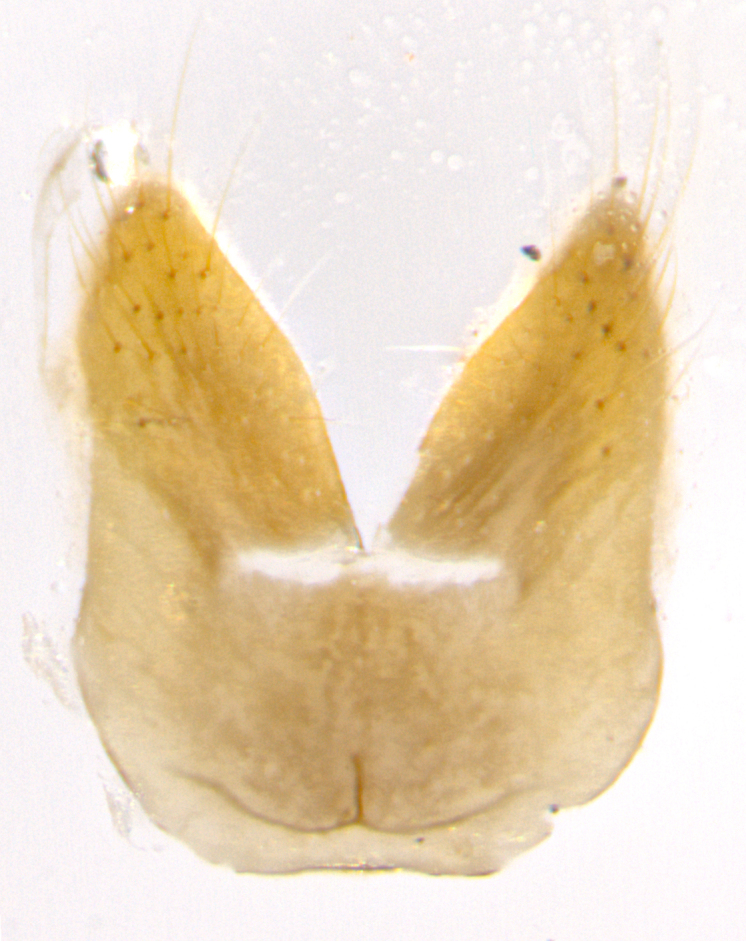
sternite 5, ventral view.

**Figure 32a.**
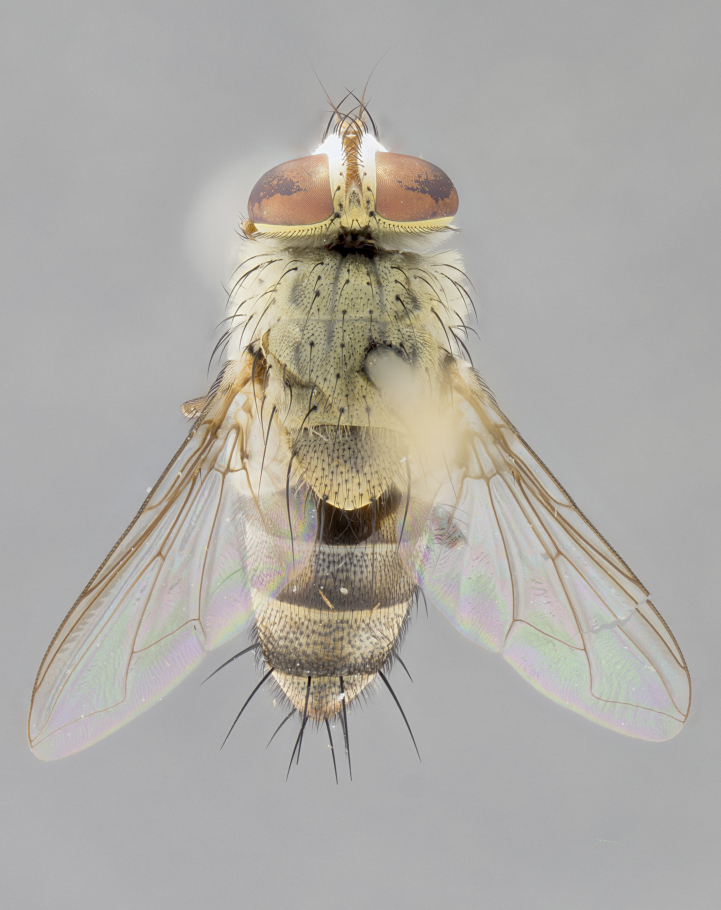
dorsal view.

**Figure 32b.**
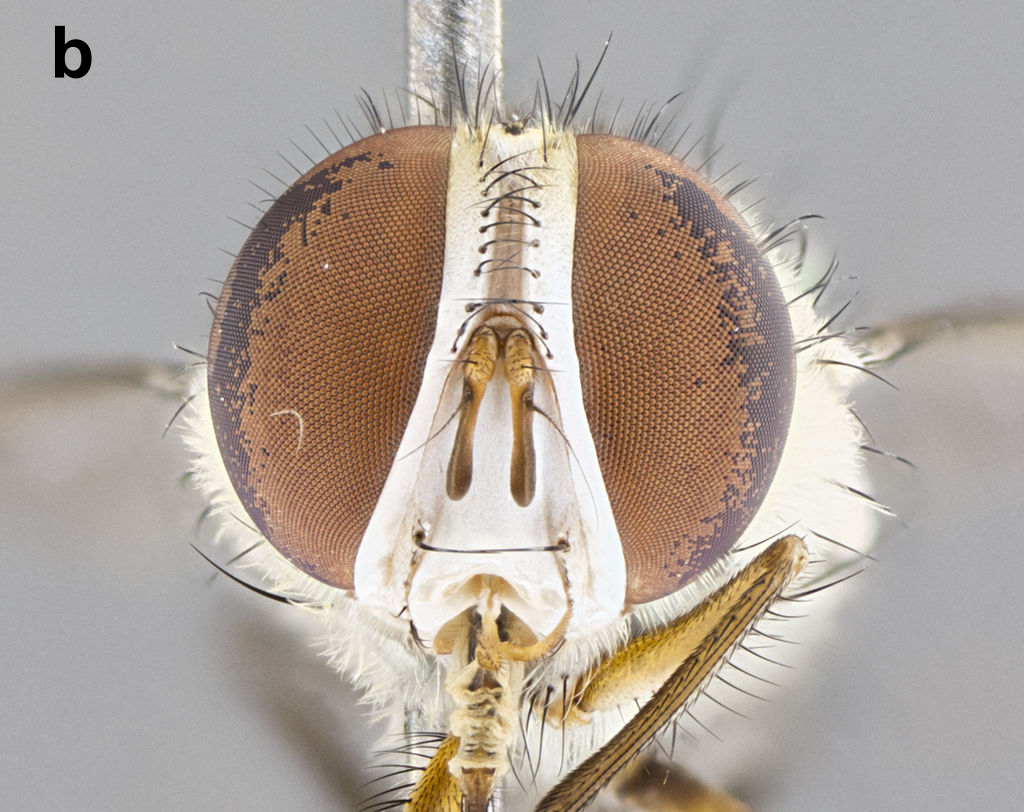
frontal view.

**Figure 32c.**
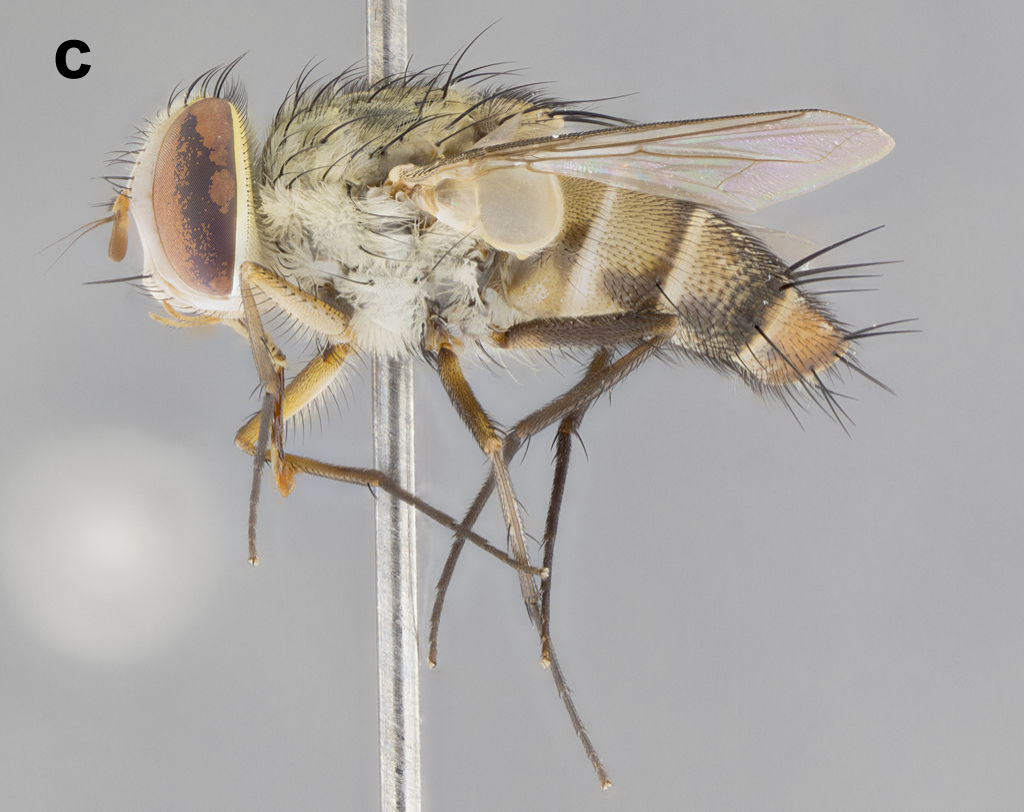
lateral view.

**Figure 32d.**
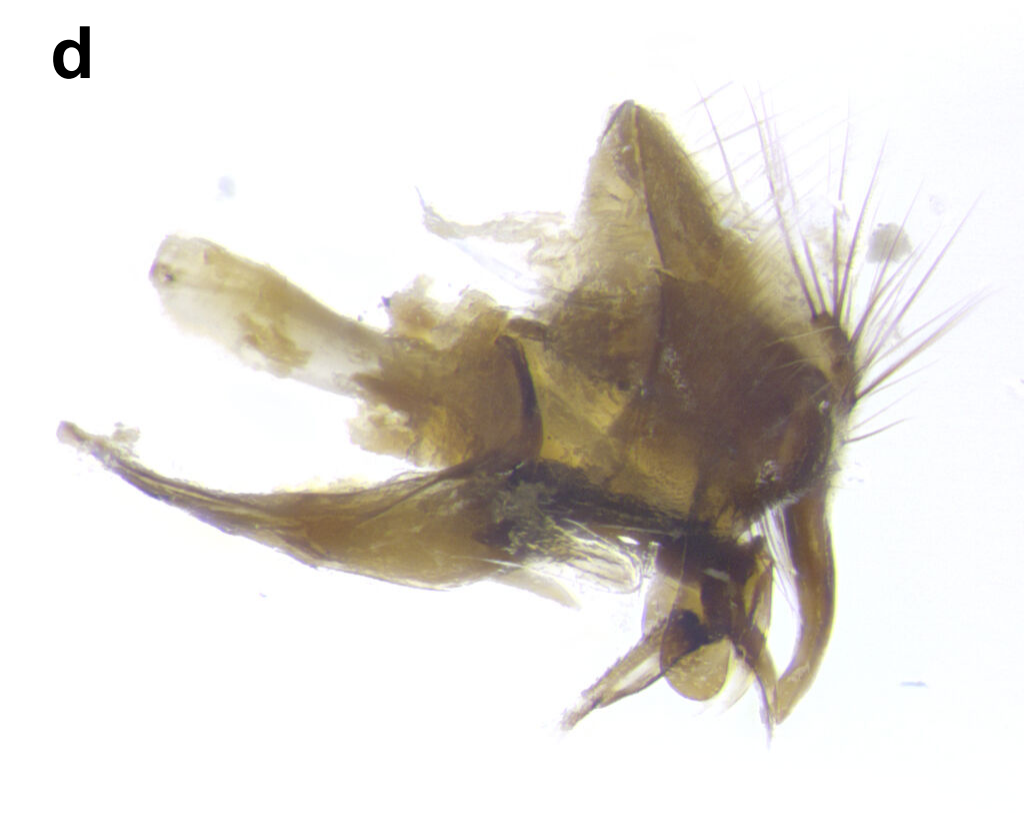
caudal view.

**Figure 32e.**
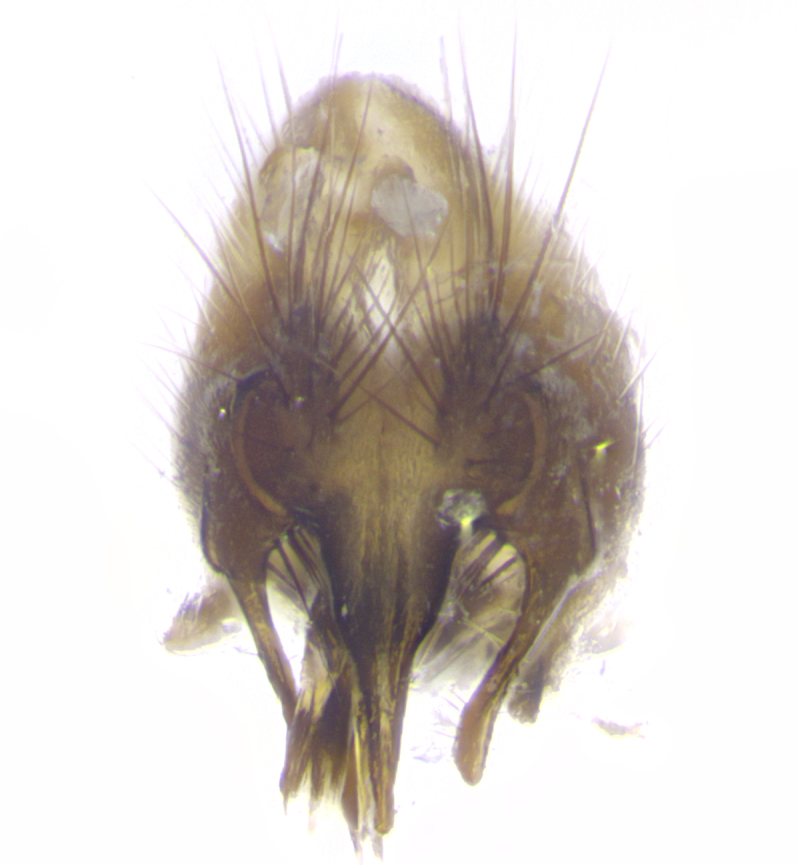
lateral view.

**Figure 32f.**
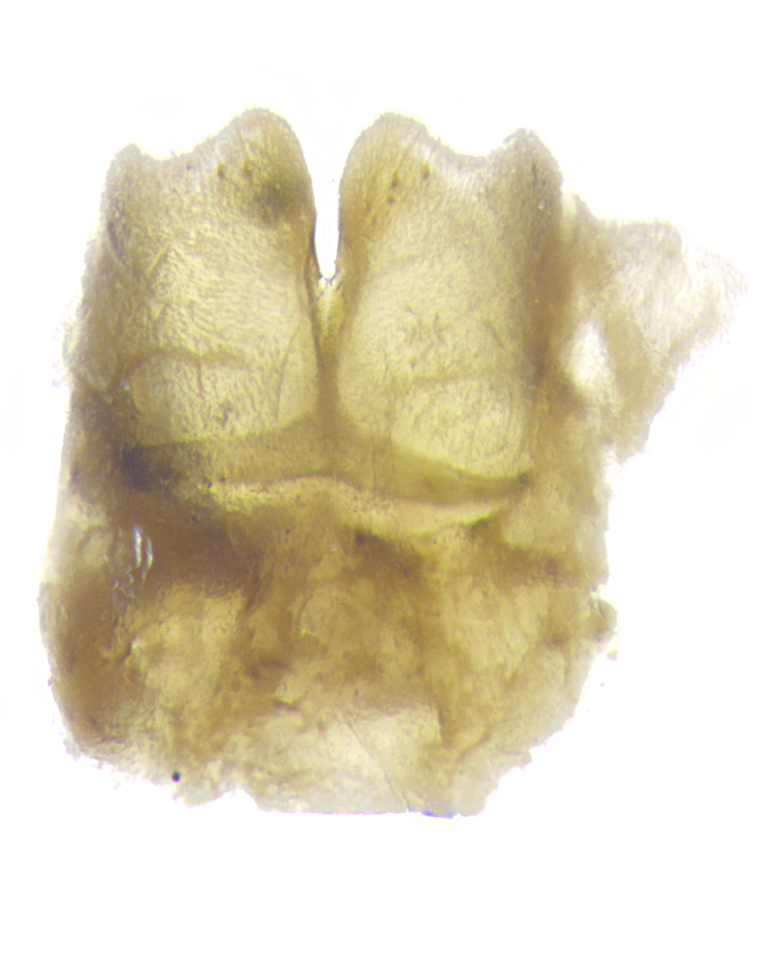
sternite 5, ventral view.

**Figure 33a.**
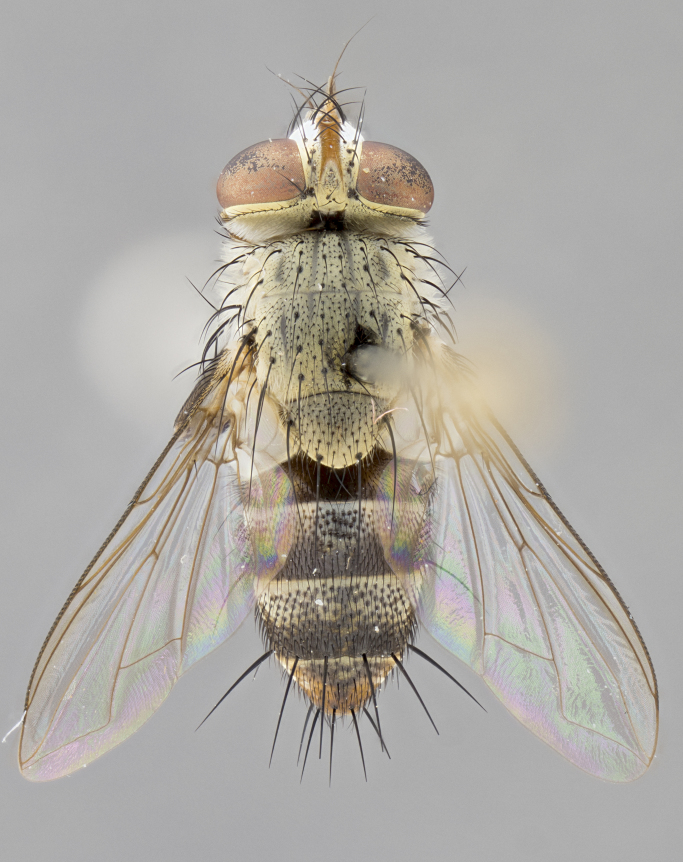
dorsal view.

**Figure 33b.**
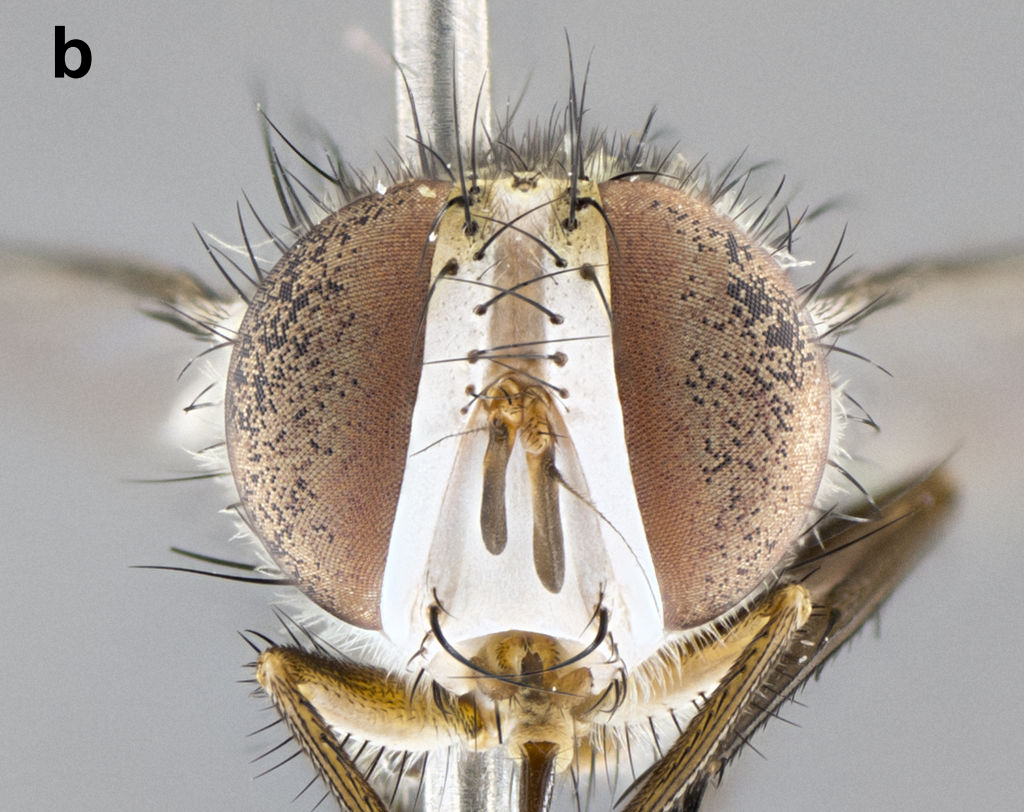
frontal view.

**Figure 33c.**
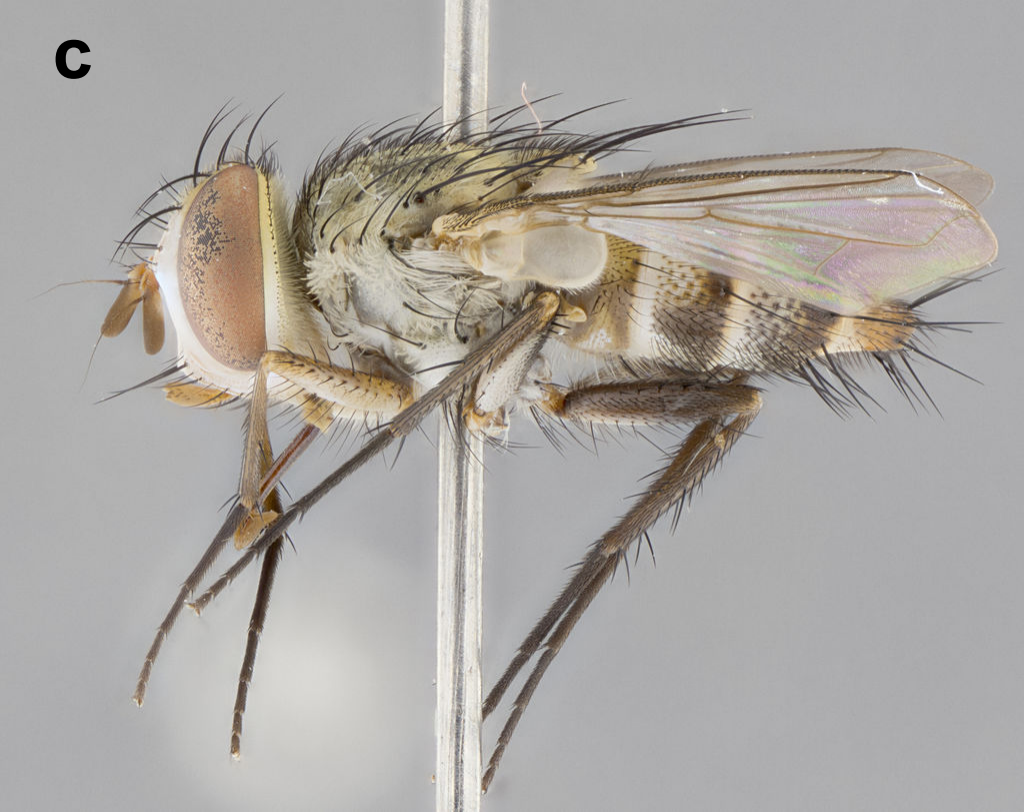
lateral view.

**Figure 34a.**
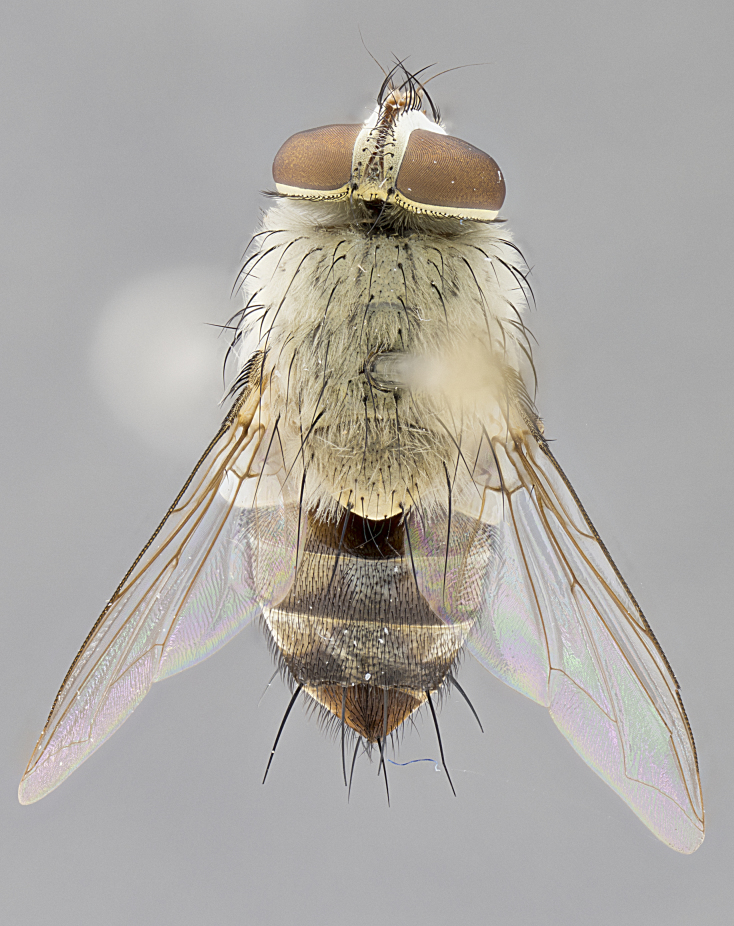
dorsal view.

**Figure 34b.**
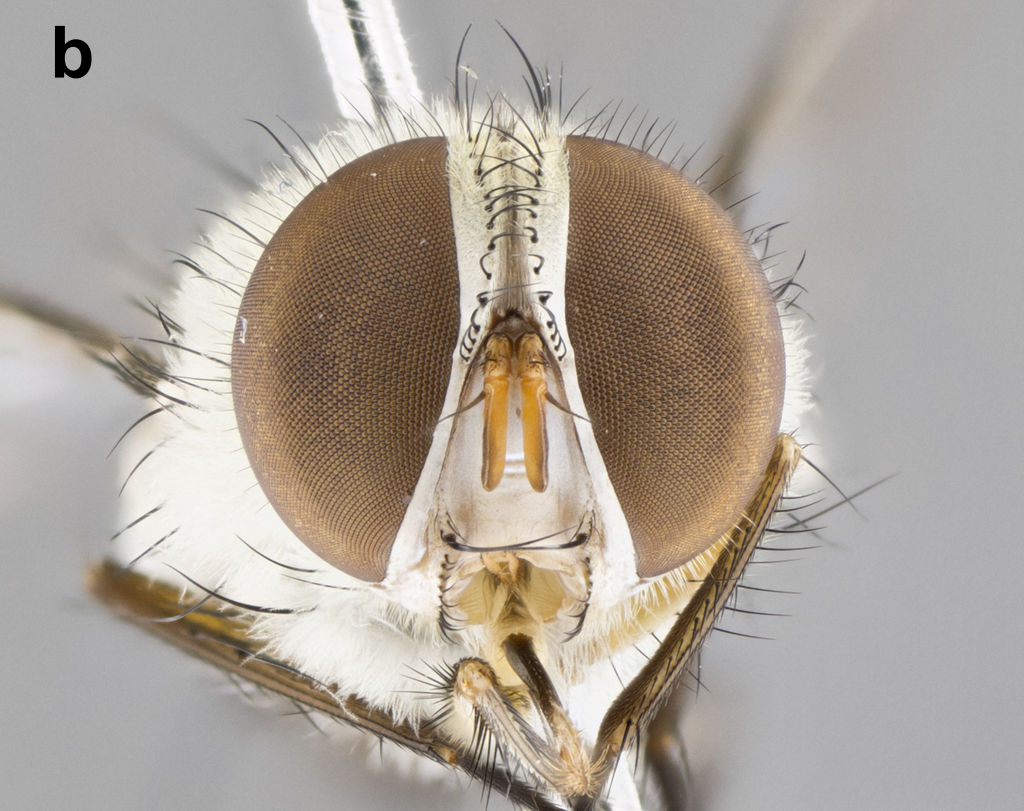
frontal view.

**Figure 34c.**
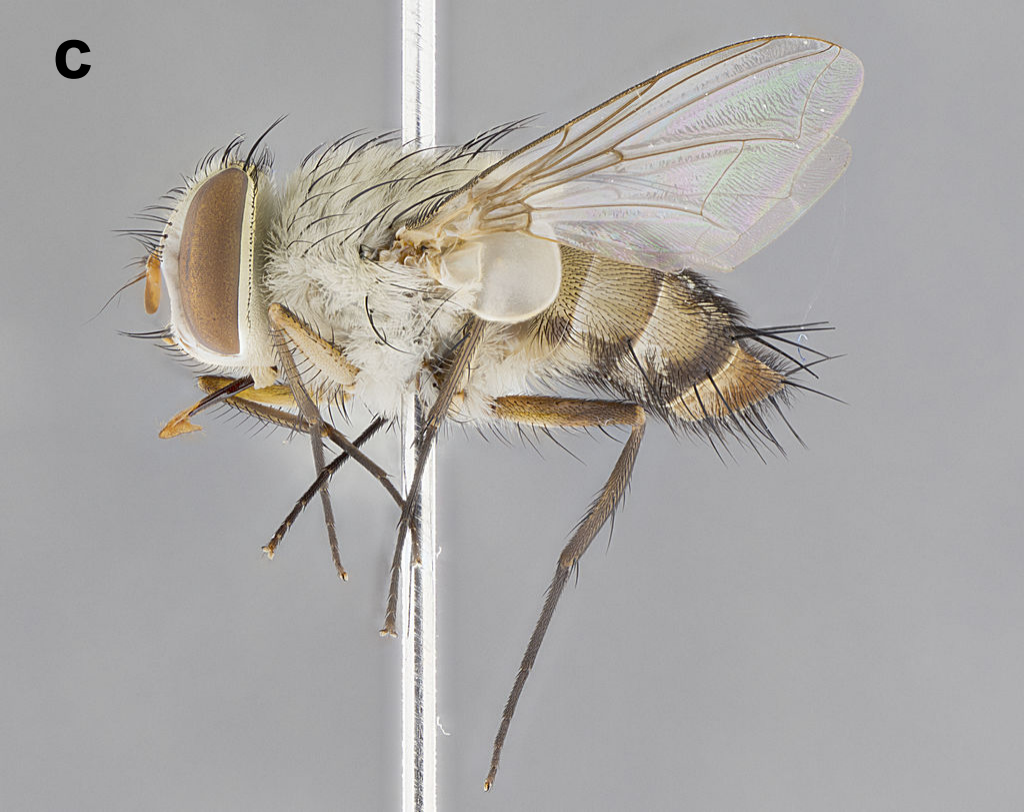
lateral view.

**Figure 34d.**
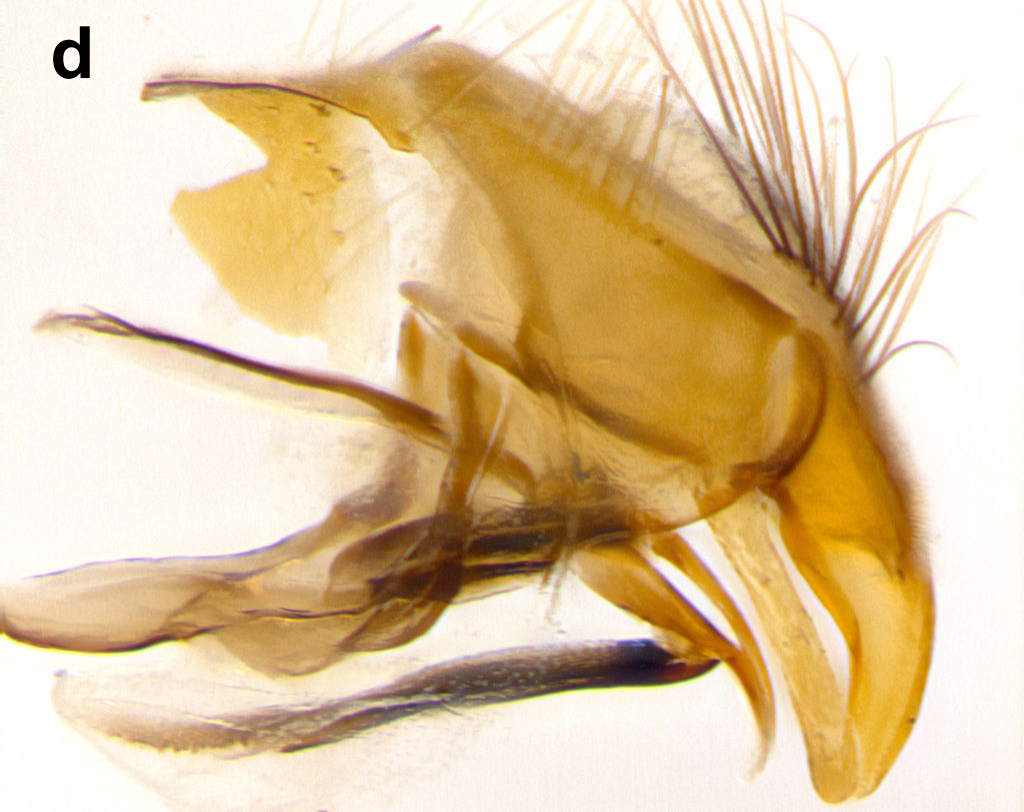
caudal view.

**Figure 34e.**
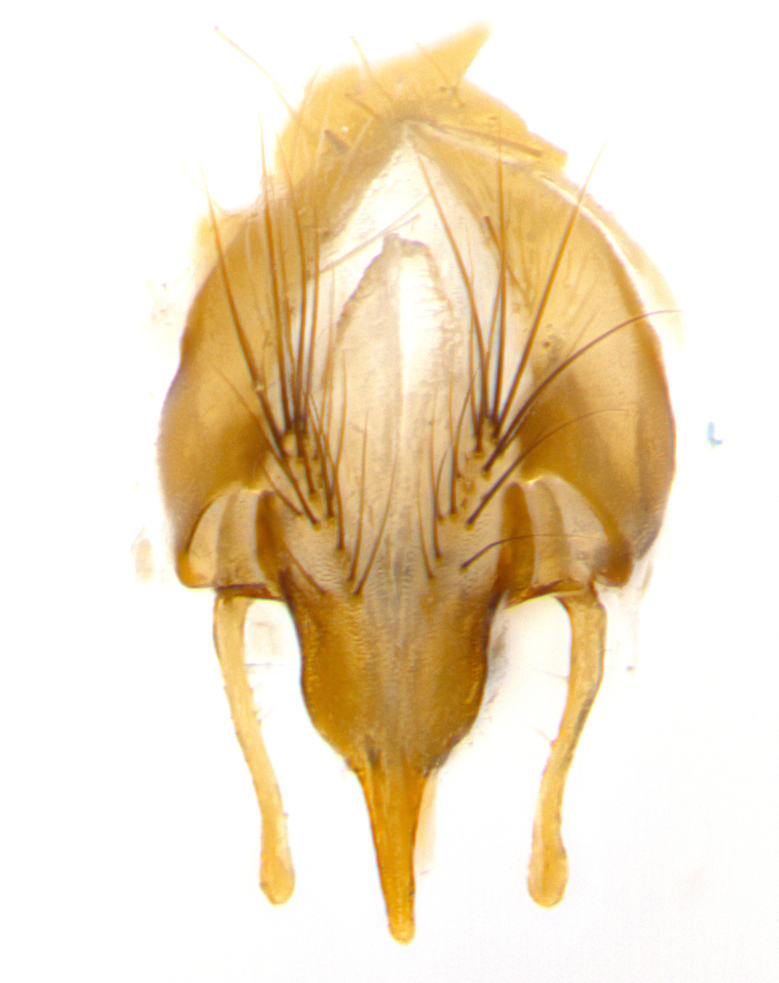
lateral view.

**Figure 34f.**
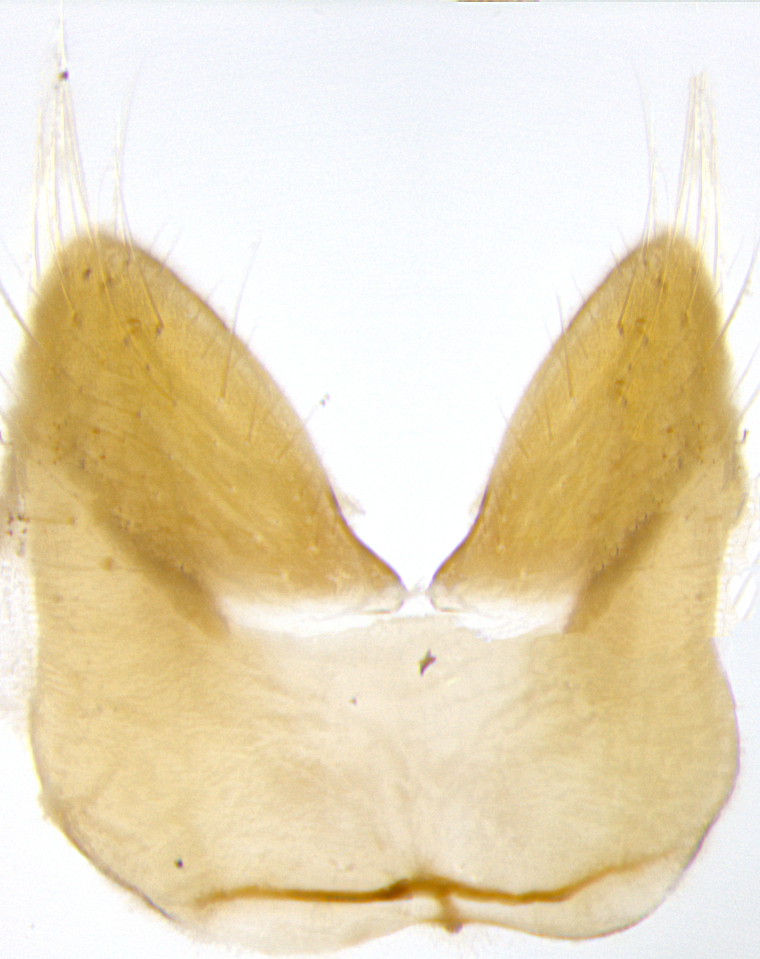
sternite 5, ventral view.

**Figure 35a.**
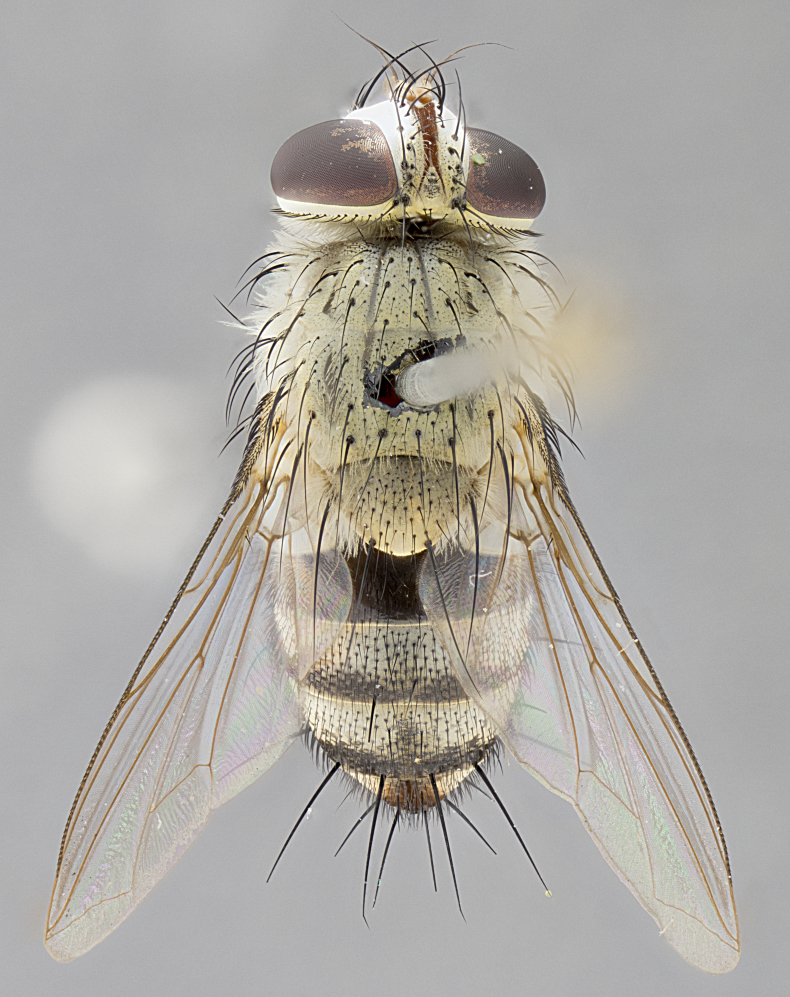
dorsal view.

**Figure 35b.**
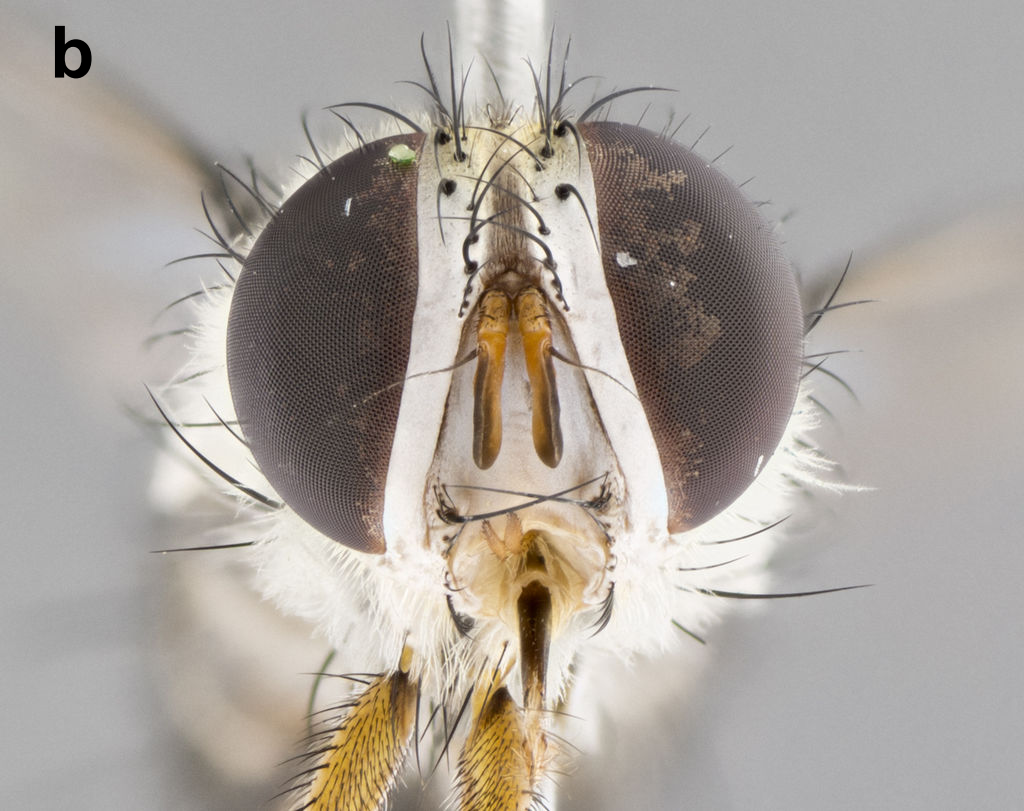
frontal view.

**Figure 35c.**
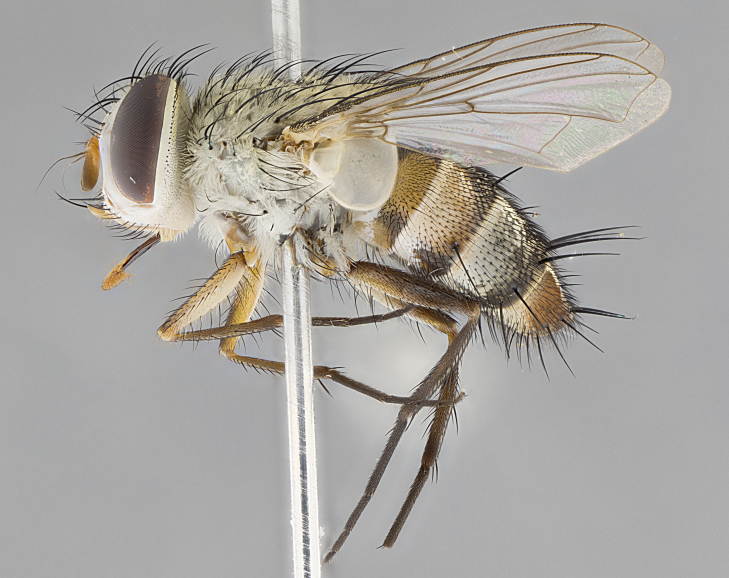
lateral view.

**Figure 36a.**
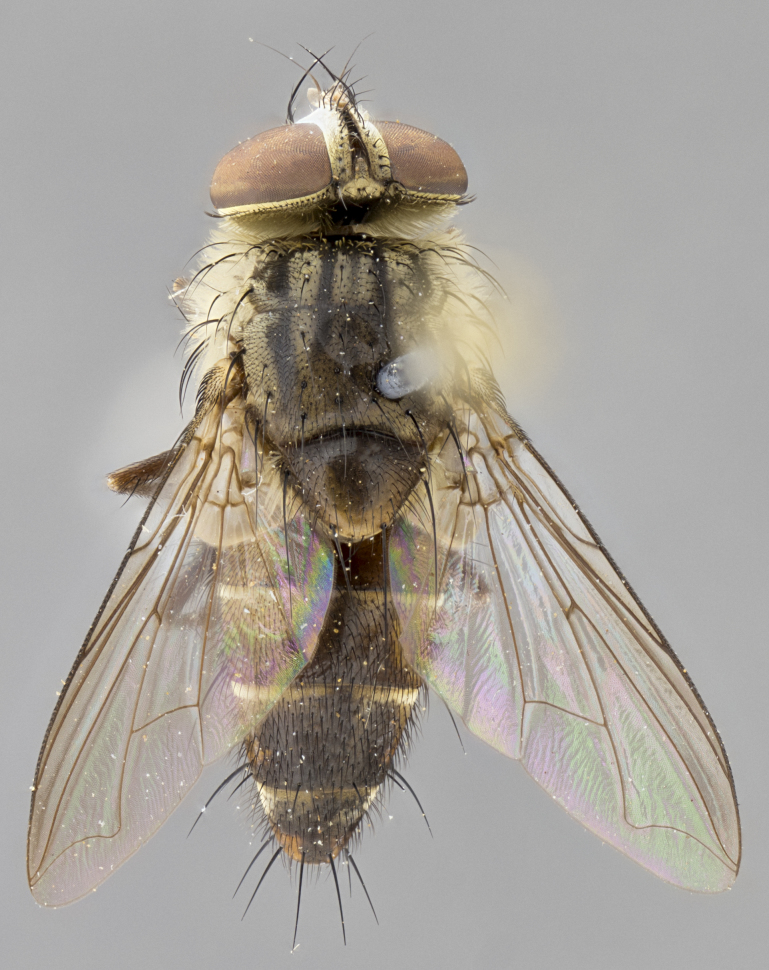
dorsal view.

**Figure 36b.**
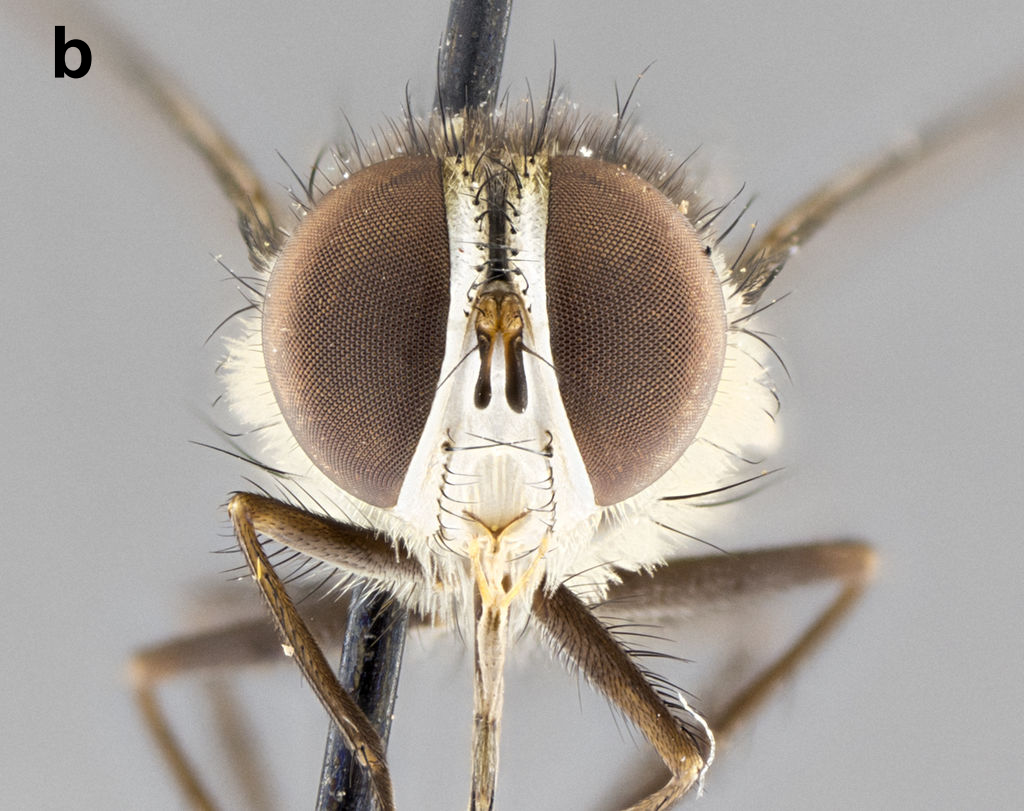
frontal view.

**Figure 36c.**
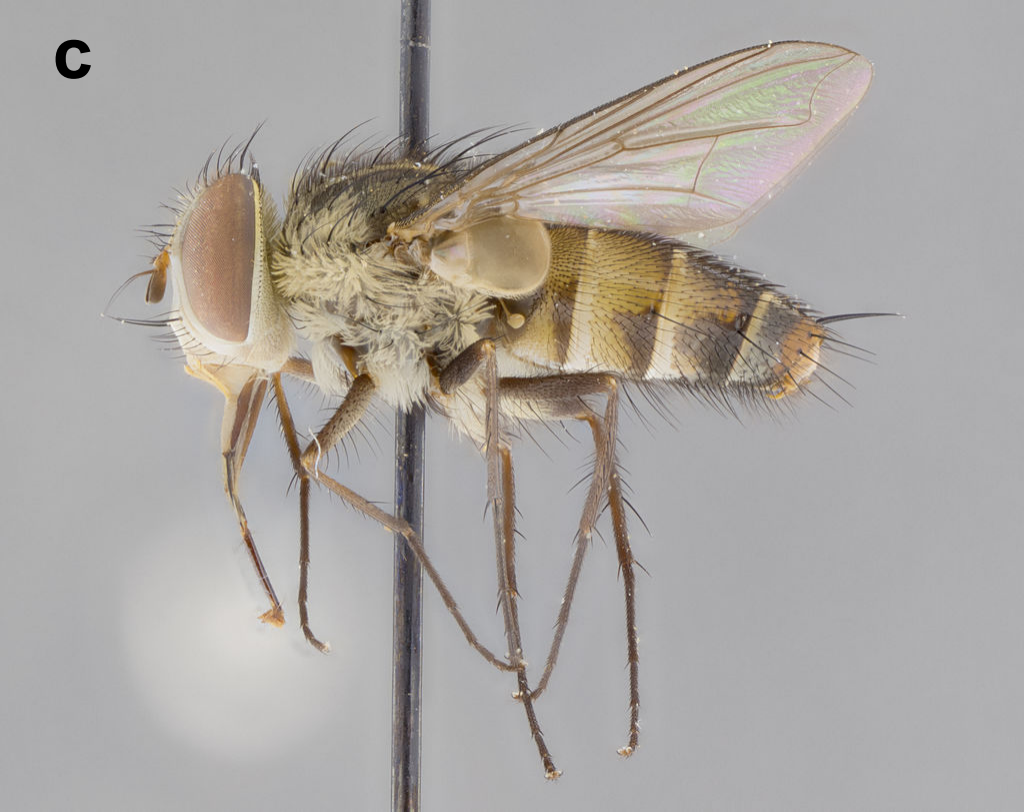
lateral view.

**Figure 36d.**
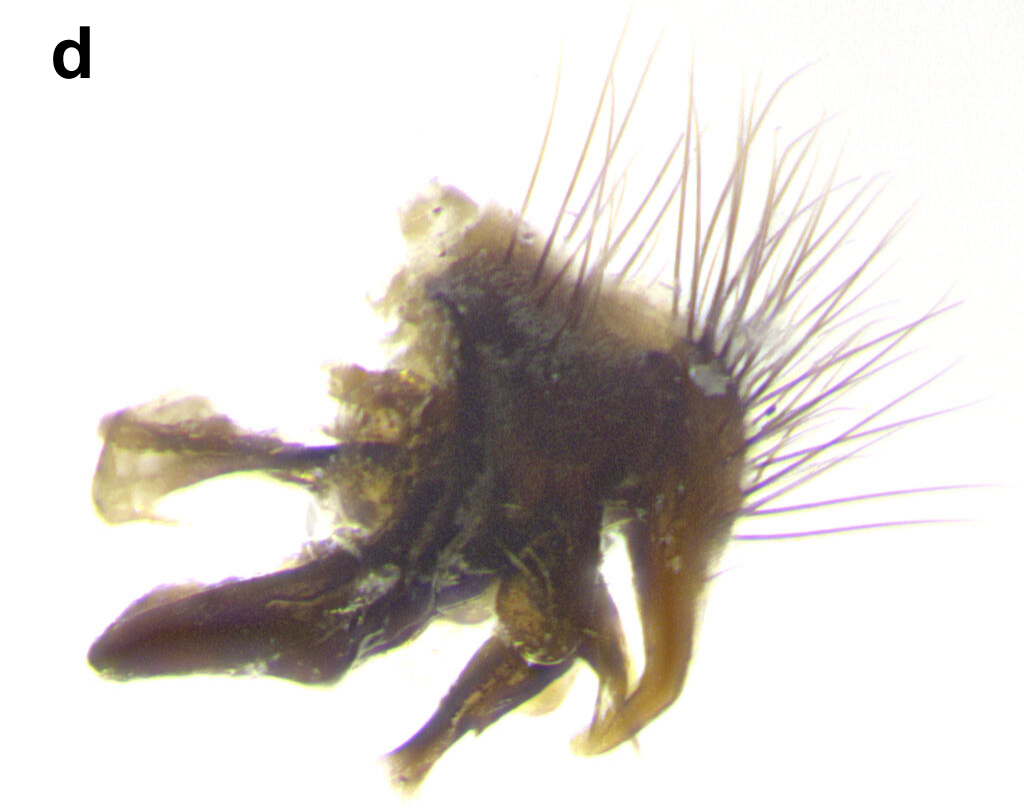
lateral view.

**Figure 36e.**
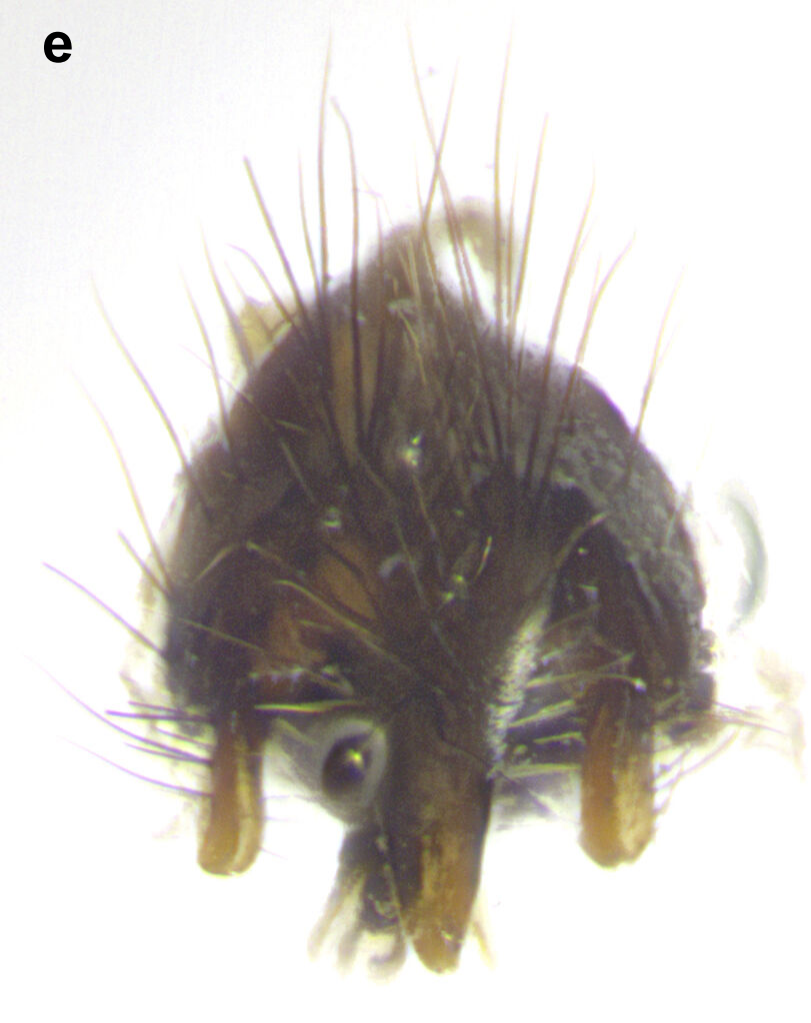
caudal view.

**Figure 36f.**
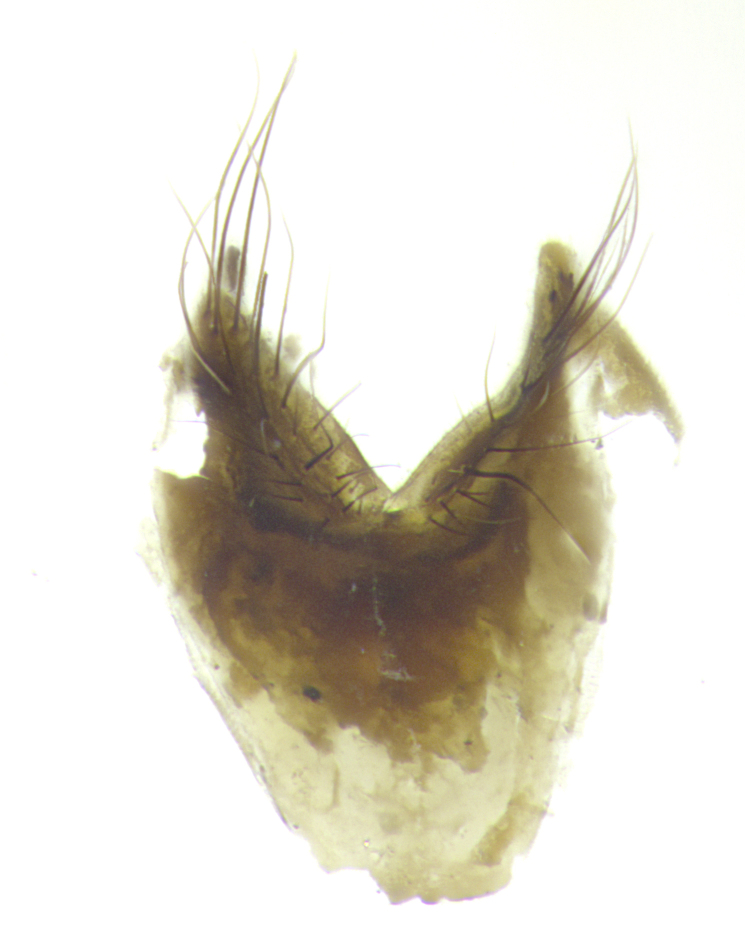
sternite 5, ventral view.

**Figure 37a.**
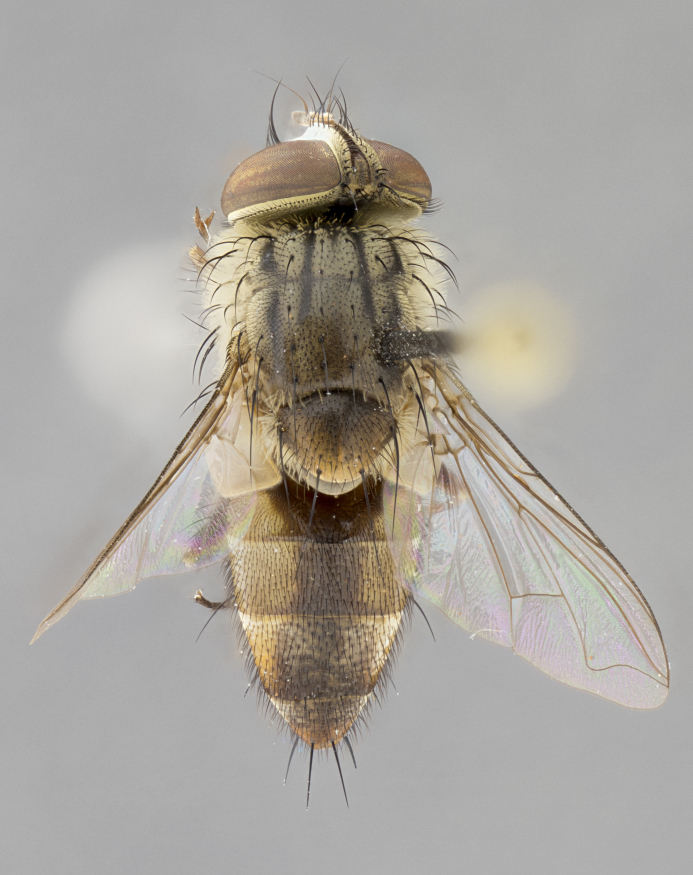
dorsal view.

**Figure 37b.**
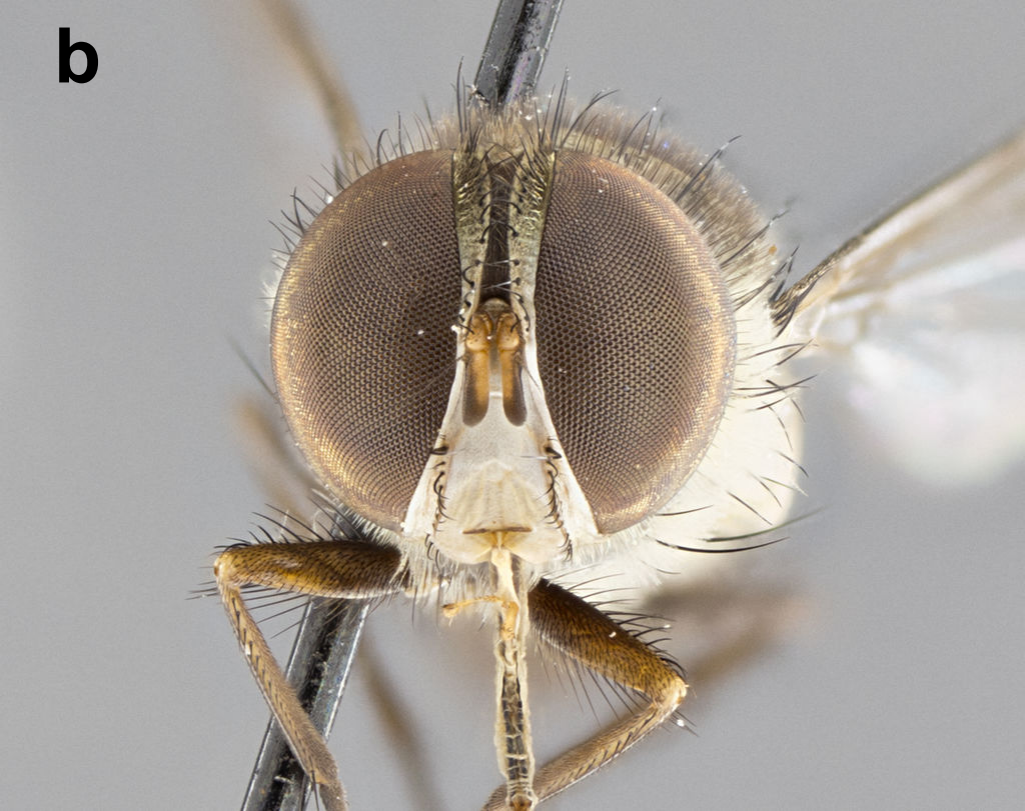
frontal view.

**Figure 37c.**
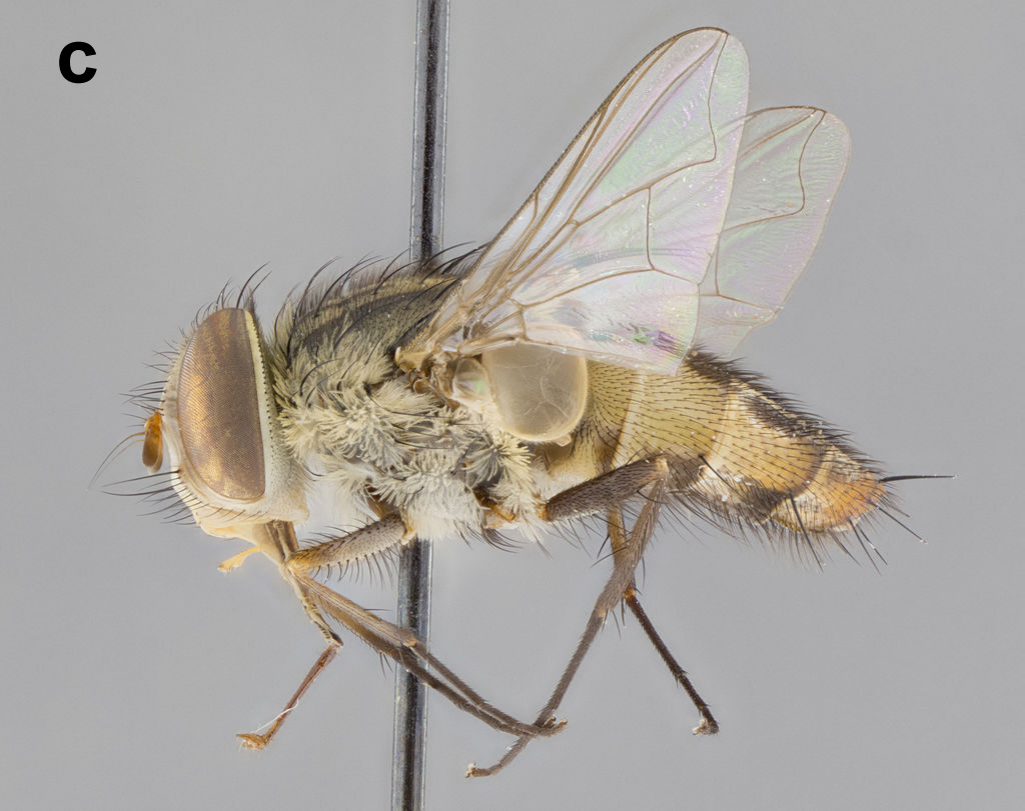
lateral view.

**Figure 38a.**
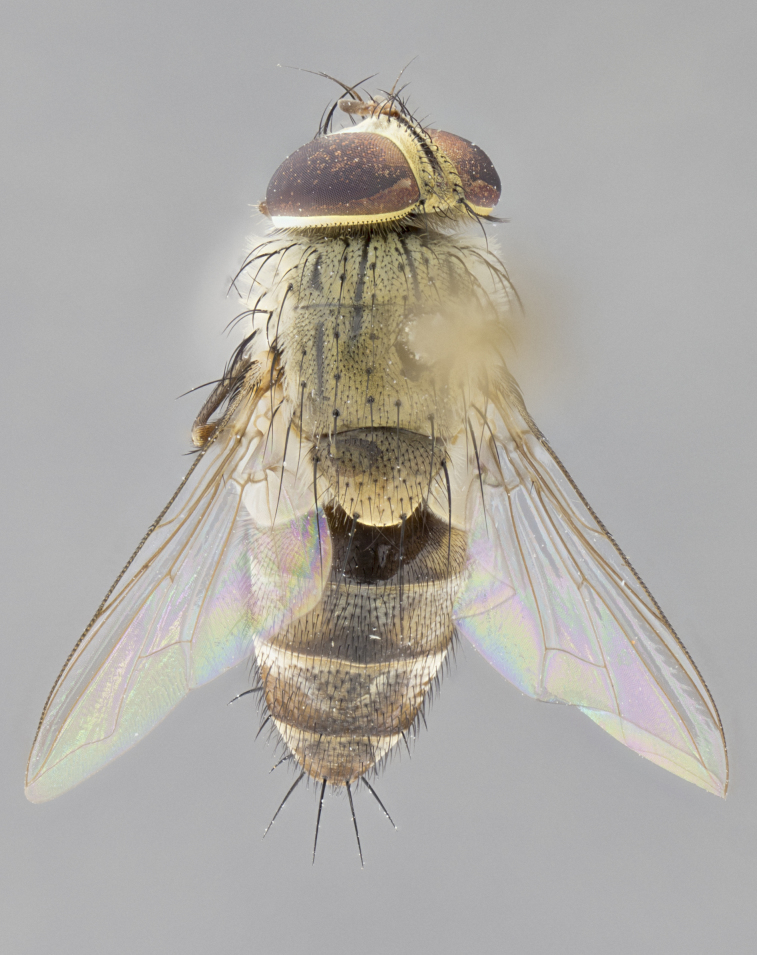
dorsal view.

**Figure 38b.**
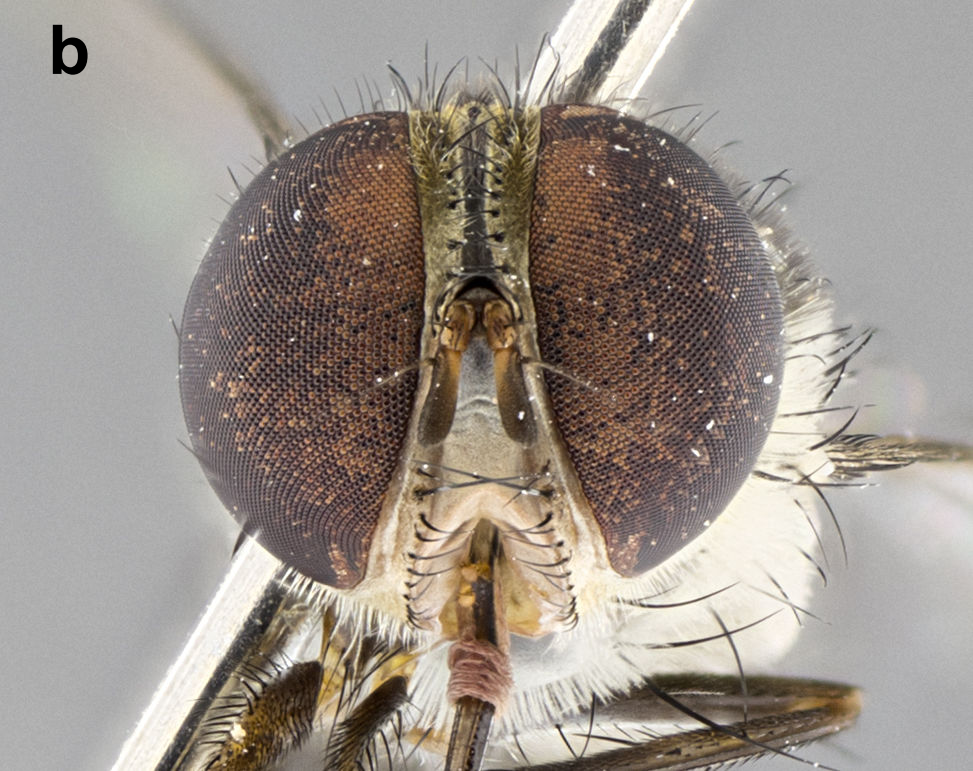
frontal view.

**Figure 38c.**
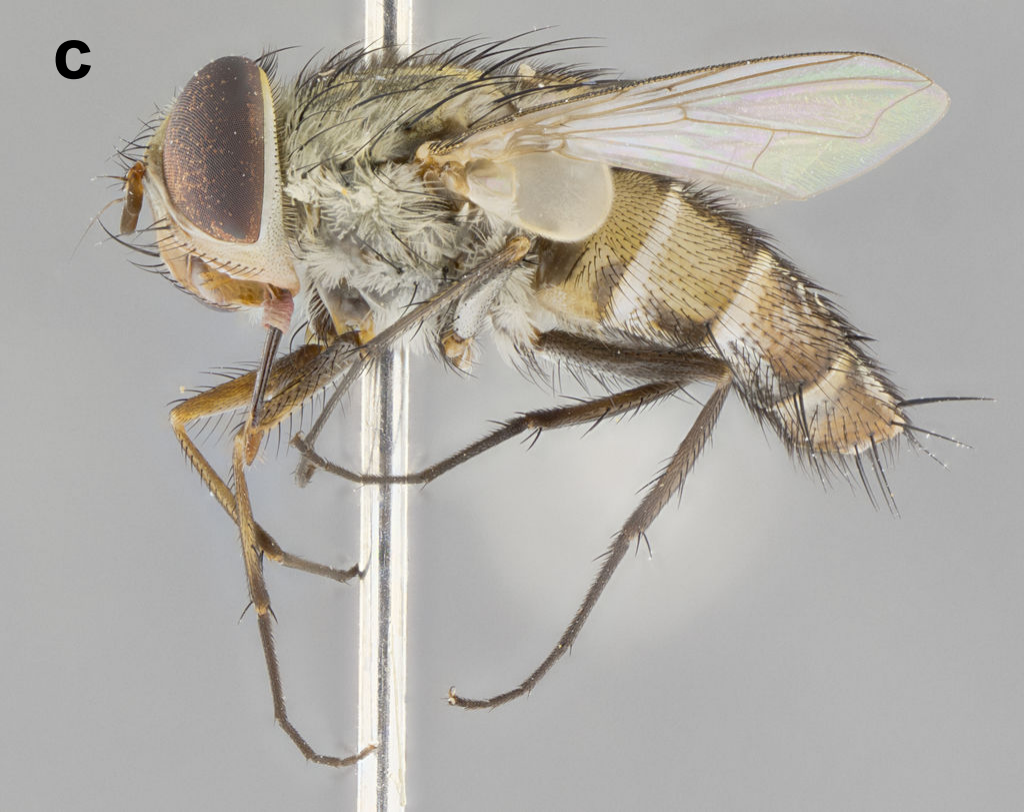
lateral view.

**Figure 39a.**
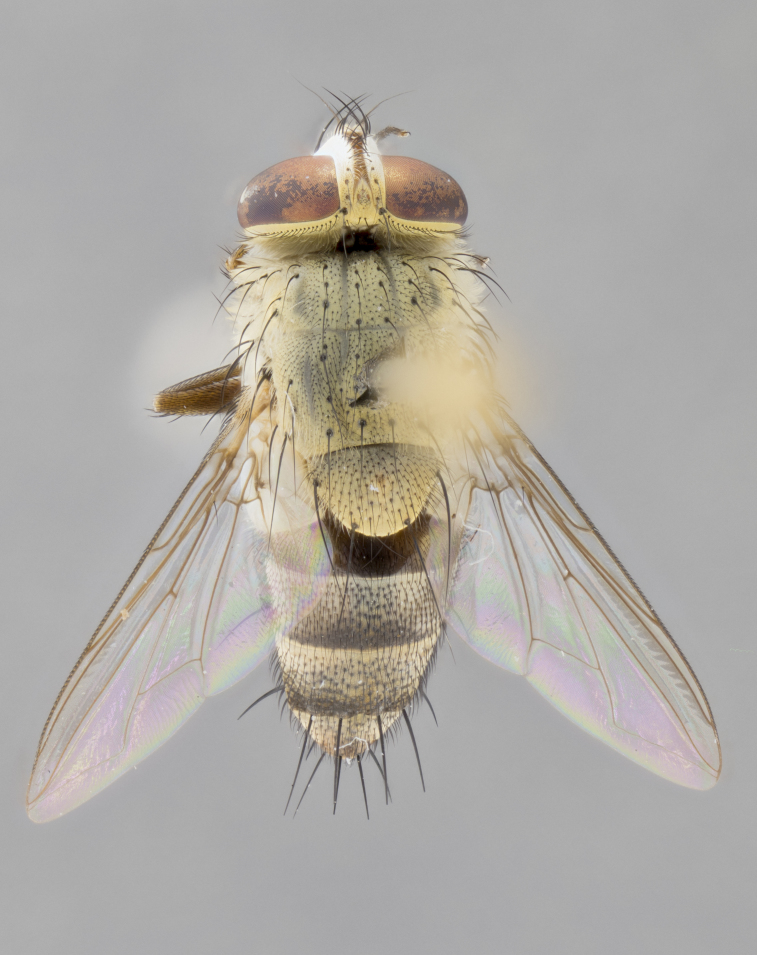
dorsal view.

**Figure 39b.**
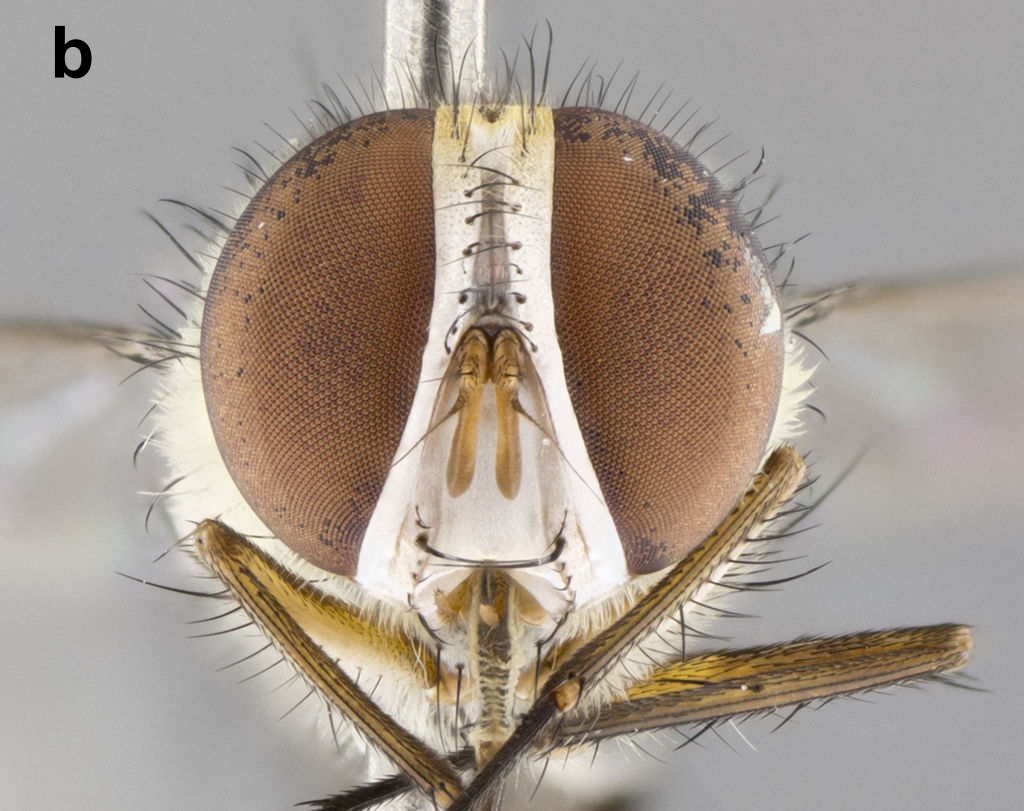
frontal view.

**Figure 39c.**
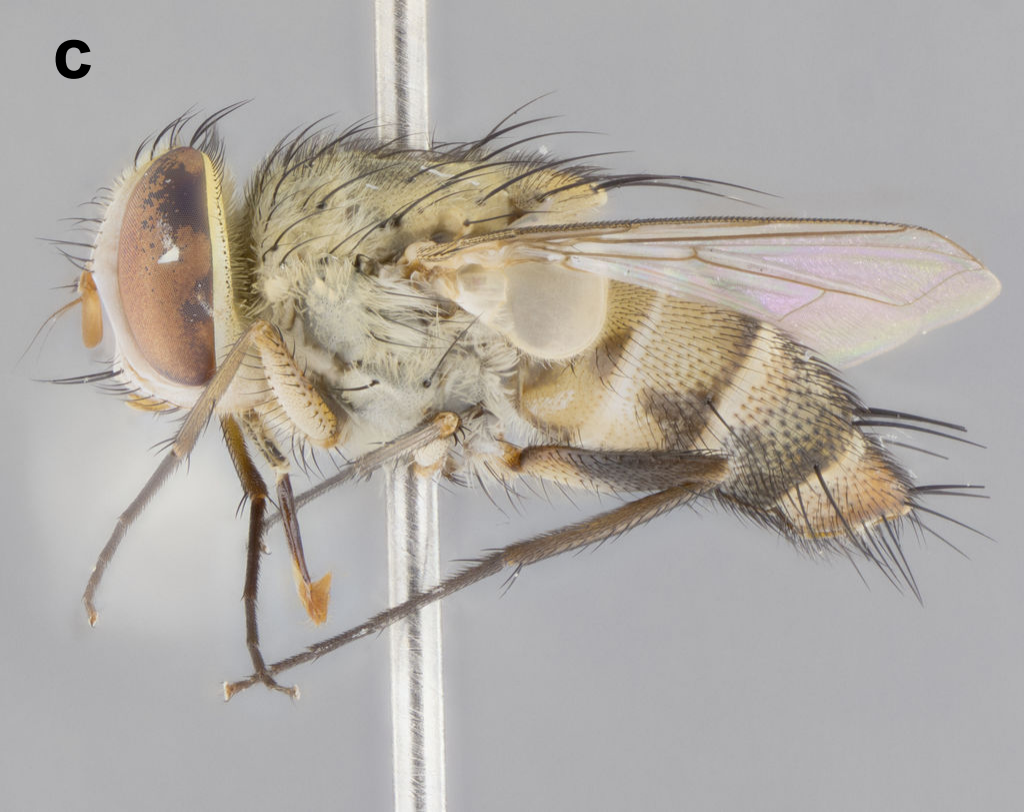
lateral view.

**Figure 39d.**
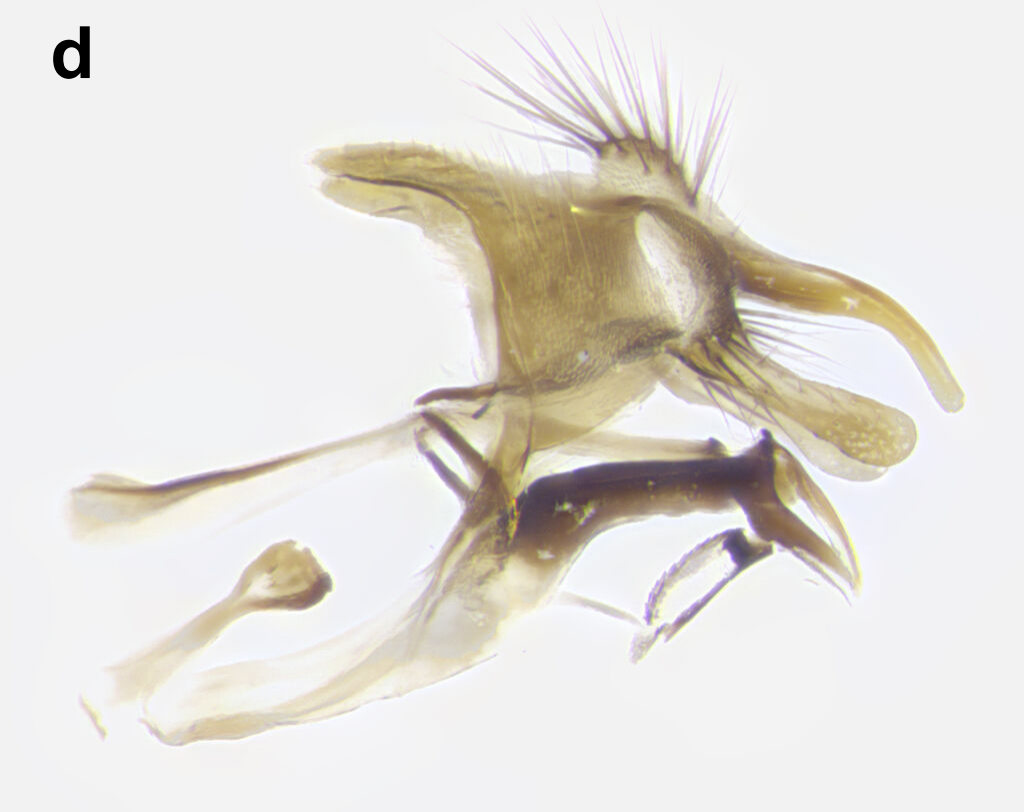
caudal view.

**Figure 39e.**
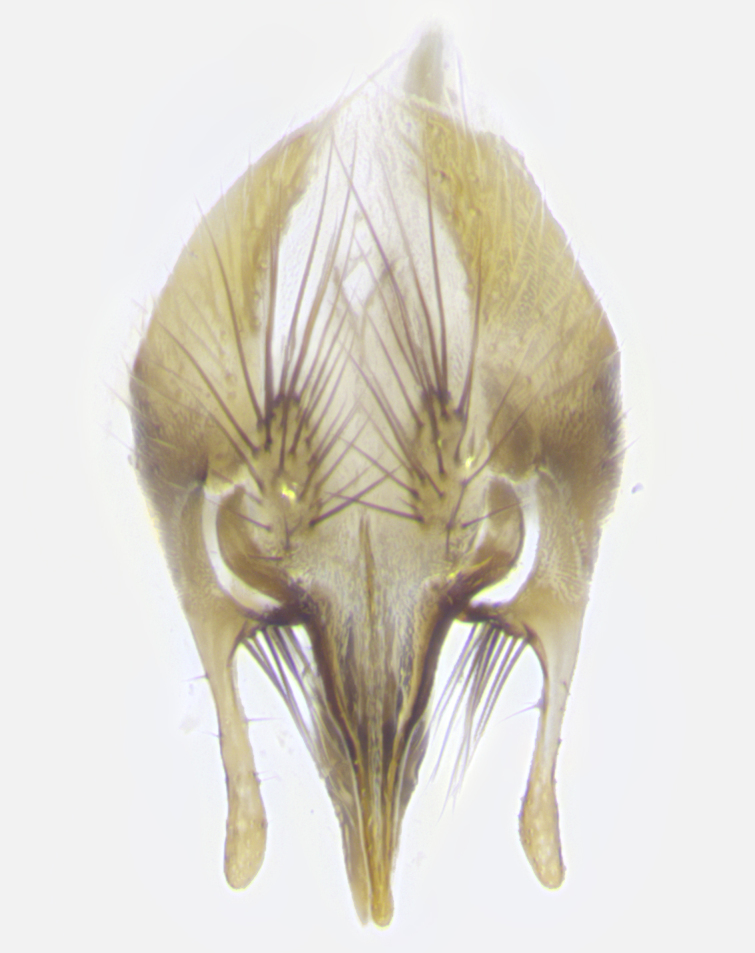
lateral view.

**Figure 39f.**
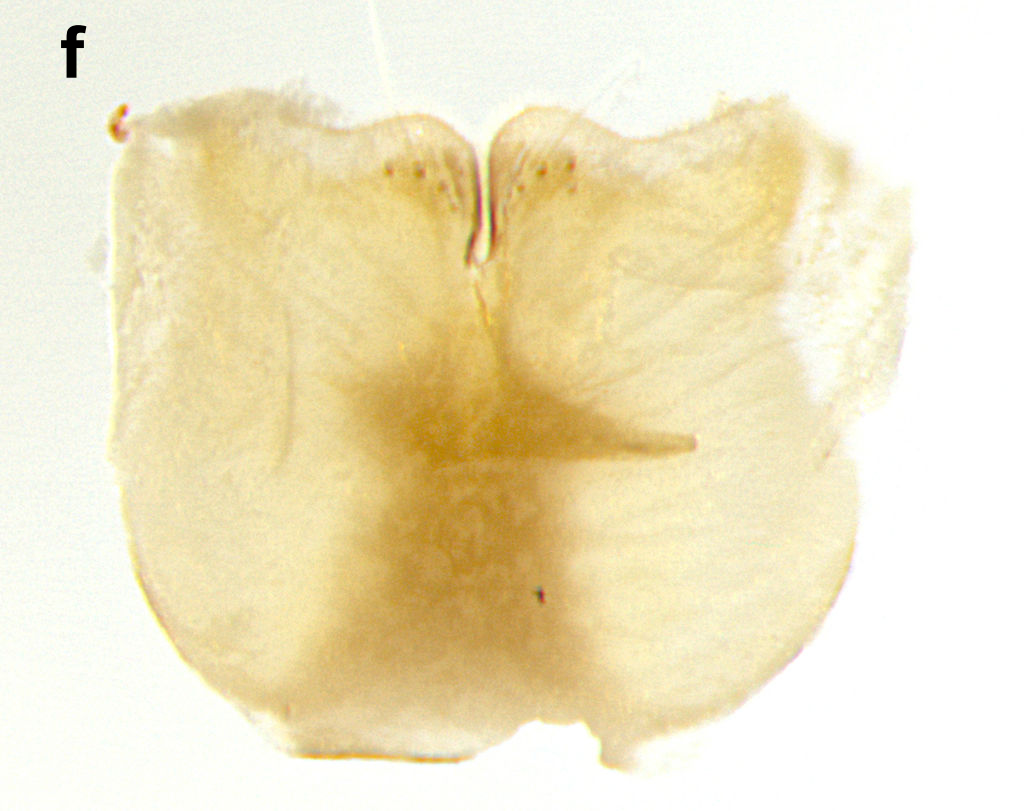
sternite 5, ventral view.

**Figure 40a.**
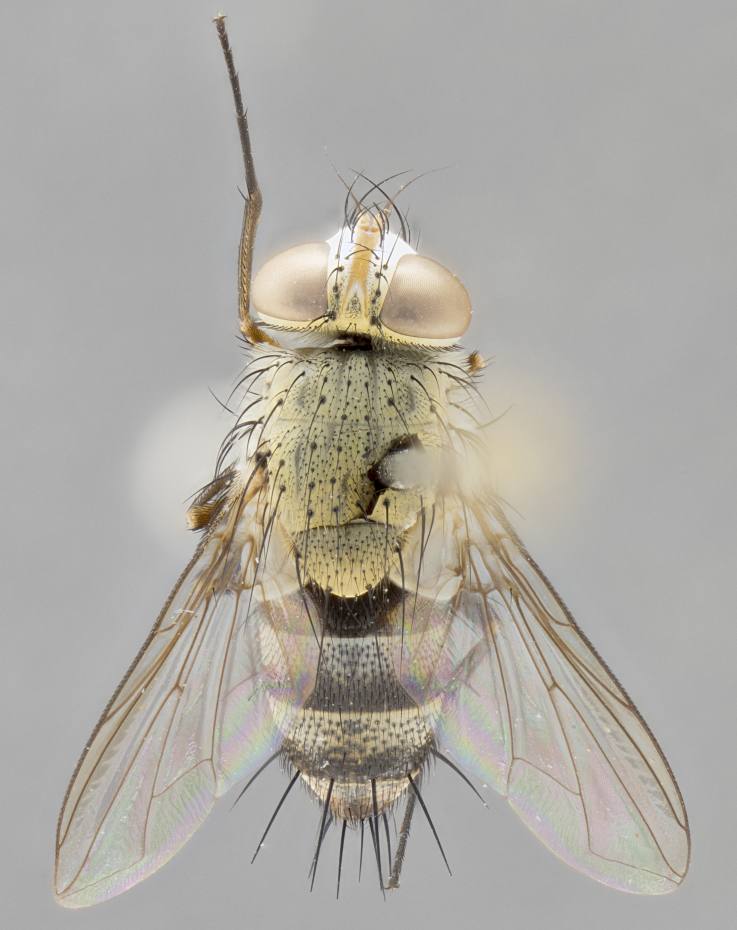
dorsal view.

**Figure 40b.**
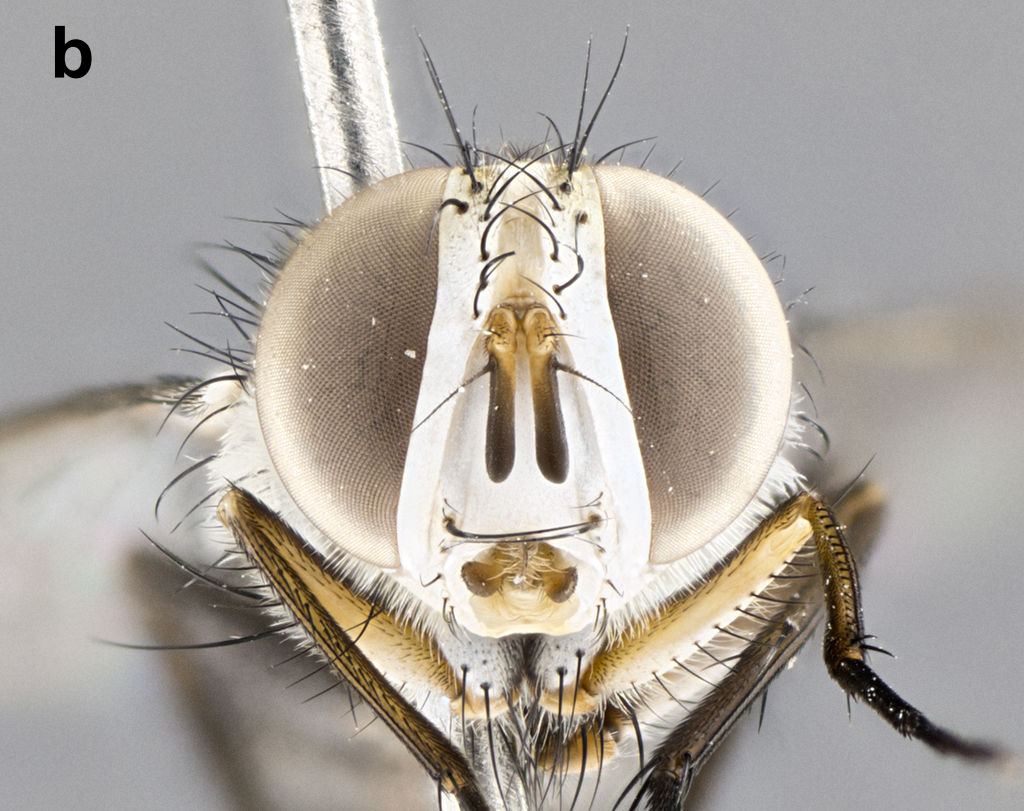
frontal view.

**Figure 40c.**
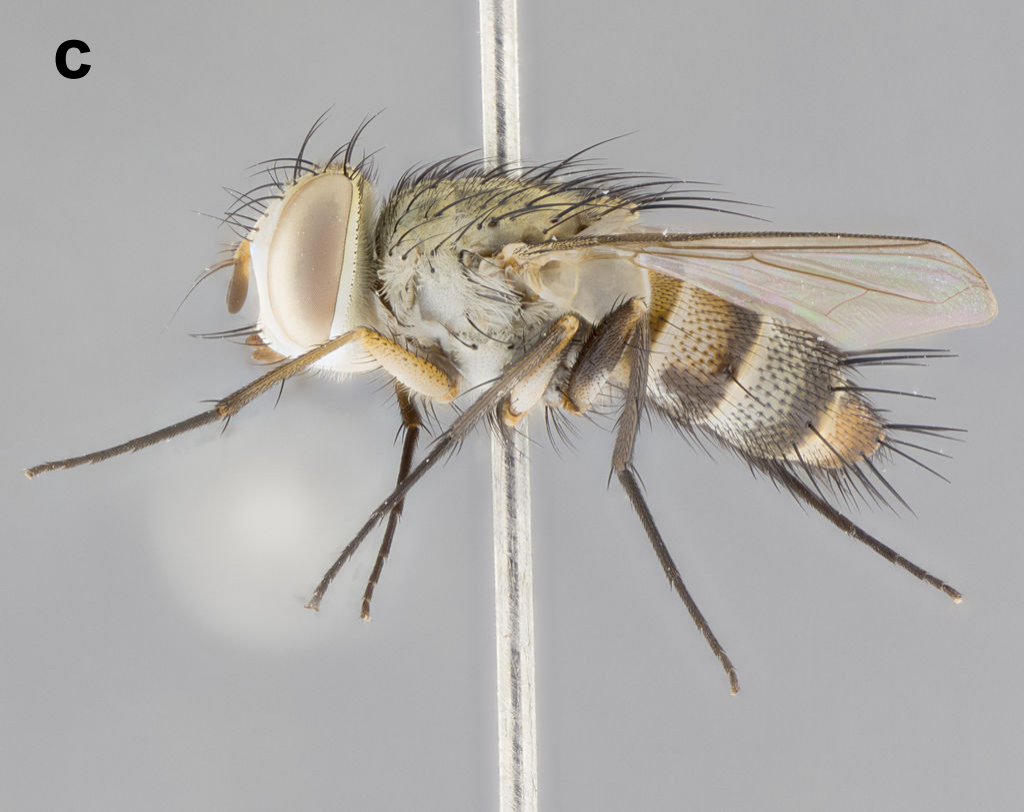
lateral view.

**Figure 41a.**
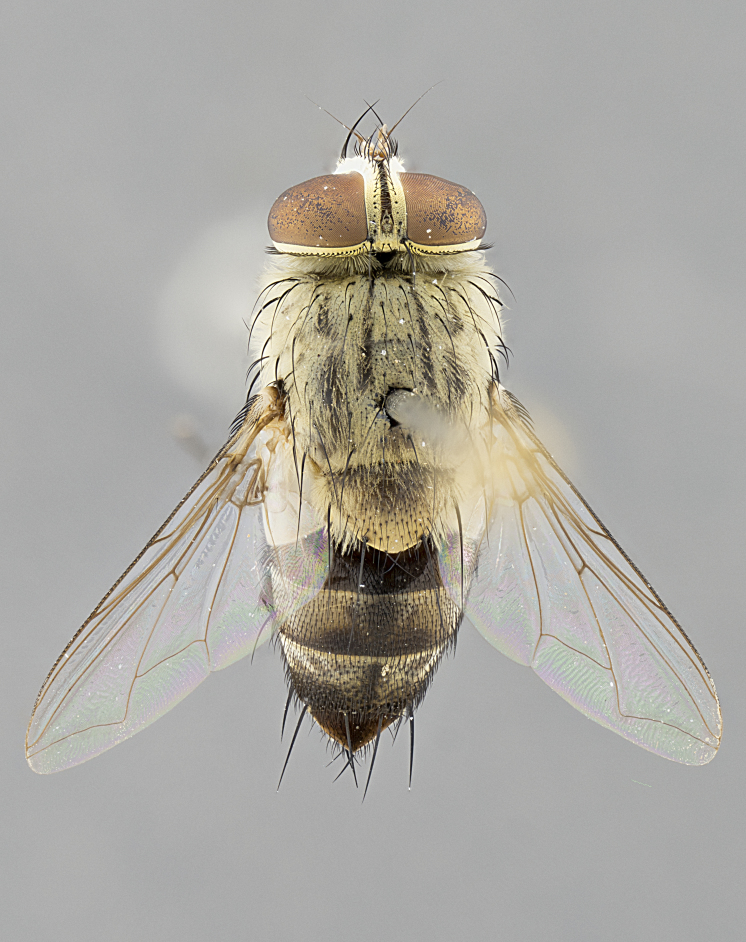
dorsal view.

**Figure 41b.**
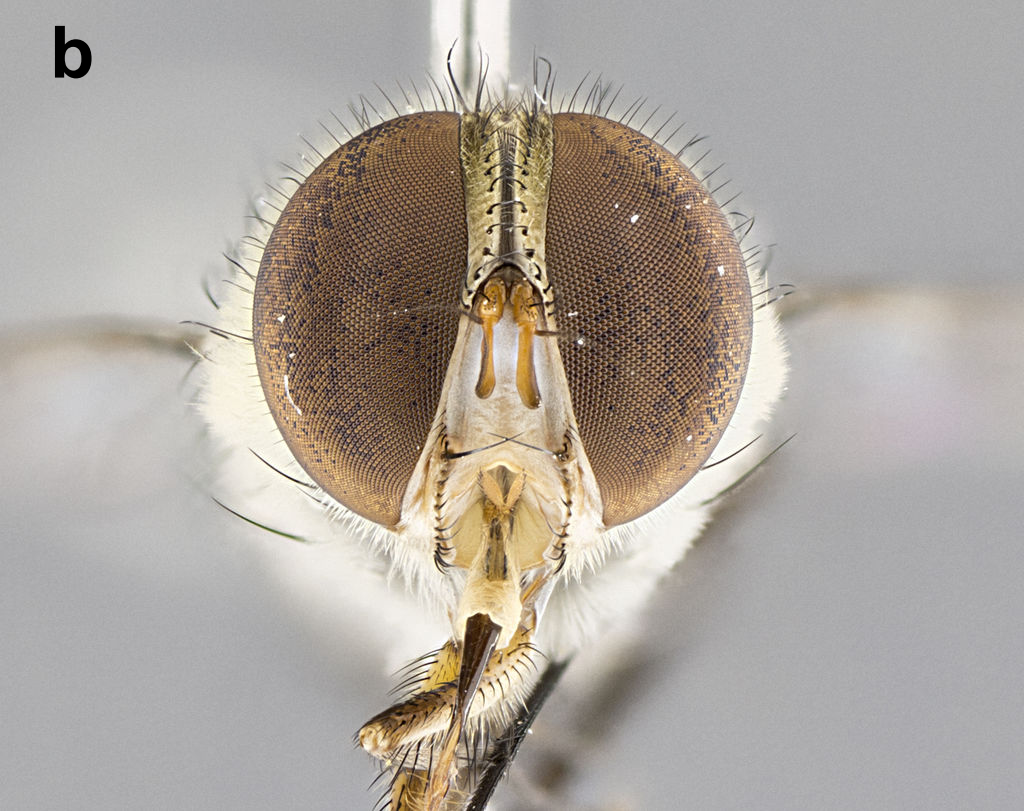
frontal view.

**Figure 41c.**
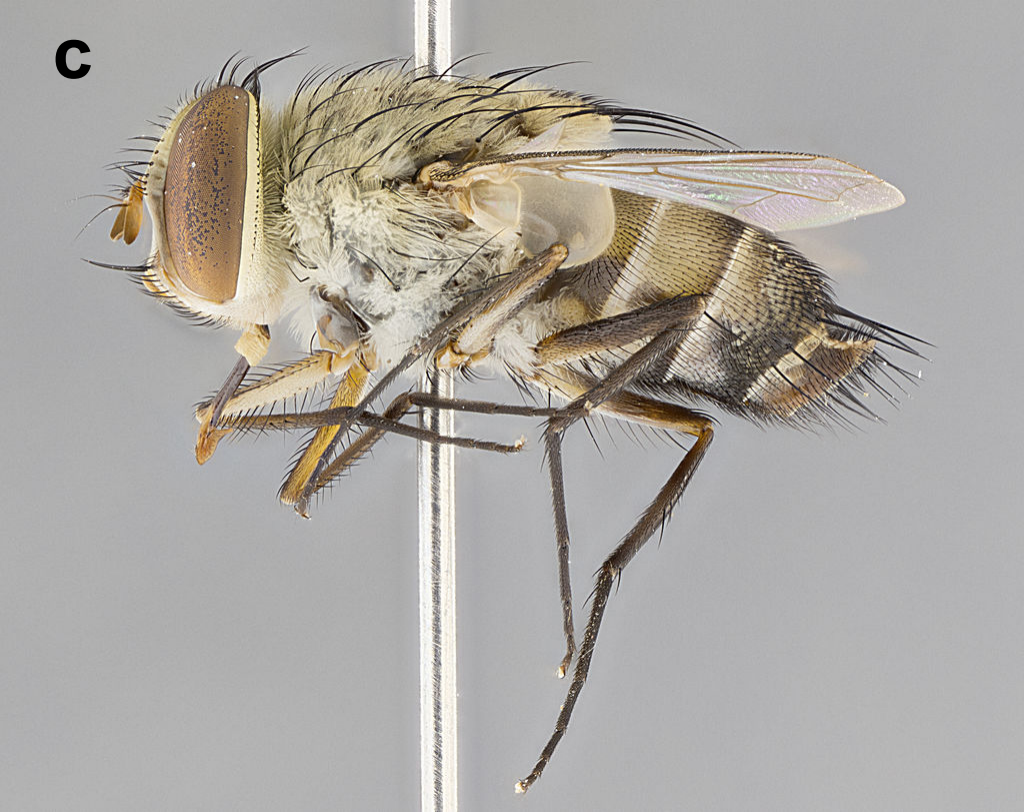
lateral view.

**Figure 41d.**
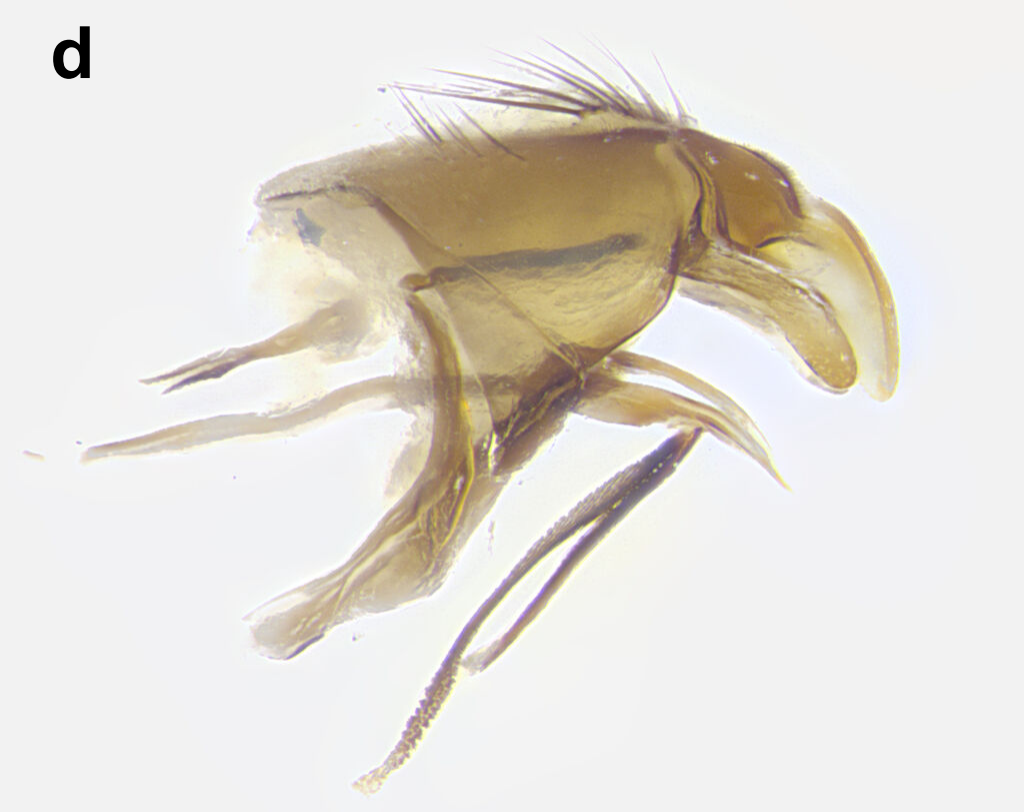
caudal view.

**Figure 41e.**
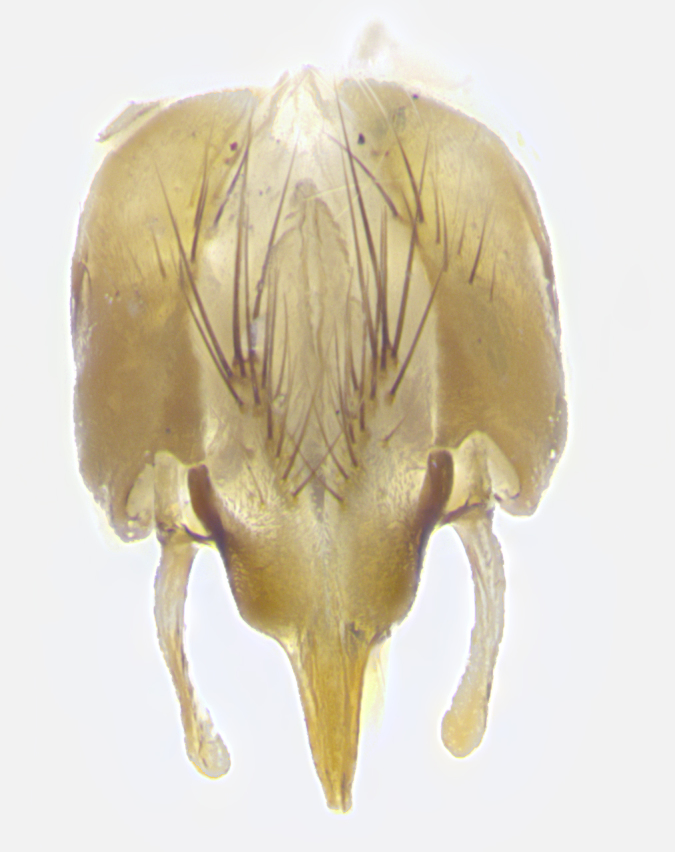
lateral view.

**Figure 41f.**
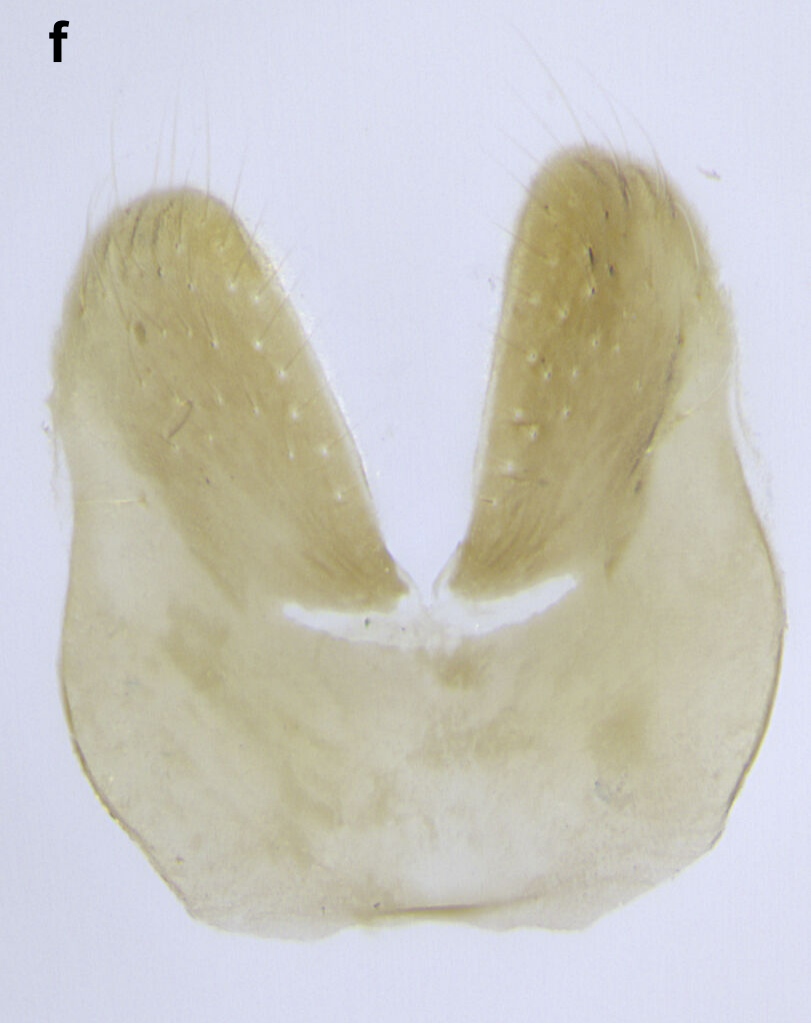
sternite 5, ventral view.

**Figure 42a.**
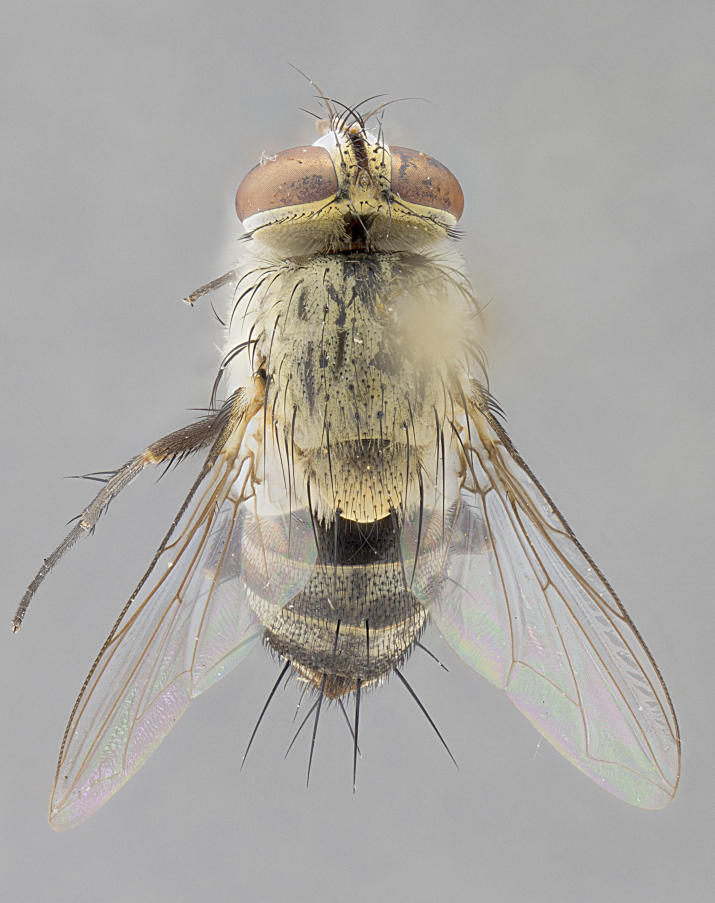
dorsal view.

**Figure 42b.**
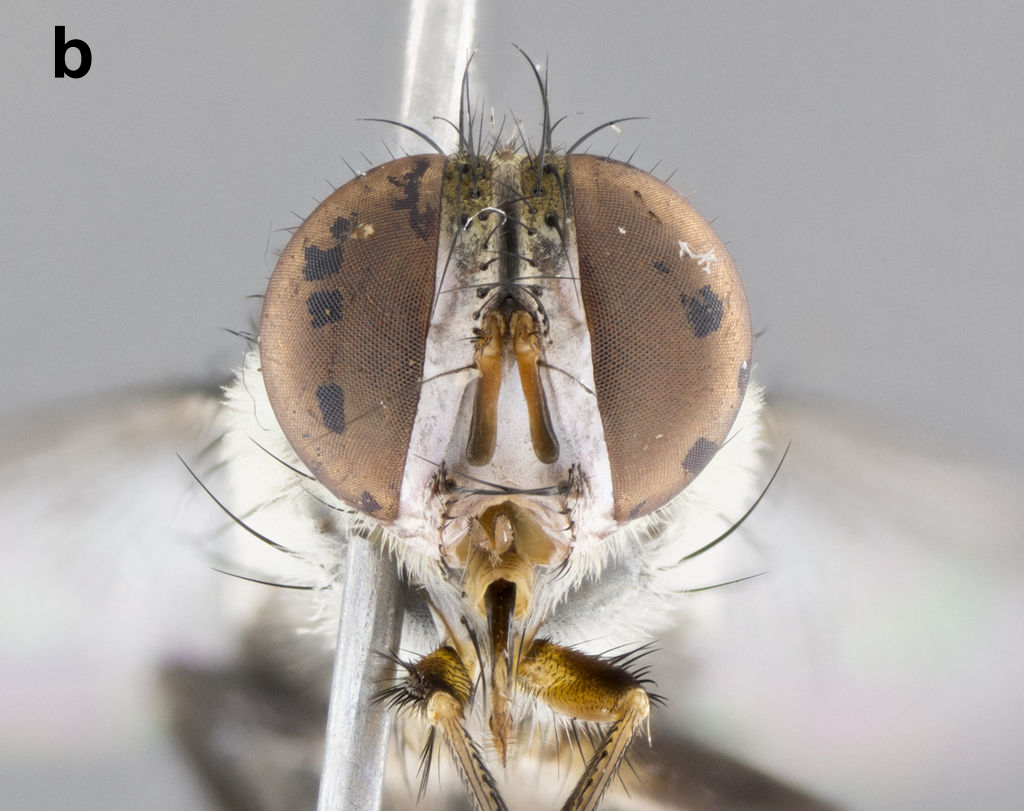
frontal view.

**Figure 42c.**
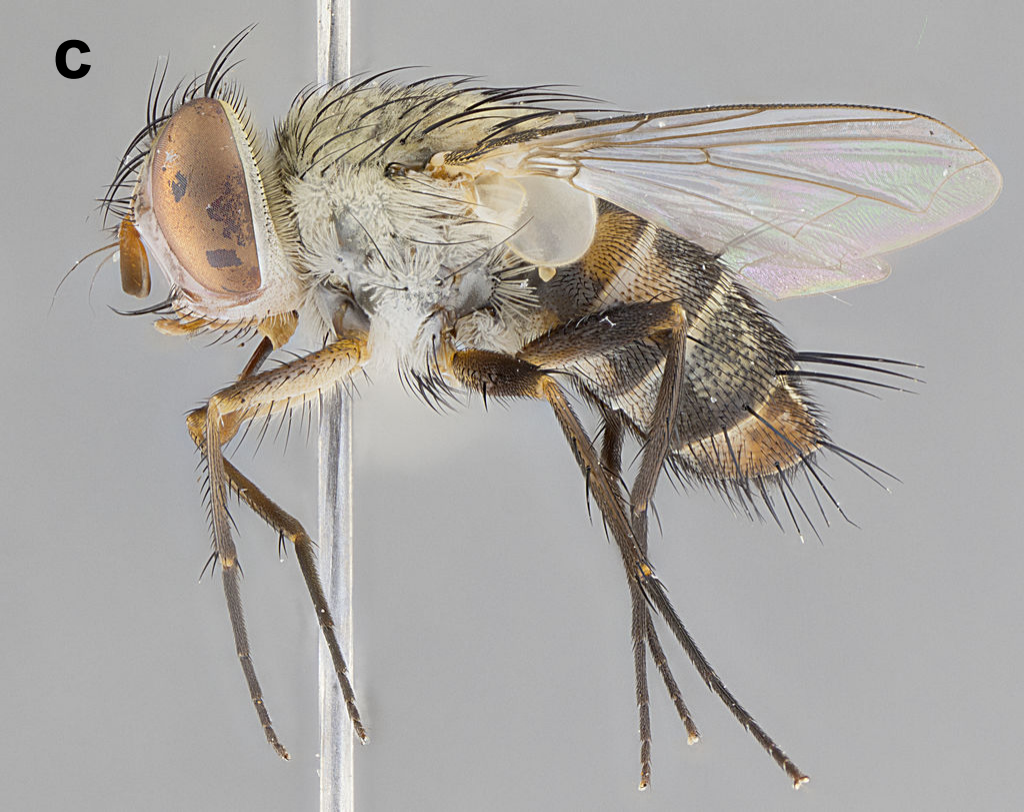
lateral view.

**Figure 43a.**
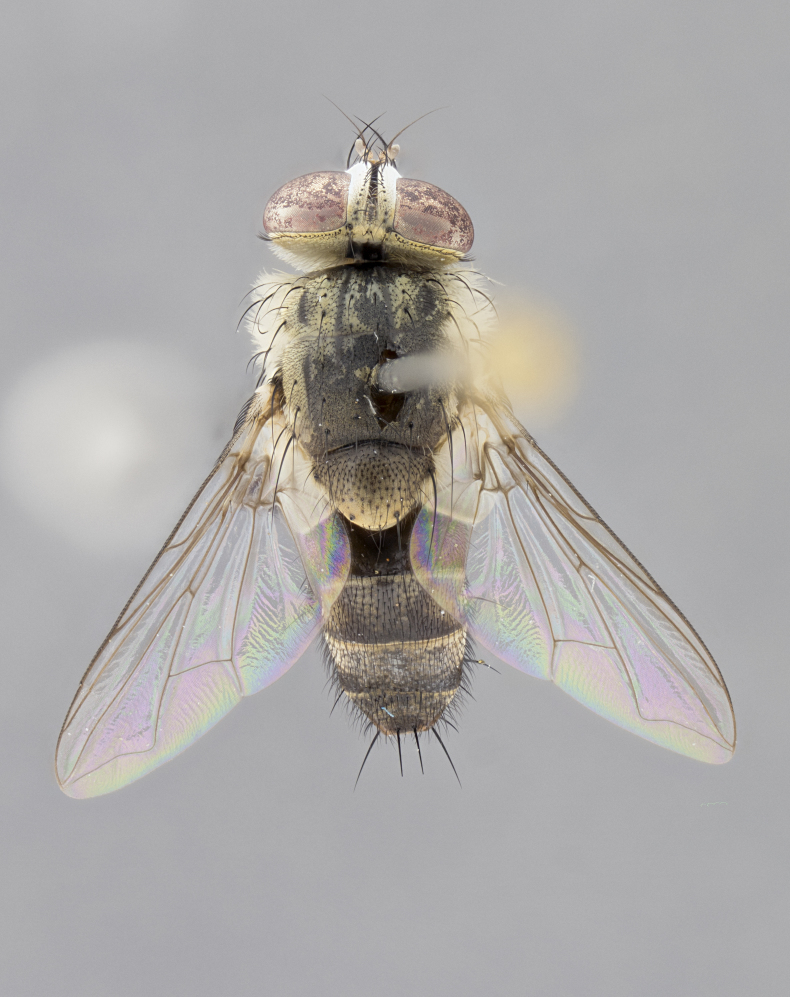
dorsal view

**Figure 43b.**
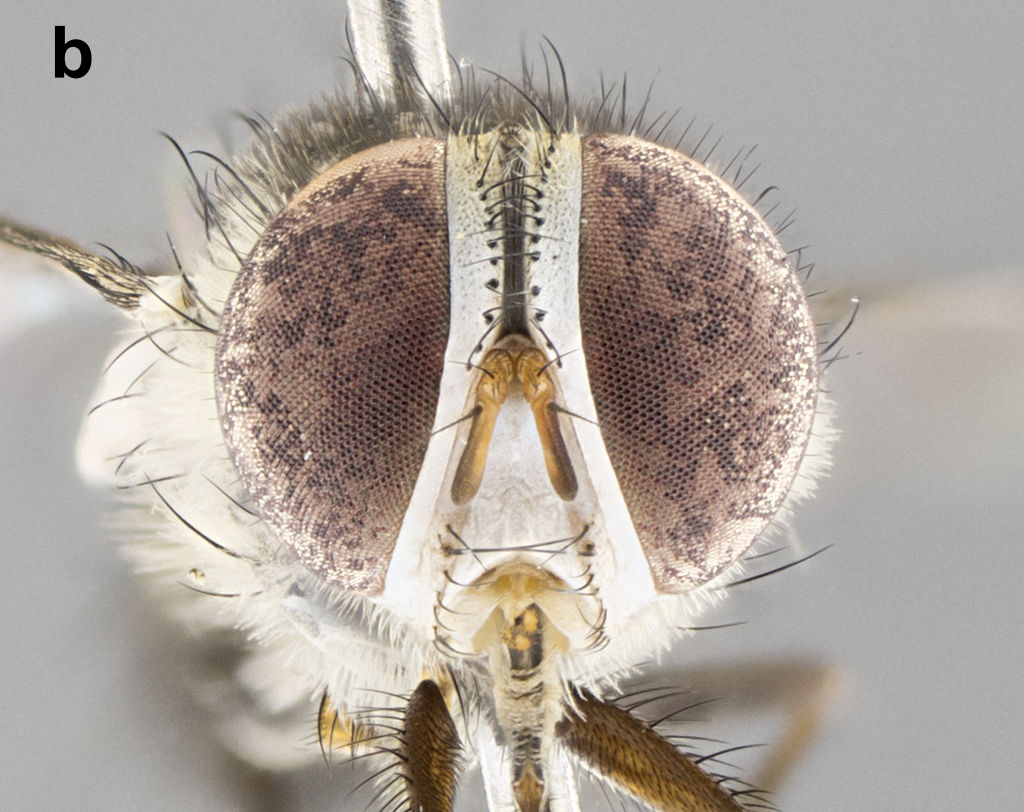
frontal view.

**Figure 43c.**
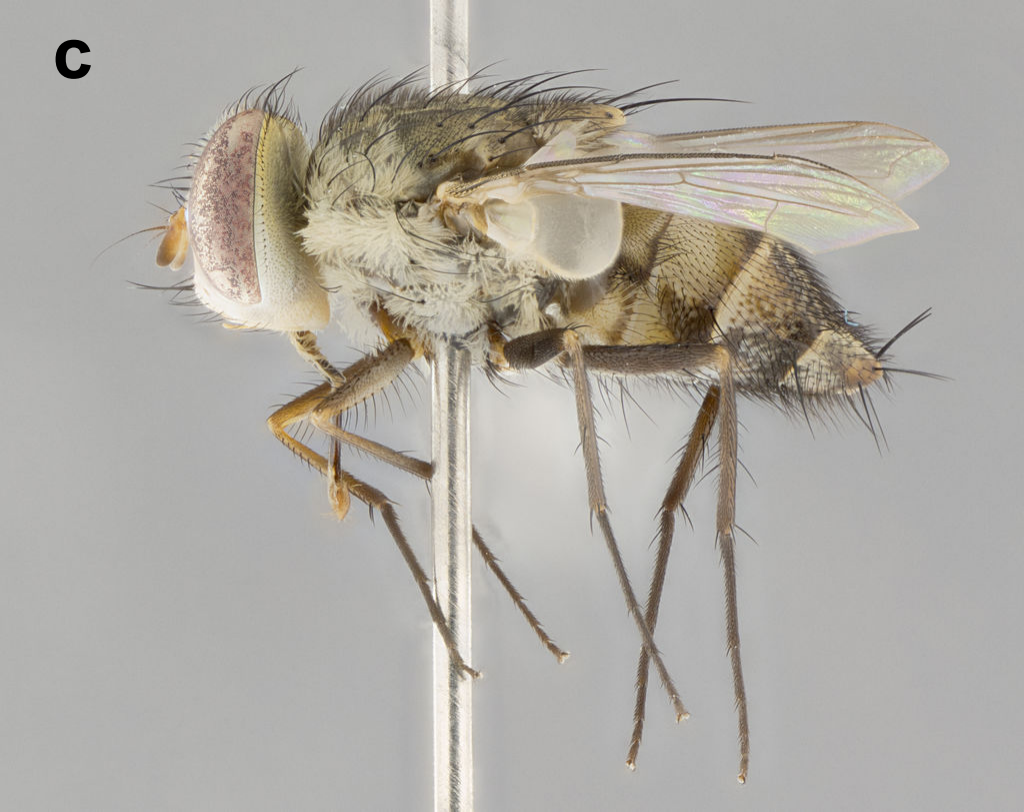
lateral view.

**Figure 43d.**
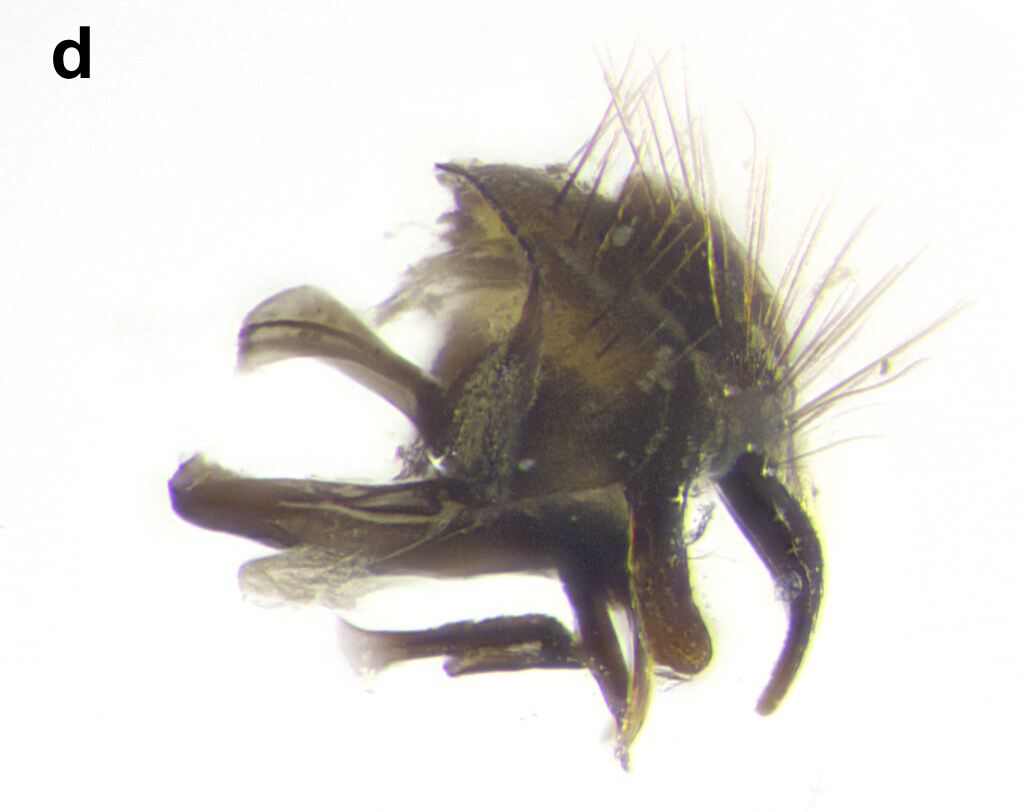
lateral view.

**Figure 43e.**
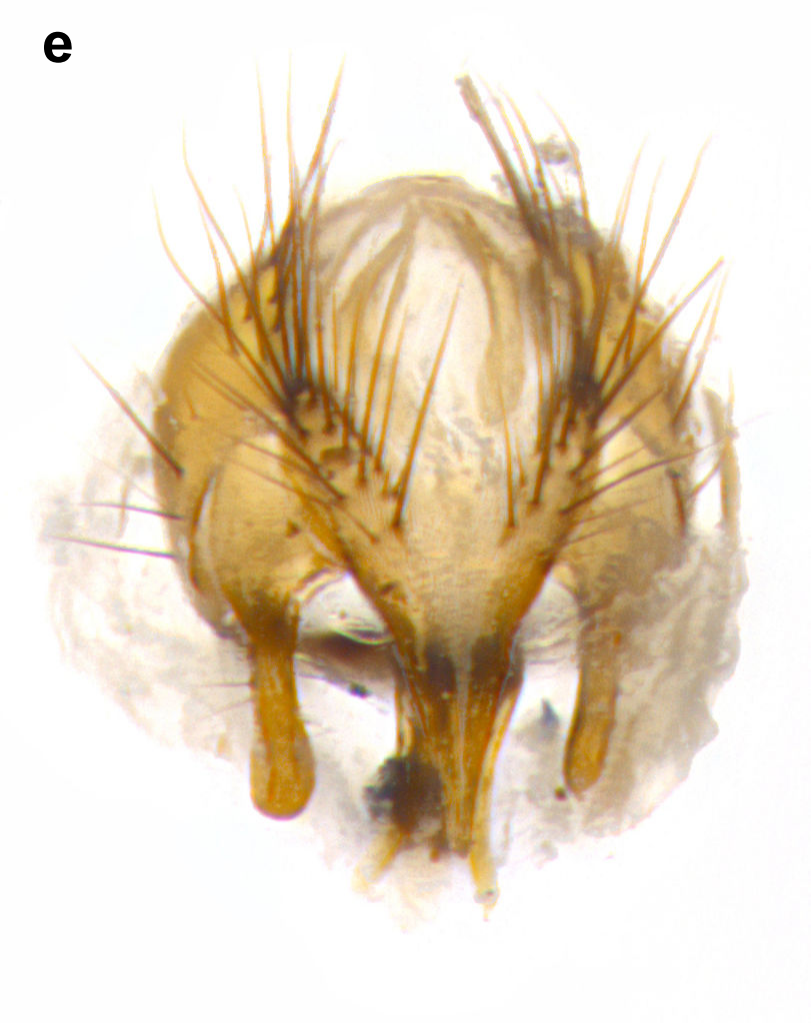
caudal view.

**Figure 43f.**
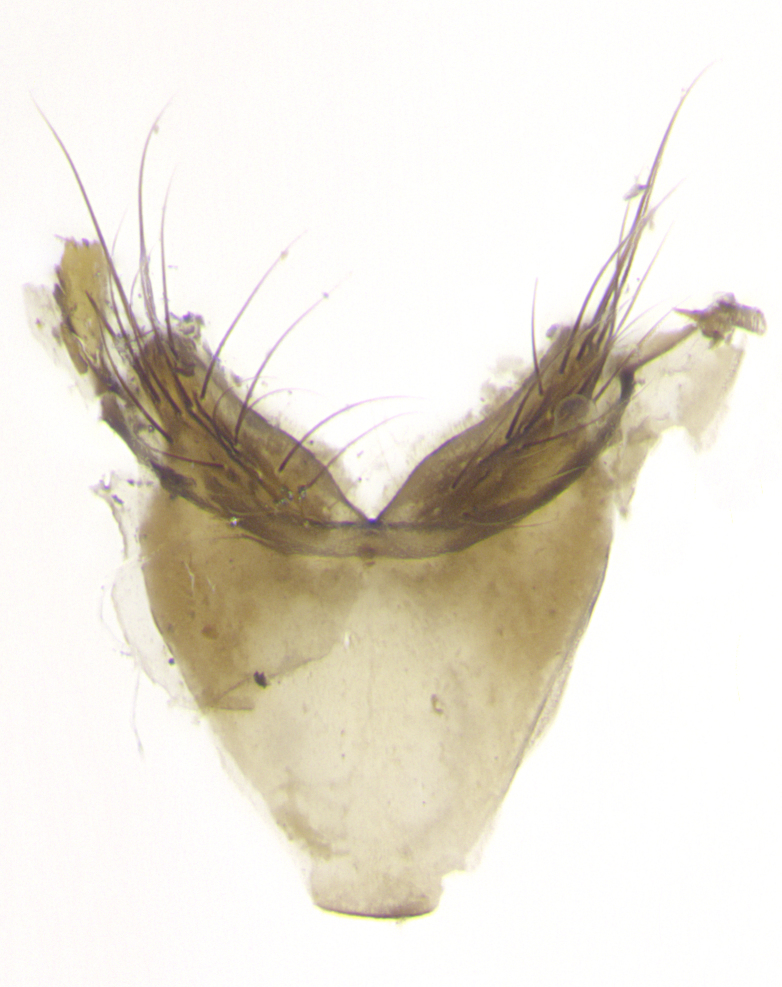
sternite 5, ventral view.

**Figure 44a.**
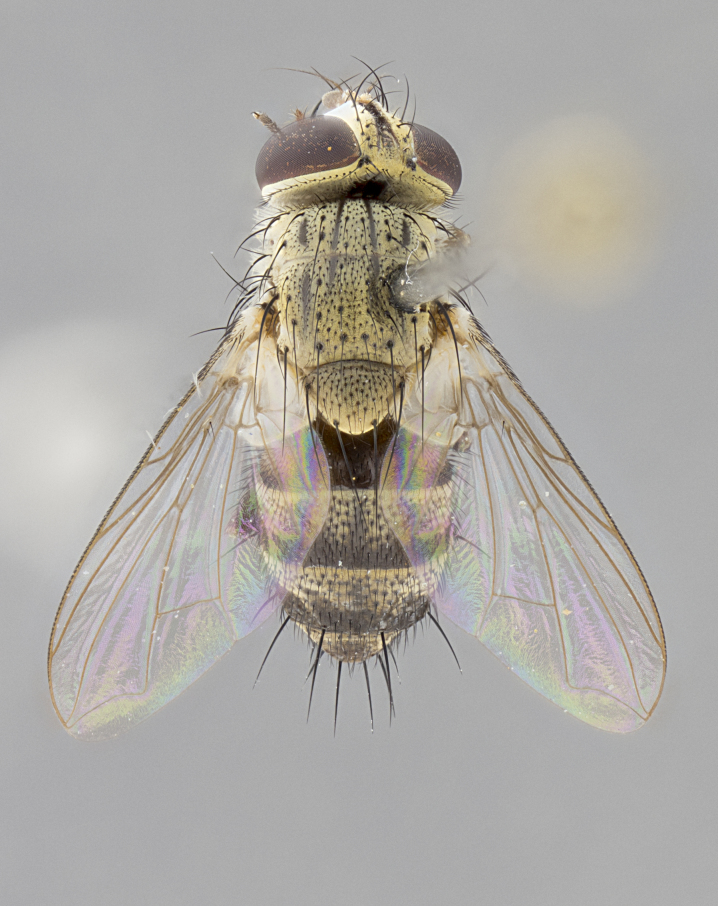
dorsal view.

**Figure 44b.**
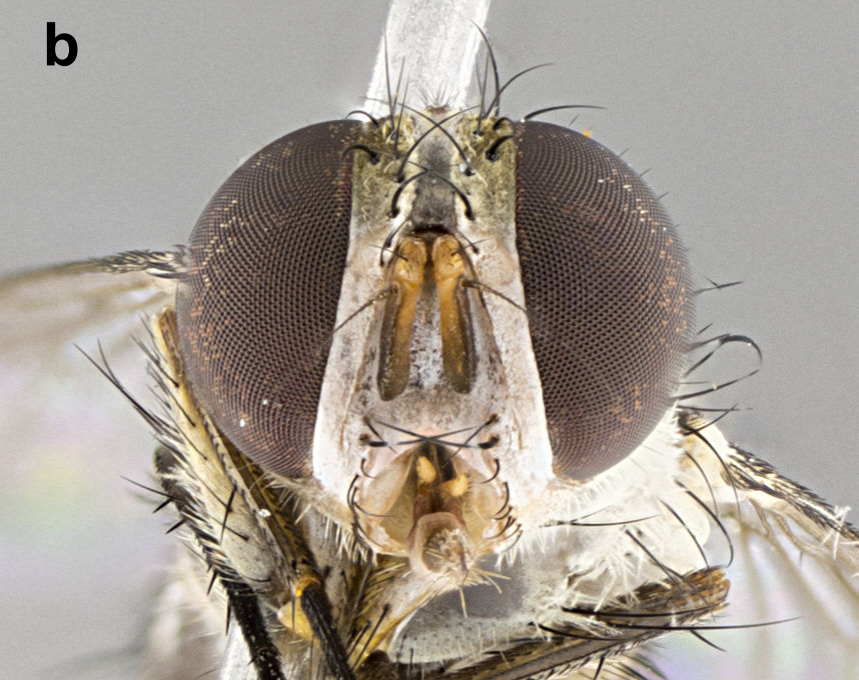
frontal view.

**Figure 44c.**
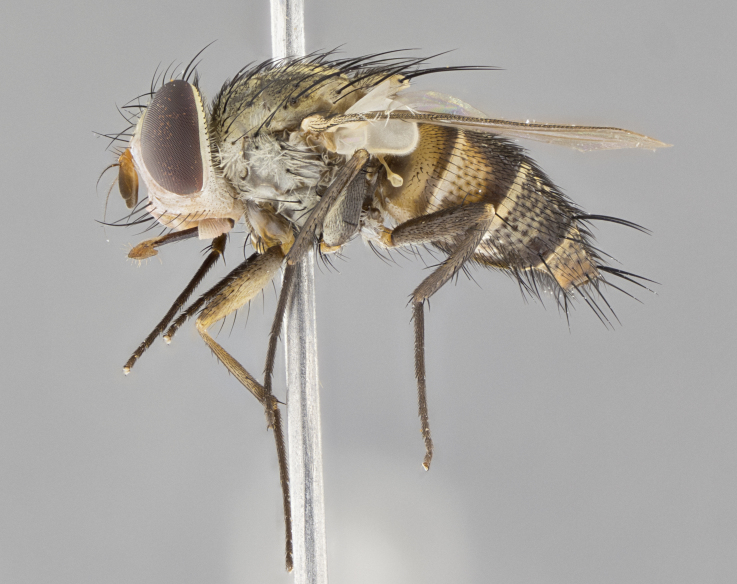
lateral view.

**Figure 44d.**
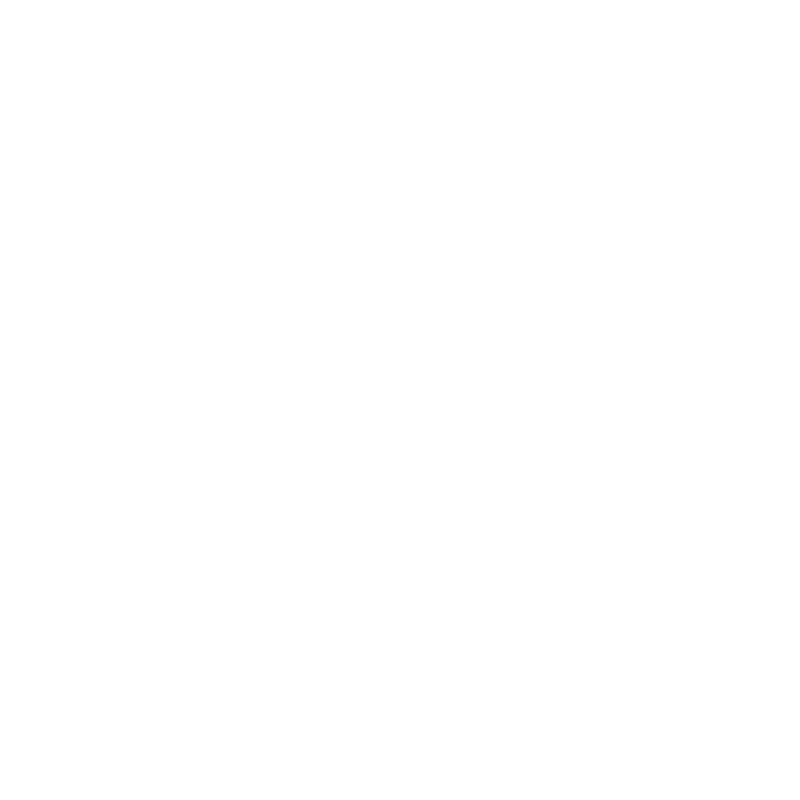


**Figure 45.**
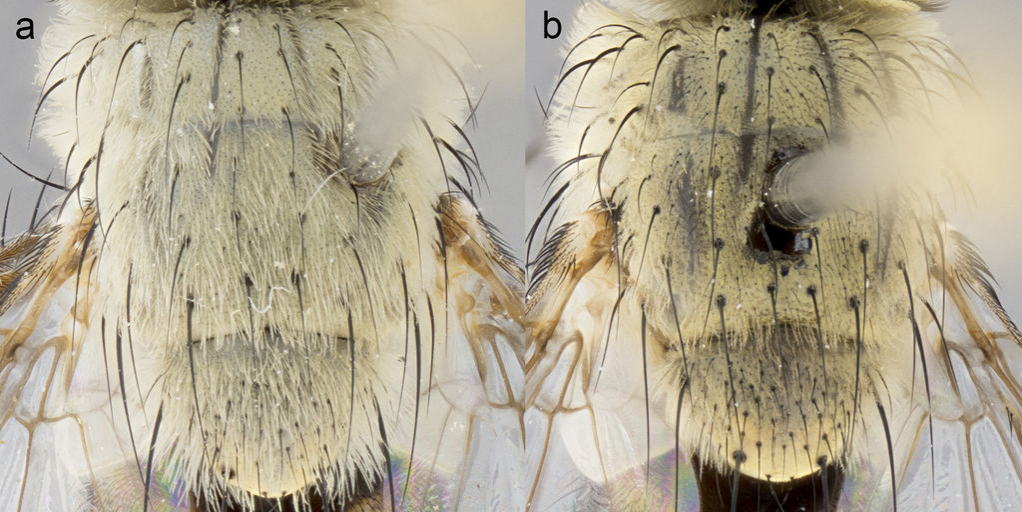
Disc of scutum with plumose hairs; **a.** *Telothyria fimbriata* **sp. n.**, **b.** *Telothyria harryramirezi* **sp. n.**

**Figure 46.**
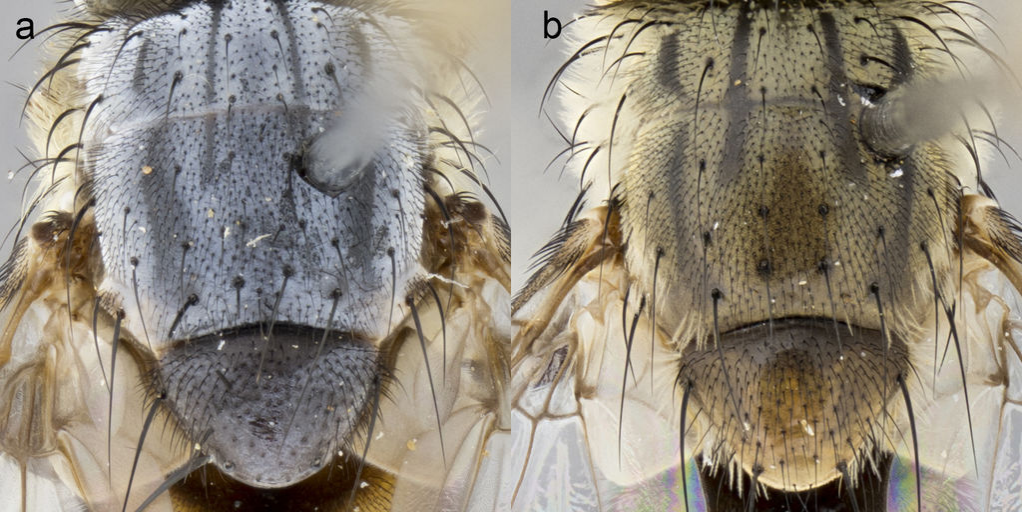
Plumose hairs absent from disc of scutum; **a.** *Telothyria cristata* **sp. n.**, **b.** *Telothyria eldaarayae* **sp. n.**

**Figure 47.**
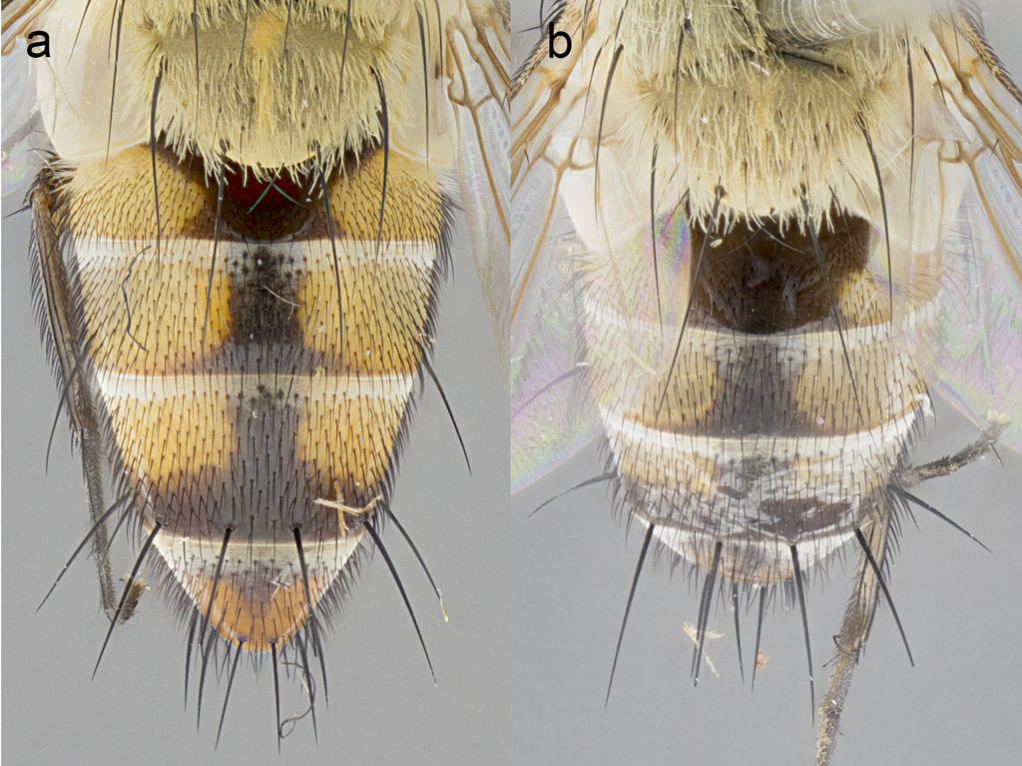
Detail showing difference in coloration of dorsomedial ST1+2; **a.** *Telothyria aidani* **sp. n.**; **b.** *Telothyria alexanderi* **sp. n.**

**Figure 48a.**
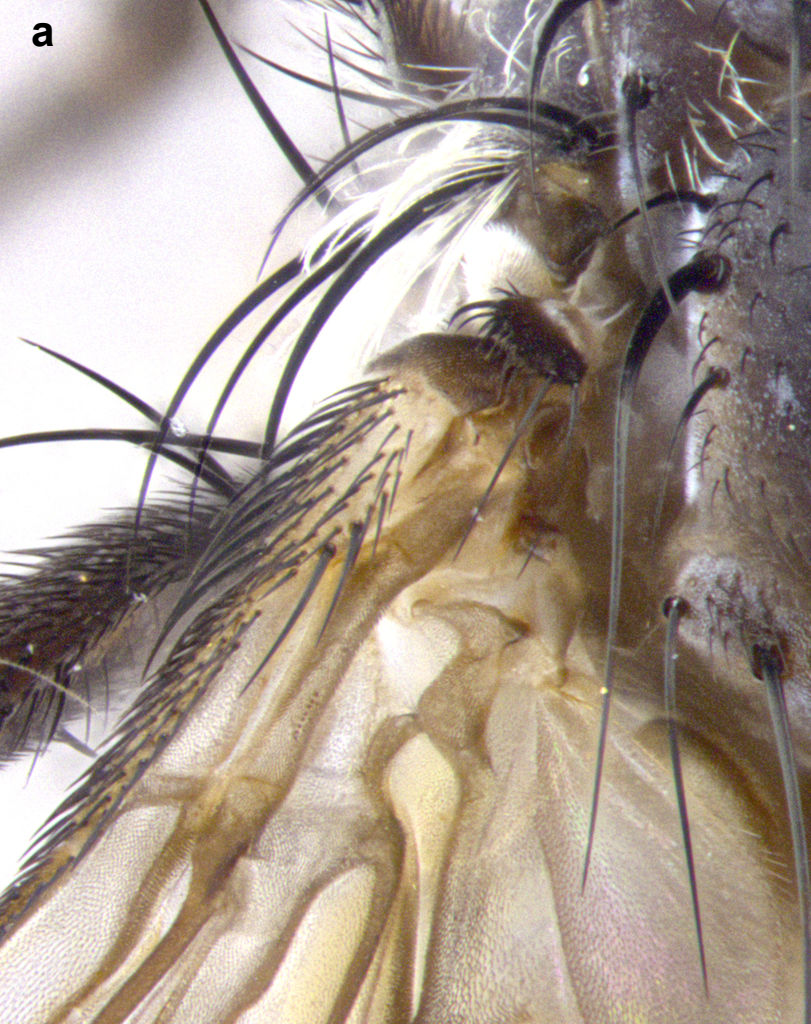
*Telothyria grisea* **sp. n.** displaying the brown color basicosta.

**Figure 48b.**
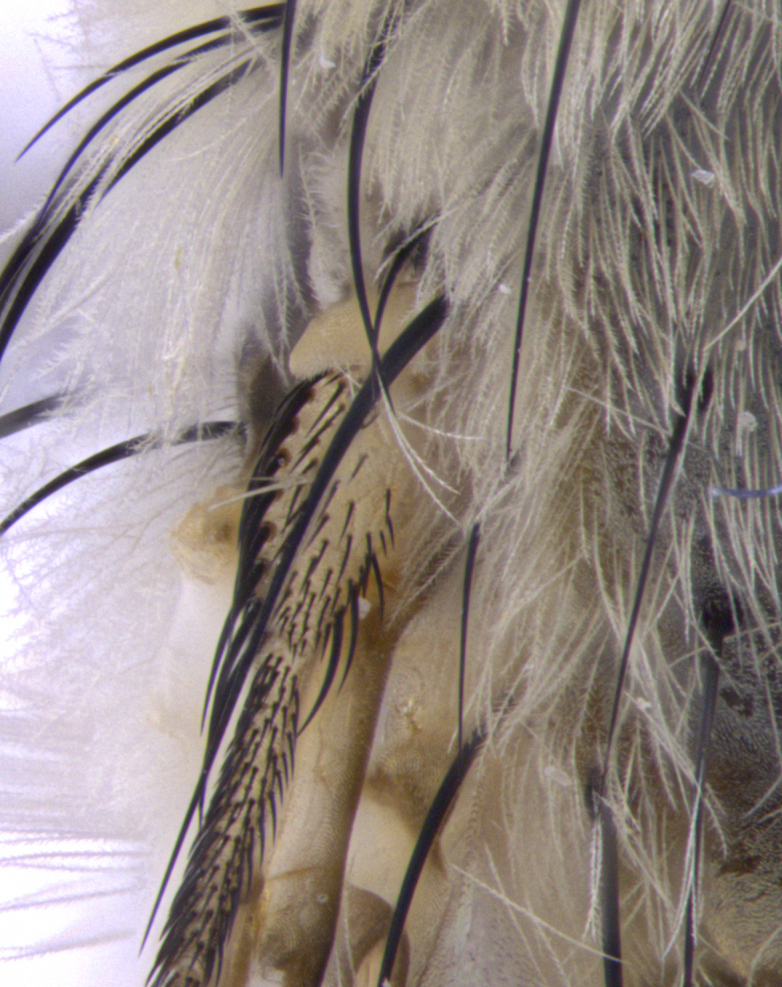
*Telothyria fimbriata* **sp. n.** displaying the ivory white color basicosta.

**Figure 48c.**
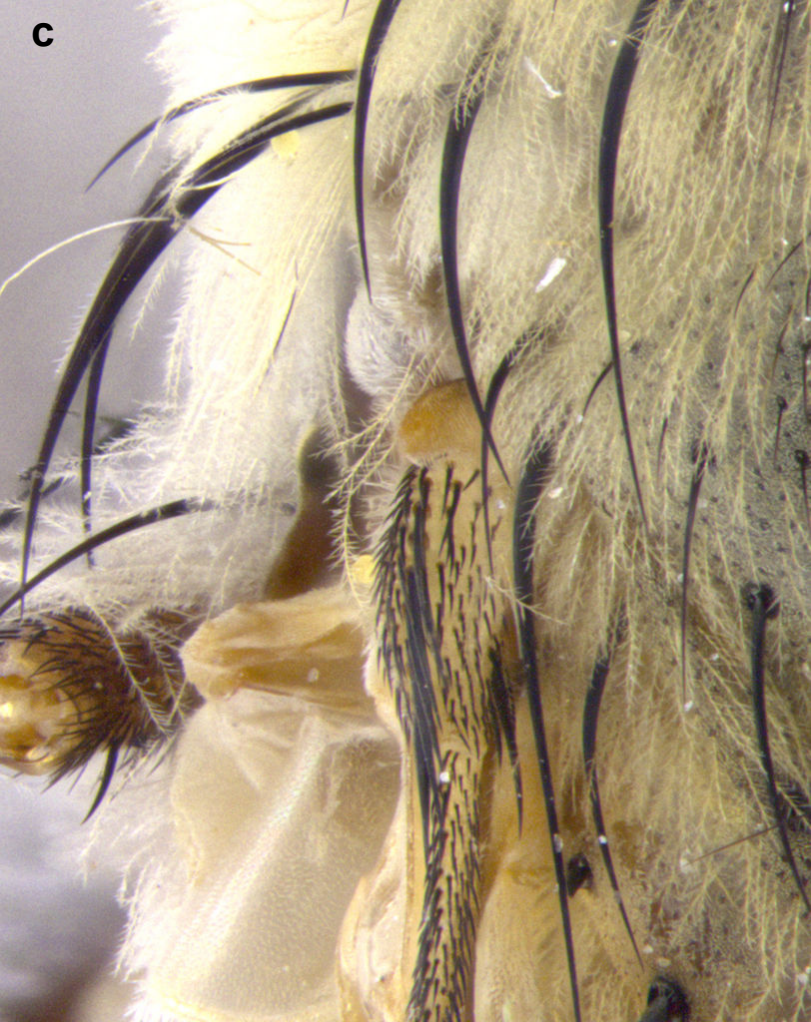
*Telothyria auranticrus* **sp. n.** displaying the beige color basicosta.

**Figure 49a.**
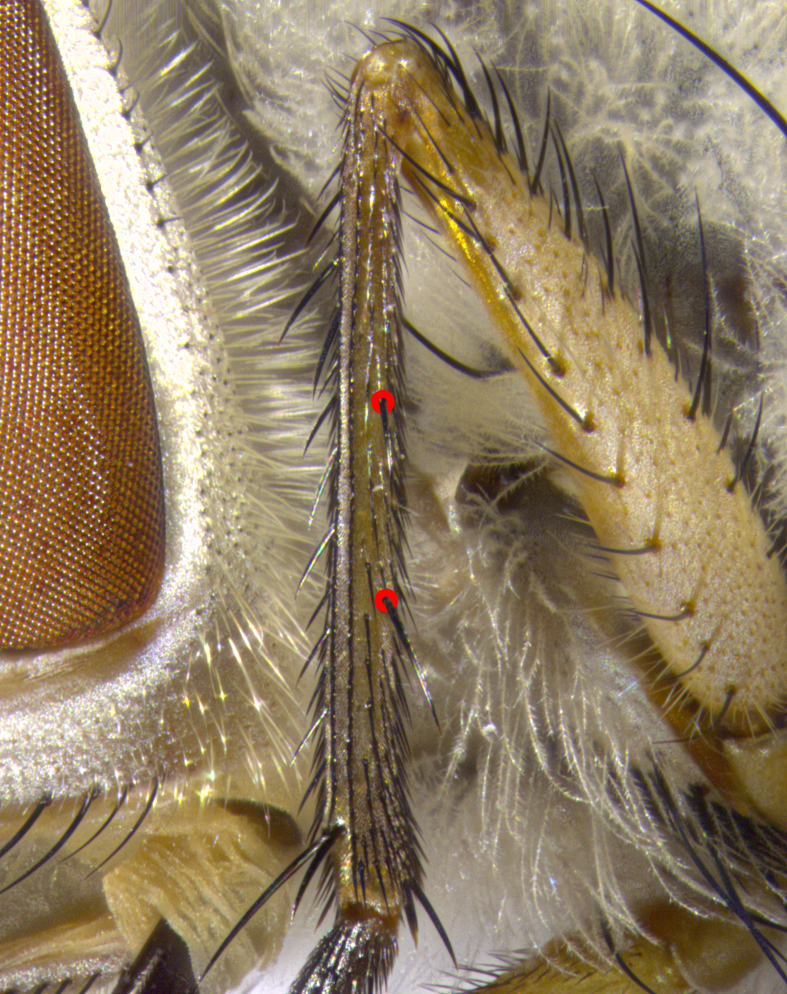
*Telothyria manuelpereirai* **sp. n.**

**Figure 49b.**
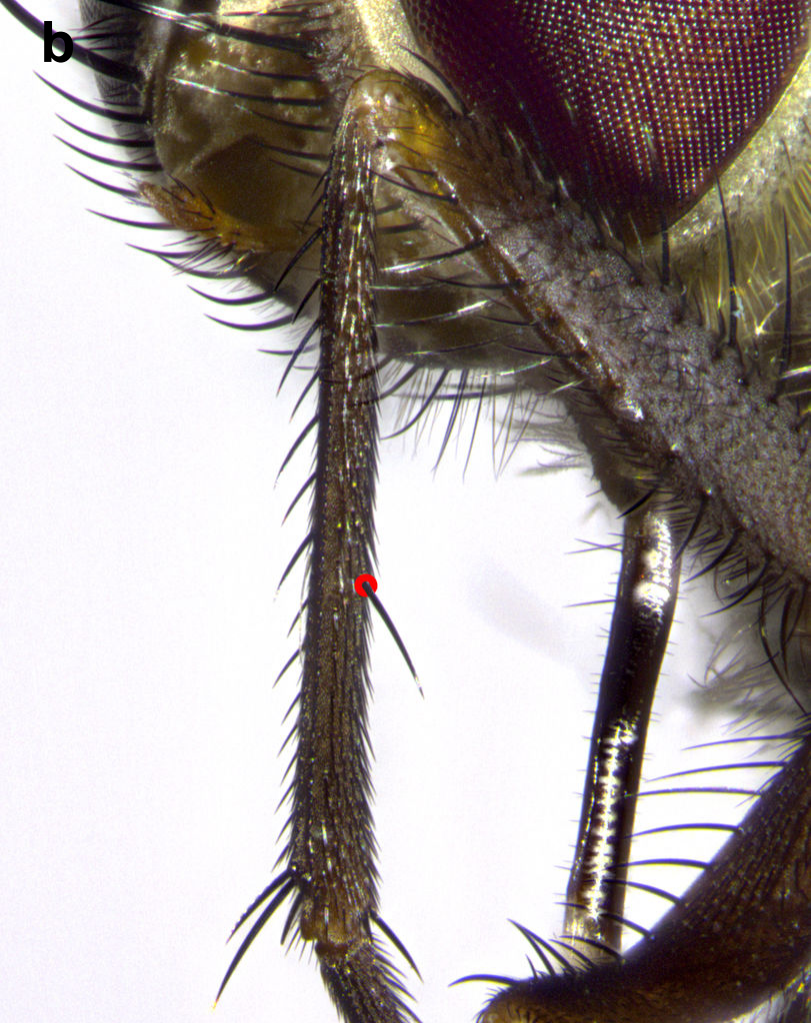
*Telothyria cristata* **sp. n.**
